# Transcriptional profiling of putative human epithelial stem cells

**DOI:** 10.1186/1471-2164-9-359

**Published:** 2008-07-30

**Authors:** Salih S Koçer, Petar M Djurić, Mónica F Bugallo, Sanford R Simon, Maja Matic

**Affiliations:** 1 Department of Biochemistry and Cell Biology, State University of New York at Stony Brook, Stony Brook, NY, USA; 2 Department of Pathology, State University of New York at Stony Brook, Stony Brook, NY, USA; 3 Department of Electrical and Computer Engineering, State University of New York at Stony Brook, Stony Brook, NY, USA

## Abstract

**Background:**

Human interfollicular epidermis is sustained by the proliferation of stem cells and their progeny, transient amplifying cells. Molecular characterization of these two cell populations is essential for better understanding of self renewal, differentiation and mechanisms of skin pathogenesis. The purpose of this study was to obtain gene expression profiles of alpha 6^+^/MHCI^+^, transient amplifying cells and alpha 6^+^/MHCI^-^, putative stem cells, and to compare them with existing data bases of gene expression profiles of hair follicle stem cells. The expression of Major Histocompatibility Complex (MHC) class I, previously shown to be absent in stem cells in several tissues, and alpha 6 integrin were used to isolate MHCI positive basal cells, and MHCI low/negative basal cells.

**Results:**

Transcriptional profiles of the two cell populations were determined and comparisons made with published data for hair follicle stem cell gene expression profiles. We demonstrate that presumptive interfollicular stem cells, alpha 6^+^/MHCI^- ^cells, are enriched in messenger RNAs encoding surface receptors, cell adhesion molecules, extracellular matrix proteins, transcripts encoding members of IFN-alpha family proteins and components of IFN signaling, but contain lower levels of transcripts encoding proteins which take part in energy metabolism, cell cycle, ribosome biosynthesis, splicing, protein translation, degradation, DNA replication, repair, and chromosome remodeling. Furthermore, our data indicate that the cell signaling pathways Notch1 and NF-κB are downregulated/inhibited in MHC negative basal cells.

**Conclusion:**

This study demonstrates that alpha 6^+^/MHCI^- ^cells have additional characteristics attributed to stem cells. Moreover, the transcription profile of alpha 6^+^/MHCI^- ^cells shows similarities to transcription profiles of mouse hair follicle bulge cells known to be enriched for stem cells. Collectively, our data suggests that alpha 6^+^/MHCI^- ^cells may be enriched for stem cells. This study is the first comprehensive gene expression profile of putative human epithelial stem cells and their progeny that were isolated directly from neonatal foreskin tissue. Our study is important for understanding self renewal and differentiation of epidermal stem cells, and for elucidating signaling pathways involved in those processes. The generated data base may serve those working with other human epithelial tissue progenitors.

## Background

Skin constantly renews throughout adult life. The proliferative compartment of epidermis is confined to the basal layer, where it harbors stem cells, and their progeny, transient amplifying cells [1-3]. Stem cells are predominantly quiescent *in situ*. Transient amplifying cells are more rapidly cycling, and after dividing for a limited period of time cease to proliferate and undergo terminal differentiation while moving towards the skin surface [4]. Slow cycling stem cells of the murine epidermis were identified by the retention of BrdU or [^3^H]thymidine after prolonged chase [5-9]. Research aimed at isolating stem cells directly from human tissue has to be based on different methodological approaches. Putative human interfollicular stem cells have been enriched based on the expression of β1 integrin [10], transferin receptor [11], connexin 43 [12], an isoform of *CD133 *[13] and desmosomal proteins [14]. However, it has not been determined whether these cells represent distinct populations, or belong to overlapping cell subsets. Databases generated from gene expression profiles of stem cells provide useful resources in evaluating putative stem cell populations. The lack or low levels of MHCI molecules have been reported in stem cells of several tissues [15-20]. Downregulation of MHCI transcripts has been observed in mouse hair follicle stem cells [21]. We have previously isolated a subpopulation of human basal keratinocytes with low/negative MHCI expression (α6^+^/MHCI^-^) [22]. Cells with α6^+^/MHCI^- ^phenotype constitute a small fraction of the basal layer (0.5–2%) as determined by flow cytometry [22]. We found that α6^+^/MHCI^- ^cells were keratinocytes as they expressed keratin 14 (K14). The α6^+^/MHCI^- ^cells exhibited characteristics attributed to stem cells: they were clonogenic *in vitro*, relatively small, and had low granularity [22]. In the present work we employ microarray technology, to report global transcriptional profiles of two cell populations: the basal cells that express MHCI, α6^+^/MHCI^+ ^(transient amplifying cells) and the basal cells that have low/negative MHCI expression, α6^+^/MHCI^- ^cells, (putative stem cells). Cells were isolated using fluorescence-activated cell sorter (FACS) directly from human epidermis. Further comparisons were made with published data of hair follicle stem cell gene expression profiles.

In addition, using flow cytometry we have analyzed the expression of nuclear proliferation antigen, Ki67. Our data indicate that MHCI^- ^cells are quiescent *in situ*. Following FACS sorting, α6^+^/MHCI^-^and α6^+^/MHCI^+ ^cells were grown at clonal densities to determine their colony forming efficiency (CFE). The analysis of CFEs in the initial, primary, culture and in the first passage indicate that α6^+^/MHCI^- ^cells have higher proliferative potential than α6^+^/MHCI^+ ^cells, another feature attributed to stem cells.

## Results and discussion

Skin is the largest and most accessible organ in the body. The differentiation axis of the interfollicular epidermis is spatially well defined: the basal layer contains proliferating cells, while suprabasal layers, stratum spinosum, stratum granulosum, and stratum corneum harbor post-mitotic, differentiating keratinocytes [23,24]. These features facilitate the analysis of cells at the specific differentiation stage. Like all self-renewing tissues, epidermis contains stem cells, which are located in the stratum basale. Several proteins have been suggested as markers for keratinocyte stem cell enrichment [10-14]. We have previously described a basal keratinocyte population that lacks gap junction protein Cx43 in human and mouse epidermis [12]. We have shown that Cx43 negative cells co-localize with label-retaining cells, hair follicle bulge stem cells [6-8]. Cx43 negative keratinocytes comprise about 10% of human basal keratinocytes and are blast like, small and have low granularity as determined by flow cytometry. Cells in the limbus of the eye, the region of the corneal epithelium that contains stem cells, were also shown to lack Cx43 [25]. In searching for additional markers that can be used to obtain viable cells, we isolated a subset of Cx43 negative keratinocytes characterized by low/negative expression of MHCI that comprised up to 2% of basal epidermal cells [22]. It was believed that almost all nucleated cells express MHCI [26]. Recently, however, stem cells of several tissues were shown to lack MHCI expression [15-21]. Molecules encoded by MHC are involved in self/non-self discrimination in vertebrates. MHCI molecules bind endogenously derived peptides and stimulate a distinct branch of the adaptive immune system mediated by CD8^+ ^T cells. The human MHC termed HLA (Human Leukocyte Antigen) encodes three classical polymorphic class I genes: HLA A, B, and C. To isolate transient amplifying cells (α6^+^/MHCI^+^) and presumptive stem cells (α6^+^/MHCI^-^), we used antibodies against α6 integrin, a basal cell marker, in combination with antibodies against β2 microglobulin, the light chain of MHCI molecule. Previously we have shown that similar results were obtained regardless of whether antibodies to MHCI heavy chain, or antibodies against β2 microglobulin were used [22].

### α6^+^/MHCI^- ^cells are quiescent *in situ*, yet in culture display higher proliferative potential

**Figure 1:**
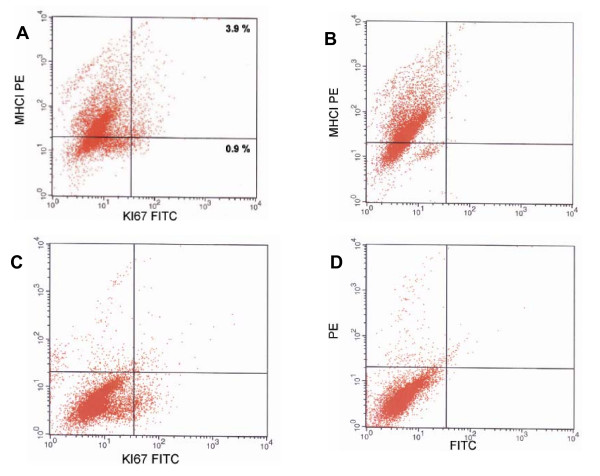
**MHCI negative cells express low levels of Ki67**. (A) A representative flow cytometric analysis of the expression of proliferation antigen Ki67 in MHCI positive, and MHCI negative cells. Gates were set using isotype control antibodies and single color control antibodies. In this experiment 3.9% of MHCI positive cells express Ki67, while only 0.9% of MHCI negative cells express Ki67. Although the exact values of proliferating population may vary from experiment to experiment the ratio of MHCI positive and MHCI negative proliferating populations stay constant. This result demonstrate quiescent nature of MHCI negative cells. (B) Single positive isotype control for PE. (C) Single positive control for FITC. (D) Secondary control.

During tissue homeostasis stem cells are infrequently dividing; thus, and one of the characteristics of stem cells is their quiescence *in situ*. To determine the proliferative status of MHCI^- ^and MHCI^+^populations, we analyzed the expression of nuclear proliferation antigen Ki67, which is a marker for actively cycling cells. The data presented reflect keratinocyte proliferation since in normal epidermis, non keratinocytes are found to be non-cycling [27,28]. Although the absolute values of Ki67 may vary depending on the total number of gated cells, the ratio of Ki67 expressing MHCI^- ^and MHCI^+ ^cells is held constant. Flow cytometric analysis showed that MHCI^+ ^cells expressed more than four time higher levels of Ki67 than MHCI^- ^cells (Figure 1). Only 0.9% of MHCI^- ^cells expressed Ki67, while 3.9% of MHCI^+^cells were in the cell cycle. Given the low percentages of MHCI^-^cells in the basal layer, it is clear that the bulk of cell production in the epidermis is accomplished through divisions of transient amplifying cells, a finding which is in accordance with the established view of epidermal homeostasis.

**Figure 2:**
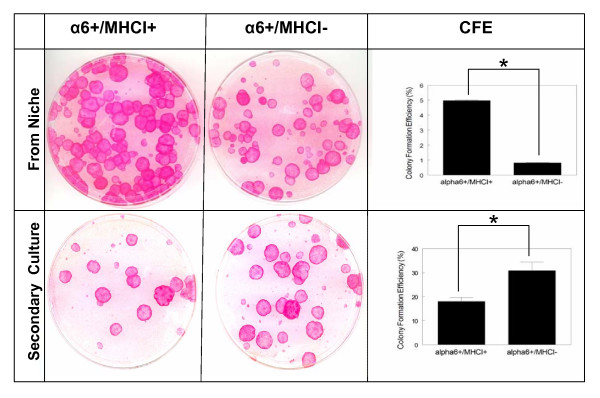
**Comparison of colony forming efficiencies of α6^+^/MHCI^+ ^and α6^+^/MHCI^- ^cells**. A representative experiment of the colony forming efficiency (CFE) of sorted α6^+^/MHCI^+ ^cells and α6^+^/MHCI^- ^cells cultured on 3T3 fibroblast feeder layer. Primary culture of α6^+^/MHCI^- ^cells exhibits lower CFE than α6^+^/MHCI^+ ^cells. However, secondary culture of α6^+^/MHCI^- ^cells exhibits higher CFE than α6^+^/MHCI^+ ^cells (*P < 0.001). The CFE of α6^+^/MHCI^+ ^cells increased ~3.6× from primary to secondary culture, while it increased ~38× for α6^+^/MHCI^-^. These results indicate higher proliferative potential of α6^+^/MHCI^- ^cells compared to α6^+^/MHCI^+ ^cells. Cells directly sorted by FACS (from niche, i.e. primary cultures) were seeded at the following concentrations: α6^+^/MHCI^+ ^cells, 3,000 cells per plate; α6^+^/MHCI^- ^cells, 10,000 cells per plate. It must be noted that even though higher cell number was plated for α6^+^/MHCI^- ^cells in the primary cultures, the CFE of these cells was lower than the CFE of α6^+^/MHCI^+ ^cells. In secondary cultures equal numbers of cells were plated (100 cells per plate) for both, α6^+^/MHCI^+ ^cells, and α6^+^/MHCI^- ^cells.

High proliferative potential is another feature of stem cells. One way of assessing proliferative potential of keratinocytes is to analyze their colony forming efficiency (CFE) [10]. To assess proliferative potential of the two cell populations' colony forming efficiency (CFE) was analyzed in the primary and secondary cultures. Sorted cells were seeded at clonal densities and colonies formed evaluated after two weeks. In primary cultures, α6^+^/MHCI^- ^cells exhibited lower colony forming efficiency than α6^+^/MHCI^+ ^cells (Figure 2). However, in secondary cultures α6^+^/MHCI^- ^showed higher CFE than α6^+^/MHCI^+ ^cells. From primary to secondary culture, an increase of 38 times was observed in the CFE of α6^+^/MHCI^- ^cells, while the CFE of α6^+^/MHCI^+ ^cells increased only 3.6 times (Figure 2). Previously, lower initial CFE was observed in limbal epithelial stem cell [29]. Our data indicate that α6^+^/MHCI^- ^cells have higher proliferative potential than α6^+^/MHCI^+ ^cells, another characteristic attributed to stem cells. The majority of available data regarding CFE are from secondary or tertiary keratinocyte cultures [10,30]. In the secondary cultures, α6^+^/MHCI^- ^cells did display higher CFE than α6^+^/MHCI^+ ^cells, which is an indication of a higher proliferative potential of the α6^+^/MHCI^- ^cells [10]. The lower CFE of α6^+^/MHCI^- ^cells compared to α6^+^/MHCI^+ ^cells that we recorded in our primary cultures may be due to the time needed for stem cells to transit from a quiescent to a proliferative state.

### Gene expression profile indicates that α6^+^/MHCI^- ^cells exhibit properties of stem cells

Microarray profiles of stem cells and their progeny provide a global view into differences of expression of a large number of genes and enable analyses of molecular processes involved in self renewal, proliferation and differentiation. Gene expression profiles of hair follicle bulge stem cells were recently reported [9,21,31-33], yet until now no data are available with regard to human interfollicular keratinocyte stem cells. We report on transcriptional profiles of putative human keratinocyte stem cells and their immediate progeny, transient amplifying cells. Global gene expression profile was obtained from sorted α6^+^/MHCI^- ^cells and α6^+^/MHCI^+ ^cells using DNA microarray chips. We identified a comprehensive list of differentially expressed genes. Notably, all of the MHCI genes were downregulated in α6^+^/MHCI^- ^cells, thus confirming the successful separation of α6^+^/MHCI^+ ^and α6^+^/MHCI^- ^cells. The data also show that expression of MHCI proteins in keratinocytes is regulated at the transcriptional level. The HLA-E transcript is downregulated in α6^+^/MHCI^- ^cells confirming our previous results obtained at the protein level [22]. The expression of non-classical HLA molecules is thought to protect cells that lack classical HLA expression from lysis by NK cells. At present, it is not known what mechanisms protect presumptive stem cells, MHCI^- ^cells, from attack by NK cells, especially since MHCI^- ^cells do not express detectable levels of non-classical HLA-E and HLA-G molecules [22].

We found that most of the mRNAs of genes encoding cellular receptors and other cell surface molecules were more abundant in α6^+^/MHCI^- ^cells than in α6^+^/MHCI^+ ^cells (see Additional file 1). Conversely, mRNAs of genes encoding proteins that take part in ribosome biosynthesis, RNA splicing, translation, protein degradation, and energy metabolism were more abundant in α6^+^/MHCI^+ ^cells (see Additional file 1). These findings are consistent with reports by other investigators who demonstrated that stem cells are characterized by few ribosomes and mitochondria (features related to undifferentiated state of stem cells) but contain a large numbers of receptors [9,34].

**Figure 3:**
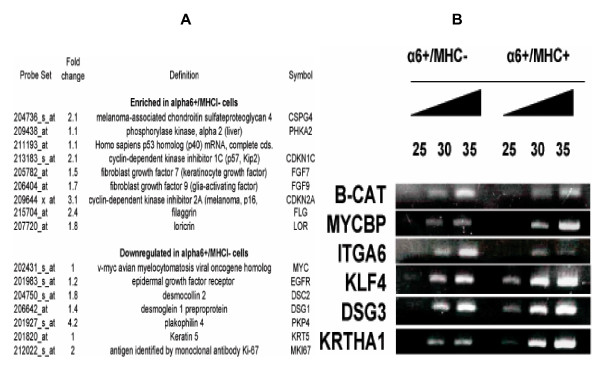
**Expression of interfollicular epidermal stem cell markers in MHCI negative cells**. (A) Differentially expressed genes identified by microarray analysis that were discussed in the text. The numbers shown are in fold change in log2 scale and are the highest score for the gene. (B) The differentially expressed genes identified by microarray analysis and/or known epidermal SC markers were confirmed using semi-quantitative RT-PCR. PCR was run for 25, 30, and 35 cycles. β-catenin mRNA, which did not show significant change of expression in the two cell populations was used as a control.

In reference to stem cells quiescence, it should also be noted that transcripts of *CDKN1C *and *CDKN2A *whose products are cyclin-dependent kinase inhibitors were enriched in α6^+^/MHCI^- ^cells (Figure 3A). The product of *CDKN1C*, p57^Kip2^, one of the Cip/Kip family members, is tightly associated with inhibition of proliferation of human interfollicular keratinocytes [35], and is upregulated in hair follicle bulge [31], while the product of *CDKN2A*, a 16 kDa protein p16^INK4a ^imposes a G1 cell cycle arrest [36,37]. Furthermore, in addition to the low protein level of Ki67 observed in MHCI negative population (Figure 1), the transcript of Ki67 was less abundant in α6^+^/MHCI^- ^cells (Table 1, and Additional file 1). Conversely, transcripts of genes encoding proteins that are related to cell proliferation such as cyclins, proteins involved in chromosome remodeling, DNA replication and repair were preferentially enriched in α6^+^/MHCI^+ ^cells (Table 1, and Additional file 1).

**Table 1:** Selected genes enriched in α6^+^/MHCI^+ ^cells compared to α6^+^/MHCI^- ^cells.


**Factors downregulated in HHFSC**	CDC2 (1.7), PRC1 (1.6), RRM2 (2.3), ZWINT (1.1), KPNA2 (1.1), FEN1 (1.1), TOP2A (4.5), TYMS (1), RHEB2 (1.2).

**Factors downregulated in MHFSC**	THBD (1.1), RBMS1 (4.2), MYC (1), ABCD4 (1.8), UGP2 (1.6), IGFBP3 (1.1), WNT3 (3.8), WNT4 (3.6), DSC2 (1.8), HSPA1A (2.4), CKMT1 (1), RORA (2.4), ANXA1 (1.4), ANXA2 (1.8), COL17A1 (2.2), IL6ST (1.7), TGFBR2 (1.3), LGALS7 (1.8), KRT5 (1), KRT15 (1.2), SERPINB2 (1), SERPINB7 (1.8), GPR87 (1.3), TGFBI (1.3), VSNL1 (1.2), CLCA2 (1.7), E48 (2), MKI67 (2), CKS2 (1.1), PRC1 (1.6), HLA-B (2.4), HLA-B39 (2), HLA-C (1.1), HLA-Cw*1701 (2.9), HLA-E (1.6), D6S81E (1.7), CCNB1 (2.2), CCNB2 (1.7), CCND1 (1.3), CCND2 (3.3), CHEK1 (1.7), CDC6 (1.6).

**TGFβ/BMP-repressed factors**	MYC (1), MT1F (1.6), MT1G (1.1), UGP2 (1.6), CCND2 (3.3), KRT15 (1.2), VIL2 (3.3), CKMT1 (1), EHF (2.2), NIBAN (1.7), VAMP8 (3.2), TNNI2 (1.4), KLF4#.

**WNT-induced factors**	MYC (1), CCNB1 (2.2), CCND1 (1.3), CCND2 (3.3), JUN (1), CKS2 (1.1), MKI67 (2), BIRC5 (2.5), TNNT1 (3.5), TNNI2 (1.4), MBNL (1.4), IGFBP3 (1.1), PTTG1 (1.1), EGFR (1.2), EMP1 (2.1), CSPG6 (2.8), CALD1 (1.2), BTEB2 (1.8), DUSP6 (3.5), FOS (2.2), JWA (1.2), HSP70 (1.4), KRT5 (1), GSTM3 (1.3), NCOA3 (1.3), OSF2 (1.1), SDC4 (1), ELF1 (1.6), HMG14 (1), TRA1 (2.3), CDC6 (1.6), DHFR (1.1), ADE2H1 (2), NSAP1 (1.2), MCM4 (3.4), KPNB3 (1.2), KRTHA1 (0.5)#, MYCBP (0.6)#.

**NF-κB-induced factors**	MYC (1), MT1F (1.6), MT1G (1.1), CCND1 (1.3), FTH1 (1.7), IGFBP1 (2), HMG14 (1), AKR1C2 (1), UGCG (1.1), GBP-1 (1.2), ATF3 (3.3), SDC4 (1), PTGS2 (1.3), BMP2 (1.3), DUSP6 (3.5), MGP (1.1), FOS (2.2), MCP-1 (5.4), PIG7 (2), MIF (1.8), PMAIP1 (1.3), LSR68 (3.6), TNFS10 (2.7), HLA-B (2.4), HLA-B39 (2), HLA-C (1.1), HLA-Cw*1701 (2.9), HLA-E (1.6), D6S81E (1.7), HSPB1 (3).

**Figure 4:**
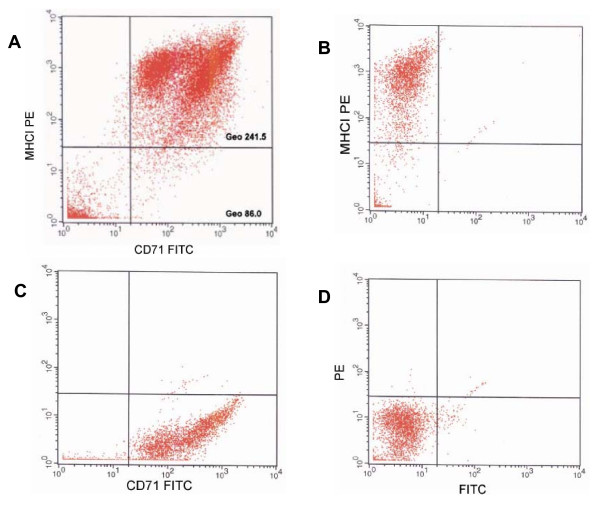
**CD71 expression in MHCI negative and MHCI positive cells**. (A) showing A representative flow cytometry analysis of the expression of MHCI and CD71 indicating that epidermal cells that exhibit lack/low expression of MHCI also exhibit lower expression of CD71 than cells that express high level of MHCI. Gates were set using isotype control antibodies and single color antibodies. The geometrical mean channel fluorescence of the populations is indicated. (B) Single positive control for PE. (C) Single positive control for FITC. (D) Isotype control.

Functional classification of some of the genes that are selectively upregulated ≥ 2 fold in α6^+^/MHCI^+ ^cells relative to α6^+^/MHCI^- ^cells (for the full list see Additional file 1). The numbers shown in parentheses are in log2 scale and are the highest score for that gene. Expression of the genes marked with (#) have been additionally verified (Figure 4).

Interestingly, type I IFN (IFN-α, and IFN-β) has been shown to inhibit cell proliferation by inducing G1 cell cycle arrest. It has been reported that interferon α (IFN-α) has antiproliferative effects on bone marrow stromal precursors, hepatic progenitor cells, and mesenchymal stem cells [39-41]. We observed enrichment of transcripts of the IFN-α family of proteins in α6^+^/MHCI^- ^cells as well as *STAT2*, the specific transducing activator of IFN-α transcription. This is the first report that suggests involvement of type I IFN in epidermal stem cell quiescence. Further studies are needed to determine whether IFN-α is synthesized by α6^+^/MHCI^- ^cells, whether its pathway is active and whether it contributes to α6^+^/MHCI^- ^cell quiescence (Table 1).

It has been reported that the components of the inositol phospholipid signaling system are present and that the system itself is active in murine embryonic stem cells [42]. In the present study, we demonstrate that several components of the inositol phospholipid signaling system are enriched in α6^+^/MHCI^- ^cells (Table 1). Nevertheless, the functional significance of this observation needs further investigation.

Among the transcripts enriched in α6^+^/MHCI^- ^cells, there were transcripts previously shown to be upregulated in cell population enriched for human interfollicular stem cells, such as α6 integrin [11], melanoma-associated chondroitin sulfate proteoglycan [43], phosphorylase kinase α2 [43], and the transcript of p53 homolog *p51/p73L/p63/p40 *[44] (Figure 3A and Additional file 1). Using flow cytometry we found that MHCI^- ^cells expressed lower level of transferrin receptor (*CD71*), a negative marker used to enrich for interfollicular epidermal stem cells [11], when compared to MHCI^+ ^cells (Figure 4). It should also be noted that although differences in the expression of *CD71 *transcript were lower than 2 fold, *CD71 *mRNA was downregulated in α6^+^/MHCI^- ^cells compared to α6^+^/MHCI^+ ^cells consistently in both arrays (see Additional file 2).

Among the transcripts enriched in α6^+^/MHCI^+ ^cells, there were mRNAs of genes whose products are expressed at low levels in cell population enriched for human interfollicular stem cells, such as desmosomal proteins including desmoglein 3 (*DSG3*) [14], as well as the proliferation associated transcription factor *c-Myc*, found to be expressed at the lower levels in cultured human interfollicular stem cells [45] (Figures 3A, 3B and Additional file 1). Moreover, it has been reported that epidermal growth factor receptor (EGFR) signaling is downregulated in putative human interfollicular stem cells [43]. In accordance with this observation, we found that *EGFR *itself was downregulated in α6^+^/MHCI^- ^cells (Fig. 3A and Additional file 1).

Most notably, we present results that demonstrate that putative stem cells have lower expression of mRNAs encoding proteins that take part in energy metabolism, which can explain how stem cells can be quiescent and at the same time maintain small size (see Additional file 1).

### Comparison of α6^+^/MHCI^- ^and α6^+^/MHCI^+ ^microarray databases with the transcriptional profiles of human and mouse hair follicle stem cells

We compared α6^+^/MHCI^- ^transcriptional profile with the published transcriptional profile of human hair follicle stem cells [32] and with four different sets of data of murine hair follicle stem cells [9,21,31,33]. We found that eleven genes, which were enriched in human hair follicle stem cells, were also enriched in α6^+^/MHCI^- ^cells (Table 1). Similarly, mRNAs of nine genes, which were downregulated in human hair follicle stem cells, were also downregulated in α6^+^/MHCI^- ^cells (Table 2). Nevertheless, the differences between the gene expression profiles of stem cells from the two human tissues, i.e. interfollicular and follicular, were also observed including the genes that demonstrated two fold difference in the expression (total of nine genes) (see Additional file 3).

**Table 2:** Selected genes enriched in α6^+^/MHCI^-^cells compared to α6^+^/MHCI^+^cells.


**mRNAs enriched in HHFSC**	TNRC9 (1.5), PHLDA1 (1), WIF-1 (6.6), RIG (1.6), DPYSL2 (1.9), DPYSL3 (1.4), GPM6B (2.5), FZD1 (1.3), NFATC1 (1.7), FST (2.3), DCT (2.5).

**mRNAs enriched in MHFSC**	LHX2 (5), TCF3 (1.5), WIF-1 (6.6), TRPS1 (2), BACH2 (1.1), LTBP1 (1.1), LTBP2 (1), ID2 (1), ID4 (1), DPYSL2 (1.9), DPYSL3 (1.4), GPR49 (2), GADD45G (1.2), ENPP1 (2.1), FBN2 (1), FOXC1 (1.7), VIM (1.7), DCT (2.5), MERTK (1.5), CRYM (1.5), CNR1 (1.4), SCD (2), TCF7 (1.1), CPE (1.9), EDNRB (2.8), AML1 (2.3), GPM6B(2.5), FGFR1 (2.5), CSPG2 (1.5), CSPG4 (2.1), NFATC1 (1.7), FYN (1.8), PRDM5 (1.5), ARG2 (1.4), MOX2 (2.9), DLX2 (1.8), ADAMTS5 (1.6), PHLDA1 (1), FZD7 (1.5), GUCY1B3 (1.5), TYR (2.1), COL1A2 (2), GPR64 (2.3), GSTM5 (1.2), PPAP2B (1.6), MITF (1.8), SNCAIP (1.2), SOX9 (1.9), MYH10 (1.1), MADH6 (1.6), INSIG1 (1.2), PLAT (1.4), PEG3 (2.8), NFIB (1.2), DAB2 (1.9), IGFBP5 (1.1), IGFBP7 (2.2), ITM2A (1.2), GFRA1 (1.5), ALCAM (1.6), BDNF (2.6), SDF1 (1.1), COL3A1 ((1), COL4A1 (1.9), COL4A2 (1.8), COL5A1 (1.1), COL6A1 (1.1), COL14A1 (1.3), HXB (1.3), ACTN1 (1.1), HPGD (1.1), APP (1.2), CTBP2 (1), MYO1B (1.1), SIAT4C (1.5), EFNB2 (1.3), EDG2 (1.2), CYP1B1 (3.5), PRLR (1.1), ALDH7A1 (1.1), DCAMKL1 (1.6), PAK3 (1.7).

**TGFβ/BMP signaling**	MADH3 (1.3), MADH6 (1.6), MADHIP (1.5), FST (2.3), BMP5 (5.1), BMP8 (2), BMP10 (1.2). BMP15 (1.3), INHBC (1.8).

**TGFβ/BMP-induced factors**	COL3A1 (1), COL4A1 (1.9), COL5A1 (1.1), COL6A1 (1.1), COL9A2 (1.6), COL11A1 (3.7), COL11A2 (1), COL14A1 (1.3), GPR56 (1.2), SOX4 (1.2), CLU (1.2), IQGAP1 (1.3), LMCD1 (2.7), SPRY4 (1.2), ITGB5 (1.1), LTBP1 (1), LTBP2 (1.1), GSN (1), PPAP2B (1.6), PEA15 (1.8), HEF1 (1.2), ID2 (1), ID4 (1), TGFB1l1 (1.3), FRZB (3.9), VCAM (1.2), FST (2), HXB (1.3), GSPG2 (2), AGC1 (1.5), THBS1 (2.3), APOE (1.1), MADH6 (1.6), NFATC1 (1.7), CKB (1.5), MMP9 (1.5), PLAUR (1.3), PLAT (1.4), APP (1.2), PTPRC (1.4), FZD1 (1.3), FYN (1.8), VAV1 (1.3), HCLS1 (1.1), TAL1 (1.1), LPL (1.3), BDNF (2.6), APBA3 (1.3), CDKN1C (2.1).

**WNT signaling**	FRZB (3.9), FZD1 (1.3), FZD4 (1.7), FZD7 (1.5), WIF-1 (6.6), DKK1 (1.9), DKK2 (1.7), TCF3 (1.5), TCF7 (1.1), TCFL2 (1.2), TLE1 (1), DAB2 (1.9), CTBP2 (1).

**WNT-repressed factors**	ACTN3(1.1), AKAP12 (1.1), CTSB (1.4), PLA2G7 (3.1), LTBP2 (1), DAB2 (1.9), FST (2.3), CLU (1.2), TCF3 (1.5), MEG3 (1.7), PPAP2B (1.6), LPL (1.3), ID4 (1), CDKN1C (2.1).

**Interferon signaling**	IFNA5 (2.9), IFNA6 (4), IFNA7 (1.4), STAT2 (1.5), CIS4 (1), SSI-3 (1.6).

**Inositol phospholipid signaling**	INPP4B (1.2), PIGB (1.2), PLCB4 (1), PLCE2 (1.4), KIAA0581 (2), GPLD1 (2.2), PIK3CD (1.2), NUDT4 (1.7), LOC51196 (1.7).

Functional classification of some of the genes that are selectively upregulated ≥ 2 fold in α6^+^/MHCI^- ^cells relative to α6^+^/MHCI^+ ^cells (for the full list see Additional file 1). The numbers shown in parentheses are in log2 scale and are the highest scores for that gene.

mRNAs of eighty-two genes, which were enriched in murine hair follicle stem cells, were also enriched in α6^+^/MHCI^- ^cells, while mRNAs of forty-one genes, which were downregulated in murine hair follicle stem cells, were also downregulated in α6^+^/MHCI^- ^cells (Tables 1, 2 and Additional file 4). Transcription factors *LHX2 *and *TCF3 *that were shown to maintain SC features [33,46], were among the genes that were upregulated in α6^+^/MHCI^- ^cells as well as in murine hair follicle stem cells. Upon screening of our microarray database for *TCF3 *targets [46], we found that transcripts of twelve genes reported to be upregulated by *TCF3 *were more abundant in α6^+^/MHCI^- ^cells and conversely transcripts of nine genes repressed by *TCF3 *were more abundant in α6^+^/MHCI^+ ^cells (see Additional file 5). Interestingly, both arrays that we performed showed that *LHX2 *was among the most upregulated mRNAs in α6^+^/MHCI^- ^cells, while *WIF-1 *mRNA, which was the most enriched mRNA in murine hair follicle stem cells according to one report [33], was the mRNA that showed the highest difference of expression between α6^+^/MHCI^- ^cells and α6^+^/MHCI^+ ^cells (97 fold).

### Uupregulation of transcripts encoding bone morphogenic factors in α6^+^/MHCI^- ^cells

It has been shown that TGF-β and the bone morphogenic factors are upregulated in epidermal stem cells [9,31,33]. Consequently, hair follicle stem cells are enriched with TGF-β/BMP targets [9]. In accordance with those findings, we observed that transcripts of several genes whose products are necessary for the activation of TGF-β/phospho-Smad pathway, such as genes necessary for latent TGF-β activation (*LTBP-1 *and *LTBP-2*), secreted activators (*BMP5, BMP8, BMP10, BMP15*), and transcriptional activators of TGF-β responses (*MADH3 *and *MADH6*), were enriched in α6^+^/MHCI^- ^cells compared to α6^+^/MHCI^+ ^cells (Table 1). In addition, transcripts of forty-nine target genes shown to be upregulated by TGF-β/BMP pathway were more abundant in α6^+^/MHCI^- ^cells. Conversely, transcripts of the genes whose expression is shown to be suppressed by TGF-β/phospho-Smad pathway, including transcription factors *c-Myc *and *KLF4*, were more abundant in α6^+^/MHCI^+ ^cells (Figure 3B, Table 2). Since TGFβ/BMP pathway is tightly associated with stem cell quiescence [31,47,48], upregulation of BMPs in α6^+^/MHCI^- ^cells might explain why these cells exhibit characteristics of quiescent cells.

### Transcripts of Wnt receptors and Wnt signaling inhibitors are enriched in α6^+^/MHCI^- ^cells

Wnt pathway plays an important role in hair follicle morphogenesis and cycling [49-51]. Researchers found that transcripts of several Wnt genes were downregulated in the mouse hair follicle bulge stem cells. In addition, higher levels of several genes that inhibit Wnt signaling pathway as well as higher levels of transcripts of the Wnt receptors were found in epidermal stem cells [9,21,32,33]. In the same cells, in general, targets of Wnt signaling (such as hair keratin, *KRTHA1*, nuclear proliferation antigen *Ki67 *[9,33,51] are downregulated. Consistent with these observations, we found that *WNT3 *and *WNT4 *were downregulated, while Wnt receptors *FZD1*, *FZD4*, *FZD7*, and the inhibitors of Wnt signaling pathway, *DAB2*, *TCF3*, *CTBP2*, *WIF1*, *DKK1*, and *DKK2 *were upregulated in α6^+^/MHCI^- ^cells (Table 1 and 2). Furthermore, transcripts of thirty eight genes that are known to be upregulated by Wnt signaling pathway were less abundant in α6^+^/MHCI^- ^cells, including *MYCBP *and type I hair keratin 1 (*KRTHA1*) (Figure 3B and Table 2). Also as expected to be found in stem cells, transcripts of genes that are downregulated by Wnt signaling pathway were found to be more abundant in α6^+^/MHCI^- ^cells. We found transcripts of fourteen such genes in α6^+^/MHCI^- ^cells (Table 2).

### Transcripts of the markers implicated in mammalian growth/differentiation are downregulated in α6^+^/MHCI^- ^cells compared to α6^+^/MHCI^+ ^cells

Stem cells are the least differentiated cells in their tissue of origin; therefore, transcription factors and signaling pathways that induce differentiation are expected to be downregulated in these cells. As mentioned above, we found that *MYC *was less abundant in α6^+^/MHCI^- ^cells compared to α6^+^/MHCI^+ ^cells (see Additional files 1 and 6). Upon screening of our microarray database for c-Myc targets, we found that the transcripts of sixty-one genes including *MYCBP *(Figure 3B and Additional file 6), which were reported previously to be upregulated by c-Myc, were upregulated in α6^+^/MHCI^+ ^cells. Conversely, transcripts of nineteen genes that were reported to be downregulated by c-Myc were upregulated in α6^+^/MHCI^- ^cells (see Additional file 6). Since many targets of c-Myc are involved in the ribosomal biogenesis, the downregulation of *MYC *may account for the observed downregulation of large numbers of genes that are involved in ribosomal biogenesis in α6^+^/MHCI^- ^cells (see Additional file 1), and may be an additional evidence that the downregulation of *MYC *in stem cells is related to their quiescent/undifferentiated state.

Similar to *c-MYC *expression, the expression of *KLF4 *(Kruppel-like factor 4), a transcription factor that is mainly expressed in the differentiating layers of epidermis [52], and *BMP2*, a member of bone morphogenetic protein family that is mainly expressed in proliferative basal and differentiated suprabasal keratinocytes [53], were downregulated in α6^+^/MHCI^- ^cells compared to α6^+^/MHCI^+ ^cells (Table 2 and Additional file 1).

**Figure 5:**
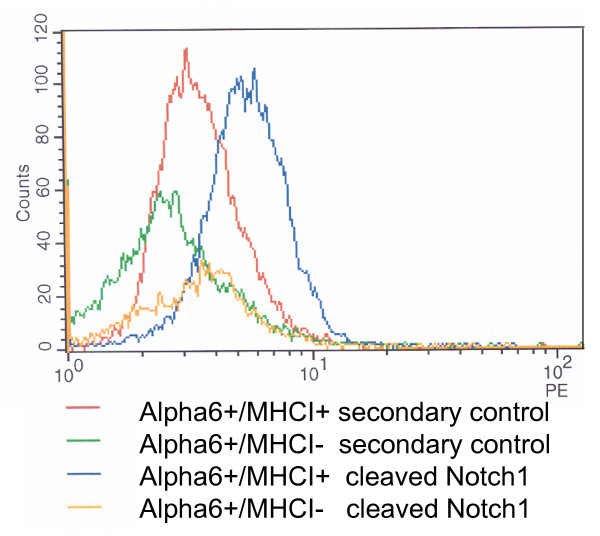
**Lack of Notch1 activity in MHCI negative cells**. Figure demonstrating lack of cleaved/active Notch1 expression in α6^+^/MHCI^- ^cells and its presence in α6^+^/MHCI^+ ^The data are obtained by flow cytometry analysis using an antibody specific for cleaved/active Notch1.

Notch1 signaling has been shown to stimulate differentiation in mammalian skin [54]. Transcripts of the genes, such as fillagrin, integrin alpha 6, loricrin, (*FLG, INTA6, LOR*) whose expression is repressed by Nothch1 signaling [54,55] were more abundant in α6^+^/MHCI^- ^cells (Figure 3A, and Additional file 1). Furthermore, expression of *KRT5*, which is upregulated by Notch1 signaling [54], was more abundant in α6^+^/MHCI^+ ^cells (Figure 3A, and see Additional file 1). We investigated whether the levels of Notch1 activity differ in α6^+^/MHCI^- ^and α6^+^/MHCI^+ ^cells. By using flow cytometry analysis with an antibody specific for cleaved/active Notch1, we could not detect any significant presence of cleaved/active Notch1 in α6^+^/MHCI^- ^cells contrary to α6^+^/MHCI^+ ^cells (Figure 5), which indicates that differentiation-inducing pathway Notch1 is downregulated/inhibited in α6^+^/MHCI^- ^cells.

**Figure 6:**
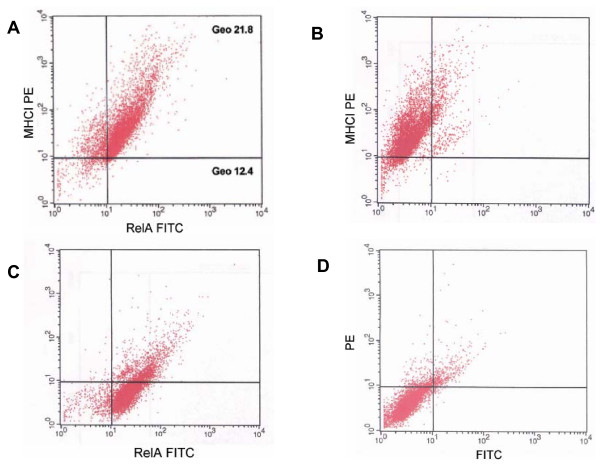
**NF-κB activity in MHCI negative and MHCI positive cells**. (A) A representative flow cytometry analysis of the expression of MHCI and NF-κB subunit RelA/p65 proteins showing that epidermal cells that exhibit low expression of RelA/p65 also exhibit lack/low expression of MHCI. The geometrical mean channel fluorescence of the populations is indicated. (B) Single positive control for PE. (C) Single positive control for FITC. (D) Secondary control.

It has been shown that Notch1 signaling pathway activates NF-κB pathway and upregulates subunits of NF-κB and its targets [56-58]. Thus, we investigated whether the levels of activity of NF-κB pathway, which is downstream of Notch1 pathway, differ in MHCI^- ^and MHCI^+ ^cells. First, we analyzed the expression of NF-κB subunit RelA in MHCI^- ^and MHCI^+ ^cells and found a positive correlation between the expression of NF-κB subunit RelA and MHCI (Figure 6). Since both MHCI molecules and NF-κB subunits are targets of NF-κB pathway, this result suggested that similar to the Notch1 pathway, NF-κB pathway is downregulated in MHCI^- ^cells.

**Figure 7:**
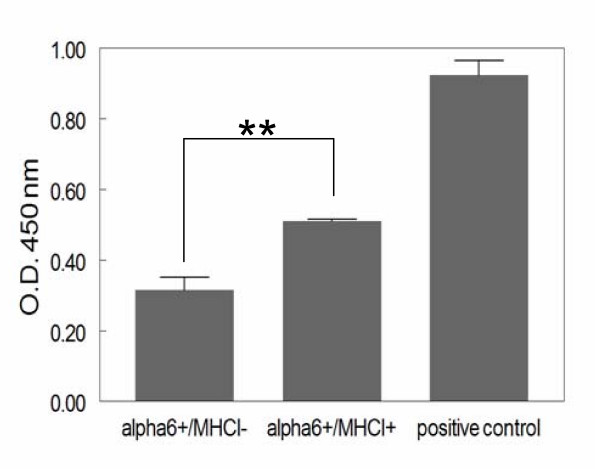
**NF-κB activity in α6^+^/MHCI negative and MHCI positive cells**. Nuclear extracts of sorted cells were analyzed for NF-κB p50-binding activity; data were expressed in optical density (O.D.) units obtained with the TransAM ELISA NF-κB assay for phosopho-p50 (**P < 0.017).

Search of our cDNA array data base for activators and targets of NF-κB pathway identified transcripts of genes, which either directly activate NF-κB pathway, or play a role in the activation of this pathway, such as *BMP2 *and *MALT1 *[59], that were more abundant in α6^+^/MHCI^+ ^cells compared to α6^+^/MHCI^- ^cells. Furthermore, in accordance with this finding, transcripts of thirty genes that are reported to be upregulated by Rel/NF-κB transcription factors [59], were enriched in α6^+^/MHCI^+ ^cells (Table 2). Thus, our microarray data suggest that the NF-κB pathway is downregulated in α6^+^/MHCI^- ^cells as compared to α6^+^/MHCI^+ ^cells. To determine whether NF-κB pathway is indeed more active in α6^+^/MHCI^+ ^cells, we performed TRANS AM NF-κB ELISA assay using nuclear extracts of α6^+^/MHCI^+ ^and α6^+^/MHCI^- ^cells. We found that the relative amount of nuclear phospho-NF-κB p50 bound to DNA, an indicator of an active NF-κB pathway, is higher in α6^+^/MHCI^+ ^cells than α6^+^/MHCI^- ^cells (Figure 7). Collectively, the data demonstrate that Notch1 signaling as well as the downstream NF-κB pathway are both downregulated in α6^+^/MHCI^- ^cells compared to α6^+^/MHCI^+ ^cells.

### The comparison of our data with the transcriptional profile of the genes that are differentially expressed in the basal and differentiating layers of the epidermis

**Figure 8:**
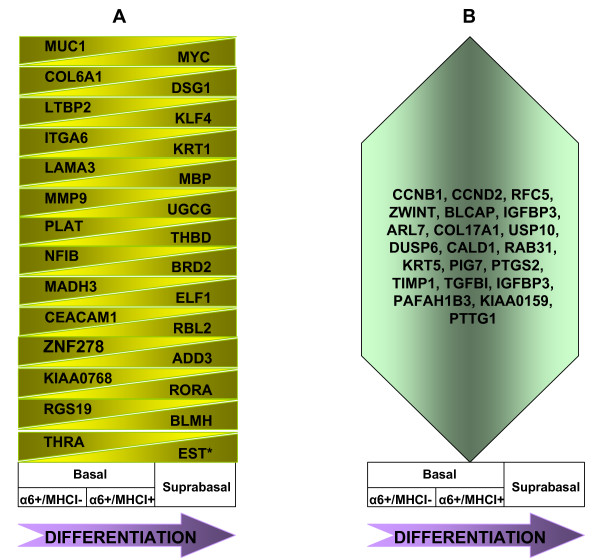
**Changes in the gene expression during epidermal differentiation**. (A) Genes that are gradually downregulated, or upregulated during epidermal differentiation. The EST* represents the Human clone 23933 mRNA (B) Genes that are upregulated in α6^+^/MHCI^+ ^cells (TA cells), and subsequently downregulated in suprabasal cells.

To gain further insights of epidermal differentiation, we compared our database with the published database of differentially expressed genes in basal and suprabasal layers of the human epidermis [60]. The comparison of our data with the transcriptional profile of the genes that are differentially expressed in the basal and differentiated layers of the epidermis suggests that TGF-β/phospho-Smad pathway-induced transcription profile fades along the epidermal differentiation axis. The abundance of transcripts, which are upregulated by TGF-β/phospho-Smad pathway (such as *COL6A1*, *LTBP2*, *MMP9*, *PLAT*), decrease during differentiation, from presumptive stem to transient amplifying cells (basal layer) and further to cells of the suprabasal layers, where these transcripts are present at the lowest level. In addition, the transcript of *MADH3*, a transcriptional activator of TGF-β responses, is also increasingly downregulated. Conversely, abundance of transcripts, which are downregulated by TGF-β/phospho-Smad pathway (such as *KLF4 *and *MYC*), appear to positively correlate with the increase of epidermal differentiation (Figure 8A).

On the other hand, the comparison suggests that Notch1 and Wnt pathway-induced transcription profiles strengthen along the epidermal differentiation axis. Transcripts of *MYC *and *ELF1*, which are upregulated by Wnt signaling gradually increase. These findings are in accordance with the previous reports, which demonstrate that TGF-β/phospho-Smad pathway prevents keratinocyte differentiation [48], and, conversely, Notch1 and Wnt pathways induce keratinocyte differentiation [54,61,62]. Thus, it might be possible that while TGF-β/phospho-Smad pathway is gradually downregulated, Wnt pathway becomes increasingly more active during epidermal differentiation. Nevertheless, further investigation is necessary to validate this hypothesis. Similarly, while the transcription of *KRT1*, which is upregulated by Notch1 signaling, increases, *ITGA6 *mRNA, which is downregulated by Notch1 signaling, decreases in keratinocytes during differentiation (Figure 8A). Several reports demonstrated that suprabasal cells have higher Notch1 activity than basal cells [55,63]. As already mentioned, employing flow cytometry analysis and antibodies against cleaved/active Notch 1, no detectable levels of cleaved/active Notch 1 was observed in MHCI^- ^cells indicating the lack of Notch1 activity (Figure 5).

Interestingly, previous reports suggested that strong adherence of stem cells to extracellular matrix-rich basement membrane may be involved in retaining these cells in their natural residence (niche) [52,64]. In accordance with these observations, the comparison of our data with published transcription profiles of the basal and suprabasal cells of the epidermis revealed that during differentiation transcripts of several genes (*MUC1*, *COL6A1*, *ITGA6*, *MMP9*, *PLAT*, *CEACAM1*) whose products are soluble or membrane-bound factors that play a role in the interaction of cells with the microenvironment, decrease gradually during differentiation (Figure 8A).

We also found that twenty-one genes were upregulated in α6^+^/MHCI^+ ^cells (TA cells) alone, and later become downregulated during terminal differentiation (Figure 8B). Among these transcripts there are ones related to cell cycle (*CCNB1*, *CCND2*, *RFC5*) as well as mRNAs of the genes whose products induce cell growth and division (*ZWINT*, *BLCAP*). Since α6^+^/MHCI^- ^cells are quiescent, and terminally differentiating cells are post-mitotic it is not surprising to find transcripts whose products accelerates cell proliferation among the genes that are upregulated only in α6^+^/MHCI^+ ^cells during epidermal differentiation. Similarly, genes whose products suppress cell growth and proliferation were enriched both in α6^+^/MHCI^- ^cells and terminally differentiating suprabasal cells, such as *PLAGL1*, a zinc finger transcription factor that induces cell cycle arrest in the skin and whose expression is diminished in basal cell carcinomas [65], putative tumor suppressors insulin-like growth factor-binding protein 7 (*IGFBP7*), and *DOC1 *[66,67] (see Additional file 1 and reference [60]).

## Conclusion

Most of our knowledge regarding epidermal stem cells comes from murine studies. This is the first report that uncovers the transcriptional profile of human interfollicular epidermal stem cells and their progeny, transient amplifying cells isolated directly from their niches and analyzed.

In summary, the results presented here show that α6^+^/MHCI^- ^cells exhibit characteristics attributed to stem cells. Comparison of the transcription profiles of α6^+^/MHCI^- ^cells and α6^+^/MHCI^+^cells with the existing profiles of hair follicle bulge stem cells further indicate that α6^+^/MHCI^- ^cells are enriched for stem cells. Our findings may bring new insights into regulatory mechanisms involved in epidermal homeostasis, and bring understanding of deregulations of these mechanisms that take place in skin disorders including cancer, and most importantly may lead to identification of potential therapeutic targets. In addition, as a first comprehensive gene expression profile of putative human epithelial cells isolated directly from tissue, the generated database may be of importance for studies of gene expression profiles of other human epithelial tissues. By defining characteristics of interfollicular epidermal stem cells and by identifying genes whose expression is altered during differentiation, we have opened new roads for better understanding of stem cell characteristics and epidermal differentiation.

## Methods

### Isolation of keratinocytes

Neonatal foreskins were obtained from routine circumcisions. After washing in PBS, and removing of subcutaneous fat, the tissue was cut into 5 × 5 mm pieces and incubated overnight at 4°C in Dulbecco's modified Eagle's medium containing 2.5 mg/ml Dispase II (Boehringer Mannheim, Indianopolis, IN), penicillin (100 units per ml), and streptomycin (100 μg/ml). Epithelial sheaths were separated from the dermis by gentle peeling with forceps. Keratinocytes were harvested after incubation with trypsin/EDTA solution (0.05% and 0.01%, respectively) for 10 minutes at 37°C [22].

### Immunocytochemistry and flow cytometry

After trypsin neutralization and blocking with buffer containing BSA and human IgG, keratinocytes were immunolabeled with R-phycoerythrin (PE)-conjugated mouse anti-human β2 microglobulin (BD Biosciences, San Diego, CA, USA), and with a monoclonal anti-human α6 integrin-fluorescein isothiocyanate (FITC) conjugate (Serotec, Oxford, UK). Control samples were incubated with appropriate isotype controls. All incubations were performed at 4°C. Cells were sorted using FACSVantage, or FACSAria (Becton Dickinson, Franklin Lakes, NJ). Flow cytometry data used for sorting cells that donated RNA for microarray experiments, as well as representative controls are shown in Additional file 7. The data were analyzed using Cell Quest software (BD Biosciences). Selection of basal keratinocytes with anti-human α6 integrin eliminates suprabasal cells (terminally differentiated keratinocytes), and non-keratinocytes, such as melanocytes and dentritic cells) [21,60].

For the expression of Ki67 isolated keratinocytes were immunolabeled with mouse anti-human antibody against MHCI (BD Biosciences) and with a polyclonal antibody against Ki67 (Zymed San Francisco, CA). Secondary antibodies were goat anti mouse IgG1 PE-conjugated antibody (Southern Biotechnology Associates Inc. Birmingham, AL) and donkey anti rabbit FITC-conjugated antibody. For the setting of gates, secondary control and single color positive controls were used.

### Keratinocyte culture

Keratinocytes were grown in a keratinocyte medium (3:1 DMEM, F12) supplemented with FBS and additives in 100 mm culture dishes, previously seeded with lethally irradiated 3T3 fibroblasts [68]. The medium was replaced every other day. Colonies were visualized after two weeks in culture following fixation in 10% formalin and staining with 2% rhodamine B (Sigma). Colony forming efficiency (CFE) is the ratio of colony number to plating cell number expressed as a percentage. Results are presented as means ± SD.

### RNA isolation, amplification and microarray analysis

Following cell sorting, RNA isolation (RNeasy Mini Kit (Qiagen)) and amplification (MessageAmp aRNA Kit (Ambion)) microarray was performed using Affymetrix HGU 133 A+B GeneChip set. Scanned images of Affymetrix GeneChip arrays were quantified using Affymetrix GCOS software, Gene Chip Operating System. The target intensity was set to 500 and the default parameters were used. The results were filtered and probe sets with a "No Change" (NC) call were removed. Additionally, probe sets that were scored "Increased" (I) or "Marginal Increase" (MI), but called absent on the experimental sample, as well as probe sets that were scored Decreased (D) or "Marginal Decrease" (MD) and called absent in the baseline sample, were removed. For the resulting list of probe sets a fold change column was calculated. Microarray Gene Expression Data have been deposited, accession number GSE11089 [69]. For the genes that are differentially expressed in both arrays see Additional files 2, 8 and 9.

### Semi-quantative RT-PCR

Total RNA was isolated directly after cell sorting using the RNeasy Mini Kit (QIAGEN) according to manufacturer's protocol. Extracted RNA was reverse transcribed by using Sensiscript RT Kit (QIAGEN) according to manufacturer's instructions. It must be noted that RNA used for RT-PCR and for each microarray analysis was isolated from cells derived from multiple skin samples that consisted of different donors. By pooling skin samples we were able to obtain sufficient amount of cells and at the same time average any potential individual differences. Primers for *KRTHA1 *[70], β-catenin [71], *KLF4 *[72], and *DSG3 *[73] were published previously. Other primers used are: ITGA6 F: 5'-TGCTGTTGGTTCCCTCTCAGAT-3'. ITGA6 R: 5'-CTGGCGGAGGTCAATTCTGT-3'. MYCBP F: 5'-ATGGCCCATTACAAAGCCGC-3'. MYCBP R: 5'-CTATTCAGCACGCTTCTCCT-3'. Initial PCR step was 1 minute at 94.0°C, followed by 25, 30, 35 cycles of a 15 seconds melting at 94.0°C, a 15 seconds annealing at 55.0°C and a 15 seconds extension at 72.0°C. The final extension was at 72.0°C for 1 minute.

### Validation of microarray data using immynocytochemistry and flow cytometry

For the analyses of protein expressions in MHCI^- ^and MHCI^+ ^cells the following antibodies were used: Antibody against MHCI, mouse anti-human (IgG1 isotype, BD biosciences); antibody against NF-κB p65, rabbit anti human (Santa Cruz); antibody against cleaved/active Notch1, rabbit anti human (Calbiochem); and antibody against CD71, mouse anti human (IgG1 isotype, Diaclone, France). Secondary antibodies used for these analyses were: FITC-conjugated goat anti-mouse IgM (Sigma), PE-conjugated goat anti mouse IgG1 (SouthernBiotechnology, Birmingham, Al), and FITC-conjugated donkey anti-rabbit (Jackson ImmunoResearch Lab. Inc., West Grove, PA).

### Analysis of NF-κB activity using TransAM NF-κB p50 kit

Nuclear extracts were obtained using Nuclear Extract Kit purchased from Active Motif (Carlsbad, CA). 100,000 cells (α6^+^/MHCI^+ ^and α6^+^/MHCI^- ^each) were directly sorted in PBS buffer that contained phosphatase inhibitors, supplied with the kit. Levels of the active/phospho-NF-κB p50 in the nucleus were assayed using TransAM NF-κB p50 Transcription Factor Assay Kit (Active Motif) according to manufacturer's protocol. Nuclear extract of HeLa cells stimulated with TNF-α for 30 minutes supplied by Active Motif was used as a positive control.

### Microarray data analysis

Additional file 1 contains all the transcripts, which demonstrated equal or higher than 2 (≥ 2) fold difference between α6^+^/MHCI^- ^cells and α6^+^/MHCI^+ ^cells (Additional file 1 shows only values that are ≥ 2 fold in log2 scale). The genes that are consistently downregulated or upregulated in both arrays were shown in tables S6, S7 and S8. All transcripts that belong to MHCI protein family are downregulated in α6^+^/MHCI^- ^cells as expected, which indicate that our selection process was successful (see Additional file 1). Several reports were used as the base to screen for c-Myc target genes [74-81], Wnt target genes [82-93], TGF-β/BMP target genes [94-97], and targets of NF-κB pathway [59,98-100].

### Statistical analysis

Student's *t*-test was applied for statistical analysis. Error bars represent ± SD.

## Abbreviations

α6: integrin alpha 6; BMP: Bone Morphogenic Protein; CDKN: cyclin dependent kinase inhibitor; CFE: colony forming efficiency; IFN-α: interferon alpha; KRT: keratin; MHCI: Major Histocompatibility Complex I; NF-κB: Nuclear Factor kappa B; α6^+^/MHCI^-^; α6^+^/MHCI^+ ^denote keratinocytes sorted according to integrin alpha 6 and MHCI expressions. MHCI^- ^and MHCI^+ ^denote total epidermal cells that express or do not express MHCI.

## Competing interests

The authors declare that they have no competing interests.

## Authors' contributions

MM designed and organized the study, performed cell isolation and labeling for flow cytometry analysis and FACS sorting, isolated RNA for microarray analysis, participated in data analysis, manuscript drafting and revision. SSK analyzed the microarray data, performed RT-PCR reactions and NF-κB determination in isolated nuclei, prepared figures and manuscript draft and was involved in organization of the study and manuscript revision. SRS participated in the design and organization of the study, and manuscript revission. PMD participated in the analysis of the microarray data and manuscript revision. MFB participated in the analysis of the microarray data.

## Supplementary Material

Additional file 1 List of the genes that are differentially expressed in α6+/MHCI- cells and in α6+/MHCI+ cells. Entire Affymetrix probe set and their annotated genes that are up-regulated ≥ 2-fold in either α6+/MHCI- cells or α6+/MHCI+ cells sorted according to their functions. Some of the genes are involved in multiple processes in the cell and could be placed in several tables. The table shows the difference in the expression α6+/MHCI+ cells vs. α6+/MHCI- cells. "-"sign indicates that the gene is upregulated in α6+/MHCI- cells. The numbers that show the difference in the level of gene expression are in log2 scale. Click here for file

Additional file 2 List of the entire genes that are differentially expressed in either α6+/MHCI+ cells or α6+/MHCI- cells and are consistently upregulated or down regulated in both arrays. "-"sign indicates that the gene is upregulated in α6+/MHCI- cells. The numbers that show the difference in the level of gene expression are in log2 scale. Click here for file

Additional file 3 List of the genes that are differentially expressed in α6^+^/MHCI^+^cells vs. α6^+^/MHCI^- ^cells and also expressed in human hair follicle SCs (HHFSC). "-"sign indicates that the gene is upregulated in α6^+^/MHCI^- ^cells. The numbers that show the difference in the level of gene expression are in log2 scale. Click here for file

Additional file 4 List of the genes that are differentially expressed in α6^+^/MHCI^+^cells vs. α6^+^/MHCI^- ^cells and also expressed in murine hair follicle SCs (MHFSC). "-"sign indicates that the gene is upregulated in α6^+^/MHCI^- ^cells. The numbers that show the difference in the level of gene expression are in log2 scale. Click here for file

Additional file 5 Expression of TCF3 targets in α6^+^/MHCI^- ^and α6^+^/MHCI^+^cells. The table shows the difference in the expression in α6^+^/MHCI^+ ^cells vs. α6^+^/MHCI^- ^cells. "-"sign indicates that the gene is upregulated in α6^+^/MHCI^- ^cells. The numbers that show the difference in the level of gene expression are in log2 scale. Click here for file

Additional file 6 Expression of MYC targets in α6^+^/MHCI^- ^and α6^+^/MHCI^+^cells. The table shows the difference in the expression in α6^+^/MHCI^+ ^cells vs. α6^+^/MHCI^- ^cells. "-"sign indicates that the gene is upregulated in α6^+^/MHCI^- ^cells. The numbers that show the difference in the level of gene expression are in log2 scale. Click here for file

Additional file 7 Flow cytometry data of sorted cells that donated RNA for microarray experiments. Click here for file

Additional file 8 List of the genes that are differentially expressed in α6+/MHCI+ cells and α6+/MHCIcells and are consistently upregulated or downregulated ≥ 2 fold in both arrays. "-"sign indicates that the gene is upregulated in α6+/MHCI- cells. The numbers that show the difference in the level of gene expression are in log2 scale. Click here for file

Additional file 9 List of the genes that are differentially expressed in at least one array ≥ 2 fold in either α6+/MHCI+ cells or α6+/MHCI- cells and are consistently upregulated or down regulated in both arrays. "-"sign indicates that the gene is upregulated in α6+/MHCI- cells. The numbers that show the difference in the level of gene expression are in log2 scale. Click here for file
