# Supplementary material for: Transcriptional profiling of putative human epithelial stem cells
Source: BMC Genomics. 2008 Jul 30;9:359. doi: 10.1186/1471-2164-9-359 (PMC2536675; doi:10.1186/1471-2164-9-359)
Supplement: Additional file 1 — List of the genes that are differentially expressed in α6+/MHCI- cells and in α6+/MHCI+ cells. Entire Affymetrix probe set and their annotated genes that are up-regulated ≥ 2-fold in either α6+/MHCI- cells or α6+/MHCI+ cells sorted according to their functions. Some of the genes are involved in multiple processes in the cell and could be placed in several tables. The table shows the difference in the expression α6+/MHCI+ cells vs. α6+/MHCI- cells. "-"sign indicates that the gene is upregulated in α6+/MHCI- cells. The numbers that show the difference in the level of gene expression are in log2 scale. [file 1471-2164-9-359-S1.pdf]

Probe Set	Exp. 1 Signal Log Ratio	Exp. 2 Signal Log Ratio	Definition / Product	symbol
			**WNT signaling**	
203697_at	-3.9	-3.2	Human Fritz mRNA	FRZB
203698_s_at	-2.8	-2.7	frizzled-related protein	FRZB
204451_at		-1.3	frizzled (Drosophila) homolog 1	FZD1
218665_at		-1.7	frizzled (Drosophila) homolog 4	FZD4
203706_s_at	-1.1	-1.5	frizzled (Drosophila) homolog 7	FZD7
204712_at	-6.6	-3.8	Wnt inhibitory factor-1	WIF-1
221455_s_at		3.8	wingless-type MMTV integration site family, member 3	WNT3
208606_s_at		3.6	wingless-type MMTV integration site family, member 4	WNT4
204602_at	-1.3	-1.9	dickkopf (Xenopus laevis) homolog 1	DKK1
219908_at	-1.7		dickkopf (Xenopus laevis) homolog 2	DKK2
202196_s_at		2.5	dickkopf (Xenopus laevis) homolog 3	DKK3
213730_x_at		-1	transcription factor 3 (E2A immunoglobulin enhancer binding factors E12E47)	TCF3
209153_s_at		-1.3	transcription factor 3 (E2A immunoglobulin enhancer binding factors E12E47)	TCF3
221016_s_at	-1.5		HMG-box transcription factor TCF-3	TCF-3
205255_x_at	-1.1		transcription factor 7 (T-cell specific, HMG-box)	TCF7
212761_at		-1.2	transcription factor 7-like 2 (T-cell specific, HMG-box)	TCF7L2
203221_at	-1		transducin-like enhancer of split 1, homolog of Drosophila E(sp1)	TLE1
210691_s_at	1.1		Homo sapiens PNAS-107 mRNA, complete cds.	CACYBP
212596_s_at		1.3	high-mobility group protein 2-like 1	HMG2L1
201278_at	-1.9		disabled (Drosophila) homolog 2 (mitogen-responsive phosphoprotein)	DAB2
210835_s_at		-1	ribeye	CTBP2
			**WNT signaling targets**	
			INDUCED TARGETS AMONG mRNAs ENRICHED IN ALPHA6+/MHCI+ CELLS	
202431_s_at		1	v-myc avian myelocytomatosis viral oncogene homolog	MYC
208711_s_at		1.3	cyclin D1 (PRAD1: parathyroid adenomatosis 1)	CCND1
201465_s_at		1	v-jun avian sarcoma virus 17 oncogene homolog	JUN
200951_s_at		3.3	cyclin D2	CCND2
204170_s_at	1.1		CDC28 protein kinase 2	CKS2
214710_s_at	2.2	1.8	cyclin B1	CCNB1
212022_s_at		2	antigen identified by monoclonal antibody Ki-67	MKI67
202095_s_at		2.5	baculoviral IAP repeat-containing 5 (survivin)	BIRC5
213201_s_at		3.5	troponin T1, skeletal, slow	TNNT1
206393_at	1.4		troponin I, skeletal, fast	TNNI2
201152_s_at		1.4	muscleblind (Drosophila)-like	MBNL
212143_s_at	1.1		insulin-like growth factor binding protein 3	IGFBP3
203554_x_at	1.1	1	pituitary tumor-transforming 1	PTTG1
201983_s_at		1.2	epidermal growth factor receptor (avian erythroblastic leukemia viral (v-erb-b) oncogene homolog)	EGFR
201325_s_at		2.1	epithelial membrane protein 1	EMP1
209257_s_at		2.5	chondroitin sulfate proteoglycan 6 (bamacan)	CSPG6
209258_s_at		2.8	chondroitin sulfate proteoglycan 6 (bamacan)	CSPG6
201615_x_at		1.2	caldesmon 1	CALD1
209212_s_at		1.8	mRNA for transcription factor BTEB2, complete cds	BTEB2
208893_s_at		3.5	clone MGC:12852, mRNA	DUSP6
209189_at		2.2	v-fos FBJ murine osteosarcoma viral oncogene homolog	FOS
200760_s_at		1.2	vitamin A responsive; cytoskeleton related	JWA
211015_s_at		1.4	heat shock protein 70 (hsp70) mRNA	hsp70
202554_s_at		1.3	glutathione S-transferase M3 (brain)	GSTM3
201820_at	1		keratin 5 (epidermolysis bullosa simplex, Dowling-MearaKobnerWeber-Cockayne types)	KRT5
209060_x_at		1.3	nuclear receptor coactivator 3	NCOA3
209061_at		1	nuclear receptor coactivator 3	NCOA3
210809_s_at		1.1	osteoblast specific factor 2 [osf-2]	
202071_at	1		syndecan 4 (amphiglycan, ryudocan)	SDC4
212420_at		1.6	E74-like factor 1 (ets domain transcription factor)	ELF1
200943_at		1	high-mobility group (nonhistone chromosomal) protein 14	HMG14
200598_s_at		2.3	tumor rejection antigen (gp96) 1	TRA1
209024_s_at	1.2	1	NS1-associated protein 1	NSAP1
201014_s_at	1.4	2	multifunctional polypeptide similar to SAICARsynthetase and AIR carboxylase	ADE2H1
202532_s_at		1.1	dihydrofolate reductase	DHFR
203968_s_at	1.6		CDC6 (cell division cycle 6, S. cerevisiae) homolog	CDC6
222037_at	1.2	3.4	minichromosome maintenance deficient (S. cerevisiae) 4	MCM4
212141_at	1		minichromosome maintenance deficient (S. cerevisiae) 4	MCM4
211953_s_at		1.2	karyopherin (importin) beta 3	KPNB3
			REPRESED TARGETS AMONG mRNAs ENRICHED IN ALPHA6+/MHCI- CELLS	
206891_at		-1.1	skeletal muscle specific actinin, alpha 3	ACTN3
210517_s_at	-1.1	-1	Homo sapiens mRNA for gravin, complete cds.	AKAP12
213275_x_at	-1.4	-1	cathepsin B	CTSB
213274_s_at	-1.1		cathepsin B	CTSB
206214_at	-3.1		phospholipase A2, group VII (platelet-activating factor acetylhydrolase, plasma)	PLA2G7
204682_at		-1	latent transforming growth factor beta binding protein 2	LTBP2
201278_at	-1.9		disabled (Drosophila) homolog 2 (mitogen-responsive phosphoprotein)	DAB2
207345_at	-2	-2.3	follistatin, transcript variant FST317	FST
204948_s_at	-1.1	-1.9	follistatin, transcript variant FST344	FST
222043_at		-1.2	clusterin (complement lysis inhibitor, SP-40,40, sulfated glycoprotein 2, testosterone-repressed prostate message 2, apolipoprotein J)	CLU

213730_x_at		-1	transcription factor 3 (E2A immunoglobulin enhancer binding factors E12E47)	TCF3
209153_s_at		-1.3	transcription factor 3 (E2A immunoglobulin enhancer binding factors E12E47)	TCF3
221016_s_at	-1.5		HMG-box transcription factor TCF-3	TCF-3
212732_at		-1.7	maternally expressed 3	MEG3
212226_s_at		-1.2	phosphatidic acid phosphatase type 2B	PPAP2B
212230_at	-1.6	-1.3	phosphatidic acid phosphatase type 2B	PPAP2B
203549_s_at	-1.3		lipoprotein lipase	LPL
203706_s_at	-1.1	-1.5	frizzled (Drosophila) homolog 7	FZD7
209291_at		-1	inhibitor of DNA binding 4, dominant negative helix-loop-helix protein	ID4
213348_at	-1.1		cyclin-dependent kinase inhibitor 1C (p57, Kip2)	CDKN1C
213183_s_at	-1.5	-2.1	cyclin-dependent kinase inhibitor 1C (p57, Kip2)	CDKN1C
			**TGF/BMP Signalling**	
205396_at	-1.3		MAD (mothers against decapentaplegic, Drosophila) homolog 3	MADH3
207069_s_at		-1.6	MAD (mothers against decapentaplegic, Drosophila) homolog 6	MADH6
208446_s_at	-1.5		MAD (mothers against decapentaplegic, Drosophila) homolog interacting protein, receptor	MADHIP
			activation anchor, transcript variant 2	
208310_s_at	1.1		follistatin-like 1	FSTL1
207345_at	-2	-2.3	follistatin, transcript variant FST317	FST
204948_s_at	-1.1	-1.9	follistatin, transcript variant FST344	FST
205289_at		1.3	bone morphogenetic protein 2	BMP2
205430_at		-5.1	bone morphogenetic protein 5	BMP5
205431_s_at	-2.4		bone morphogenetic protein 5	BMP5
207865_s_at		-2	bone morphogenetic protein 8 (osteogenic protein 2)	BMP8
208292_at	-1.2		bone morphogenetic protein 10	BMP10
221332_at	-1.3		bone morphogenetic protein 15	BMP15
202526_at	-1.5		homozygous deletion target in pancreatic carcinoma	DPC4
205187_at		1.9	SMAD5	
205209_at		1.1	activin A receptor, type IB	ACVR1B
207687_at	-1.4		inhibin, beta C	INHBC
207688_s_at		-1.8	inhibin, beta C	INHBC
208944_at		1.3	TGF-betaIIR alpha	TGFBR2
216917_s_at	-3.8		H.sapiens mRNA for SCP1 protein.	SCP1
			**TGF/BMP-Induced Factors**	
			INDUCED TARGETS AMONG mRNAs ENRICHED IN ALPHA6+/MHCI- CELLS	
203697_at	-3.9	-3.2	Human Fritz mRNA	FRZB
203698_s_at	-2.8	-2.7	frizzled-related protein	FRZB
211980_at	-1.9	-1.2	collagen, type IV, alpha 1	COL4A1
212070_at	-1.2		G protein-coupled receptor 56	GPR56
201416_at		-1.2	SRY (sex determining region Y)-box 4	SOX4
222043_at		-1.2	clusterin (complement lysis inhibitor, SP-40,40, sulfated glycoprotein 2, testosterone-	CLU
			repressed prostate message 2, apolipoprotein J)	
213446_s_at	-1.3		IQ motif containing GTPase activating protein 1	IQGAP1
218574_s_at	-1.3	-2.7	LIM and cysteine-rich domains 1	LMCD1
221489_s_at	-1.2		sprouty (Drosophila) homolog 4	SPRY4
201124_at	-1.1		integrin, beta 5	ITGB5
201125_s_at		-1	integrin, beta 5	ITGB5
202729_s_at		-1.1	latent transforming growth factor beta binding protein 1	LTBP1
204682_at		-1	latent transforming growth factor beta binding protein 2	LTBP2
200696_s_at	-1		gelsolin (amyloidosis, Finnish type)	GSN
212226_s_at		-1.2	phosphatidic acid phosphatase type 2B	PPAP2B
212230_at	-1.6	-1.3	phosphatidic acid phosphatase type 2B	PPAP2B
200787_s_at	-1.8		phosphoprotein enriched in astrocytes 15	PEA15
202149_at		-1.2	enhancer of filamentation 1 (cas-like docking; Crk-associated substrate related)	HEF1
213560_at		-1.3	growth arrest and DNA-damage-inducible, beta	GADD45B
209305_s_at		1.4	growth arrest and DNA-damage-inducible proteinGADD45beta	GADD45B
213931_at	-1		inhibitor of DNA binding 2, dominant negative helix-loop-helix protein	ID2
209651_at		-1.3	Similar to transforming growth factor beta 1induced transcript 1	TGFB1I1
203868_s_at	-1.2		vascular cell adhesion molecule 1	VCAM1
207345_at	-2	-2.3	follistatin, transcript variant FST317	FST
204948_s_at	-1.1	-1.9	follistatin, transcript variant FST344	FST
201645_at	-1.3		hexabrachion (tenascin C, cytotactin)	HXB
203325_s_at		-1.1	collagen, type V, alpha 1	COL5A1
221731_x_at	-1.5		chondroitin sulfate proteoglycan 2 (versican)	CSPG2
204620_s_at	-2	-1.4	chondroitin sulfate proteoglycan 2 (versican)	CSPG2
217161_x_at		-1.5	cartilage specific proteoglycan (600 AA)	AGC1
209291_at		-1	inhibitor of DNA binding 4, dominant negative helix-loop-helix protein	ID4
221963_x_at	-1.1		thrombospondin 1	THBS1
211161_s_at	-1		PRO3121	COL3A1
201110_s_at	-2.3	-1.9	thrombospondin 1	THBS1
213428_s_at		-1.1	collagen, type VI, alpha 1	COL6A1
213622_at	-1.5	-1.6	collagen, type IX, alpha 2	COL9A2
37892_at		-3.7	alpha-1 type XI collagen	COL11A1
216993_s_at	-1		collagen, type XI, alpha 2	COL11A2
212865_s_at	-1.3	-1.1	collagen, type XIV, alpha 1 (undulin)	COL14A1
212884_x_at	-1.1		apolipoprotein E	APOE
207069_s_at		-1.6	MAD (mothers against decapentaplegic, Drosophila) homolog 6	MADH6
210162_s_at	-1.1	-1.7	NF-ATc mRNA	NFATC1
200884_at	-1.5		creatine kinase, brain	CKB
203936_s_at	-1.5		matrix metalloproteinase 9 (gelatinase B, 92kD gelatinase, 92kD type IV collagenase)	MMP9
214866_at		-1.3	mRNA for urokinase plasminogen activator receptor	PLAUR
201860_s_at	-1.4		plasminogen activator, tissue	PLAT
200602_at		-1.2	Homo sapiens amyloid beta (A4) precursor protein (protease nexin-II, Alzheimer disease)	APP
212588_at	-1.4		mRNA for T200 leukocyte common antigen (CD45, LC-A).	PTPRC

204451_at		-1.3	frizzled (Drosophila) homolog 1	FZD1
210105_s_at	-1.6	-1.8	FYN oncogene related to SRC, FGR, YES	FYN
206219_s_at	-1.3		vav 1 oncogene	VAV1
202957_at	-1.1		hematopoietic cell-specific Lyn substrate 1	HCLS1
206283_s_at	-1.1		T-cell acute lymphocytic leukemia 1	TAL1
203549_s_at	-1.3		lipoprotein lipase	LPL
206382_s_at		-2.6	Homo sapiens brain-derived neurotrophic factor	BDNF
215148_s_at	-1.3		amyloid beta (A4) precursor protein-binding, family A, member 3 (X11-like 2)	APBA3
			REPRESSED TARGETS AMONG mRNAs ENRICHED IN ALPHA6+/MHCI+ CELLS	
202431_s_at		1	v-myc avian myelocytomatosis viral oncogene homolog	MYC
217165_x_at		1.6	metallothionein 1F (functional)	MT1F
204745_x_at		1.1	metallothionein 1G	MT1G
205480_s_at		1.6	UDP-glucose pyrophosphorylase 2	UGP2
200951_s_at		3.3	cyclin D2	CCND2
204734_at		1.2	keratin 15	KRT15
217234_s_at		3.3	cytovillin 2	VIL2
202712_s_at	1		creatine kinase, mitochondrial 1 (ubiquitous), nuclear gene encoding mitochondrial protein, mRNA	CKMT1
219850_s_at		2.2	Homo sapiens Ets homologous factor (EHF), mRNA.	EHF
217966_s_at		1.7	niban protein	NIBAN
202546_at	1	3.2	vesicle-associated membrane protein 8 (endobrevin)	VAMP8
206393_at	1.4		troponin I, skeletal, fast	TNNI2
			**p53-Related**	
211193_at	-1.1		Homo sapiens p53 homolog (p40) mRNA, complete cds.	
			INDUCED TARGETS AMONG mRNAs ENRICHED IN ALPHA+/MHCI+ CELLS	
203725_at		1.2	growth arrest and DNA-damage-inducible, alpha	GADD45A
202672_s_at		3.3	activating transcription factor 3	ATF3
205891_at		1.5	adenosine A2b receptor	ADORA2B
212143_s_at	1.1		insulin-like growth factor binding protein 3	IGFBP3
			REPRESED TARGETS AMONG mRNAs ENRICHED IN ALPHA+/MHCI- CELLS	
202729_s_at		-1.1	latent transforming growth factor beta binding protein 1	LTBP1
			**NFkB Activators**	
205289_at		1.3	bone morphogenetic protein 2	BMP2
208309_s_at	1	1.4	mucosa associated lymphoid tissue lymphomatranslocation gene 1	MALT1
			**NFkB Targets**	
			INDUCED TARGETS AMONG mRNAs ENRICHED IN ALPHA6+/MHCI+ CELLS	
200748_s_at		1.7	ferritin, heavy polypeptide 1	FTH1
210095_s_at		2	Human growth hormone-dependent insulin-like growth factor-binding protein mRNA	IGFBP1
200943_at		1	high-mobility group (nonhistone chromosomal) protein 14	HMG14
209699_x_at	1		dihydrodiol dehydrogenase	AKR1C2
204881_s_at		1.1	UDP-glucose ceramide glucosyltransferase	UGCG
202269_x_at		1.2	guanylate binding protein 1, interferon-inducible, 67kD	GBP-1
208711_s_at		1.3	cyclin D1 (PRAD1: parathyroid adenomatosis 1)	CCND1
202672_s_at		3.3	activating transcription factor 3	ATF3
202071_at	1		syndecan 4 (amphiglycan, ryudocan)	SDC4
204748_at		1.3	prostaglandin-endoperoxide synthase 2 (prostaglandin GH synthase and cyclooxygenase)	PTGS2
205289_at		1.3	bone morphogenetic protein 2	BMP2
208893_s_at		3.5	clone MGC:12852, mRNA	DUSP6
211985_s_at	1.1		matrix Gla protein	MGP
209189_at		2.2	v-fos FBJ murine osteosarcoma viral oncogene homolog	FOS
216598_s_at		5.4	monocyte chemotactic protein (human, aortic endothelial cells, mRNA, 661 nt).	MCP-1
200706_s_at		2	LPS-induced TNF-alpha factor	PIG7
217871_s_at		1.8	macrophage migration inhibitory factor (glycosylation-inhibiting factor)	MIF
204285_s_at		1.3	phorbol-12-myristate-13-acetate-induced protein 1	PMAIP1
220494_s_at	1	3.6	lipopolysaccharide specific response-68 protein	LSR68
202688_at		2.7	tumor necrosis factor (ligand) superfamily, member 10	TNFSF10
202687_s_at		1	Apo-2 ligand	TNFSF10
211911_x_at	1.1		MHC class I HLA B71	HLA-B
217456_x_at		1.1	MHC class I HLA-B51	HLA-B
208729_x_at	2.4		major histocompatibility complex, class I, B	HLA-B
209140_x_at	1	2	MHC HLA-B39	HLA-B39
214459_x_at		1.1	Cw1 antigen mRNA	HLA-C
211799_x_at	2.9		HLA class I heavy chain	HLA-Cw*1701
200905_x_at	1		major histocompatibility complex, class I, E	HLA-E
200904_at		1.6	major histocompatibility complex, class I, E	HLA-E
200041_s_at	1.7	1	HLA-B associated transcript-1	D6S81E
212384_at		1	HLA-B associated transcript-1	D6S81E
202431_s_at		1	v-myc avian myelocytomatosis viral oncogene homolog	MYC
201841_s_at		3	heat shock 27kD protein 1	HSPB1
217165_x_at		1.6	metallothionein 1F (functional)	MT1F
204745_x_at		1.1	metallothionein 1G	MT1G
			REPRESSED TARGETS AMONG mRNAs ENRICHED IN ALPHA6+/MHCI- CELLS	
208650_s_at	-1.5	-1.1	CD24 antigen (small cell lung carcinoma cluster 4 antigen)	CD24
209771_x_at	-1.8	-1.6	CD24 antigen (small cell lung carcinoma cluster 4 antigen)	CD24

266_s_at	-1.9	-1.5	CD24 signal transducer	
208651_x_at	-1.8		signal transducer CD24	CD24
216379_x_at	-1.8	-1.6	CD24 signal transducer	
207345_at	-2	-2.3	follistatin, transcript variant FST317	FST
204948_s_at	-1.1	-1.9	follistatin, transcript variant FST344	FST
215554_at	-2.2		glycosylphosphatidylinositol specific phospholipase D1	GPLD1

Insulin-like Growth Factor Receptor Signalling

210095_s_at		2	Human growth hormone-dependent insulin-like growth factor-binding protein mRNA	IGFBP1
212143_s_at	1.1		insulin-like growth factor binding protein 3	IGFBP3
211959_at		-1.1	Human insulin-like growth factor binding protein 5	IGFBP5
201163_s_at	-2.2	-1.6	insulin-like growth factor binding protein 7	IGFBP7
203627_at		-1.2	insulin-like growth factor 1 receptor	IGF1R
209184_s_at		-1	insulin receptor substrate 2	IRS2
201626_at		-1.2	insulin induced gene 1	
218847_at		-1	IGF-II mRNA-binding protein 2	IMP-2
203820_s_at	1.6		IGF-II mRNA-binding protein 3	KOC1

Inositol Phospholipid Signalling

203126_at		1.1	inositol(myo)-1(or 4)-monophosphatase 2	IMPA2
205376_at	-1.2		inositol polyphosphate-4-phosphatase, type II, 105kD	INPP4B
214151_s_at		-1	phosphatidylinositol glycan, class B	PIGB
214152_at		-1.2	phosphatidylinositol glycan, class B	PIGB
212680_x_at		3.9	phospholipase C, beta 3, neighbor pseudogene	PLCB3NP
216217_at	-1.4		phospholipase C, epsilon 2	PLCE2
213222_at		-2	phosphoinositide-specific phospholipase C-beta 1	KIAA0581
215554_at	-2.2		glycosylphosphatidylinositol specific phospholipase D1	GPLD1
203879_at		-1.2	phosphatidylinositol 3-kinase catalytic subunit p110delta	PIK3CD
212181_s_at		-1.7	diphosphoinositol polyphosphate phosphohydrolase type 2	NUDT4
212249_at		1	phosphoinositide-3-kinase, regulatory subunit, polypeptide 1 (p85 alpha)	PIK3R1
215938_s_at		-1.3	phospholipase A2, group VI (cytosolic, calcium-independent)	PLA2G6
203895_at	-1		phospholipase C, beta 4	PLCB4
205111_s_at		-1.7	pancreas-enriched phospholipase C	LOC51196

Ribosome Biosynthesis

208692_at		1.8	XP1PO ribosomal protein S3	rpS3
212391_x_at		1.6	ribosomal protein S3A	RPS3A
209134_s_at		1.2	ribosomal protein S6	RPS6
217747_s_at		1	ribosomal protein S9	RPS9
214001_x_at		-1.9	ribosomal protein S10	RPS10
213350_at		-1.8	ribosomal protein S11	RPS11
212114_at		-1	ribosomal protein S13	RPS13
208645_s_at		2.6	PRO2640	RPS14
201258_at		2.4	ribosomal protein S16	RPS16
213414_s_at		1.6	ribosomal protein S19	RPS19
216246_at	-1	-1.8	ribosomal protein S20	RPS20
214097_at	1.2		ribosomal protein S21	RPS21
201094_at		-1	ribosomal protein S29	RPS29
201154_x_at		1.2	ribosomal protein L4	RPL4
211710_x_at		1.2	ribosomal protein L4	RPL4
200937_s_at		3.2	ribosomal protein L5	RPL5
221989_at		1.1	ribosomal protein L10	RPL10
213588_x_at		-1	ribosomal protein L14	RPL14
200022_at		1.4	ribosomal protein L18	RPL18
214143_x_at		-1	ribosomal protein L24	RPL24
213642_at		-1.5	ribosomal protein L27	RPL27
203034_s_at		1.1	ribosomal protein L27a	RPL27a
212044_s_at	-1.1		ribosomal protein L27a	RPL27a
200003_s_at		1.6	ribosomal protein L28	RPL28
209203_s_at		2.1	ribosomal protein L30	RPL30
200002_at		1.7	ribosomal protein L35	RPL35
200092_s_at		-1	ribosomal protein L37	RPL37
214041_x_at	-1.6	-2.5	ribosomal protein L37a	RPL37a
202028_s_at		-1.4	ribosomal protein L38	RPL38
221943_x_at		-1.6	ribosomal protein L38	RPL38
210115_at	2.8	1.3	ribosomal protein L39	RPL39
208695_s_at		-1.1	ribosomal protein L39	RPL39
212199_at		1.1	putative ribosomal protein S1	
201033_x_at		1.1	ribosomal protein, large, P0	RPLP0
208856_x_at		1.4	ribosomal protein, large, P0, clone MGC:4770	RPLP0
214167_s_at		1.4	ribosomal protein, large, P0	RPLP0
211720_x_at		1.5	ribosomal protein, large, P0	RPLP0
211972_x_at		1	ribosomal protein, large, P0	RPLP0
200908_s_at		-1.3	ribosomal protein, large P2	RPLP2
209567_at		1.5	Similar to regulator for ribosome resistance homolog (S. cerevisiae)	KIAA0112
217559_at		1.1	Highly similar to A42735 ribosomal protein L10, cytosolic	ESTs
207877_s_at	3.3		nuclear VCP-like	NVL
201305_x_at	1		acidic protein rich in leucines	SSP29
AFFX-r2-Hs18SrRNA-3_s_at		1.1	Human 18S rRNA sequence, length 1969 bases, 3 prime target bases 1293-1938	
AFFX-HUMRGE/M10098_3_at		1	Human 18S rRNA gene, complete.	18S rRNA
AFFX-r2-Hs18SrRNA-M_x_at	-1.1		Human 18S rRNA sequence, length 1969 bases, middle target bases 647-1292	

Hedgehog Signalling

205201_at		-1	GLI-Kruppel family member GLI3 (Greig cephalopolysyndactyly syndrome)	GL3

Hedgehog Signalling Targets

205916_at		-4	S100 calcium-binding protein A7 (psoriasin 1)	S100A7
203320_at	-1.5		lymphocyte adaptor protein	LNK
213931_at	-1		inhibitor of DNA binding 2, dominant negative helix-loop-helix protein	ID2
208711_s_at		1.3	cyclin D1 (PRAD1: parathyroid adenomatosis 1)	CCND1
210105_s_at	-1.6	-1.8	FYN oncogene related to SRC, FGR, YES	FYN
202431_s_at		1	v-myc avian myelocytomatosis viral oncogene homolog	MYC
204734_at		1.2	keratin 15	KRT15
203213_at	1.7	1.5	cell division cycle 2, G1 to S and G2 to M	CDC2
203214_x_at	1.4		cell division cycle 2, G1 to S and G2 to M	CDC2
210559_s_at	1.2		CDC2 delta T	CDC2
201645_at	-1.3		hexabrachion (tenascin C, cytotactin)	HXB

NOTCH Signalling

204888_s_at	-2.4		neuralized (Drosophila)-like	NEURL
202360_at		-1	homolog of Drosophila mastermind	MAML1
209758_s_at	-1.2		Human microfibril-associated glycoprotein-2 MAGP-2 mRNA, complete cds.	MAGP-2

NOTCH Signalling Targets

207720_at		-1.8	loricrin	LOR
201820_at	1		keratin 5 (epidermolysis bullosa simplex, Dowling-MearaKobnerWeber-Cockayne types)	KRT5
215704_at	-2.4	-2	filaggrin	FLG

Cell Cycle

214710_s_at	2.2	1.8	cyclin B1	CCNB1
202705_at	1.7		cyclin B2	CCNB2
208711_s_at		1.3	cyclin D1 (PRAD1: parathyroid adenomatosis 1)	CCND1
200951_s_at		3.3	cyclin D2	CCND2
204995_at	-1		cyclin-dependent kinase 5, regulatory subunit 1 (p35)	CDK5R1
211297_s_at	1.5	2.3	serinethreonine kinase stk1 mRNA	CDK7
203468_at		-1	cyclin-dependent kinase (CDC2-like) 10	CDK10
213348_at	-1.1		cyclin-dependent kinase inhibitor 1C (p57, Kip2)	CDKN1C
213183_s_at	-1.5	-2.1	cyclin-dependent kinase inhibitor 1C (p57, Kip2)	CDKN1C
209644_x_at		-3.1	cyclin-dependent kinase inhibitor 2A (melanoma, p16, inhibits CDK4)	CDKN2A
210567_s_at	1.3		Similar to S-phase kinase-associated protein 2 (p45), clone MGC:1366	
209642_at	1.3		putative mitotic checkpoint kinase mRNA	BUB1
206205_at	1.3		M-phase phosphoprotein 9	MPHOSPH9
215731_s_at	1.1		mRNA for M-phase phosphoprotein	mpp9
203213_at	1.7	1.5	cell division cycle 2, G1 to S and G2 to M	CDC2
203214_x_at	1.4		cell division cycle 2, G1 to S and G2 to M	CDC2
210559_s_at	1.2		CDC2 delta T	CDC2
203968_s_at	1.6		CDC6 (cell division cycle 6, S. cerevisiae) homolog	CDC6
208022_s_at		1.3	CDC14 (cell division cycle 14, S. cerevisiae) homolog B	CDC14B
221555_x_at	-1.1		CDC14 (cell division cycle 14, S. cerevisiae) homolog B	CDC14B
204695_at	2.6		cell division cycle 25A	CDC25A
214230_at	1.2		cell division cycle 42 (GTP-binding protein, 25kD)	CDC42
208728_s_at	1.2		cell division cycle 42 (GTP-binding protein, 25kD), clone MGC:5044	CDC42
201897_s_at	1.1	1.6	CDC28 protein kinase 1	CKS1
204170_s_at	1.1		CDC28 protein kinase 2	CKS2
205393_s_at	1.7		CHK1 (checkpoint, S.pombe) homolog	CHEK1
214607_at		-1.7	p21 (CDKN1A)-activated kinase 3	PAK3
213524_s_at		-1	putative lymphocyte G0G1 switch gene	G0S2
201725_at	1.4	1.1	D123 gene product	D123
204021_s_at		1	purine-rich element binding protein A	PURA
208743_s_at		4.8	tyrosine 3-monooxygenasetryptophan5-monooxygenase activation protein, beta polypeptide	YWHAB
200638_s_at		2.2	tyrosine 3-monooxygenasetryptophan 5-monooxygenase activation protein, zeta polypeptide	YWHAZ
200640_at		1.6	tyrosine 3-monooxygenasetryptophan5-monooxygenase activation protein, zeta polypeptide	YWHAZ

Chromosome Remodeling, Replication & Repair

201291_s_at	4.5	2.1	topoisomerase (DNA) II alpha (170kD)	TOP2A
201292_at	2	2.1	topoisomerase (DNA) II alpha (170kD)	TOP2A
207168_s_at		1	H2A histone family, member Y	H2AFY
209911_x_at	-1		H2B histone family, member B	H2BFB
205659_at	-1.6	-2.5	histone deacetylase 7B (HDAC7B-PENDING)	HDAC7B-PENDING
208025_s_at	-1.8		high-mobility group (nonhistone chromosomal) protein isoform I-C	HMGIC
217937_s_at	-2.6		histone deacetylase 7A (HDAC7), transcript variant 2	HDAC7
200853_at		1.3	H2A histone family, member Z	H2AFZ
200679_x_at		2.6	high-mobility group (nonhistone chromosomal) protein 1	HMG1
200943_at		1	high-mobility group (nonhistone chromosomal) protein 14	HMG14
206052_s_at		1	Hairpin binding protein, histone	HBP
202591_s_at		1.2	single-stranded DNA-binding protein	SSBP
204146_at	1.1		RAD51-interacting protein	PIR51
200607_s_at		1.6	RAD21 (S. pombe) homolog	RAD21
209849_s_at	1.2		Rad51C	RAD51C
37793_r_at	-1.2		RAD51D mRNA	RAD51D
210416_s_at	1.1		protein kinase Chk2	RAD53
203461_at	-1.9		chromodomain helicase DNA binding protein 2	CHD2
1053_at	1.3		HUMA1SBU Human replication factor C, 40-kDa subunit (A1)	
203696_s_at	1		replication factor C (activator 1) 2 (40kD)	RFC2
211503_s_at		3.2	replication factor C (activator 1) 3 (38kD)	RFC3
204023_at	1.1		replication factor C (activator 1) 4 (37kD)	RFC4
203209_at	1.2		replication factor C (activator 1) 5 (36.5kD)	RFC5

209421_at		1.3	mutS (E. coli) homolog 2 (colon cancer, nonpolyposis type 1)	hMSH2
205887_x_at	-1.1		mutS (E. coli) homolog 3	MSH3
212913_at		-1.2	mutS (E. coli) homolog 5	MSH5
203755_at	1.6		budding uninhibited by benzimidazoles 1 (yeast homolog), beta	BUB1B
201457_x_at		1.3	kinetochore protein BUB3	BUB3
218663_at	3.9	3.6	chromosome condensation protein G	HCAP-G
218662_s_at	1.7		chromosome condensation protein G	HCAP-G
201774_s_at		1.2	chromosome condensation-related SMC-associated protein 1	KIAA0159
222037_at	1.2	3.4	minichromosome maintenance deficient (S. cerevisiae) 4	MCM4
212141_at	1		minichromosome maintenance deficient (S. cerevisiae) 4	MCM4
220651_s_at	1.2		homolog of yeast MCM10	PRO2249
218009_s_at	1.6		protein regulator of cytokinesis 1	PRC1
209172_s_at	1.4		mitosin	CENPF
204853_at	1.3		origin recognition complex, subunit 2 (yeasthomolog)-like	ORC2L
204957_at	1		origin recognition complex, subunit 5 (yeasthomolog)-like	ORC5L
205296_at	1		retinoblastoma-like 1 (p107)	RBL1
201112_s_at	1.1	2.1	chromosome segregation 1 (yeast homolog)-like	CSE1L
203362_s_at	1		MAD2 (mitotic arrest deficient, yeast, homolog)-like 1	MAD2L1
216039_at	-1.5		postmeiotic segregation increased 2-like 6 / Human PMS8 mRNA (yeast mismatch repair gene PMS1 homologue), partial cds (C-terminal region)	
207598_x_at	-1.6		X-ray repair complementing defective repair in Chinese hamster cells 2, mRNA. / X-ray repair cross complementing protein 2	XRCC2
200792_at		1.4	thyroid autoantigen 70kD (Ku antigen)	G22P1
204962_s_at	1.5	1.7	centromere protein A (17kD)	CENPA
207828_s_at	1.9		centromere protein F (350400kD, mitosin)	CENPF
201970_s_at	1.7	2.9	nuclear autoantigenic sperm protein (histone-binding)	NASP
201663_s_at	1.4		chromosome-associated polypeptide C	CAP-C
201606_s_at	1.3		nuclear phosphoprotein similar to S. cerevisiae PWP1	PWP1
215424_s_at	1.3	1.5	SKI-INTERACTING PROTEIN	SNW1
212257_s_at	-4.1		SWISNF related, matrix associated, actin dependent regulator of chromatin, subfamily a, member 2	SMARCA2
206542_s_at		1	SWISNF related, matrix associated, actin dependent regulator of chromatin, subfamily a, member 2	SMARCA2
206544_x_at	1.4		SWISNF related, matrix associated, actindependent regulator of chromatin, subfamily a, member 2	SMARCA2
202303_x_at	1		SWISNF related, matrix associated, actindependent regulator of chromatin, subfamily a, member 5	SMARCA5
203183_s_at	-1.3		SWISNF related, matrix associated, actin dependent regulator of chromatin, subfamily d, member 1	SMARCD1
209518_at		-1.4	SWISNF related, matrix associated, actin dependent regulator of chromatin, subfamily d, member 1	SMARCD1
209305_s_at		1.4	growth arrest and DNA-damage-inducible proteinGADD45beta	GADD45B
213560_at		-1.3	growth arrest and DNA-damage-inducible, beta	GADD45B
204121_at	-1.2		growth arrest and DNA-damage-inducible, gamma	GADD45G
205848_at	-2.1	-2.6	growth arrest-specific 2	GAS2
211067_s_at		-1.4	growth arrest-specific 7	
209922_at	4.4		BRCA1-associated protein 2	BRAP2
204531_s_at	1		breast cancer 1, early onset	BRCA1
205345_at	1	2.5	BRCA1 associated RING domain 1	BARD1
203764_at	1.9	1.4	KIAA0008 gene product	KIAA0008
204162_at	2.9	1.9	highly expressed in cancer, rich in leucine heptad repeats	HEC
217985_s_at		2.1	bromodomain adjacent to zinc finger domain, 1A	BAZ1A
213971_s_at		1	KIAA0160 protein	KIAA0160
218585_s_at	1.5		L2DTL protein	L2DTL
214911_s_at		1.3	bromodomain-containing 2	BRD2
208685_x_at		1	bromodomain-containing 2	BRD2
221776_s_at	1.4		bromodomain-containing 7	BRD7
213879_at		-1.6	SMT3 (suppressor of mif two 3, yeast) homolog 2	SMT3H2
204026_s_at	1.1		ZW10 interactor	ZWINT
208740_at	1.1		sin3-associated polypeptide, 18kD	SAP18
211450_s_at	1.1		Homo sapiens GTBP mRNA for GTBP-ALT, complete cds.	GTBP
213289_at		1.2	proliferation-associated 2G4, 38kD	PA2G4
221381_s_at		1.1	mortality factor 4	MORF4
213864_s_at		1	nucleosome assembly protein 1-like 1	NAP1L1
208752_x_at		1.2	nucleosome assembly protein 1-like 1	NAP1L1
202305_s_at		1.1	fasciculation and elongation protein zeta 2 (zygin II)	FEZ2
211826_s_at	-1.1		Human MLL-AF4 der(11) fusion protein mRNA, complete cds.	MLLT2
212079_s_at		2.2	myeloidlymphoid or mixed-lineage leukemia (trithorax (Drosophila) homolog)	MLL
209486_at		2.2	Homo sapiens, disrupter of silencing 10, clone MGC:11290, mRNA, complete cds.	SAS10
209715_at		1.1	chromobox homolog 5 (Drosophila HP1 alpha)	CBX5
220605_s_at	-1.4		Homo sapiens sirtuin (silent mating type information regulation 2, S.cerevisiae, homolog) 2 (SIRT2), transcript variant 1, mRNA.	SIRT2
202383_at		-1.2	SMC (mouse) homolog, X chromosome	SMCX
209579_s_at		1	methyl-CpG binding domain protein 4	MBD4

RNA Helicases

212107_s_at		3.1	DEADH (Asp-Glu-Ala-AspHis) box polypeptide 9 (RNA helicase A, nuclear DNA helicase II; leukophysin)	DDX9
201386_s_at	1.1		dead box protein 15	DDX15
208151_x_at	-1		DEADH (Asp-Glu-Ala-AspHis) box polypeptide 17,isoform 2	DDX17
217862_at		1.3	DEADH (Asp-Glu-Ala-AspHis) box binding protein 1	DDXBP1
205001_s_at	1.3	1.6	Homo sapiens dead box, Y isoform (DBY) mRNA, alternative transcript 1, complete cds.	DBY
201161_s_at		3.9	cold shock domain protein A	CSDA

Cell Proliferation / Metastasis

213906_at	1.2		v-myb avian myeloblastosis viral oncogene homolog-like 1	MYBL1
206219_s_at	-1.3		vav 1 oncogene	VAV1

205051_s_at	-2.6		v-kit Hardy-Zuckerman 4 feline sarcoma viraloncogene homolog precursor	KIT
202932_at		2.1	v-yes-1 Yamaguchi sarcoma viral oncogene homolog 1	YES1
208893_s_at		3.5	clone MGC:12852	DUSP6
201032_at		2.3	bladder cancer associated protein	BLCAP
212332_at		1.8	retinoblastoma-like 2 (p130)	RBL2
203554_x_at	1.1	1	pituitary tumor-transforming 1	PTTG1
211599_x_at		-1.4	Human (tpr-met fusion) oncogene mRNA, complete cds.	tprmet fusion
214321_at		-2	nephroblastoma overexpressed gene	NOV
201730_s_at		1.1	translocated promoter region (to activated MET oncogene)	TPR
212768_s_at	-4.5	-2	differentially expressed in hematopoietic lineages	GW112
205053_at		1	primase, polypeptide 1 (49kD)	PRIM1
204783_at	-1.1		myeloid leukemia factor 1	MLF1
207002_s_at	-1		pleiomorphic adenoma gene-like 1 isoform 1	PLAGL1
207943_x_at	-1		pleiomorphic adenoma gene-like 1 isoform 1	PLAGL1
205372_at		-1.3	pleiomorphic adenoma gene-like 1 isoform 1	PLAGL1
202925_s_at	-1.2		pleiomorphic adenoma gene-like 2	PLAGL2
203246_s_at	1.3		homologous to yeast nitrogen permease (candidatetumor suppressor)	NPR2L
219813_at	-2		LATS (large tumor suppressor, Drosophila)homolog 1	LATS1
220253_s_at		1	potential tumor suppressor	ST7
201577_at	1.2	1.2	non-metastatic cells 1 protein	NME1
203798_s_at	1.2		visinin-like 1	VSNL1
218557_at		1.6	Nit protein 2	NIT2
210517_s_at	-1.1	-1	Homo sapiens mRNA for gravin, complete cds.	AKAP12
213764_s_at		-2.8	paternally expressed 3	PEG3
209242_at		-1.2	paternally expressed 3	PEG3
200811_at		1.4	cold inducible RNA-binding protein	CIRBP
217996_at	-1		pleckstrin homology-like domain, family A, member 1	PHLDA1
206132_at	-1.1		mutated in colorectal cancers	MCC
200920_s_at		3.5	B-cell translocation gene 1, anti-proliferative	BTG1
219416_at		-1.2	CSR1 protein	CSR1
213134_x_at		1	BTG family, member 3	BTG3
201904_s_at		1.1	HYA22 protein	HYA22
205081_at		1.4	cysteine-rich protein 1 (intestinal)	CRIP1
214139_at	-1.2		RBP1-like protein	BCAA
204135_at		-1	downregulated in ovarian cancer 1	DOC1

Ribonucleoproteins and Related Genes

217829_s_at	2.6		SnRNP assembly defective 1 homolog	SAD1
200751_s_at		1.7	heterogeneous nuclear ribonucleoprotein C (C1C2)	HNRPC
212626_x_at		1.2	heterogeneous nuclear ribonucleoprotein C (C1C2)	HNRPC
209330_s_at	1.3		heterogeneous nuclear ribonucleoprotein D	HNRPD
214379_at	-1.2		heterogeneous nuclear ribonucleoprotein D-like	HNRPDL
201132_at		1	heterogeneous nuclear ribonucleoprotein H2 (H)	HNRPH2
207127_s_at	1.4		heterogeneous nuclear ribonucleoprotein H3 (2H9)	HNRPH3
210110_x_at	1.4		hnRNP 2H9D	HNRPH3
210588_x_at	1.6		ribonucleoprotein	HNRPH3
216977_x_at		1.1	U2 snRNP-specific A protein	SNRPA1
200826_at		1.5	small nuclear ribonucleoprotein D2 polypeptide(16.5kD)	SNRPD2
208174_x_at		-1	U2 small nuclear ribonucleoprotein auxiliary factor, small subunit 2	U2AF1RS2
202209_at	1.1		Lsm3 protein	LSM3
204559_s_at		4.9	U6 snRNA-associated Sm-like protein LSm7	LOC51690
211747_s_at		1.3	U6 snRNA-associated Sm-like protein	
208859_s_at		2.1	alpha thalassemiamental retardation syndrome X-linked (RAD54 (S. cerevisiae) homolog)	ATRX
208900_s_at		1.9	topoisomerase (DNA) I	TOP1
215410_at	-1.6		postmeiotic segregation increased 2-like 8	PMS2L14
200072_s_at		1.3	Homo sapiens M4 protein deletion mutant mRNA, complete cds.	HNRPM
205443_at		1.2	small nuclear RNA activating complex,polypeptide 1, 43kD	SNAPC1
209104_s_at		1.5	likely homolog of yeast Nhp2, component of theHACA snoRNP; hypothetical protein FLJ20479	NOLA2

Related to Mitochondria

219645_at	-1.3	-1.6	calsequestrin 1 (fast-twitch, skeletal muscle), nuclear gene encoding mitochondrial protein, mRNA	CASQ1
221437_s_at	1.3	2.6	mitochondrial ribosomal protein S15	MRPS15
201322_at		1	ATP synthase, H+ transporting, mitochondrial F1 complex, beta polypeptide	ATP5B
205711_x_at		2.3	ATP synthase, H+ transporting, mitochondrial F1 complex, gamma polypeptide 1	ATP5C1
213366_x_at		1.5	ATP synthase, H+ transporting, mitochondrial F1 complex, gamma polypeptide 1	ATP5C1
207573_x_at		1	ATP synthase, H+ transporting, mitochondrial F1F0, subunit g	ATP5JG
202961_s_at	1		ATP synthase, H+ transporting, mitochondrial F0 complex, subunit f, isoform 2	ATP5J2
200818_at		1.2	ATP synthase, H+ transporting, mitochondrial F1 complex, O subunit (oligomycin sensitivity conferring protein)	ATP5O
201634_s_at		2	cytochrome b5 outer mitochondrial membrane precursor	CYB5-M
201931_at		1.9	electron-transfer-flavoprotein, alpha polypeptide (glutaric aciduria II), nuclear gene encoding mitochondrial protein, mRNA	ETFA
211855_s_at	1.5		mitochondrial uncoupling protein 5 short form with insertion mRNA	SLC25A14
204587_at	1.1		solute carrier family 25 (mitochondrial carrier, brain), member 14, transcript variant long, nuclear gene encoding mitochondrial protein, mRNA	SLC25A14
212085_at		1.6	solute carrier family 25 (mitochondrial carrier; adenine nucleotide translocator), member 6	SLC25A6
217955_at		1.5	MIL1 protein, nuclear gene encoding mitochondrial protein, mRNA	MIL1
209932_s_at		1.1	deoxyuridine triphosphate nucleotidohydrolase precursor mRNA, nuclear gene encoding mitochondrial protein, complete cds	DUT
207275_s_at		-1.4	fatty-acid-Coenzyme A ligase, long-chain 1, nuclear gene encoding mitochondrial protein, mRNA	FACL1
203945_at		-1.4	arginase, type II, nuclear gene encoding mitochondrial protein, mRNA	ARG2
210069_at		-1.4	palmitoyltransferase I mRNA, nuclear gene encoding mitochondrial protein, complete cds	CPT1B
202002_at		-1.5	acetyl-Coenzyme A acyltransferase 2 (mitochondrial 3-oxoacyl-Coenzyme A thiolase)	ACAA2
211150_s_at	3		M2 mitochondrial autoantigen dihydrolipoamide acetyltransferase	DLAT

211594_s_at	2		MRPL9 mRNA for mitochondrial ribosomal protein L9 (L9mt)	MRPL9
218119_at	1.6		translocase of inner mitochondrial membrane 23 (yeast) homolog	TIM23
218118_s_at	1.6		translocase of inner mitochondrial membrane 23 (yeast) homolog	TIM23
210154_at	1.4		mitochondrial NAD(P)+ dependent malic enzyme mRNA	NAD(P)+ - dependent malic enzyme
202712_s_at	1		creatine kinase, mitochondrial 1 (ubiquitous), nuclear gene encoding mitochondrial protein, mRNA	CKMT1
36830_at	1.2		mitochondrial intermediate peptidase precursor	MIPEP
217772_s_at	1.1		mitochondrial carrier homolog 2, nuclear gene encoding mitochondrial protein	MTCH2
204389_at	1		monoamine oxidase A, nuclear gene encoding mitochondrial protein, mRNA	MAOA
210418_s_at	1		NAD+-specific isocitrate dehydrogenase beta subunit isoform A mRNA, nuclear gene encoding mitochondrial product, complete cds	IDH3B
202785_at		2.6	NADH dehydrogenase (ubiquinone) 1 alpha subcomplex, 7 (14.5kD, B14.5a)	NDUFA7
201226_at		2.6	NADH dehydrogenase (ubiquinone) 1 beta subcomplex, 8 (19kD, ASHI)	NDUFB8
202941_at	1		NADH dehydrogenase (ubiquinone) flavoprotein 2 (24kD)	NDUFV2
203039_s_at	1.1		NADH dehydrogenase (ubiquinone) Fe-S protein 1 (75kD) (NADH-coenzyme Q reductase)	NDUFS1
210800_at	-1.3	-1.2	Homo sapiens, clone MGC:12262, mRNA, complete cds.	TIMM8A

Apoptosis

208945_s_at		1	beclin 1 (coiled-coil, myosin-like BCL2-interacting protein)	BECN1
202984_s_at		3.3	BCL2-associated athanogene 5	BAG5
202036_s_at		1.3	secreted apoptosis related protein 2	SARP2
222158_s_at		1.9	apoptosis-related protein PNAS-4	PNAS-4
210792_x_at	2.4		Siva-2 mRNA	SIVA-2
203139_at	-2		death-associated protein kinase 1	DAPK1
209323_at		1.4	death associated protein 4	DAP4
220864_s_at		2	CGI-39 protein; cell death-regulatory protein GRIM19	LOC51079
211367_s_at	1.5		interleukin 1-beta converting enzyme isoform delta	IL1BCE
211368_s_at	1.1		interleukin 1-beta converting enzyme isoform epsilon	IL1BCE
211366_x_at	1		interleukin 1-beta converting enzyme isoform gamma	IL1BCE
220451_s_at		-1.1	livin inhibitor-of-apotosis	LIVIN
204237_at	-1.2	-1	CED-6 protein	CED-6
204235_s_at	-1.6		PTB domain adaptor protein CED-6	CED-6
203089_s_at		2.6	HtrA-like serine protease	OMI
213762_x_at		1	RNA binding motif protein, X chromosome	RBMX
216842_x_at		-1.8	Human RNA binding motif (RBM) gene, partial cds	
214618_at	-1.4		Usurpin-gamma	CFLAR
205063_at		1.4	survival of motor neuron protein interactingprotein 1	SIP1

Growth Factor Related

216061_x_at	-1	-1.1	platelet-derived growth factor beta polypeptide (simian sarcoma viral (v-sis) oncogene homolog)	PDGFB
217886_at		-1	epidermal growth factor receptor pathway substrate 15	EPS15
222113_s_at	-1	-1.3	epidermal growth factor receptor substrate EPS15R	EPS15R
209409_at	-1.1	-1.8	growth factor receptor-bound protein 10	KIAA0207
35150_at	-1.2		CDw40 mRNA for nerve growth factor receptor-related B-lymphocyte activation molecule	
219304_s_at	-1.2	-1.4	spinal cord-derived growth factor-B	SCDGF-B
205782_at	-1.5		fibroblast growth factor 7 (keratinocyte growth factor)	FGF7
209466_x_at	-1.5		nerve growth factor	HBNF-1
206404_at	-1	-1.7	fibroblast growth factor 9 (glia-activating factor)	FGF9

Transmembrane Molecules and Related genes

Receptors

211535_s_at		-2.5	fibroblast growth factor receptor	FGFR1
213816_s_at	-1.1		met proto-oncogene (hepatocyte growth factor receptor)	MET
205696_s_at		-1.5	GDNF family receptor alpha 1	GFRA1
204464_s_at	-1.1	-1.4	endothelin receptor type A	EDNRA
204273_at	-2.8	-1.3	endothelin receptor type B	EDNRB
204271_s_at	-2	-1.3	endothelin receptor	ETs
217045_x_at	-1.1		lymphocyte antigen 95 (activating NK-receptor ; NK-p44)	LY95
211516_at	-1.1		interleukin 5 receptor alpha-subunit	IL5R
220056_at	-1.1		interleukin 22 receptor	IL22R
221444_at	-1.2		taste receptor, type 2, member 16	TAS2R16
205159_at	-1.2		colony stimulating factor 2 receptor, beta, low-affinity (granulocyte-macrophage)	CSF2RB
203989_x_at	-1.2		coagulation factor II (thrombin) receptor	F2R
209894_at	-1.2		leptin receptor short form	db
205926_at	-1.2		class I cytokine receptor	WSX-1
209822_s_at	-1.3		very low density lipoprotein receptor	VLDLR
213193_x_at	-1.3		T cell receptor beta locus	TRB@
217033_x_at	-1.4		neurotrophic tyrosine kinase, receptor, type 3	trkC
212588_at	-1.4		mRNA for T200 leukocyte common antigen (CD45, LC-A).	PTPRC
216837_at	-1.4		receptor protein-tyrosine kinase	HEK7
211846_s_at	-1.4		herpesvirus immunoglobulin-like receptor HIgR	PVRL1
221464_at	-1.7	-2	olfactory receptor, family 1, subfamily D, member 2	OR1D2
214515_at	-1.7		olfactory receptor, family 1, subfamily E, member 1	OR1E1
221451_s_at	-1.5		olfactory receptor, family 2, subfamily W, member 1	OR2W1
215770_at		-4.6	olfactory receptor, family 7, subfamily E, member 2 pseudogene	OR7E2P
215463_at	-1.4		olfactory receptor, family 7, subfamily E, member 24 pseudogene	OR7E24P
208520_at	-1.8		olfactory receptor, family 10, subfamily H, member 3	OR10H3
216408_at	-1.8		6M1-10*01 gene for olfactory receptor, cell line BM28.7	
207633_s_at	-1.5		muscle, skeletal, receptor tyrosine kinase	MUSK
217060_at	-1.6		V beta T-cell receptor gene locus	TCRBV
217484_at	-1.7		complement component (3b4b) receptor 1, including Knops blood group system	CR1
205209_at		1.1	activin A receptor, type IB	ACVR1B

205945_at		1	interleukin 6 receptor	IL6R
207840_at		-1.3	natural killer cell receptor, immunoglobulin superfamily member	BY55
203749_s_at		-1.3	retinoic acid receptor, alpha	RARA
214866_at		-1.3	mRNA for urokinase plasminogen activator receptor	PLAUR
217028_at		-1.7	chemokine (C-X-C motif), receptor 4 (fusin)	CXCR4
209354_at		-1.8	tumor necrosis factor receptor superfamily, member 14 (herpesvirus entry mediator)	TNFRSF14
211837_s_at		-2.9	pre-T-cell receptor alpha chain	PTA
214770_at		-3.3	macrophage scavenger receptor 1	MSR1
213880_at	-1.6	-2	G protein-coupled receptor 49	GPR49
212070_at	-1.2		G protein-coupled receptor 56	GPR56
221394_at	-1.6		G protein-coupled receptor 58	GPR58
206002_at	-2.1	-2.3	G protein-coupled receptor 64	GPR64
219936_s_at		1.3	G protein-coupled receptor 87	GPR87
214506_at	-1.3		G-protein coupled receptor similar to theadrenomedullin receptor	ADMR
219789_at		1.4	natriuretic peptide receptor Cguanylate cyclase C (atrionatriuretic peptide receptor C)	NPR3
210412_at	-1.8		Human N-methyl-D-aspartate receptor subunit NR3 (hNR3) mRNA, complete cds.	NR3
210832_x_at	-1.5		prostaglandin E receotor EP3 subtype 1b isoform	PTGER3
210374_x_at	-1		prostaglandin E receptor EP3 subtype 4 isoform	PTGER3
210055_at		-4.6	thyroid stimulating hormone receptor	TSHR
210729_at	-3.7		Human type 2 neuropeptide Y receptor mRNA, complete cds.	NPY2R
213436_at	-1.4		cannabinoid receptor 1 (brain)	CNR1
206346_at	-1.1		prolactin receptor	PRLR
219282_s_at	-1.2		vanilloid receptor-like protein 1	VRL-1
202820_at	-1		aryl hydrocarbon receptor	AHR
221107_at	-1.2		cholinergic receptor, nicotinic, alphapolypeptide 9	CHRNA9
201591_s_at		-1.2	imidazoline receptor candidate	I-1
209866_s_at	-1.2		KIAA0768 protein	KIAA0768

solute carrier family proteins

203580_s_at		-1.2	solute carrier family 7 (cationic amino acidtransporter, y+ system), member 6	SLC7A6
206143_at		-4.7	down-regulated in adenoma protein	SLC26A3
206529_x_at	-1.2		pendrin	SLC26A4
201802_at	1.3		solute carrier family 29 (nucleosidetransporters), member 1	SLC29A1

Other Transmembrane/ Cell Surface Proteins

207291_at	1.1		transmembrane gamma-carboxyglutamic acid protein 4	TIMG4
207847_s_at	-1		mucin 1, transmembrane	MUC1
217117_x_at	-1.3		mucin	MUC3
208349_at	-1.1		ankyrin-like with transmembrane domains 1	ANKTM1
220205_at	-1.6		transmembrane phosphatase with tensin homology	TPTE
203824_at	-1.7		transmembrane 4 superfamily member 3	TM4SF3
209263_x_at	-1.8		transmembrane 4 superfamily member 7	TM4SF7
204808_s_at		1.4	transmembrane protein 5	TMEM5
205122_at		-1.2	transmembrane protein with EGF-like and two follistatin-like domains 1	TMEFF1
220454_s_at		-1.4	sema domain, transmembrane domain (TM), and cytoplasmic domain, (semaphorin) 6A	SEMA6A
209498_at		-1.8	mRNA for transmembrane carcinoembryonic antigen BGPa (formerly TM1-CEA)	CEACAM1
214606_at	-1.3		tetraspan 2	TSPAN-2
200973_s_at	1.2		tetraspan 3	TSPAN-3
203456_at		-3.4	JM4 protein	JM4
214594_x_at	-1.3		ATPase, Class I, type 8B, member 1	ATP8B1
214934_at		-1.2	ATPase, Class II, type 9B	ATP9B
214255_at		-1.8	ATPase, Class V, type 10C	ATP10C
221898_at	1		lung type-I cell membrane-associated glycoprotein	T1A-2
201195_s_at		1	Homo sapiens mRNA for L-type amino acid transporter 1, complete cds.	hLAT1
219025_at	-1.2		tumor endothelial marker 1 precursor	TEM1
214650_x_at	-1.4		myelin oligodendrocyte glycoprotein	MOG
31835_at	-1.4		Human histidine-rich glycoprotein mRNA, complete cds	
218625_at	-1.4		neuritin	LOC51299
211373_s_at		-2.1	seven trans-membrane domain protein AD3LPAD5	AD3LPAD5
219643_at		-2.1	low density lipoprotein-related protein 1B (deleted in tumors)	LRP1B
205674_x_at		-1.3	FXYD domain-containing ion transport regulator2, isoform 1	FXYD2

Antigens

204055_s_at	-2		meningioma expressed antigen 6 (coiled-coil proline-rich)	MGEA6
206218_at	-1.6		melanoma antigen, family B, 2	MAGEB2
206276_at	2		lymphocyte antigen 6 complex, locus D	E48
218715_at	1		hepatocellular carcinoma-associated antigen 66	HCA66
205668_at	-1		lymphocyte antigen 75	LY75
210325_at	-1		thymocyte antigen CD1a	CD1a
206816_s_at	-1.1		sperm associated antigen 8	SPAG8
212468_at	-1.2		sperm associated antigen 9	SPAG9
207198_s_at	-1.3		LIM and senescent cell antigen-like domains 1	LIMS1
209719_x_at	-1.3		squamous cell carcinoma antigen 1	SCCA1
201005_at		1.9	CD9 antigen (p24)	CD9
211189_x_at	-1.4		leukocyte differentiation antigen CD84 isoform CD84a	CD84
208650_s_at	-1.5	-1.1	CD24 antigen (small cell lung carcinoma cluster 4 antigen)	CD24
209771_x_at	-1.8	-1.6	CD24 antigen (small cell lung carcinoma cluster 4 antigen)	CD24
266_s_at	-1.9	-1.5	CD24 signal transducer	
208651_x_at	-1.8		signal transducer CD24	CD24
216379_x_at	-1.8	-1.6	CD24 signal transducer	
206545_at	-1.6		CD28 antigen (Tp44)	CD28
203416_at	-1.6		CD53 antigen	CD53
207176_s_at	-1.6		CD80 antigen (CD28 antigen ligand 1, B7-1 antigen)	CD80
210413_x_at	-1.6		squamous cell carcinoma antigen 2	SCCA2
212607_at	-1.8		serologically defined colon cancer antigen 8	SDCCAG8
207437_at		-2.1	neuro-oncological ventral antigen 1	NOVA1

205794_s_at	-1.8	-1.6	neuro-oncological ventral antigen 1	NOVA1
201644_at		3	tissue specific transplantation antigen P35B	TSTA3
200598_s_at		2.3	tumor rejection antigen (gp96) 1	TRA1
212022_s_at		2	antigen identified by monoclonal antibody Ki-67	Ki-67
211744_s_at		1.5	Similar to CD58 antigen, (lymphocyte function-associated antigen 3)	
217523_at		1.2	Moderately similar to CD44_HUMAN CD44 ANTIGEN PRECURSOR	ETS
220957_at	-1.6		CTAGE-1 protein	CTAGE-1
203996_s_at		-1.1	chromosome 21 open reading frame 2	C21ORF2
216213_at	-1.4		Homo sapiens NY-REN-55 antigen mRNA, partial cds.	NEK1
211657_at	-1.5		non-specific cross reacting antigen	NCA
211906_s_at	-1.6		SCCA2b	SCCA2
207483_s_at		1.1	TIP120 protein	TIP120
201138_s_at		1.3	Sjogren syndrome antigen B (autoantigen La)	SSB
			Major Hictocompatibility Complex and Antigen Presenting	
211911_x_at	1.1		MHC class I HLA B71	HLA-B
217456_x_at		1.1	MHC class I HLA-B51	HLA-B
208729_x_at	2.4		major histocompatibility complex, class I, B	HLA-B
209140_x_at	1	2	MHC HLA-B39	HLA-B39
214459_x_at		1.1	Cw1 antigen mRNA	HLA-C
211799_x_at	2.9		HLA class I heavy chain	HLA-Cw*1701
203932_at	-1		major histocompatibility complex, class II, DM beta	HLA-DMB
221491_x_at	-2		major histocompatibility complex, class II, DR beta 3	HLA-DRB3
208306_x_at	-1		major histocompatibility complex, class II, DR beta 4	HLA-DRB4
200905_x_at		1	major histocompatibility complex, class I, E	HLA-E
200904_at		1.6	major histocompatibility complex, class I, E	HLA-E
200041_s_at	1.7	1	HLA-B associated transcript-1	D6S81E
212384_at		1	HLA-B associated transcript-1	D6S81E
203712_at	1.1		KIAA0020 gene product	KIAA0020
35974_at	1		Human lymphoid-restricted membrane protein (Jaw1) mRNA, complete cds	
			Cell Adhesion	
201952_at		-1.6	activated leucocyte cell adhesion molecule	ALCAM
201884_at	-1.6		carcinoembryonic antigen-related cell adhesion molecule 5	CEACAM5
204584_at		-2.5	L1 cell adhesion molecule (hydrocephalus, stenosis of aqueduct of Sylvius 1, MASA (mental retardation, aphasia, shuffling gait and adducted thumbs) syndrome, spastic paraplegia 1)	L1CAM
211340_s_at	-1	-1.5	MUC18 glycoprotein mRNA	MCAM
205669_at	-1.5		neural cell adhesion molecule 2	NCAM2
216959_x_at	-1.1		neuronal cell adhesion molecule / hBRAVONr-CAM precursor (hBRAVONr-CAM) gene	NRCAM
203868_s_at	-1.2		vascular cell adhesion molecule 1	VCAM1
206049_at	-1.8		selectin P (granule membrane protein 140kD, antigen CD62)	SELP
213325_at		-1.8	nectin 3	DKFZP566B0846
214705_at		1.2	Homo sapiens mRNA for PDZ domain protein	INADL
211701_s_at	-1.3	-1.3	magphinin beta	TRO
			Desmosomal Proteins	
204750_s_at		1.8	desmocollin 2	DSC2
206642_at		1.4	desmoglein 1 preproprotein	DSG1
207717_s_at	-1.8		plakophilin 2	PKP2
201927_s_at		4.2	plakophilin 4	PKP4
			Integrins	
206766_at	-1.1	-1.4	integrin alpha 10 subunit	ITGA10
201124_at	-1.1		integrin, beta 5	ITGB5
201125_s_at		-1	integrin, beta 5	ITGB5
			heparan sulfate proteoglycans	
202071_at	1		syndecan 4 (amphiglycan, ryudocan)	SDC4
			Collagens	
202311_s_at		-2	collagen, type I, alpha 1	COL1A1
202403_s_at	-1.3	-2	collagen, type I, alpha 2	COL1A2
202404_s_at		-3.7	collagen, type I, alpha 2	COL1A2
211161_s_at	-1		PRO3121	COL3A1
211980_at	-1.9	-1.2	collagen, type IV, alpha 1	COL4A1
211343_s_at		-1.8	(clones HT-(125,133)) alpha-2 type IV collagen	COL4A2
216368_s_at	-1		collagen type IV alpha 3	COL4A3
203325_s_at		-1.1	collagen, type V, alpha 1	COL5A1
213428_s_at		-1.1	collagen, type VI, alpha 1	COL6A1
213622_at	-1.5	-1.6	collagen, type IX, alpha 2	COL9A2
37892_at		-3.7	alpha-1 type XI collagen	COL11A1
216993_s_at	-1		collagen, type XI, alpha 2	COL11A2
212865_s_at	-1.3	-1.1	collagen, type XIV, alpha 1 (undulin)	COL14A1
204636_at		2.2	collagen, type XVII, alpha 1	COL17A1
			Cadherins	
207173_x_at	-1.8	-2.4	mRNA for OB-cadherin-1	osf-4
209079_x_at	-1		protocadherin gamma A1	PCDH-gamma-A1
205717_x_at	-1		protocadherin gamma subfamily C, 3	PCDHGC3
206935_at		-1.8	protocadherin 8	PCDH8
205656_at	-1		protocadherin 17	PCDH17

220115_s_at	-1.7	-1.2	cadherin 10, type 2 (T2-cadherin)	CDH10
206898_at	-1.6		cadherin 19, type 2	CDH19

Tight Junctions

202085_at		1.1	tight junction protein 2 (zona occludens 2)	TJP2

Channel Related

212038_s_at		1.9	voltage-dependent anion channel 1	VDAC1
217140_s_at	1.6		voltage-dependent anion channel 1 pseudogene	VDAC1P
208846_s_at	1.3		voltage dependent anion channel form 3	VDAC3
214933_at		-1.4	calcium channel, voltage-dependent, PQ type, alpha 1A subunit	CACNA1A
213714_at	1.1		calcium channel, voltage-dependent, beta 2 subunit	CACNB2
208578_at	-1.4		sodium channel, voltage-gated, type X, alpha polypeptide	SCN10A
210853_at	-1		voltage-gated sodium channel alpha subunit, alternate splice variant SCN12A-s	SCN12A
203402_at		-1.3	potassium voltage-gated channel, shaker-related subfamily, beta member 2	KCNAB2
220776_at	-1.1		potassium inwardly-rectifying channel,subfamily J, member 14	KCNJ14
219883_at	-1.3		potassium inwardly-rectifying channel, subfamily K, member 4	KCNK4
218084_x_at	-1.4		FXYD domain-containing ion transport regulator 5	FXYD5
205803_s_at	-2.1		transient receptor potential channel 1	TRPC1
206165_s_at		1.7	calcium activated chloride channel 2 precursor	CLCA2
201732_s_at		3.8	chloride channel protein 3	CLCN3
38069_at		-1.3	mRNA for CLC-7 chloride channel protein	
208659_at		1.6	chloride channel ABP	CLIC1

Multi Drug Resistance Transporters

208635_x_at		1.9	ATP-binding cassette, sub-family B (MDRTAP), member 2	ABCB2
208161_s_at		-1.1	Homo sapiens ATP-binding cassette, sub-family C (CFTRMRP), member 3 (ABCC3), transcript variant MRP3A, mRNA.	ABCC3
203981_s_at		1.8	ATP-binding cassette, sub-family D (ALD), member 4	ABCD4

Extracellular Matrix

211985_s_at	1.1		matrix Gla protein	MGP
206580_s_at		-1.2	EGF-containing fibulin-like extracellular matrix protein 2	EFEMP2
213993_at		-3.9	spondin 1, (f-spondin) extracellular matrix protein	SPON1
207289_at		-1.3	matrix metalloproteinase-like 1	MMPL1
203936_s_at	-1.5		matrix metalloproteinase 9 (gelatinase B, 92kD gelatinase, 92kD type IV collagenase)	MMP9
214913_at	-1.5		a disintegrin-like and metalloprotease (reprolysin type) with thrombospondin type 1 motif, 3	KIAA0366
219935_at	-1.6		a disintegrin-like and metalloprotease (reprolysin type) with thrombospondin type 1 motif, 5 (aggrecanase-2)	ADAMTS5
220287_at	-1.3		disintegrin and metalloproteinase withthrombospondin motifs-9 preproprotein	ADAMTS9
207665_at	-1.3		a disintegrin and metalloproteinase domain 21	ADAM21
207165_at	2.1	1.6	hyaluronan-mediated motility receptor (RHAMM), transcript variant 2	HMMR
209709_s_at	1.4		hyaluronan receptor	RHAMM
206010_at	-1.3		hyaluronan-binding protein 2	HABP2
203184_at	-1		fibrillin 2 (congenital contractural arachnodactyly)	FBN2
206134_at	-1.2		disintegrin protease	M12.219
221731_x_at	-1.5		chondroitin sulfate proteoglycan 2 (versican)	CSPG2
204620_s_at	-2	-1.4	chondroitin sulfate proteoglycan 2 (versican)	CSPG2
200795_at	-1.7		SPARC-like 1 (mast9, hevin)	SPARCL1
219087_at	1.9	2.2	asporin (LRR class 1)	ASPN
204614_at		1	serine (or cysteine) proteinase inhibitor, cladeB (ovalbumin), member 2	SERPINB2
209720_s_at		-1.6	serine (or cysteine) proteinase inhibitor, cladeB (ovalbumin), member 3	SERPINB3
206421_s_at	1.8	4	serine (or cysteine) proteinase inhibitor, clade B (ovalbumin), member 7	SERPINB7
200986_at	-1		complement component 1 inhibitor precursor	SERPING1
205352_at	-1.2		protease inhibitor 12 (neuroserpin)	SERPINI1
200654_at		4.8	procollagen-proline, 2-oxoglutarate 4-dioxygenase (proline 4-hydroxylase), beta polypeptide (protein disulfide isomerase; thyroid hormone binding protein p55)	P4HB
219625_s_at		2.4	collagen, type IV, alpha 3 (Goodpasture antigen) binding protein	COL4A3BP
201666_at		1.4	tissue inhibitor of metalloproteinase 1 (erythroid potentiating activity, collagenase inhibitor)	TIMP1
216250_s_at	-1.3		laminin, alpha 3 (nicein (150kD), kalinin (165kD), BM600 (150kD), epilegrin)	LAMA3
202202_s_at		-1.4	laminin, alpha 4 precursor	LAMA4
215516_at		2	laminin, beta 4	LAMB4
206400_at		1.8	lectin, galactoside-binding, soluble, 7 (galectin 7)	LGALS7
208934_s_at	1.4		colorectal carcinoma-derived galectin-8 variant I	LGALS8
210731_s_at		-1.2	lectin, galactoside-binding, soluble, 8 (galectin 8)	LGALS8
204736_s_at		-2.1	melanoma-associated chondroitin sulfateproteoglycan 4	CSPG4
214297_at		-2.1	chondroitin sulfate proteoglycan 4 (melanoma-associated)	CSPG4
202709_at	-1		fibromodulin precursor	FMOD
204987_at	-1.9		inter-alpha (globulin) inhibitor, H2 polypeptide	ITIH2
217161_x_at		-1.5	cartilage specific proteoglycan (600 AA)	AGC1
209396_s_at		-2.6	Human glycoprotein mRNA, complete cds	CHI3L1
202363_at	-1.3		testican-1	SPOCK
209395_at		-1.5	glycoprotein	CHI3L1
209732_at		1.4	Similar to C-type (calcium dependent,carbohydrate-recognition domain) lectin, superfamilymember 2 (activation-induced)	CLECSF2
205871_at		-1.4	Similar to plasminogen	PLGL

Cytoskeleton

213515_x_at	-1.9		myosin, light polypeptide 4, alkali; atrial, embryonic	MYL4
205144_at	-1.5		regulatory myosin light chain	MYL5
212372_at		-1.1	Homo sapiens cDNA: FLJ23324 fis, clone HEP12482, highly similar to HUMMYOHCB Human nonmuscle myosin heavy chain-B (MYH10) mRNA.	
203215_s_at		2.8	myosin VI	MYO6
220288_at	-1.1		myosin XV	MYO15

220319_s_at	1.1		myosin regulatory light chain interacting protein	MIR
206891_at		-1.1	skeletal muscle specific actinin, alpha 3	ACTN3
200728_at		2	ARP2 (actin-related protein 2, yeast) homolog	ACTR2
200996_at		1.8	ARP3 (actin-related protein 3, yeast) homolog	ACTR3
211995_x_at		1.4	actin, gamma 1	ACTG1
212363_x_at		1	actin, gamma 1	ACTG1
208679_s_at		2.2	Homo sapiens PNAS-139 mRNA, complete cds.	ARPC2
209344_at		4.3	tropomyosin 4	TPM4
200696_s_at	-1		gelsolin (amyloidosis, Finnish type)	GSN
214331_at	2.7		advillin	AVIL
218252_at	1		cytoskeleton associated protein 2	CKAP2
204444_at	1.2		kinesin-like 1	KNSL1
219306_at	1.3		kinesin-like protein 2	hklp2
204709_s_at	1.4		kinesin-like 5 (mitotic kinesin-like protein 1)	KNSL5
218755_at	1.2		RAB6 interacting, kinesin-like (rabkinesin6)	RAB6KIFL
210527_x_at		2.7	alpha-tubulin	TUBA2
209118_s_at		-1.1	hum-a-tub2 alpha-tubulin mRNA	TUBA3
214023_x_at		-1.5	tubulin, beta polypeptide	TUBB
213476_x_at		3.2	tubulin, beta, 4	TUBB4
203894_at	-1.1		tubulin, gamma 2	TUBG2
213266_at		-1.3	gamma tubulin ring complex protein (76p gene)	76P
201975_at		-1	restin (Reed-Steinberg cell-expressed intermediate filament-associated protein)	RSN
207780_at	-1.4		cylicin, basic protein of sperm head cytoskeleton 2	CYLC2
213201_s_at		3.5	troponin T1, skeletal, slow	TNNT1
206393_at	1.4		troponin I, skeletal, fast	TNNI2
217234_s_at		3.3	cytovillin 2	VIL2
210461_s_at		1.2	Homo sapiens, Similar to actin binding LIM protein 1, clone MGC:1224, mRNA, complete cds	ABLIM
201615_x_at		1.2	caldesmon 1	CALD1
200906_s_at		1.1	palladin	KIAA0992
213371_at		-1.5	Z-band alternatively spliced PDZ-motif	ZASP
206807_s_at	-1.2		adducin 2, isoform b	ADD2
205882_x_at		1.7	adducin 3 (gamma)	ADD3
201752_s_at		1.3	adducin 3 (gamma)	ADD3
217892_s_at		1.6	epithelial protein lost in neoplasm beta	EPLIN
205399_at		-1.6	doublecortin and CaM kinase-like 1	DCAMKL1
202806_at		-1.5	drebrin 1	DBN1
221748_s_at		-1.3	tensin	TSN
203243_s_at		1.1	LIM protein (similar to rat protein kinaseC-binding enigma)	LIM
214174_s_at		-1.1	LIM domain protein	RIL
221236_s_at	-1.2		stathmin-like-protein RB3	RB3
207724_s_at	-1.5		spastin	SPG4
212551_at		-1.4	adenylyl cyclase-associated protein 2	CAP2

Neurofilaments

221805_at		-1.3	neurofilament, light polypeptide (68kD)	NEFL
205113_at		-2.2	neurofilament 3 (150kD medium)	NEF3
204465_s_at	-1.3		internexin neuronal intermediate filament protein, alpha	INA

Vimentinlike Proteins

201426_s_at	-1.7		vimentin	VIM

Keratins

207908_at	-1.1		keratin 2A (epidermal ichthyosis bullosa of Siemens)	KRT2A
213240_s_at		-2.2	mRNA for cytokeratin 4 C-terminal region	KRT4
201820_at	1		keratin 5 (epidermolysis bullosa simplex, Dowling-MearaKobnerWeber-Cockayne types)	KRT5
209126_x_at	1		keratin 6 isoform K6f	KRT6F
207716_at	-1.4		keratin, hair, acidic, 8	KRTHA8
204734_at		1.2	keratin 15	KRT15

Related to Immune System

212764_at	-1.1		transcription factor 8 (represses interleukin 2 expression)	TCF8
208930_s_at		1.7	interleukin enhancer binding factor 3, 90kD	ILF3
204863_s_at		1.7	interleukin 6 signal transducer (gp130, oncostatin M receptor)	IL6ST
207315_at	-2.2		adhesion glycoprotein	DNAM-1
204777_s_at	-2	-1.2	T-cell differentiation protein MAL, isoform a	MAL
823_at	-1		Human CX3C chemokine precursor, mRNA, alternatively spliced, complete cds	
221963_x_at	-1.1		thrombospondin 1	THBS1
201110_s_at	-2.3	-1.9	thrombospondin 1	THBS1
215775_at	-1.3		Human thrombospondin-1 gene, partial cds	
202510_s_at		-1	tumor necrosis factor, alpha-induced protein 2	TNFAIP2
206181_at	-1.2		signaling lymphocytic activation molecule	SLAM
214617_at		-1.6	perforin 1 (pore forming protein)	PRF1
205863_at	-1.9		S100 calcium-binding protein A12	S100A12
209447_at		1.2	lymphocyte membrane associated protein	8B7
214650_x_at	-1.4		myelin oligodendrocyte glycoprotein	MOG
207777_s_at	-1.5		nuclear body protein Sp140	SP140
214119_s_at		-1.2	FK506-binding protein 1A (12kD)	FKBP1A
217923_at		1.4	peflin	PEF
221323_at	-1.3		UL16-binding protein 1	ULBP1
202019_s_at	-1.1		LanC (bacterial lantibiotic synthetase component C)-like 1	LANCL1

Interferon Signalling

214569_at	-2.9		interferon, alpha 5	IFNA5

208548_at		-4	interferon, alpha 6	IFNA6
208259_x_at	-1.4		interferon, alpha 7	IFNA7
205170_at	-1.5	-1.4	signal transducer and activator of transcription 2, 113kD	STAT2
214462_at	-1		STAT induced STAT inhibitor-4	CIS4
214105_at		-1.6	STAT induced STAT inhibitor 3	SSI-3
205841_at		2.8	Janus kinase 2 (a protein tyrosine kinase)	JAK2
203153_at		3	interferon-induced protein with tetratricopeptide repeats 1	IFIT1
210163_at		1.9	interferon stimulated T-cell alpha chemoattractant precursor	SCYB11
208965_s_at		1.4	interferon, gamma-inducible protein 16	IFI16

Immunoglobulin Related Molecules

216372_at	-1.1		clone N97 immunoglobulin heavy chain variable region mRNA	
216371_at	-1.1		clone N97 immunoglobulin heavy chain variable region mRNA	
206420_at	-1.4		immunoglobulin superfamily, member 6	IGSF6
216034_at	-1.5	-1.9	immunoglobulin lambda gene locus DNA, clone:288A10	
216557_x_at	-1.6		mRNA for single-chain antibody / Human rearranged immunoglobulin heavy chain (A1VH3) gene, partial cds	
203788_s_at	-1.7		sema domain, immunoglobulin domain (Ig), short basic domain, secreted, (semaphorin) 3C	SEMA3C
203789_s_at	-1.9		sema domain, immunoglobulin domain (Ig), short basic domain, secreted, (semaphorin) 3C	SEMA3C
35666_at		-1.2	Human semaphorin III family homolog mRNA, complete cds	
217193_x_at	-2.1		H.sapiens mRNA for IgG lambda light chain V-J-C region (clone Tgl11)	
217390_x_at		-1.4	Homo sapiens partial IGVH3 gene for immunoglobulin heavy chain V region, case 1, clone 16	
38241_at	1	3.2	butyrophilin	BTF3
212613_at	1.2	2.3	butyrophilin, subfamily 3, member A2	BTN3A2
209846_s_at	1	2	Similar to butyrophilin, subfamily 3, member A2	BTN3A2
204821_at	1.6		butyrophilin, subfamily 3, member A3	BTN3A3
208191_x_at	-1.2		pregnancy specific beta-1-glycoprotein 4	SPG4
206520_x_at	-1.3		sialic acid binding Ig-like lectin 6	SIGLEC6
210796_x_at	-1.5		CD33L2	SIGLEC6

Transcription

202485_s_at	-1.6		methyl-CpG binding domain protein 2, isoform 1	MBD2
203748_x_at		4.2	RNA binding motif, single stranded interacting protein 1 (RBMS1), transcript variant MSSP-2	RBMS1
207266_x_at		3.5	RNA binding motif, single stranded interacting protein 1 (RBMS1), transcript variant MSSP-3	RBMS1
208627_s_at		3.9	nuclease sensitive element binding protein 1	NSEP1
201996_s_at		2.1	KIAA0929 protein Msx2 interacting nuclear target (MINT) homolog	KIAA0929
202864_s_at		1.8	nuclear antigen Sp100	SP100
202487_s_at	1		purine-rich element binding protein B	PURB
205931_s_at		-1.2	Homo sapiens cAMP response element-binding protein CRE-BPa (H_GS165L15.1), mRNA.	H_GS165L15.1
214050_at		-1.5	CGG triplet repeat binding protein 1	CGGBP1
209362_at		1	SRB7 (suppressor of RNA polymerase B, yeast) homolog	SURB7
201085_s_at		1.2	SON DNA binding protein	SON

Basic Transcription Machinery

220113_x_at	-1.7		similar to DNA-directed RNA polymerase I (135 kDa)	FLJ10816
203664_s_at		1.5	polymerase (RNA) II (DNA directed) polypeptide D	POLR2D
206654_s_at	-1.9		polymerase (RNA) III (DNA directed) (32kD)	RPC32
209382_at		1.6	RNA polymerase III subunit	RPC62
209463_s_at	3		TFIID subunit p22	TAF2J
204772_s_at	1		transcription termination factor, RNA polymerase I	TTF1
214451_at	-1		transcription factor AP-2 beta (activating enhancer-binding protein 2 beta)	TFAP2B
222104_x_at	-1.5		general transcription factor IIH, polypeptide 3 (34kD subunit)	GTF2H3
210537_s_at		1.7	Similar to transcriptional adaptor 2 (ADA2, yeast, homolog)-like, clone MGC:1984	TADA2L
216941_s_at		2.9	TATA box binding protein (TBP)-associated factor, RNA polymerase I, B, 63kD	TAF1B
209430_at		-1	mRNA for TBP-associated factor 170	TAFII170
203542_s_at		1.3	basic transcription element binding protein 1	BTEB1
203543_s_at		1.8	basic transcription element binding protein 1	BTEB1
209212_s_at		1.8	mRNA for transcription factor BTEB2, complete cds	bteb2
208517_x_at		1.3	basic transcription factor 3	BTF3

Transcription Factors

206940_s_at	-1		POU domain, class 4, transcription factor 1	POU4F1
209947_at	-1.2		KIAA0144 gene product	KIAA0144
205861_at	-1		Spi-B transcription factor (Spi-1PU.1 related)	SPIB
221234_s_at	-1.1		BTB and CNC homology 1, basic leucine zipper transcription factor 2	BACH2
201416_at		-1.2	SRY (sex determining region Y)-box 4	SOX4
202936_s_at	-1.1		SRY (sex determining region Y)-box 9 (campomelic dysplasia, autosomal sex-reversal)	SOX9
202935_s_at	-1.9		SRY (sex determining region Y)-box 9 (campomelic dysplasia, autosomal sex-reversal)	SOX9
209842_at		-1.4	SRY (sex determining region Y)-box 10	SOX10
38918_at		-1.9	HMG box factor SOX-13	
210827_s_at	-1.2		epithelial-specific transcription factor ESE-1a	ESE-1
217053_x_at	-1.3		mRNA for ER81 transcription factor	ER81
211105_s_at	-1.3		transcription factor	NF-ATcC
211106_at	-1.4		transcription factor SUPT3H	SUPT3H
205365_at	-1.4		homeo box B6	HOXB6
220625_s_at	-1.5		Ets transcription factor ESE-2b mRNA	ELF5
205529_s_at	-1.8		core-binding factor, runt domain, alpha subunit2; translocated to, 1; cyclin D-related	CBFA2T1
210365_at	-1.8	-1.5	AML1 mRNA for AML1a protein (alternatively spliced product)	AML1
209360_s_at	-2.3	-1.4	AML1 mRNA for AML1b protein (alternatively spliced product)	AML1
204198_s_at		-1.7	runt-related transcription factor 3	RUNX3
204197_s_at	-1.6	-1.1	runt-related transcription factor 3	RUNX3
210479_s_at		2.5	transcription factor RZR-alpha mRNA	RORA

211466_at	-1.2	-1.1	nuclear factor I B3	NFIB	
213298_at		1.8	mRNA for CAAT-box binding transcription factor CTF-1 (syn. CTFNFI or CTF or NF-I or NF-1)	NFIC	
218724_s_at		1.7	TGF(beta)-induced transcription factor 2	TGIF2	
214499_s_at		1.7	Bcl-2-associated transcription factor short form mRNA	KIAA0164	
212420_at		1.6	E74-like factor 1 (ets domain transcription factor)	ELF1	
220625_s_at		-2.1	Ets transcription factor ESE-2b mRNA,	ELF5	
208961_s_at	1.3		mRNA for DNA-binding zinc finger(GBF)	COPEB	
218286_s_at		1.1	ring finger protein 7	RNF7	
201779_s_at		1.2	clone 24450 RING zinc finger protein RZF mRNA	RNF13	
207236_at		1.1	zinc finger protein 10 (KOX 1)	ZNF10	
218006_s_at		3.2	zinc finger protein 22 (KOX 15)	ZNF22	
212684_at		2.2	zinc finger protein 38 (KOX 25)	ZNF38	
203603_s_at	-2.2	-1.6	zinc finger homeobox 1B	ZFHX1B	
214741_at	1.2	3.4	zinc finger protein 131 (clone pHZ-10)	ZNF131	
207394_at		-1.3	zinc finger protein 137 (clone pHZ-30)	ZNF137	
206683_at		1.4	zinc finger protein 165	ZNF165	
206261_at	-1.3		zinc finger protein 239	ZNF239	
218645_at		1.7	zinc finger protein 277	ZNF277	
211393_at	-1.4		krueppel-related zinc finger protein SBZF5 mRNA	ZNF278	
205383_s_at	-1.1	-1.5	zinc finger protein 288	ZNF288	
200867_at	1		zinc finger protein 313	ZNF313	
218735_s_at		-1.2	zinc finger protein	AF020591	
206314_at		-1.2	zinc finger protein (ZFP)	ZFP	
209494_s_at	-1		zinc finger protein 278	ZFP278	
219104_at	1.1		C3HC4-like zinc finger protein	ZFP26	
220653_at	-1		zinc finger, imprinted 2	ZIM2	
206511_s_at	-2.4		sine oculis homeobox (Drosophila) homolog 2	SIX2	
206140_at	-5	-4.7	LIM homeobox protein 2	LHX2	
216973_s_at	-1		homeo box B7		
209706_at	-1.1		homeobox protein NKX3.1	NKX3A	
205991_s_at	-1.4		paired mesoderm homeo box 1, transcript variant pmx-1a	PMX1	
204689_at	-1.7		hematopoietically expressed homeobox	HHEX	
207147_at		-1.8	distal-less homeo box 2	DLX2	
208216_at		-1.2	distal-less homeobox 4	DLX4	
210259_s_at	-1.7		beta protein 1 BP1	DLX4	
210162_s_at	-1.1	-1.7	NF-ATc mRNA	NFATC1	
208003_s_at	-2.2		nuclear factor of activated T-cells 5, tonicity-resonsive	NFAT5	
215092_s_at		3.1	nuclear factor of activated T-cells 5, tonicity-resonsive	NFAT5	
213260_at	-1	-1.7	forkhead box C1	FOXC1	
206377_at	-1.3	-1.2	forkhead box F2	FOXF2	
202580_x_at	1.2		forkhead box M1	FOXM1	
209583_s_at	-2.9	-1.5	brain my033 protein mRNA	MOX2	I
202645_s_at	-1.5		multiple endocrine neoplasia I	MEN1	
212007_at	1	2.4	UBX domain-containing 1	UBXDC1	
212008_at		1.1	UBX domain-containing 1	UBXDC1	
203176_s_at		1.6	transcription factor 6-like 1 (mitochondrial transcription factor 1-like)	TCF6L1	
218502_s_at	-2	-1.6	trichorhinophalangeal syndrome I gene	TRPS1	
200878_at	1.2		endothelial PAS domain protein 1	EPAS1	
204651_at		-1	nuclear respiratory factor 1	NRF1	
201694_s_at		-1.3	early growth response 1	EGR1	
214077_x_at		-1.6	Meis (mouse) homolog 3	MEIS3	
206968_s_at	-1		nuclear factor related to kappa B bindingprotein	NFRKB	
215511_at	-1.1		AR1	TCF20	
206283_s_at	-1.1		T-cell acute lymphocytic leukemia 1	TAL1	
202046_s_at	-1.2		glucocorticoid receptor DNA binding factor 1	GRLF1	
219850_s_at		2.2	Homo sapiens Ets homologous factor (EHF), mRNA.	EHF	
221950_at		-1.2	empty spiracles (Drosophila) homolog 2	EMX2	
216509_x_at	-1.4		type V AF10 protein	AF10	
220684_at	-1.5		T-box 21	TBX21	
216364_s_at		-2.6	FMR2 protein	fmr2	
220559_at		-2.4	engrailed homolog 1	EN1	
209431_s_at		-1.3	zinc finger sarcoma gene long A isoform	ZSG	
209928_s_at		-1	activated B-cell factor-1	ABF-1	
203517_at		1	metaxin 2	MTX2	
203346_s_at	1		PHD finger DNA binding protein isoform 1	M96	
200959_at	1.5		fusion, derived from t(12;16) malignantliposarcoma	FUS	
217427_s_at	1.6		TUP1 like enhancer of SPLIT gene 1	TUPLE1	
212916_at		-1.1	KIAA1111 protein	KIAA1111	
203554_x_at	1.1	1	pituitary tumor-transforming 1	PTTG1	
210426_x_at		2.4	orphan hormone nuclear receptor RORalpha1	RORA	

Co-factor

202132_at		1.9	transcriptional co-activator with PDZ-binding motif	TAZ
217120_s_at	-1.9		cofactor required for Sp1 transcriptional activation, subunit 2 (150kD)	CRSP2
201504_s_at	1.6		translin	TSN
202817_s_at	1.5		synovial sarcoma, translocated to X chromosome	SSXT
203204_s_at	-1.7		KIAA0677 gene product	KIAA0677
218574_s_at	-1.3	-2.7	LIM and cysteine-rich domains 1	LMCD1
209059_s_at		1.6	Homo sapiens mRNA for hMBF1alpha, complete cds.	EDF1
209129_at		1.4	Human zyxin related protein ZRP-1 mRNA, complete cds.	TRIP6
221496_s_at		1.2	tob family	TOB2
214688_at		-1.4	transducin-like enhancer of split 4, homolog of Drosophila E(sp1)	TLE4
204872_at		-1	BCE-1 protein	BCE-1
201862_s_at		1.5	leucine rich repeat (in FLII) interactingprotein 1	LRRFIP1
214397_at	-1		methyl-CpG binding domain protein 2	MBD2

RNA Splicing

209381_x_at		-1.9	Homo sapiens, clone MGC:3975, mRNA, complete cds.	SF3A2
214305_s_at		2	splicing factor 3b, subunit 1, 155kD	SF3B1
201070_x_at		1.6	splicing factor 3b, subunit 1, 155kD	SF3B1
201741_x_at	-1.4		Human SF2p33 mRNA, complete cds.	SFRS1
202899_s_at		1.7	splicing factor, arginineserine-rich 3	SFRS3
212266_s_at		1.2	splicing factor, arginineserine-rich 5	SFRS5
213649_at	1	1.6	splicing factor, arginineserine-rich 7 (35kD)	SFRS7
214141_x_at		1.2	splicing factor, arginineserine-rich 7 (35kD)	SFRS7
202773_s_at		1.2	splicing factor, arginineserine-rich 8 (suppressor-of-white-apricot, Drosophila homolog)	SFRS8
201698_s_at		2.3	splicing factor, arginineserine-rich 9	SFRS9
210180_s_at		1	Homo sapiens htra2-beta-2 mRNA, complete cds.	SFRS10
207435_s_at		-1	Homo sapiens RNA binding protein; AT-rich element binding factor (SRM300), mRNA. / splicing coactivator subunit SRm300	SRM300
208910_s_at		4.1	Homo sapiens pre-mRNA splicing factor 2 p32 subunit (SF2p32) mRNA, complete cds	C1QBP
209161_at		2.4	PRP4STKWD splicing factor	HPRP4P
213898_at	-1.6		RNA binding motif protein 9	RBM9
201152_s_at		1.4	muscleblind (Drosophila)-like	MBNL
203103_s_at	1.1	1.6	nuclear matrix protein NMP200 related tosplicing factor PRP19	NMP200
37462_i_at		-2	Human spliceosomal protein (SAP 62) gene, complete cds	
203597_s_at	1.2		WW domain binding protein 4 (formin binding protein 21)	WBP4
205961_s_at		1.1	PC4 and SFRS1 interacting protein 2	PSIP2
210178_x_at		1.3	TLS-associated protein TASR	TASR1
210093_s_at		1.4	Mago homolog	MAGOH

Translation

214395_x_at		-1.3	eukaryotic translation elongation factor 1 delta (guanine nucleotide exchange protein)	EEF1D
200689_x_at		2.6	eukaryotic translation elongation factor 1 gamma	EEF1G
204102_s_at		2.1	translation elongation factor 2	EEF2
201144_s_at	1		eukaryotic translation initiation factor 2, subunit 1 (alpha, 35kD)	EIF2S1
200005_at		1.1	eukaryotic translation initiation factor 3, subunit 7 (zeta, 6667kD)	EIF3S7
210949_s_at		1.4	Similar to eukaryotic translation initiation factor 3, subunit 8 (110kD)	EIF3S8
200596_s_at		1.7	eukaryotic translation initiation factor 3, subunit 10	EIF3S10
201530_x_at		1	eukaryotic translation initiation factor 4A, isoform 1	EIF4A1
209393_s_at		2.6	cap-binding protein 4EHP mRNA	EIF4EL3
206621_s_at		1.2	Williams-Beuren syndrome chromosome region 1 / eukaryotic translation initiation factor 4H	WBSCR1
208705_s_at	1.5		eukaryotic translation initiation factor 5	EIF5
201027_s_at	1.3		translation initiation factor IF2	IF2
212566_at		-1.5	putative translation initiation factor	SUI1
218696_at		3	eukaryotic translation initiation factor 2-alpha kinase 3	EIF2AK3
213899_at		2.3	methionine aminopeptidase; eIF-2-associated p67	MNPEP
209861_s_at	1.2	2.2	eIF-2-associated p67 homolog	MNPEP
208319_s_at		1.5	RNA binding motif protein 3	RBM3
209024_s_at	1.2	1	NS1-associated protein 1	NSAP1
202157_s_at	-1	-1.2	RNA-binding protein BRUNOL3	BRUNOL3
208628_s_at		1.8	Homo sapiens, Similar to Y box protein 1, clone MGC:8655, mRNA, complete cds.	NSEP1
202156_s_at	-1.4		CUG triplet repeat, RNA-binding protein 2	CUGBP2
213517_at	-1		poly(rC)-binding protein 2	PCBP2

Protein Degredation

220422_at	-2		ubiquilin 3	UBQLN3

Proteasome

201532_at		1.8	proteasome (prosome, macropain) subunit, alpha type, 3	PSMA3
216088_s_at		5.2	proteasome (prosome, macropain) subunit, alpha type, 7	PSMA7
200876_s_at		3.1	proteasome (prosome, macropain) subunit, beta type, 1	PSMB1
202243_s_at		2.4	proteasome (prosome, macropain) subunit, beta type, 4	PSMB4
204279_at	1.4		proteasome (prosome, macropain) subunit, beta type, 9 (large multifunctional protease 2)	PSMB9
201067_at	1		proteasome (prosome, macropain) 26S subunit, ATPase, 2	PSMC2
201068_s_at	1	1	proteasome (prosome, macropain) 26S subunit, ATPase, 2	PSMC2
209503_s_at	1.5		proteasome (prosome, macropain) 26S subunit, ATPase, 5	PSMC5
201198_s_at	1.2		proteasome (prosome, macropain) 26S subunit, non-ATPase, 1	PSMD1
200814_at		1	proteasome (prosome, macropain) activator subunit 1 (PA28 alpha)	PSME1
209040_s_at	1.5	3.4	proteasome subunit LMP7 (allele LMP7B)	LMP7
210759_s_at	1.2	1.1	prosomal protein P30-33K	pros-30

ubiquitination

204022_at		-1.3	Nedd-4-like ubiquitin-protein ligase	WWP2
201001_s_at	1.2		ubiquitin-conjugating enzyme E2 variant 1	UBE2V1
200682_s_at		2.9	ubiquitin-conjugating enzyme E2L 3	UBE2L3
213822_s_at		-1.4	ubiquitin protein ligase	UBE3B
208761_s_at		2.5	ubiquitin-related protein SUMO-1 mRNA	UBL1
202412_s_at		2.7	ubiquitin specific protease 1	USP1
206405_x_at	-1.1		ubiquitin specific protease 6 (Tre-2 oncogene)	USP6
209136_s_at		1.6	ubiquitin specific protease 10	USP10
219211_at		-2	ubiquitin specific protease 18	USP18
214674_at		-1.2	ubiquitin specific protease 19	USP19
207614_s_at	1.2		cullin 1	CUL1
201840_at		1.6	neural precursor cell expressed, developmentallydown-regulated 8	NEDD8
203637_s_at		-1	midline 1	MID1
201846_s_at		1.1	RING1 and YY1 binding protein	RYBP
209743_s_at		1.1	atrophin-1 interacting protein 4	AIP4
203078_at		-1.5	CUL-2	CUL-2

F-Box Proteins

212218_s_at		-1.1	F-box only protein 9	FBXO9

Kinases

208079_s_at	2		serinethreonine kinase 6	STK6
208095_s_at	1.8	1.2	calciumcalmodulin-dependent protein kinase (CaM kinase) II gamma	CAMK2G
219148_at	1.7	1.1	PDZ-binding kinase; T-cell originated protein kinase	TOPK
209799_at	1.4		AMP-activated kinase alpha 1 subunit	PRKAA1
202801_at		-1	protein kinase, cAMP-dependent, catalytic, alpha	PRKACA
201805_at		2.2	protein kinase, AMP-activated, gamma 1 non-catalytic subunit	PRKAG1
200605_s_at		1.5	protein kinase, cAMP-dependent, regulatory, type I, alpha (tissue specific extinguisher 1)	PRKAR1A
209685_s_at	-1		protein kinase C beta-II type	PRKCB1
218236_s_at	-1		protein kinase C, nu	PRKCN
211993_at		1.7	protein kinase, lysine deficient 1	PRKWNK1
206562_s_at	1.2	1.5	casein kinase 1, alpha 1	CSNK1A1
211623_s_at		1.2	casein kinase II beta subunit	CSNK2B
204822_at	1.1	1.1	TTK protein kinase	TTK
207540_s_at	2.4		spleen tyrosine kinase	SYK
217356_s_at	1.1		phosphoglycerate kinase {alternatively spliced}	
203856_at	1		vaccinia related kinase 1	VRK1
204060_s_at	1		protein kinase, X-linked	PRKX
215909_x_at		-1	MisshapenNIK-related kinase	MINK
215916_at	-1		MisshapenNIK-related kinase	MINK
204813_at	-1.2		mitogen-activated protein kinase 10	MAPK10
211536_x_at		-5.1	mRNA for TGF-beta activated kinase 1c	MAP3K7
203652_at		-1	mitogen-activated protein kinase kinase kinase 11	MAP3K11
213263_s_at		1.8	mitogen-activated protein kinase kinase kinase 12	MAP3K12
205192_at		-1	mitogen-activated protein kinase kinase kinase 14	MAP3K14
211504_x_at	-1.2		mRNA for Rho kinase	ROCK2
206028_s_at	-1.5		c-mer proto-oncogene tyrosine kinase	MERTK
200884_at	-1.5		creatine kinase, brain	CKB
200644_at	-1.8	-1	macrophage myristoylated alanine-rich C kinase substrate	MACMARCKS
200075_s_at		1.3	guanylate kinase 1	
204635_at		1.2	ribosomal protein S6 kinase, 90kD, polypeptide 5	RPS6KA5
209043_at		1.2	bifunctional ATP sulfurylaseadenosine 5-phosphosulfate kinase	PAPSS1
202971_s_at		1.1	dual-specificity tyrosine-(Y)-phosphorylationregulated kinase 2 isoform 2	DYRK2
213328_at		1	NIMA (never in mitosis gene a)-related kinase 1	NEK1
209438_at		-1.1	phosphorylase kinase, alpha 2 (liver)	PHKA2
207821_s_at		-1.3	PTK2 protein tyrosine kinase 2	PTK2
207106_s_at		-1.4	leukocyte tyrosine kinase	LTK
219028_at		-1.5	homeodomain-interacting protein kinase 2	HIPK2
202686_s_at		-1.6	AXL receptor tyrosine kinase isoform 1precursor	AXL
202118_s_at		2.3	copine III	CPNE3
216680_s_at	-1.2		Homo sapiens, Similar to EphB4, clone IMAGE:3611312, mRNA, partial cds.	EPHB4

Phosphatases

201538_s_at	-1.3		dual specificity phosphatase 3 (vaccinia virus phosphatase VH1-related)	DUSP3
204014_at		-1.7	dual specificity phosphatase 4	DUSP4
206374_at	-1		dual specificity phosphatase 8	DUSP8
202703_at	1.3		dual specificity phosphatase 11 (RNARNP complex 1-interacting)	DUSP11
213305_s_at	1	3.8	protein phosphatase 2A B56-gamma1	PP2A
210946_at	-1	-1.3	type-2 phosphatidic acid phosphatase alpha-2	PAP2-a2
214693_x_at		4.4	phosphatidic acid phosphatase type 2A	PPAP2A
212226_s_at		-1.2	phosphatidic acid phosphatase type 2B	PPAP2B
212230_at	-1.6	-1.3	phosphatidic acid phosphatase type 2B	PPAP2B
218273_s_at	-1.1		pyruvate dehydrogenase phosphatase	PDP
216988_s_at		1.4	protein tyrosine phosphatase type IVA, member 2	PTP4A2
208615_s_at		1.6	protein tyrosine phosphatase type IVA, member 2	PTP4A2
209695_at	-1.1		Similar to protein tyrosine phosphatase type IVA, member 3	PTP4A3
206344_at	-1.5		serum aryldialkylphosphatase precursor mRNA	PON1
206687_s_at	-1.7		protein tyrosine phosphatase, non-receptor type 6	PTPN6
213465_s_at		1.1	protein phosphatase 1, regulatory subunit 7	PPP1R7
201214_s_at		1.4	protein phosphatase 1, regulatory subunit 7	PPP1R7
201702_s_at		2	protein phosphatase 1, regulatory subunit 10	PPP1R10
205194_at		-1.1	phosphoserine phosphatase	PSPH
211600_at	-2.3	-1.5	glomerular epithelial protein 1	GLEPP1

Other Signaling / Scaffolding related / accessory / adaptor

215280_s_at		-1.1	protein tyrosine phosphatase, receptor type, f polypeptide (PTPRF), interacting protein (liprin), alpha 3	PPFIA3
213368_x_at		-1	protein tyrosine phosphatase, receptor type, f polypeptide (PTPRF), interacting protein (liprin), alpha 3	PPFIA3
205359_at	-1.5		A kinase (PRKA) anchor protein 6	AKAP6
211172_x_at		-1.5	A-kinase anchoring protein 18 beta	AKAP7
212334_at		-1	Homo sapiens AKAP350C mRNA sequence, alternatively spliced	
215336_at	-2		A kinase (PRKA) anchor protein 11	AKAP11
210057_at		1.8	lambdaiota protein kinase C-interacting protein mRNA	KIAA0421
204746_s_at		-1	protein kinase C, alpha binding protein	PRKCABP
202732_at		-1.3	protein kinase (cAMP-dependent, catalytic) inhibitor gamma	PKIG
205008_s_at		-1.5	DNA-dependent protein kinase catalytic subunit-interacting protein 2	KIP2
219024_at		2.6	pleckstrin homology domain-containing, family A (phosphoinositide binding specific) member 1	PLEKHA1
201811_x_at	-1		SH3-domain binding protein 5 (BTK-associated)	SH3BP5
206144_at	-1.1	-1	BAI1-associated protein 1	BAIAP1
202746_at	-1.2		integral membrane protein 2A	ITM2A

205367_at	-1.8		Homo sapiens adaptor protein with pleckstrin homology and src homology 2 domains (APS), mRNA.	APS
213307_at	-1.8	-1.6	cortactin SH3 domain-binding protein	KIAA1022
215418_at	-1.8		alpha-parvin	PARVA
200787_s_at	-1.8		phosphoprotein enriched in astrocytes 15	PEA15
207706_at	-3.3		Usher syndrome type IIa protein	USH2A
208670_s_at		1.4	Homo sapiens PNAS-26 mRNA, complete cds.	CRI1
201811_x_at		-1.2	Homo sapiens SH3-domain binding protein 5 (BTK-associated) (SH3BP5), mRNA.	SH3BP5
34031_i_at		1.6	Human Krit1 mRNA, complete cds	
203300_x_at	-1.3		adaptor-related protein complex 1, sigma 2 subunit	AP1S2
200613_at		1.3	adaptor-related protein complex 2, mu 1 subunit	AP2M1
208710_s_at		1	adaptor-related protein complex 3, delta 1 subunit	AP3D1
204400_at		-1	Homo sapiens signal transduction protein (SH3 containing) (EFS2), mRNA.	EFS2
219132_at	-1.3		pellino (Drosophila) homolog 2	PELI2
202342_s_at	-1.3		tripartite motif protein TRIM2	KIAA0517
210579_s_at	-1.3		tripartite motif protein TRIM10 alpha	TRIM10
203735_x_at		-1.1	PTPRF interacting protein, binding protein 1 (liprin beta 1)	PPFIBP1
221489_s_at	-1.2		sprouty (Drosophila) homolog 4	SPRY4
217465_at	-1.3		NCK-associated protein 1	NCKAP1
205741_s_at	-1.3		dystrobrevin, alpha	DTNA
214375_at	-1.4		PTPRF interacting protein, binding protein 1 (liprin beta 1)	PPFIBP1
203299_s_at	-1.4		Homo sapiens DC22 mRNA, complete cds.	AP1S2
202474_s_at	-1.4		host cell factor C1 (VP16-accessory protein)	HCFC1
220405_at	-1.9		syntrophin, gamma 1	SNTG1
203593_at		1.1	CD2-associated protein	CD2AP
204362_at		1.1	SKAP55 homologue	SKAP-HOM

G proteins and related Genes

207791_s_at	1.3		RAB1, member RAS oncogene family	RAB1
208734_x_at		2.6	GTP-binding protein	RAB2
208730_x_at		-1	RAB2, member RAS oncogene family	RAB2
219151_s_at		2.8	RAB, member of RAS oncogene family-like 2B	RABL2B
218360_at		1.5	RAB22A, member RAS oncogene family	RAB22A
217764_s_at	1		small GTP-binding protein rab22b mRNA	RAB31
202483_s_at		3.4	RAN binding protein 1	RANBP1
200993_at	1	1.2	RAN binding protein 7	RANBP7
214449_s_at	-1.2		ras-like protein	TC10
205590_at	-1.3	-2.6	RAS guanyl releasing protein 1 (calcium and DAG-regulated)	RASGRP1
214352_s_at		1.9	v-Ki-ras2 Kirsten rat sarcoma 2 viral oncogene homolog	KRAS2
207629_s_at	-1.1		rhorac guanine nucleotide exchange factor (GEF) 2	ARHGEF2
203264_s_at		-2.1	Cdc42 guanine exchange factor (GEF) 9	ARHGEF9
212724_at		1.2	ras homolog gene family, member E	ARHE
201659_s_at		1.1	ADP-ribosylation factor-like 1	ARL1
202641_at		1	ADP-ribosylation factor-like 3	ARL3
202206_at		1.6	ADP-ribosylation factor-like 7	ARL7
201453_x_at		1.2	Ras homolog enriched in brain 2	RHEB2
204680_s_at		1.1	guanine nucleotide exchange factor for Rap1; M-Ras-regulated GEF	KIAA0277
213467_at		-1.1	GTP-binding protein Rho7	RHO7
200651_at		1.2	guanine nucleotide binding protein (G protein), beta polypeptide 2-like 1	GNB2L1
204115_at		-1.1	guanine nucleotide binding protein 11	GNG11
564_at	-1		guanine nucleotide-binding regulatory protein (G-y-alpha)	HUMGTPBRPA
40562_at		-1	guanine nucleotide-binding regulatory protein (G-y-alpha)	
206608_s_at	-1.2		retinitis pigmentosa GTPase regulator interacting protein 1	RPGRIP1
206323_x_at	-1.2	-1.2	oligophrenin 1, Rho-GTPase acivating protein	OPHN1
213446_s_at	-1.3		IQ motif containing GTPase activating protein 1	IQGAP1
222077_s_at	1.1		GTPase activating protein	ID-GAP
200750_s_at		3.5	GTP binding protein mRNA	RAN
212977_at		1.1	G protein-coupled receptor	RDC1
214104_at	-1.5		G-protein coupled receptor	RE2
213880_at	-1.6	-2	G protein-coupled receptor 49	GPR49
212070_at	-1.2		G protein-coupled receptor 56	GPR56
221394_at	-1.6		G protein-coupled receptor 58	GPR58
206002_at	-2.1	-2.3	G protein-coupled receptor 64	GPR64
219936_s_at		1.3	G protein-coupled receptor 87	GPR87
200008_s_at	1.1		human rab GDI	GDI2
209180_at	1		geranylgeranyl transferase type II beta-subunit	RABGGTB
217835_x_at		1	putative Rab5-interacting protein	LOC55969
209637_s_at	-1.1		regulator of G-protein signalling 12	RGS12
204336_s_at	-1		G protein signalling regulator 19	RGS19
204882_at	1.8		KIAA0053 gene product	KIAA0053
210138_at		3.1	regulator of G protein signaling-Z	RGSZ1
216520_s_at		2.6	tumor protein, translationally-controlled 1	TPT1
200734_s_at		-1.5	ADP-ribosylation factor 3	ARF3
204732_s_at	-2		ADP-ribosylation factor domain protein 1, 64kD	ARFD1
203910_at		1.2	Homo sapiens PTPL1-associated RhoGAP 1	PARG1
207369_at	-1.4	-1.8	bombesin-like receptor 3	BRS3
204819_at	-1.1		faciogenital dysplasia protein	FGD1
221466_at	-1.3		pyrimidinergic receptor P2Y, G-protein coupled,4	P2RY4
203817_at		-1.5	guanylate cyclase 1, soluble, beta 3	GUCY1B3
218625_at	-1.4		neuritin	LOC51299
206455_s_at		-2.4	Homo sapiens rhodopsin (opsin 2, rod pigment) (retinitis pigmentosa 4, autosomal dominant) (RHO), mRNA.	RHO
221524_s_at	1.1	1.2	Rag D	RAGD
211503_s_at		3.2	ras-related protein rab-14	Rab14

Chaperones & Heath Shock

200806_s_at	1.8	1.7	heat shock 60kD protein 1 (chaperonin)	HSPD1

200800_s_at		2.4	heat shock 70kD protein 1A	HSPA1A
201841_s_at		3	heat shock 27kD protein 1	HSPB1
211968_s_at	1.6	2.2	heat shock 90kD protein 1, alpha	HSPCA
211969_at	1.5		heat shock 90kD protein 1, alpha	HSPCA
221742_at	1.5		DnaJ (Hsp40) homolog, subfamily C, member 3	DNAJC3
210211_s_at		1.4	Hsp89-alpha-delta-N	HSPCA
214359_s_at	1.3		heat shock 90kD protein 1, beta	HSPCB
200692_s_at	1.3	2.7	heat shock 70kD protein 9B (mortalin-2)	HSPA9B
210338_s_at	1.3		HSC54 mRNA for heat shock cognate protein 54	HSC54
219212_at	1.1	1.1	heat shock protein hsp70-related protein	LOC51182
211015_s_at		1.4	heat shock protein 70 (hsp70) mRNA	hsp70
201946_s_at	1.2	2.6	chaperonin containing TCP1, subunit 2 (beta)	CCT2
208696_at		2.3	PNAS-102 mRNA	CCT5
209867_s_at	-1.7		lectomedin-3	LEC3
213919_at	-1.2		DnaJ (Hsp40) homolog, subfamily C, member 4	DNAJC4
			**Metabolism and Homeostasis**	
211478_s_at	2.5		dipeptidyl peptidase IV	CD26
216202_s_at	2.5		palmitoyltransferase	LCB2
208993_s_at	1.7		peptidyl-prolyl isomerase G (cyclophilin G)	PPIG
202179_at	1.5		bleomycin hydrolase	BLMH
201477_s_at	1		ribonucleotide reductase M1 polypeptide	RRM1
209773_s_at	1.4	2.3	ribonucleotide reductase M2 polypeptide	RRM2
201890_at	1.1		ribonucleotide reductase M2 polypeptide	RRM2
205949_at	-2.6		carbonic anhydrase I	CAI
209301_at	1.2		carbonic anhydrase II	CA2
205199_at	-1		carbonic anhydrase IX	CA9
203963_at		1.5	carbonic anhydrase XII precursor	CA12
219759_at	1.2		aminopeptidase	LOC64167
213653_at	1.2		putative methyltransferase	M6A
218111_s_at	1.1		CMP-N-acetylneuraminic acid synthase	LOC55907
210544_s_at	1.1		aldehyde dehydrogenase 10 (fatty aldehyde dehydrogenase)	ALDH3A2
211004_s_at	-1.3		Similar to aldehyde dehydrogenase 7	ALDH3B1
221590_s_at	-1.3		Homo sapiens clone FLB9440 PRO2550 mRNA, complete cds	ALDH6A1
208911_s_at	1.1		pyruvate dehydrogenase (lipoamide) beta	PDHB
203711_s_at	1.1		3-hydroxyisobutyryl-Coenzyme A hydrolase	HIBCH
204767_s_at	1.1		flap structure-specific endonuclease 1	FEN1
215125_s_at	1		UDP glycosyltransferase 1 family, polypeptide A9	UGT1A9
209580_s_at	1		methyl-CpG binding endonuclease	MED1
202589_at	1		thymidylate synthetase	TYMS
204646_at	-1		dihydropyrimidine dehydrogenase	DPYD
214088_s_at	-1		fucosyltransferase 3 (galactoside 3(4)-L-fucosyltransferase, Lewis blood group included)	FUT3
211548_s_at	-1		NAD+-dependent 15-hydroxyprostaglandin dehydrogenase	PGDH
221304_at	-1		UDP glycosyltransferase 1 family, polypeptide A8	UGT1A8
216203_at	-1		palmitoyltransferase	LCB2
203895_at	-1		phospholipase C, beta 4	PLCB4
205111_s_at		-1.7	pancreas-enriched phospholipase C	LOC51196
209392_at	-2	-1.7	Human autotaxin mRNA	ENPP2
221240_s_at	-1		beta-1,3-N-acetylglucosaminyltransferase bGn-T4	B3GN-T4
219773_at	-1		NADPH oxidase 4	NOX4
220929_at	-1		UDP-N-acetyl-alpha-D-galactosamine:polypeptide N-acetylgalactosaminyltransferase 8 (GalNAc-T8)	GALNT8
218313_s_at	-1.1		UDP-N-acetyl-alpha-D-galactosamine:polypeptide N-acetylgalactosaminyltransferase 7 (GalNAc-T7)	GALNT7
211673_s_at	-1		molybdenum cofactor biosynthesis protein A and molybdenum cofactor biosynthesis protein C mRNA	
205752_s_at	-1	-1.2	glutathione S-transferase M5	GSTM5
203914_x_at	-1.1		hydroxyprostaglandin dehydrogenase 15-(NAD)	HPGD
208591_s_at	-1.1		phosphodiesterase 3B, cGMP-inhibited	PDE3B
214091_s_at	-1.1		glutathione peroxidase 3 (plasma)	GPX3
206212_at	-1.1		carboxypeptidase A2 (pancreatic)	CPA2
202422_s_at	-1.1		fatty-acid-Coenzyme A ligase, long-chain 4	FACL4
221062_at	-1.2		heparan sulfate (glucosamine) 3-O-sulfotransferase 3B1	HS3ST3B1
221416_at	-1.2		phospholipase A2, group IIF	PLA2G2F
215095_at	-1.3		esterase Dformylglutathione hydrolase	ESD
209009_at		1.1	Similar to esterase 10	ESD
211387_x_at	-1.3		mRNA capping enzyme	hCAP1b
205333_s_at	-1.3		prenyl protein protease RCE1B	RCE1B
220446_s_at	-1.3		carbohydrate (N-acetylglucosamine 6-O) sulfotransferase 4	CHST4
206792_x_at	-1.3		phosphodiesterase 4C, cAMP-specific (dunce (Drosophila)-homolog phosphodiesterase E1)	PDE4C
203549_s_at	-1.3		lipoprotein lipase	LPL
204154_at	-1.4	-1.1	cysteine dioxygenase, type I	CDO1
221764_at		1.3	glycerol-3-phosphate dehydrogenase 1 (soluble)	GPD1
204881_s_at		1.1	UDP-glucose ceramide glucosyltransferase	UGCG
205480_s_at		1.6	UDP-glucose pyrophosphorylase 2	UGP2
213882_at		3.2	glucose phosphate isomerase	GPI
213093_at	-1.4		glucose-6-phosphate dehydrogenase	G6PD
220979_s_at	-1.5		similar to sialyltransferase 7 ((alpha-N-acetylneuraminyl 2,3-betagalactosyl-1,3)-N-acetyl galactosaminide alpha-2,6-sialyltransferase) E	MGC3184
213936_x_at	-1.5		N-acylsphingosine amidohydrolase (acid ceramidase)-like	ASAHL
205351_at	-1.5		gamma-glutamyl carboxylase	GGCX
215743_at	-1.5		ribonuclease P (38kD)	RPP38
207303_at	-1.5		phosphodiesterase 1C, calmodulin-dependent (70kD)	PDE1C
209919_x_at	-1.5		gamma-glutamyl transpeptidase	GGT1
211207_s_at	-1.5		long-chain acyl-CoA synthetase 5	LACS5
209420_s_at	-1.5		acid sphingomyelinase	ASM
205066_s_at	-1.6	-2.1	ectonucleotide pyrophosphatasephosphodiesterase 1	ENPP1

210839_s_at	-1.6		phosphodiesterase I alpha	ENPP2
218322_s_at	-1.6		long-chain fatty acid coenzyme A ligase 5	FACL5
214582_at	-1.6	-1.6	2,3-cyclic nucleotide 3 phosphodiesterase	CNP
204869_at	-1.6	-1.7	proprotein convertase subtilisinkexin type 2	PCSK2
201785_at	-1.7		ribonuclease, RNase A family, 1 (pancreatic)	RNASE1
205174_s_at	-1.8		glutaminyl-peptide cyclotransferase (glutaminyl cyclase)	QPCT
204836_at	-2	-1.9	glycine dehydrogenase (decarboxylating; glycine decarboxylase, glycine cleavage system protein P)	GLDC
200832_s_at	-2		Scd mRNA for stearoyl-CoA desaturase	Scd
205338_s_at	-2.2		dopachrome tautomerase (dopachrome delta-isomerase, tyrosine-related protein 2)	DCT
205337_at	-2.5	-1.2	dopachrome tautomerase (dopachrome delta-isomerase, tyrosine-related protein 2)	DCT
216512_s_at	-1.1		dopachrome tautomerase (dopachrome delta-isomerase, tyrosine-related protein 2)	DCT
207016_s_at	-2.2	-2	Homo sapiens mRNA for RALDH2-T, complete cds.	RALDH2
210147_at	-2.3	-2.6	mono-ADP-ribosyltransferase	htMART
206465_at	-2.3	-3.5	very long-chain acyl-CoA synthetase; lipidosin	KIAA0631
206214_at	-3.1		phospholipase A2, group VII (platelet-activating factor acetylhydrolase, plasma)	PLA2G7
211264_at	-5		glutamate decarboxylase	GAD65
201014_s_at	1.4	2	multifunctional polypeptide similar to SAICARsynthetase and AIR carboxylase	ADE2H1
200883_at	1.4	1	ubiquinol-cytochrome c reductase core protein II	UQCRC2
208909_at		1	ubiquinol-cytochrome c reductase, Rieske iron-sulfur polypeptide 1	UQCRFS1
209699_x_at	1		dihydrodiol dehydrogenase	AKR1C2
214421_x_at	-1		cytochrome P450, subfamily IIC (mephenytoin 4-hydroxylase), polypeptide 9	CYP2C9
216025_x_at	-1.1		cytochrome P450, subfamily IIC (mephenytoin 4-hydroxylase), polypeptide 9 / Human cytochrome P-450 S-mephenytoin 4-hydroxylase (P-450mp)	CYP2C
214610_at	-1.1	-1.3	cytochrome P450, subfamily XIB (steroid 11-beta-hydroxylase), polypeptide 1	CYP11B1
202437_s_at	-3.5	-2.7	cytochrome P450, subfamily I (dioxin-inducible), polypeptide 1 (glaucoma 3, primary infantile)	CYP1B1
202435_s_at	-2.3		cytochrome P450, subfamily I (dioxin-inducible), polypeptide 1 (glaucoma 3, primary infantile)	CYP1B1
202436_s_at	-2.2	-1.2	cytochrome P450, subfamily I (dioxin-inducible), polypeptide 1 (glaucoma 3, primary infantile)	CYP1B1
210816_s_at	-1.3		Similar to cytochrome b-561	CYB561
203923_s_at	-1.4		cytochrome b-245, beta polypeptide (chronic granulomatous disease)	CYBB
207608_x_at	-1.6		cytochrome P450, subfamily I (aromatic compound-inducible), polypeptide 2	CYP1A2
205998_x_at	-1.4		cytochrome P450, subfamily IIIA (niphedipine oxidase), polypeptide 4	CYP3A4
210576_at		-2.6	cytochrome P450	CYP4F8
219565_at		2.2	cytochrome P450 monooxygenase	LOC57404
216696_s_at	-1.7		kidney and liver proline oxidase 1	HSPOX1
201117_s_at	-1.9	-1.3	carboxypeptidase E precursor	CPE
200968_s_at		3.7	peptidylprolyl isomerase B (cyclophilin B)	PPIB
208629_s_at		3.1	hydroxyacyl-Coenzyme A dehydrogenase3-ketoacyl-Coenzyme A thiolaseenoyl-Coenzyme A hydratase (trifunctional protein), alpha subunit	HADHA
203128_at		2.9	serine palmitoyltransferase, long chain basesubunit 2	SPTLC2
212995_x_at		2.6	phosphoglycerate dehydrogenase	PHGDH
203803_at		2	prenylcysteine lyase	PCL1
217933_s_at		1.7	leucine aminopeptidase	LOC51056
203219_s_at		1.7	adenine phosphoribosyltransferase	APRT
207076_s_at		1.6	argininosuccinate synthetase	ASS
201923_at		1.3	thioredoxin peroxidase (antioxidant enzyme)	AOE372
202554_s_at		1.3	glutathione S-transferase M3 (brain)	GSTM3
203560_at		1.2	gamma-glutamyl hydrolase (conjugase,folylpolygammaglutamyl hydrolase) precursor	GGH
203228_at		1.2	platelet-activating factor acetylhydrolase,isoform Ib, gamma subunit (29kD)	PAFAH1B3
200848_at		1.2	S-adenosylhomocysteine hydrolase-like 1	AHCYL1
202619_s_at		1.2	procollagen-lysine, 2-oxoglutarate 5-dioxygenase (lysine hydroxylase) 2	PLOD2
202532_s_at		1.1	dihydrofolate reductase	DHFR
214045_at		1.1	lipoic acid synthetase	LAS
217995_at		1	CGI-44 protein; sulfide dehydrogenase like (yeast) (CGI-44)	CGI-44
219932_at		1	very long-chain acyl-CoA synthetase homolog 1	VLCS-H1
201030_x_at		1	lactate dehydrogenase B	LDHB
212360_at		-1	adenosine monophosphate deaminase 2 (isoform L)	AMPD2
203188_at		-1	i-beta-1,3-N-acetylglucosaminyltransferase	BETA3GNTI
213591_at		-1.1	aldehyde dehydrogenase 7 family, member A1	ALDH7A1
208951_at	1		Homo sapiens, antiquitin 1, clone MGC:1569, mRNA, complete cds.	ALDH7A1
220148_at		-1.3	aldehyde dehydrogenase 12	ALDH12
213736_at		-1.4	cytochrome c oxidase subunit Vb	COX5B
203551_s_at		-1.2	COX11 (yeast) homolog, cytochrome c oxidase assembly protein	COX11
204067_at		-1.3	sulfite oxidase	SUOX
222011_s_at		-1.3	acetyl-Coenzyme A acetyltransferase 2 (acetoacetyl Coenzyme A thiolase)	ACAT2
206720_at		-1.5	alpha-1,3(6)-mannosylglycoproteinbeta-1,6-N-acetyl-glucosaminyltransferase	MGAT5
213705_at		-1.5	methionine adenosyltransferase II, alpha	MAT2A
205083_at		-1.6	aldehyde oxidase 1	AOX1
206037_at		-1.8	cysteine conjugate-beta lyase; cytoplasmic (glutamine transaminase K, kyneurenine aminotransferase)	CCBL1
200762_at	-1.9	-1.9	dihydropyrimidinase-like 2	DPYSL2
201431_s_at	-1.4		dihydropyrimidinase-like 3	DPYSL3
202218_s_at		-1.9	delta-6 fatty acid desaturase	FADSD6
204294_at		-2	aminomethyltransferase (glycine cleavage systemprotein T)	AMT
201791_s_at		-2	7-dehydrocholesterol reductase	DHCR7
209613_s_at		-2	alcohol dehydrogenase 2 (class I), beta polypeptide	ADH2
204515_at		-2.3	hydroxy-delta-5-steroid dehydrogenase, 3 beta-and steroid delta-isomerase 1	HSD3B1
205404_at	-2.1	-2.3	hydroxysteroid (11-beta) dehydrogenase 1	HSD11B1
219429_at		-2.4	fatty acid hydroxylase	FAAH
205307_s_at		-2.4	kynurenine 3-monooxygenase (kynurenine 3-hydroxylase)	KMO
219799_s_at		-3	retinol dehydrogenase homolog	RDHL
218967_s_at		1.5	phosphotriesterase related	PTER
205370_x_at	-1.2		dihydrolipoamide branched chain transacylase (E2 component of branched chain keto acid dehydrogenasecomplex; maple syrup urine disease)	DBT
204572_s_at		-1.2	protein (peptidyl-prolyl cistrans isomerase)NIMA-interacting, 4 (parvulin)	PIN4
206643_at	-2.1		histidine ammonia-lyase	HAL

202499_s_at		-1	solute carrier family 2 (facilitated glucose transporter), member 3	SLC2A3
200978_at		1.8	malate dehydrogenase 1, NAD (soluble)	MDH1
217990_at		3.1	Homo sapiens GMPR2 for guanosine monophosphate reductase isolog (LOC51292), mRNA	LOC51292
211416_x_at		-1.1	gamma-glutamyl transpeptidase	GGT1
209218_at		1.1	squalene epoxidase	ERG1
208864_s_at	1	1	Homo sapiens thioredoxin mRNA, complete cds.	TXN
220452_x_at	-1.1		cytochrome c-like antigen	CYCL
210686_x_at	-1.1		cytochrome c-like antigen	
210830_s_at		1.7	paraoxonase	PON2
208940_at		1.2	SELENOPHOSPHATE SYNTHETASE ; Human selenium donor protein	SPS
218671_s_at	1.1		ATPase inhibitor precursor	LOC51189
220671_at	-1.3		CCR4-like (carbon catabolite repression 4, S.cerevisiae)	CCRN4L
204416_x_at		-1.9	apolipoprotein C-I	APOC1
212884_x_at	-1.1		apolipoprotein E	APOE
221013_s_at		-1.3	apolipoprotein L, 2	APOL2
201339_s_at		1	sterol carrier protein 2	SCP2
215695_s_at		-1.9	glycogenin-2 delta	glycogenin-2
210959_s_at		-1.4	steroid-5-alpha-reductase isoform	SRD5A1
218301_at		-1.3	arginyl aminopeptidase (aminopeptidase B)-like1	RNPEPL1
212221_x_at		-1.2	popeye protein 3	POP3
213060_s_at		-1.1	chitinase	HUMTCHIT
206227_at		-1.1	Homo sapiens cartilage intermediate layer protein, nucleotide pyrophosphohydrolase (CILP), mRNA.	CLIP
209154_at		-1	glutaminase-interacting protein 3	TIP-1
206958_s_at		1	UPF3	UPF3
218197_s_at		1	oxidation resistance 1	OXR1
204143_s_at		1.1	rTS beta protein	HSRTSBETA
218729_at		1.1	latexin protein	LXN
201455_s_at		1.3	puromycin sensitive aminopeptidase	NPEPPS
201454_s_at		1.4	aminopeptidase puromycin sensitive	NPEPPS
213026_at		1.4	Apg12 (autophagy 12, S. cerevisiae)-like	APG12L
213496_at	-1.9	-1.3	KIAA0455 gene product	KIAA0455

Melanogenesis

206426_at	-1	-1.5	melan-A	MLANA
206427_s_at	-1.8	-1.3	differentiation antigen melan-A protein	MLANA
207233_s_at	-1.8		microphthalmia-associated transcription factor	MITF
205694_at	-2.3	-1.5	tyrosinase-related protein 1	TYRP1
206630_at	-2.1		tyrosinase (oculocutaneous albinism IA)	TYR
209848_s_at	-1.7		me20m	SILV

Ion Homeostasis

203296_s_at	-1.2		ATPase, Na+K+ transporting, alpha 2 (+) polypeptide	ATP1A2
207546_at	-1.5		ATPase, H+K+ exchanging, beta polypeptide	ATP4B
205198_s_at		-1.3	ATPase, Cu++ transporting, alpha polypeptide (Menkes syndrome)	ATP7A
204624_at	-1.1		ATPase, Cu++ transporting, beta polypeptide (Wilson disease)	ATP7B
203908_at	-1.7		solute carrier family 4, sodium bicarbonate cotransporter, member 4	SLC4A4
209884_s_at	-1.5		sodium bicarbonate cotransporter 3	SLC4A7
204404_at	-1		solute carrier family 12 (sodiumpotassiumchloride transporters), member 2	SLC12A2
220740_s_at	-1.4		solute carrier family 12 (potassiumchloride transporters), member 6	SLC12A6
207308_at	-2.1		solute carrier family 21 (organic anion transporter), member 3	SLC21A3
220867_s_at	-1.2	-1	solute carrier family 24 (sodiumpotassiumcalcium exchanger), member 2	SLC24A2
220475_at	-1.5		concentrative Na+-nucleoside cotransporter	CNT3
207408_at	-1.5		organic cationic transporter-like 4	ORCTL4
210286_s_at	-2.1		bicarbonate transporter	BT
200748_s_at		1.7	ferritin, heavy polypeptide 1	FTH1
217897_at	-1.4	-1.6	FXYD domain-containing ion transport regulator 6	FXYD6
210078_s_at		-1.6	(clone hKvBeta3) K+ channel beta subunit	KCNAB1
221372_s_at		-1.8	purinergic receptor P2X, ligand-gated ionchannel, 2	P2RX2
220551_at	-1.7		differentiation-associated Na-dependent inorganic phosphate cotransporter	DNPI
206306_at	-1.6		ryanodine receptor 3	RYR3
209620_s_at	1.9		ABC transporter 7 protein	hABC7
203903_s_at	-1.2	-1.8	hephaestin	HEPH
217165_x_at		1.6	metallothionein 1F (functional)	MT1F
204745_x_at		1.1	metallothionein 1G	MT1G
211330_s_at	-1.2		Homo sapiens hemochromatosis splice variant delE3 mRNA, complete cds.	HFE
207568_at		-1.1	cholinergic receptor, nicotinic, alphapolypeptide 6	CHRNA6
210739_x_at	-1.3		sodium bicarbonate cotransporter	NBC

RNA Metabolism

204766_s_at	3.7	3.3	nudix (nucleoside diphosphate linked moietyX)-type motif 1	NUDT1
206302_s_at		-1.1	nudix (nucleoside diphosphate linked moietyX)-type motif 4	NUDT4
220183_s_at	2.4	3.7	nudix (nucleoside diphosphate linked moietyX)-type motif 6	NUDT6
218269_at		-1	putative ribonuclease III	RNASE3L
222035_s_at		1.4	poly(A) polymerase alpha	PAPOLA
215136_s_at	1		Opa-interacting protein 2	OIP2
203594_at	1		RNA 3-terminal phosphate cyclase	RPC

tRNA synthesis

200841_s_at		2.5	glutamyl-prolyl-tRNA synthetase	EPRS
201263_at		1	threonyl-tRNA synthetase	TARS
209252_at	1.2		histidyl-tRNA synthetase homolog	HO3

Intracellular Vesicular Traffic and Protein Sorting

201831_s_at	3.1	4.4	vesicle docking protein p115	P115
200900_s_at	2.8	1.4	mannose-6-phosphate receptor (cation dependent)	M6PR
214336_s_at	-1		coatomer protein complex, subunit alpha	COPA
201358_s_at		-1.2	coatomer protein complex, subunit beta	COPB
203106_s_at	-1		vacuolar protein sorting 41 (yeast homolog)	VPS41
204324_s_at	-1.8		type II Golgi membrane protein	GPP130
201273_s_at		1.6	signal recognition particle 9kD	SRP9
216296_at	-1.4		H.sapiens clathrin light chain a gene.	
209167_at	-2		glycoprotein M6B	GPM6B
209168_at	-1.5	-1.3	glycoprotein M6B	GPM6B
209169_at	-1.9		glycoprotein M6B	GPM6B
209170_s_at	-2.5	-1.7	glycoprotein M6B	m6b1
203484_at		1.6	Homo sapiens Sec61 gamma	SEC61G
202113_s_at		3.1	sorting nexin 2	SNX2
213206_at		-1.2	golgi SNAP receptor complex member 2	GOSR2
213212_x_at		-1.3	golgin-67	KIAA0855
212807_s_at	-1.4		sortilin 1	SORT1
204840_s_at		1	early endosome antigen 1, 162kD	EEA1
212417_at		1	secretory carrier membrane protein 1	SCAMP1
212425_at		-1.2	secretory carrier membrane protein 1	SCAMP1
218143_s_at	-1.1		secretory carrier membrane protein 2	SCAMP2
201176_s_at		2.5	archain	ARCN1
201278_at	-1.9		disabled (Drosophila) homolog 2 (mitogen-responsive phosphoprotein)	DAB2
209558_s_at		-1.9	HIP1R	HIP1R
210293_s_at		-1	Sec23 (S. cerevisiae) homolog B	SEC23B
207707_s_at		1	SEC13 (S. cerevisiae)-like 1	SEC13L1
221504_s_at		1.3	Vacuolar proton pump subunit SFD alpha isoform	LOC51606

Protein Secretion/ Secreted Proteins / Hormone related

206639_x_at	-1.7		histatin 1	HTN1
216341_s_at	-1.7	-1.4	gonadotropin-releasing hormone receptor	GNRHR
211753_s_at	-1.6		relaxin 1 (H1)	
201859_at	-1.6	-1.1	proteoglycan 1, secretory granule	PRG1
202546_at	1	3.2	vesicle-associated membrane protein 8 (endobrevin)	VAMP8
206382_s_at		-2.6	Homo sapiens brain-derived neurotrophic factor	BDNF
210195_s_at	-1		Human pregnancy-specific beta-1-glycoprotein mRNA PSG95, complete cds.	PSG1
210355_at	-1.8		Human, parathyroid-like protein (associated with humoral hypercalcemia of malignancy)	PTHLH
213456_at	-1.5		DKFZP564D206	
205609_at	-2.1		angiopoietin 1	ANGPT1
206509_at	-3.9		prolactin-induced protein	PIP
202205_at	-4		vasodilator-stimulated phosphoprotein	VASP
206378_at		-5.1	mammaglobin 1	MGB1
203666_at	-1.1		stromal cell-derived factor 1	SDF1
202957_at	-1.1		hematopoietic cell-specific Lyn substrate 1	HCLS1
215599_at	-1.7		H.sapiens SMA4 mRNA.	SMA4
222043_at		-1.2	clusterin (complement lysis inhibitor, SP-40,40, sulfated glycoprotein 2, testosterone-repressed prostate message 2, apolipoprotein J)	CLU

Thyroid Hormone Related

205489_at	-1	-1.5	crystallin, mu	CRYM
212544_at		1.9	thyroid hormone receptor interactor 3	TRIP3
202734_at	-1.2		thyroid hormone receptor interactor 10	TRIP10
1316_at	-1.4		Homo sapiens mRNA for thyroid hormone receptor alpha 1 THRA1, (c-erbA-1 gene)	

Estrogen related

204278_s_at		1.6	Homo sapiens estrogen receptor binding site associated, antigen, 9 (EBAG9), mRNA	EBAG9
207981_s_at		-2.4	Homo sapiens estrogen-related receptor gamma (ESRRG), mRNA.	ESRRG

Lysosomes

201089_at	1.2		ATPase, H+ transporting, lysosomal (vacuolarproton pump), beta polypeptide, 5658kD, isoform 2	ATP6B2
208737_at		1.2	ATPase, H+ transporting, lysosomal (vacuolarproton pump), member J	ATP6J
213728_at	1.1		lysosomal-associated membrane protein 1	LAMP1
201552_at		1.9	lysosomal-associated membrane protein 1	LAMP1
213650_at	-1		golgin-67	KIAA0855
213275_x_at	-1.4	-1	cathepsin B	CTSB
213274_s_at	-1.1		cathepsin B	CTSB
203758_at		1	cathepsin O	CTSO

Endoplasmic Reticulum

211936_at	1.1		endoplasmic reticulum lumenal Ca2+ bindingprotein grp78	HSPA5
208689_s_at		1.9	ribophorin II	RPN2

Related with Blood Cells & Serum

214414_x_at	-2.7	-2.2	hemoglobin, alpha 1	HBA1
211745_x_at	-1.8		hemoglobin, alpha 2	
204848_x_at	-2.3		hemoglobin, gamma A	HBG1
204419_x_at	-2.2		hemoglobin, gamma G	HBG2
218450_at		1.6	heme-binding protein	HEBP
211696_x_at	-1.3		beta globin chain variant	HBB
204505_s_at	-1.7		erythrocyte membrane protein band 4.9 (dematin)	EPB49
40093_at		-1.4	H.sapiens LU gene for Lutheran blood group glycoprotein	

Related with Coagulation

209676_at	-1.4		tissue factor pathway inhibitor (lipoprotein-associated coagulation inhibitor)	TFPI
203305_at		-3.5	coagulation factor XIII, A1 polypeptide	F13A1
203888_at	1.1		thrombomodulin	THBD
203989_x_at	-1.2		coagulation factor II (thrombin) receptor	F2R

Others

216231_s_at		1.9	beta-2-microglobulin	B2M
207096_at	-1.4		serum amyloid A4, constitutive	

Related with Neurons, Neurogenesis & Diseases related to nervous system

207135_at	-1.2		5-hydroxytryptamine (serotonin) receptor 2A	HTR2A
207577_at	-1.1		serotonin receptor 5-HT4 (splice variant h5-HT4(b))	htr4
203999_at	-1.9		synaptotagmin I	SYT1
206161_s_at	-1		synaptotagmin V	SYT5
207594_s_at	-1	-1.7	synaptojanin 1	SYNJ1
210923_at	-1.2		solute carrier family 1 (glutamate transporter), member 7	
207184_at	-1.1	-1.5	solute carrier family 6 (neurotransmitter transporter, GABA), member 13	SLC6A13
219795_at	-1.1	-2.1	solute carrier family 6 (neurotransmitter transporter), member 14	SLC6A14
213326_at	-1.1	-1.4	vesicle-associated membrane protein 1 (synaptobrevin 1)	VAMP1
206525_at	-2.2		gamma-aminobutyric acid (GABA) receptor, rho 1	GABRR1
208217_at	-1.2		gamma-aminobutyric acid (GABA) receptor, rho 2	GABRR2
210247_at	-1.3	-1	synapsin II	SYN2
202508_s_at	-1.7		synaptosomal-associated protein, 25kD	SNAP25
206740_x_at	-1.9		synaptonemal complex protein 1	SYCP1
216021_s_at	-1.7		glycine receptor, alpha 3	GLRA3
209890_at		2.2	tetraspan NET-4	TSPAN-5
205440_s_at	1.2		neuropeptide Y receptor Y1	NPY1R
210198_s_at	-1.5		proteolipid protein (Pelizaeus-Merzbacherdisease, spastic paraplegia 2, uncomplicated)	PLP1
209693_at		-1	astrotactin2	ASTN2
202341_s_at		-1.9	tripartite motif protein TRIM2	KIAA0517
208486_at	-1.1		dopamine receptor D5	DRD5
206023_at	1.2		neuromedin U	NMU
221127_s_at		-1.6	regulated in glioma	RIG
214247_s_at		-1	regulated in glioma	RIG
203899_s_at	-1		calcitonin gene-related peptide-receptor component protein	CGRP-RCP
203898_at	-1.1		calcitonin gene-related peptide-receptor component protein	CGRP-RCP
206953_s_at	-1	-1.6	latrophilin	KIAA0786
206251_s_at	-1.1		arginine vasopressin receptor 1A	AVPR1A
205475_at	-1.2		scrapie responsive protein 1	SCRG1
205893_at	-1.2		neuroligin 1	NLGN1
210136_at		1.8	myelin basic protein	MBP
207545_s_at		-1.3	numb (Drosophila) homolog	NUMB
208626_s_at		-1.1	Similar to membrane protein of cholinergicsynaptic vesicles	VATI
201860_s_at	-1.4		plasminogen activator, tissue	PLAT
203413_at	-1.3		nel (chicken)-like 2	NELL2
219511_s_at		-1.2	Homo sapiens synuclein, alpha interacting protein (synphilin) (SNCAIP), mRNA.	SNCAIP
221832_s_at		-1.1	leucine zipper protein 1	LUZP1
220024_s_at		-1	periaxin	KIAA1620
211458_s_at		-1	GABA-A receptor-associated protein	
204237_at	-1.2	-1	CED-6 protein	CED-6
214527_s_at		1.4	PQBP-1d	PQBP1
214198_s_at		-1	DiGeorge syndrome critical region gene 2	DGCR2
213791_at	-1.1		proenkephalin	PENK
202908_at	-1.1	-1.2	Wolfram syndrome 1 (wolframin)	WFS1

Alzheimer Related

204262_s_at	-1.2		presenilin 2 isoform 1	PSEN2
215148_s_at	-1.3		amyloid beta (A4) precursor protein-binding, family A, member 3 (X11-like 2)	APBA3
202268_s_at		1.4	Amyloid beta precursor protein-binding protein1	APPBP1
207953_at	-1.3		neuronal thread protein AD7c-NTP	AD7C-NTP
200602_at		-1.2	Homo sapiens amyloid beta (A4) precursor protein (protease nexin-II, Alzheimer disease)	APP

Related to Myogenesis

210944_s_at		-1.4	Similar to calpain 3, (p94)	CAPN3
206707_x_at	-2.1		PL48	C6ORF32
221152_at		-5.1	smooth muscle cell-expressed and macrophageconditioned medium-induced protein smag-64	LOC57086
215295_at	-1.6		Homo sapiens mRNA for dystrobrevin B DTN-B2.	DTN-B2

Adipocytosis

208998_at		-1.8	uncoupling protein homolog	UCPH
207175_at	-1.9	-2.3	adipose most abundant gene transcript 1	APM1
212274_at		-1.5	lipin 1	LPIN1

Peroxisome

205160_at		-1.3	peroxisomal biogenesis factor 11A	PEX11A
205246_at		2.5	peroxisome biogenesis factor 13	PEX13
207881_at	-1.8		putative peroxisome microbody protein 175.1	LOC51051
210771_at	-3.9		Similar to peroxisome proliferative activated receptor, alpha	PPARA
320_at	-1.1		mRNA for peroxisome assembly factor-2, complete cds	

37152_at		-1.3	peroxisome proliferator activated receptor mRNA	
203105_s_at	1.4		Homo sapiens dynamin 1-like (DNM1L), transcript variant 1, mRNA.	DNML1
219428_s_at	-1.1		24 kDa intrinsic membrane protein	PMP24

Nuclear Export/Import

211762_s_at	1.1		karyopherin alpha 2 (RAG cohort 1, importinalpha 1)	
213803_at		1.2	karyopherin (importin) beta 1	KPNB1
221829_s_at	1		karyopherin (importin) beta 2	KPNB2
209226_s_at		1.5	karyopherin beta2	KPNB2
211953_s_at		1.2	karyopherin (importin) beta 3	KPNB3

Related to Nuclear Receptors

209105_at		1.3	nuclear receptor coactivator 1	NCOA1
209060_x_at		1.3	nuclear receptor coactivator 3	NCOA3
209061_at		1	nuclear receptor coactivator 3	NCOA3
206645_s_at		-2.6	nuclear receptor subfamily 0, group B, member 1	NR0B1
209750_at		2.7	nuclear receptor subfamily 1, group D, member 2	NR1D2
210531_at	-1.6		nuclear receptor subfamily 2, group C, member 1	NR2C1
209120_at		-1	nuclear receptor subfamily 2, group F, member 2	NR2F2
216979_at	-1.5		nuclear receptor	NR4A3

Other Nuclear Proteins

208835_s_at	1.1		cisplatin resistance-associated overexpressed protein	LUC7A
221699_s_at	1.2		nucleolar protein GU2	
209088_s_at	-1.3		ubinuclein 1	UBN1
200626_s_at		1.4	matrin 3	MATR3

Ataxin and Related Genes

208833_s_at	1.1		Homo sapiens E46 protein mRNA, complete cds	E46L
222252_x_at	-1.4		ataxin-1 ubiquitin-like interacting protein	A1U
221217_s_at	-2.5		ataxin 2-binding protein 1	A2BP1
205415_s_at		1.6	Machado-Joseph disease (spinocerebellar ataxia 3, olivopontocerebellar ataxia 3, autosomal dominant, ataxin 3)	MJD

Annexin and Related Genes

201012_at		1.4	annexin I	ANXA1
208816_x_at		1.8	annexin A2 / Human lipocortin (LIP) 2 pseudogene mRNA, complete cds-like region	ANXA2

Miscellenous

218177_at	1.4		CHMP1.5 protein	CHMP1.5
202793_at	2.6		putative protein similar to nessy (Drosophila)	C3F
214804_at	1.7		FSH primary response (LRPR1, rat) homolog 1	FSHPRH1
213555_at	1.6		Human DNA sequence from clone 747H23 on chromosome 6q13-15. Contains the 3 part of the ME1 gene for malic enzyme 1, soluble (NADP-dependent malic enzyme, malate oxidoreductase, EC 1.1.1.40), a novel gene and the 5 part of the gene for N-acetylglucosamine-phosphate mutase. Contains ESTs, STSs, GSSs and two putative CpG islands	
201506_at	1.2	1.3	transforming growth factor, beta-induced, 68kD	TGFBI
204868_at	1.3		immature colon carcinoma transcript 1	ICT1
202596_at	1		Homo sapiens, endosulfine alpha, clone MGC:8394, mRNA, complete cds.	ENSA
211947_s_at		3.8	KIAA1096 protein	KIAA1096
209127_s_at		2.5	KIAA0156 gene product	KIAA0156
212650_at		1.9	KIAA0903 protein	KIAA0903
201462_at		1.7	KIAA0193 gene product	KIAA0193
211944_at		1.4	KIAA1096 protein	KIAA1096
201593_s_at		1.4	uncharacterized hypothalamus protein HT010	HT010
205003_at	-1.2		KIAA0716 gene product	KIAA0716
220995_at	-1.2		HSPC156 protein	HSPC156
215012_at	-1.2		KIAA0576 protein	KIAA0576
212806_at	-1.3		KIAA0367 protein	KIAA0367
214707_x_at	-1.3		KIAA0328 protein	KIAA0328
220050_at	-1.5		chromosome 9 open reading frame 9	C9ORF9
212942_s_at	-1.6		Homo sapiens mRNA for KIAA1199 protein, partial cds.	KIAA1199
206776_x_at	-1.5		Homo sapiens acrosomal vesicle protein 1 (ACRV1), transcript variant 1, mRNA.	ACRV1
207969_x_at	-1.5		Homo sapiens acrosomal vesicle protein 1 (ACRV1), transcript variant 5, mRNA.	ACRV1
207990_x_at	-1.1		Homo sapiens acrosomal vesicle protein 1 (ACRV1), transcript variant 9, mRNA.	ACRV1
207991_x_at	-1.8		acrosomal vesicle protein 1, isoform hprecursor	ACRV1
204529_s_at	-1.8	-2.2	KIAA0808 gene product	KIAA0808
212915_at	-2	-2.6	KIAA1095 protein	KIAA1095
212012_at	-2.1	-1.9	Melanoma associated gene	D2S448
219179_at	-2.2		heptacellular carcinoma novel gene-3	LOC51339
208154_at	-2.8		mesenchymal stem cell protein DSCD28	LOC51336
214861_at	-3.1	-1.5	gene amplified in squamous cell carcinoma 1; KIAA0780 protein	KIAA0780
203491_s_at		1.2	KIAA0092 gene product	KIAA0092
212373_at		1.1	FEM-1 (C.elegans) homolog b	FEM1B
217722_s_at		1.2	mesenchymal stem cell protein DSC92	LOC51335
221974_at		-1	imprinted in Prader-Willi syndrome	IPW
213447_at	-1	-1	imprinted in Prader-Willi syndrome	IPW
207996_s_at		-3.3	chromosome 18 open reading frame 1	C18ORF1
212732_at		-1.7	maternally expressed 3	MEG3
212190_at	-1.8	-2.5	trinucleotide repeat containing 3	TNRC3
214774_x_at		-1.5	trinucleotide repeat containing 9	TNRC9
202416_at	1		tetratricopeptide repeat domain 2	TTC2

208661_s_at		1.2	tetratricopeptide repeat domain 3	TTC3
208663_s_at		2	tetratricopeptide repeat domain 3	TTC3
208662_s_at	1	1.4	tetratricopeptide repeat domain 3	TTC3
213322_at	1		Human DNA sequence from clone 34B21 on chromosome 6p12.1-21.1. Contains part of a gene for a novel protein with ZU5 domain similar to part of Tight Junction Protein ZO1 (TJP1) and UNC5 Homologs, the gene for a novel BZRP (peripheral benzodiazapine receptor (MBR,PBR, PBKS, IBP, Isoquinoline-binding protein)) LIKE protein, the gene for a novel protein similar to part of APOBEC1 (Phorbolin 1, Apolipoprotein B mRNA editing protein), and the NFYA gene for nuclear transcription factor Y, alpha (CCAAT-Binding transcription factor subunit B, CBF-B, CAAT-Box DNA binding protein subunit A). Contains ESTs, STSs, GSSs, two putative CpG islands and a ca repeat polymorphism /DEF=Human DNA sequence from clone 34B21 on chromosome 6p12.1-21.1. Contains part of a gene for a novel protein with ZU5 domain similar to part of Tight Junction Protein ZO1 (TJP1) and UNC5 Homologs, the gene for a novel BZRP (peripheral benzodiazapine...	
205809_s_at		3.3	Wiskott-Aldrich syndrome-like	WASL
221123_x_at		3.1	Homo sapiens papillomavirus regulatory factor PRF-1 (LOC55893), mRNA.	LOC55893
216580_at		3	Human DNA sequence from clone 263J7 on chromosome 6q14.3-15. Contains an RPL7 (60S Ribosomal Protein L7) pseudogene, a RAB1 (RAB1, member RAS oncogene family) pseudogene, ESTs, an STS and GSSs	
203203_s_at	1.1	1.4	HIV-1 rev binding protein 2	HRB2
221473_x_at		1.1	tumor differentially expressed 1	Diff33
202300_at		1.9	hepatitis B virus x-interacting protein	XIP
206022_at		-1.7	Norrie disease protein	NDP
212730_at		-1.9	KIAA0353 protein	KIAA0353
203940_s_at		-2.7	KIAA1036 protein	KIAA1036
221052_at		-1.2	putative RNA binding protein	TDRKH
221053_s_at		-3.8	putative RNA binding protein	TDRKH
204530_s_at		-2.8	KIAA0808 gene product	KIAA0808
215527_at		-1.8	Human DNA sequence from clone 240B8 on chromosome 6p11.2-q12. Contains the 3 part of a gene for a novel protein similar to T-STAR, Etoile, Sam68, SLM1 and p62 Tyrosine Phosphoprotein. Contains ESTs, STSs, GSSs and genomic marker D6S1695	
218917_s_at		1.2	Homo sapiens uncharacterized bone marrow protein BM029 (BM029), mRNA.	BM029
219978_s_at		1	Homo sapiens uncharacterized bone marrow protein BM037 (BM037), mRNA.	BM037
219555_s_at	1.8	3.5	uncharacterized bone marrow protein BM039	BM039
202232_s_at		2.5	Homo sapiens dendritic cell protein (GA17), mRNA	GA17
214711_at	-2		Human DNA sequence from BAC 15E1 on chromosome 12. Contains Cytochrome C Oxidase Polypeptide VIa-liver precursor gene, 60S ribosomal protein L31 pseudogene, pre-mRNA splicing factor SRp30c gene, two putative genes, ESTs, STSs and putative CpG islands	
216358_at	-1.7		Human DNA sequence from clone RP3-526L9 on chromosome 6. Contains a SMARCE1 (SWISNF related, matrix associated, actin dependent regulator of chromatin, subfamily e, member 1) pseudogene, ESTs and GSSs	
215100_at	-1.5		Human DNA sequence from clone 413H6 on chromosome 6p22.3-24.3. Contains a hamster Androgen-dependent Expressed Protein like protein gene, ESTs and GSSs	
215082_at	-1.1		Human DNA sequence from clone RP3-483K16 on chromosome 6p12.1-21.1. Contains (parts of) two novel genes, RPS16 (40S Ribosomal protein S16) and RPL31 (60S Ribosomal protein L31) pseudogenes, ESTs, STSs, GSSs and four putative CpG islands	
217322_x_at	1		Human DNA sequence from clone 522P13 on chromosome 6p21.31-22.3. Contains a 60S Ribosomal Protein L21 pseudogene and an HNRNP A3 (Heterogenous Nuclear Riboprotein A3, FBRNP) pseudogene. Contains ESTs, STSs and GSSs	
212144_at		-1.3	Human DNA sequence from clone RP3-508I15 on chromosome 22q12-13 Contains the gene for GTPBP1 (GTP binding protein 1), two novel genes KIAA0063 and KIAA0668, a novel gene based on ESTs and cDNA, a pseudogene similar to AOP1 (antioxidant protein 1)...	
222023_at		-4	Homo sapiens cDNA FLJ11952 fis, clone HEMBB1000831, weakly similar to Homo sapiens breast cancer nuclear receptor-binding auxiliary protein (BRX) mRNA	
216505_x_at		1.7	Human DNA sequence from clone RP11-371L19 on chromosome 20 Contains a novel gene, a gene similar to the gene for ribosomal protein S10, ESTs, STSs, GSSs and CpG islands	
200099_s_at		1.5	Human DNA sequence from clone RP11-486O22 on chromosome 10 Contains the 3part of a gene for KIAA1128 protein, a novel pseudogene, a gene for protein similar to RPS3A (ribosomal protein S3A), ESTs, STSs, GSSs and CpG islands	
213226_at	1.2		polymyositisscleroderma autoantigen 1 (75kD)	PMSCL1
211075_s_at	1.1	1.8	integrin associated protein	
208631_s_at	1.1		78 kDa gastrin-binding protein	HADHA
213729_at	1	1.1	Huntingtin-interacting protein A	HYPA
219910_at	-1.3		Huntingtin interacting protein E	HYPE
204994_at	-1		myxovirus (influenza) resistance 2, homolog of murine	MX2
211460_at	-1.1		Homo sapiens testis transcript Y 9 (TTY9) mRNA, complete cds.	TTY9
211014_s_at	-1.2		Homo sapiens tripartite motif protein TRIM19 kappa mRNA, complete cds.	PML
208360_s_at	-1.7		endogenous retrovirus H D1 leaderregionintegrase-derived ORF1, ORF2, and putative envelopeprotein	HSU88895
209700_x_at	-2.1		Homo sapiens mRNA, similar to rat myomegalin, complete cds.	LOC64182
34764_at		-1	Human mRNA for KIAA0028 gene, partial cds	
211276_at	-1.3	-2.4	brain my048 protein	
220770_s_at		2.1	Homo sapiens transposon-derived Buster3 transposase-like (LOC63920), mRNA.	LOC63920
218876_at		1.8	brain specific protein	LOC51673
209875_s_at		1.7	Human nephropontin mRNA, complete cds.	
217966_s_at		1.7	niban protein	NIBAN
203345_s_at	1.3	1.7	putative DNA binding protein	M96
212018_s_at		1.6	Homo sapiens cDNA FLJ20815 fis, clone ADSE01038, highly similar to AJ007398 Homo sapiens mRNA for PBK1 protein.	DKFZP564M182
208778_s_at		1.6	t-complex 1	TCP1
218320_s_at		1.6	similar to mouse neuronal protein 15.6	FLJ20494
211066_x_at		-1.2	Homo sapiens, Similar to protocadherin gamma subfamily A, 5, clone MGC:13163, mRNA, complete cds.	
201165_s_at		1	pumilio (Drosophila) homolog 1	PUM1
216187_x_at	-1.6		Homo sapiens Alu repeat (LNX1) mRNA sequence.	
212496_s_at		-1.2	KIAA0876 protein	KIAA0876

215184_at		-1.2	Homo sapiens cDNA: FLJ23148 fis, clone LNG09313, highly similar to AB018001 Homo sapiens mRNA for Death-associated protein kinase 2	
203713_s_at		-1.2	lethal giant larvae (Drosophila) homolog 2	LLGL2
213796_at		-1.7	Small proline-rich protein SPRK (human, odontogenic keratocysts, mRNA Partial, 317 nt)	
216249_at		-1.7	Human transcription unit PVT gene, exons I-III.	
205064_at	-1.5	-1.3	small proline-rich protein 1B (cornifin)	SPRR1B
217902_s_at		-1.1	hect domain and RLD 2	HERC2
221221_s_at		-1.1	kelch (Drosophila)-like 3	KLHL3
203255_at		-1.2	vitiligo-associated protein VIT-1	VIT1
202170_s_at		1.2	Homo sapiens HSPC223 mRNA, complete cds.	AASDHPPT
203428_s_at		1.2	Homo sapiens mRNA for CIA, complete cds.	DKFZP547E2110
212028_at		1.4	S164 protein	S164
214235_at		-1.1	H.sapiens DNA for cyp related pseudogene.	
208238_x_at	-1.2		leucine zipper-like protein	LZLP
213169_at		-1.3	Homo sapiens clone TUA8 Cri-du-chat region mRNA	
209997_x_at	-1.2		Similar to pericentriolar material 1	
215810_x_at	-1.1		Dystonia musculorum of mouse, human homolog of	D6S1101
218049_s_at		1.5	L13 protein	L13
217457_s_at	1		smg GDS	hGDS
218970_s_at	1		CGI-32 protein	LOC51076
220755_s_at	1.1		G8 protein	G8
205525_at	-1.1		NAG22 protein	LOC55873
215985_at	-1.1		HCGVIII-1 protein	HCGVIII-1
40020_at	-1.1		Homo sapiens mRNA for MEGF2, partial cds	
220158_at	-1.1		placental protein 13-like protein	LOC56891
215817_at	-1.2		UV-B repressed sequence, HUR 7	HSHUR7SEQ
209737_at	-1.2		KIAA0705 protein	KIAA0705
212679_at	-1.2		transducin (beta)-like 2	TBL2
210873_x_at	-1.2		phorbolin I	DJ742C19.2
203854_at	-1.3		I factor (complement)	IF
206767_at	-1.3		RNA binding motif, single stranded interactingprotein 3	RBMS3
216931_at	-1.3		Homo sapiens (clone Z146) retinal mRNA, 3 end and repeat region.	
214409_at	-1.3		ret finger protein-like 3 antisense	RFPL3S
222106_at	-1.3		prion gene complex, downstream	PRND
201645_at	-1.3		hexabrachion (tenascin C, cytotactin)	HXB
220951_s_at	-1.3		Apobec-1 complementation factor; APOBEC-1stimulating protein	ACF
222187_x_at	-1.3		H.sapiens mRNA for TRE5.	
213332_at	-1.3		similar to pregnancy-associated plasma protein A precursor	LOC57036
201981_at	-2.1		pregnancy-associated plasma protein A	PAPPA
211071_s_at	-1.3	-1	ALL1-fused gene from chromosome 1q	
215011_at	-1.3		Homo sapiens RNA transcript from U17 small nucleolar RNA host gene, variant U17HG-AB.	RNU17D
37022_at	-1.4		Human prolargin (PRELP) gene, 5 flanking sequence and	
209584_x_at	-1.4		phorbolin I protein	PBI
210943_s_at	-1.4		Chediak-Higashi syndrome protein short isoform	LYST
214772_at	-1.5	-1.6	G2 protein	G2
220792_at	-1.5		PR domain containing 5	PRDM5
219746_at	-1.5		cer-d4 (mouse) homolog	CERD4
205328_at	-1.5		claudin 10	CLDN10
211751_at	-1.6		similar to rat myomegalin	
211055_s_at	-1.7		Similar to inversin	
205636_at	-1.9		Homo sapiens EEN-B2-L1 mRNA, complete cds.	EEN-B2-L1
217020_at	-1.9		Homo sapiens DNA for HBV integration sites	
221492_s_at		1.3	PC3-96	PC3-96
219372_at		1.3	CDV-1 protein	CDV-1
203893_at		1.3	adrenal gland protein AD-004	
208029_s_at		1.4	putative integral membrane transporter	LC27
211000_s_at		1.4	gp130 of the rheumatoid arthritis antigenicpeptide-bearing soluble form (gp130-RAPS)	gp130
209219_at		1.2	RD protein	RD
212208_at		1.2	Homo sapiens cDNA FLJ13775 fis, clone PLACE4000369, moderately similar to Homo sapiens thyroid hormone receptor-associated protein complex component TRAP240 mRNA.	KIAA1025
204409_s_at		1.1	Homo sapiens, clone MGC:12282, mRNA, complete cds.	EIF1AY
219843_at		1.1	intracisternal A particle-promoted polypeptide	IPP
202124_s_at		1.1	amyotrophic lateral sclerosis 2 (juvenile) chromosome region, candidate 3	ALS2CR3
221702_s_at		1	BBP-like protein 2	BLP2
209254_at		1	KIAA0265 protein	KIAA0265
200616_s_at		1	KIAA0152 gene product	KIAA0152
36612_at		-1	Human mRNA for KIAA0280 gene, partial cds	
205428_s_at		-1	calbindin 2, full length protein isoform	CALB2
206734_at		-1.1	jerky (mouse) homolog-like	JRKL
213900_at		-1.1	Friedreich ataxia region gene X123	X123
220237_at		-1.2	PC3-96 protein	PC3-96
217128_s_at		-1.3	Human gene isolated from PAC 272L16, chromosome 1, similar to calciumcalmodulin dependent protein kinases.	DJ272L16.1
209405_s_at		-1.3	Similar to predicted osteoblast protein	2.19
209568_s_at		-1.3	RGL protein	RGL
211403_x_at		-1.3	variably charged X-B	VCXB
220974_x_at		-1.3	similar to rat tricarboxylate carrier-likeprotein	BA108L7.2
211602_s_at		-1.4	Human alternatively spliced trp-1 protein and unspliced trp-1 protein (trp-1) mRNA, complete cds.	TRP-1
205413_at		-1.4	chromosome 11 open reading frame 8	C11ORF8
210227_at		-1.7	discs, large (Drosophila) homolog-associatedprotein 2	DLGAP2
40446_at		-1.8	Cluster Incl. AL021366:cICK0721Q.4.1 (PHD finger protein 2) (isoform 2)	
212739_s_at		-1.8	non-metastatic cells 4, protein expressed in	NME4
212013_at		-1.9	Melanoma associated gene	KIAA0230
217130_at		-2.4	HFSE-1	HFSE-1
210409_at		-3.1	myeloidlymphoid or mixed-lineage leukemia (trithorax (Drosophila) homolog); translocated to, 4	HGC6.4
210667_s_at		-4.2	KNP-Ib	KNP-I
210117_at		1.1	Homo sapiens infertility-related sperm protein mRNA, complete cds.	SPAG1

222358_x_at	-1.1		ESTs, Weakly similar to ALU1_HUMAN ALU SUBFAMILY J	
217703_x_at	-1.2		ESTs, Moderately similar to ALU7_HUMAN ALU SUBFAMILY	
217687_at	-1.2		ESTs, Weakly similar to CYA7_HUMAN ADENYLATE CYCLASE, TYPE VII	
222301_at	-1.2		ESTs, Highly similar to transcriptional acitvator	
217713_x_at	-1.3		ESTs, Weakly similar to ALU6_HUMAN ALU SUBFAMILY SP	
222377_at	-1.3		ESTs, Highly similar to T-box-containing transcriptional activator	
217679_x_at	-1.4		ESTs, Moderately similar to ALU8_HUMAN ALU SUBFAMILY SX	
217534_at	-1.7		ESTs, Weakly similar to ALU7_HUMAN ALU SUBFAMILY SQ	
217586_x_at	-1.7		ESTs, Weakly similar to ALU1_HUMAN ALU SUBFAMILY J	
217579_x_at	-1.7		ESTs, Weakly similar to ALU1_HUMAN ALU SUBFAMILY J	
216874_at	-1.7		Human EST clone 251800 mariner transposon Hsmar1 sequence	
221734_at		3.6	ESTs, Weakly similar to A43932 mucin 2 precursor, intestinal	
217602_at		2.7	ESTs	
217550_at		1.9	ESTs	
221883_at		1.1	ESTs	
221765_at		1	ESTs	
221961_at		-1	ESTs	
222369_at		-1	ESTs	
217714_x_at		-1.1	ESTs	
222326_at		-1.1	ESTs	
222275_at		-1.2	ESTs	
222273_at		-1.2	ESTs	
222280_at		-1.2	ESTs, Weakly similar to ALUC_HUMAN	
222361_at		-1.4	ESTs, Moderately similar to TBB2_HUMAN TUBULIN BETA-2 CHAIN	
204825_at	2		KIAA0175 gene product	KIAA0175
218039_at	1.5		clone HQ0310 PRO0310p1	LOC51203
220030_at	4.2		hypothetical protein DKFZp761P1010	DKFZp761P1010
218890_x_at	2.4		hypothetical protein (LOC51318)	LOC51318
218751_s_at	2.3		hypothetical protein FLJ11071	FLJ11071
218542_at	2	2.2	hypothetical protein FLJ10540 (FLJ10540)	FLJ10540
209683_at	1.6		hypothetical protein DKFZp566A1524	DKFZP566A1524
220199_s_at	1.5		hypothetical protein FLJ12806 (FLJ12806)	FLJ12806
219918_s_at	1.4	1.3	hypothetical protein FLJ10517 (FLJ10517)	FLJ10517
218123_at	1.4	1.5	chromosome 21 open reading frame 59 (C21ORF59)	C21ORF59
219002_at	1.4		hypothetical protein FLJ21901 (FLJ21901)	FLJ21901
222151_s_at	1.3		hypothetical protein FLJ13386	FLJ13386
219493_at	1.3		hypothetical protein FLJ22009 (FLJ22009)	FLJ22009
221771_s_at	1.3		Unknown (protein for IMAGE:3611719)	
209479_at	1.3		Homo sapiens, clone MGC:2698, mRNA, complete cds.	DKFZP586D0623
206316_s_at	1.3		KIAA0166 gene product	KIAA0166
206364_at	1.3		KIAA0042 gene product	KIAA0042
206860_s_at	1.3		hypothetical protein (FLJ20323)	FLJ20323
219990_at	1.3		hypothetical protein FLJ23311 (FLJ23311)	FLJ23311
213718_at	1.2		Homo sapiens, clone MGC:10871, mRNA, complete cds	
219588_s_at	1.2		hypothetical protein FLJ20311 (FLJ20311)	FLJ20311
208868_s_at	1.2		Homo sapiens mRNA; cDNA DKFZp564N1272 (from clone DKFZp564N1272); complete cds	
220477_s_at	1.1		HSPC274 protein	HSPC274
212738_at	1.1		Human clone 23719 mRNA sequence	
221452_s_at	1.1	1	hypothetical protein MGC1223 (MGC1223)	MGC1223
220329_s_at	1.1		hypothetical protein FLJ20627 (FLJ20627)	FLJ20627
219054_at	1.1		hypothetical protein FLJ14054 (FLJ14054)	FLJ14054
214876_s_at	1.1		cDNA FLJ13872 fis, clone THYRO1001322	
218883_s_at	1.1		hypothetical protein FLJ23468 (FLJ23468)	FLJ23468
219355_at	1.1		hypothetical protein FLJ10178 (FLJ10178)	FLJ10178
218349_s_at	1.1	2.3	hypothetical protein FLJ10036 (FLJ10036)	FLJ10036
211505_s_at	1.1		mRNA; cDNA DKFZp564J1516 (from clone DKFZp564J1516); complete cds	DKFZp564J1516
213092_x_at	1.1		KIAA0974 protein	KIAA0974
209092_s_at	1.1		Homo sapiens clone 016b03 My027 protein mRNA, complete cds.	LOC51031
213379_at	1.1		Homo sapiens clone 640 unknown mRNA, complete sequence.	CL640
219004_s_at	1.1		chromosome 21 open reading frame 45	C21ORF45
217919_s_at	1		PTD007 protein	PTD007
202887_s_at	1		hypothetical protein (FLJ20500)	FLJ20500
219122_s_at	1		hypothetical protein FLJ20546 (FLJ20546)	FLJ20546
220060_s_at	1		hypothetical protein FLJ20641 (FLJ20641)	FLJ20641
219787_s_at	1	1.1	hypothetical protein FLJ10461 (FLJ10461)	FLJ10461
221986_s_at	1	2.5	hypothetical protein FLJ20059	FLJ20059
218622_at	1	1.6	hypothetical protein MGC5585 (MGC5585)	MGC5585
219979_s_at	1	2.1	hypothetical protein (HSPC138)	HSPC138
218852_at	1		hypothetical protein FLJ20644 (FLJ20644)	FLJ20644
218558_s_at	1		hypothetical protein	PRED22
218859_s_at	1		HDCMC28P protein	HDCMC28P
221766_s_at	1		hypothetical protein FLJ20037	FLJ20037
33768_at	-1		Homo sapiens 59 protein mRNA, 3 end	
209766_at	-1		Homo sapiens PRO1748 mRNA, complete cds.	PRO1748
213109_at	-1		KIAA0551 protein	KIAA0551
214902_x_at	-1		mRNA; cDNA DKFZp586A061 (from clone DKFZp586A061)	
216678_at	-1		cDNA FLJ20766 fis, clone COL07978	
220596_at	-1		DKFZP434F1735 protein (DKFZP434F1735), mRNA	DKFZP434F1735
220725_x_at	-1		hypothetical protein FLJ23558 (FLJ23558)	FLJ23558
215387_x_at	-1		cDNA FLJ11443 fis, clone HEMBA1001330	
216682_s_at	-1		cDNA FLJ11395 fis, clone HEMBA1000594	
221030_s_at	-1		hypothetical protein DKFZp564B1162 (DKFZP564B1162)	DKFZP564B1162
59437_at	-1		IMAGE-2406340	
221629_x_at	-1		HSPC188 mRNA, complete cds	LOC51236
219981_x_at	-1		hypothetical protein FLJ20813 (FLJ20813)	FLJ20813
213486_at	-1	-1.6	hypothetical protein DKFZp761N09121	DKFZP761N09121
81811_at	-1		IMAGE-2362767	
213686_at	-1		clone 24583 mRNA sequence	

219706_at	-1		hypothetical protein FLJ11168 (FLJ11168)	FLJ11168
206548_at	-1		hypothetical protein FLJ23556 (FLJ23556)	FLJ23556
222108_at	-1	-1.4	Human BAC clone GS1-99H8	
44696_at	-1		IMAGE-1473925	
219757_s_at	-1		hypothetical protein FLJ20392 (FLJ20392)	FLJ20392
212419_at	-1	-2	mRNA; cDNA DKFZp564L0822 (from clone DKFZp564L0822)	
219732_at	-1	-1.4	hypothetical protein FLJ20300 (FLJ20300)	FLJ20300
215128_at	-1		cDNA FLJ11682 fis, clone HEMBA1004880	
214722_at	-1		cDNA FLJ11946 fis, clone HEMBB1000709	
221038_at	-1		hypothetical protein PRO1942 (PRO1942)	PRO1942
44702_at	-1	-1.3	IMAGE-144061	
210723_x_at	-1		clone MGC:4771	
201775_s_at	-1		KIAA0494 gene product	KIAA0494
214785_at	-1		Homo sapiens mRNA for KIAA0986 protein, partial cds.	KIAA0986
204308_s_at	-1		KIAA0329 gene product	KIAA0329
212976_at	-1		KIAA0231 protein	KIAA0231
202972_s_at	-1		KIAA0914 gene product	KIAA0914
215848_at	-1		KIAA1454 protein	KIAA1454
213056_at	-1		KIAA1013 protein	KIAA1013
49329_at	-1		IMAGE-273900	
41553_at	-1		IMAGE-2390973	
206818_s_at	-1		hypothetical protein FLJ20064 (FLJ20064)	FLJ20064
216739_at	-1		cDNA: FLJ20874 fis, clone ADKA02818	
220227_at	-1		hypothetical protein FLJ22202 (FLJ22202)	FLJ22202
220578_at	-1		hypothetical protein FLJ13544 (FLJ13544)	FLJ13544
59705_at	-1		IMAGE-1476617	
217629_at	-1.1		ESTs	
211406_at	-1.1		PRO2309	HSPC039
211445_x_at	-1.1		FKSG17	FKSG17
215109_at	-1.1		KIAA0492 protein	KIAA0492
213922_at	-1.1		KIAA0847 protein	KIAA0847
201815_s_at	-1.1		KIAA0210 gene product	KIAA0210
209048_s_at	-1.1		KIAA1125 protein	KIAA1125
218155_x_at	-1.1		hypothetical protein FLJ10534	FLJ10534
220897_at	-1.1	-1.5	hypothetical protein FLJ11556 (FLJ11556)	FLJ11556
220796_x_at	-1.1		hypothetical protein FLJ14251 (FLJ14251)	FLJ14251
220071_x_at	-1.1		hypothetical protein FLJ10460 (FLJ10460)	FLJ10460
215521_at	-1.1		hypothetical protein FLJ12729	FLJ12729
214053_at	-1.1		clone 23736 mRNA sequence	
219951_s_at	-1.1	-1.2	hypothetical protein FLJ10600 (FLJ10600)	FLJ10600
219156_at	-1.1		hypothetical protein FLJ11271 (FLJ11271)	FLJ11271
215557_at	-1.1		cDNA FLJ10146 fis, clone HEMBA1003327	
49878_at	-1.1		IMAGE-936303	
209656_s_at	-1.1	-2.4	mRNA; cDNA DKFZp761J17121 (from clone DKFZp761J17121)	DKFZp761J17121
215032_at	-1.1		cDNA FLJ12380 fis, clone MAMMA1002556	
214862_x_at	-1.1		mRNA; cDNA DKFZp564G1162 (from clone DKFZp564G1162)	
39891_at	-1.1	-1	IMAGE-1871401	
220826_at	-1.1		hypothetical protein FLJ10932 (FLJ10932)	FLJ10932
216153_x_at	-1.1		cDNA FLJ12835 fis, clone NT2RP2003165	
217991_x_at	-1.1		hypothetical protein FLJ10355 (FLJ10355)	FLJ10355
217197_x_at	-1.1		Novel human gene mapping to chomosome 13	
44563_at	-1.1		IMAGE-2408049	
219194_at	-1.2		KIAA1619 protein	KIAA1619
207436_x_at	-1.2		KIAA0894 protein	KIAA0894
220672_at	-1.2		hypothetical protein MGC4163	KIAA1622
213605_s_at	-1.2		mRNA; cDNA DKFZp564F112 (from clone DKFZp564F112)	
206557_at	-1.2		hypothetical protein FLJ12985 (FLJ12985)	FLJ12985
220215_at	-1.2		hypothetical protein FLJ12606 (FLJ12606)	FLJ12606
208849_at	-1.2		PRO2047	PRO2047
215208_x_at	-1.2		cDNA FLJ11509 fis, clone HEMBA1002166	
222370_x_at	-1.2		IMAGE:246776	
211422_at	-1.2		cDNA DKFZp761A19121 (from clone DKFZp761A19121)	
215898_at	-1.2		cDNA FLJ11817 fis, clone HEMBA1006421	
222079_at	-1.2		IMAGE:3576732	
218814_s_at	-1.2		hypothetical protein FLJ10874 (FLJ10874)	FLJ10874
215445_x_at	-1.2		clone 23605 mRNA sequence	
216466_at	-1.2		mRNA; cDNA DKFZp566D153 (from clone DKFZp566D153)	
215404_x_at	-1.2		cDNA FLJ14326 fis, clone PLACE4000247	
220352_x_at	-1.2		hypothetical protein MGC4278 (MGC4278)	MGC4278
215397_x_at	-1.2		cDNA FLJ12379 fis, clone MAMMA1002554	
58780_s_at	-1.2	-1.6	IMAGE-30831	
215200_x_at	-1.2		cDNA FLJ12300 fis, clone MAMMA1001854	
221104_s_at	-1.2		hypothetical protein FLJ11275 (FLJ11275)	FLJ11275
216159_s_at	-1.2		cDNA FLJ13695 fis, clone PLACE2000124	
220823_at	-1.2		hypothetical protein FLJ20019 (FLJ20019)	FLJ20019
216121_at	-1.2		mRNA; cDNA DKFZp566O053 (from clone DKFZp566O053)	
63305_at	-1.2		GEN-408A08	
218175_at	-1.2		hypothetical protein FLJ22471 (FLJ22471)	FLJ22471
220242_x_at	-1.2		hypothetical protein FLJ10891 (FLJ10891)	FLJ10891
214762_at	-1.2		Homo sapiens DNA, cosmid clones TN62 and TN82	
217643_x_at	-1.2		IMAGE:784743	
215321_at	-1.2		BAC clone CTB-60N22 from 7q21	
207728_at	-1.2		hypothetical protein FLJ10139 (FLJ10139)	FLJ10139
220873_at	-1.2		hypothetical protein PRO0386 (PRO0386)	PRO0386
203640_at	-1.2		hypothetical protein PRO2032	PRO2032
215528_at	-1.2		mRNA; cDNA DKFZp586O1318 (from clone DKFZp586O1318)	
208015_at	-1.2		DKFZP586M0622 protein (DKFZP586M0622)	DKFZP586M0622
203546_at	-1.2		KIAA0724 gene product	KIAA0724
216374_at	-1.2		BAC clone RP11-155J5 from Y	

215183_at	-1.2		clone HQ0072.	
221910_at	-1.2		clone 24421 mRNA sequence	
220082_at	-1.2		hypothetical protein FLJ20251 (FLJ20251)	FLJ20251
216310_at	-1.2		KIAA1361 protein	KIAA1361
218554_s_at	-1.2		hypothetical protein ASH1	ASH1
211364_at	-1.2		Homo sapiens hypothetical methylthioadenosine phosphorylase fusion protein mRNA, complete cds.	
214923_at	-1.2		Homo sapiens cDNA FLJ10293 fis, clone NT2RM1000280, highly similar to VACUOLAR ATP SYNTHASE SUBUNIT D (EC 3.6.1.34)	
220853_at	-1.2		PRO0159 protein	PRO0159
215754_at	-1.3		Homo sapiens cDNA FLJ12370 fis, clone MAMMA1002428, weakly similar to LYSOSOME MEMBRANE PROTEIN II	
215175_at	-1.3		KIAA0995 protein	KIAA0995
215750_at	-1.3		KIAA1659 protein	KIAA1659
215999_at	-1.3		chromosome 17 open reading frame 1A	C17ORF1A
209829_at	-1.3		chromosome 6 open reading frame 32	KIAA0386
216863_s_at	-1.3		KIAA0852 protein	KIAA0852
205862_at	-1.3		KIAA0575 gene product	KIAA0575
210718_s_at	-1.3		Homo sapiens PRO2667 mRNA, complete cds.	
215383_x_at	-1.3		mRNA; cDNA DKFZp761K23121 (from clone DKFZp761K23121)	
209841_s_at	-1.3		mRNA; cDNA DKFZp761K2424 (from clone DKFZp761K2424)	
217446_x_at	-1.3		mRNA; cDNA DKFZp434M054 (from clone DKFZp434M054)	
213158_at	-1.3		mRNA; cDNA DKFZp586B211 (from clone DKFZp586B211)	
220533_at	-1.3	-1.4	hypothetical protein FLJ13385 (FLJ13385)	FLJ13385
219450_at	-1.3		hypothetical protein FLJ11017 (FLJ11017)	FLJ11017
217540_at	-1.3		ESTs	
215587_x_at	-1.3		DNA FLJ13829 fis, clone THYRO1000625	
216662_at	-1.3		cDNA FLJ20138 fis, clone COL07172	
215006_at	-1.3		cDNA FLJ13754 fis, clone PLACE3000362	
220221_at	-1.3		hypothetical protein FLJ10619 (FLJ10619)	FLJ10619
222271_at	-1.3		ESTs	
213106_at	-1.3	-1.5	clone 23664 and 23905 mRNA sequence	
215608_at	-1.3		Homo sapiens cDNA FLJ11475 fis, clone HEMBA1001734, moderately similar to CADHERIN-11 PRECURSOR	
216176_at	-1.3		cDNA: FLJ21690 fis, clone COL09538	
221916_at	-1.3	-2	hypothetical protein	DKFZp434B0417
215126_at	-1.3		mRNA full length insert cDNA clone EUROIMAGE 208948	
222207_x_at	-1.3		cDNA: FLJ20949 fis, clone ADSE01902	
51226_at	-1.3		IMAGE-284209	
215600_x_at	-1.3		cDNA FLJ12112 fis, clone MAMMA1000043	
220439_at	-1.3		hypothetical protein FLJ11700 (FLJ11700)	FLJ11700
215612_at	-1.3		cDNA FLJ12349 fis, clone MAMMA1002308	
222366_at	-1.3		ESTs	
215978_x_at	-1.3		cDNA FLJ11452 fis, clone HEMBA1001435	
216081_at	-1.3		cDNA: FLJ23498 fis, clone LNG02683	
217616_at	-1.3		ESTs	
207730_x_at	-1.3		hypothetical protein FLJ20700 (FLJ20700)	FLJ20700
32540_at	-1.3		IMAGE-2388224	
217715_x_at	-1.3		ESTs	
215553_x_at	-1.3		cDNA FLJ14253 fis, clone OVARC1001376	
216703_at	-1.3		mRNA; cDNA DKFZp434N199 (from clone DKFZp434N199)	
220717_at	-1.3		hypothetical protein FLJ13166 (FLJ13166)	FLJ13166
209435_s_at	-1.3	-1	clone MGC:3182	
217662_x_at	-1.3		ESTs	
215854_at	-1.3		cDNA FLJ11844 fis, clone HEMBA1006665	
220186_s_at	-1.3		hypothetical protein FLJ20124 (FLJ20124)	FLJ20124
221159_at	-1.3		clone FLB1727 (LOC51215)	LOC51215
221214_s_at	-1.3		DKFZP586J1624 protein (DKFZP586J1624)	DKFZP586J1624
214989_x_at	-1.3		clone IMAGE:3446800	
214081_at	-1.3		clone 24787 mRNA sequence	
216005_at	-1.3		cDNA: FLJ20933 fis, clone ADSE01388	
205150_s_at	-1.4		KIAA0644 gene product	KIAA0644
215067_x_at	-1.4	-1	Homo sapiens cDNA FLJ12333 fis, clone MAMMA1002198, highly similar to THIOREDOXIN PEROXIDASE 1	
207471_at	-1.4		Homo sapiens PRO1992 mRNA, complete cds.	PRO1992
211515_s_at	-1.4		Homo sapiens HDCMD38P mRNA, complete cds	KIAA0472
41386_i_at	-1.4		Human mRNA for KIAA0346 gene, partial cds	
221155_x_at	-1.4		hypothetical protein PRO1496	PRO1496
211424_x_at	-1.4		Homo sapiens PRO0066 mRNA, complete cds.	DKFZP586A0522
215604_x_at	-1.4		cDNA FLJ13721 fis, clone PLACE2000450	
220719_at	-1.4		hypothetical protein FLJ13769 (FLJ13769)	FLJ13769
215204_at	-1.4	-1.1	cDNA FLJ14090 fis, clone MAMMA1000264	
220906_at	-1.4		hypothetical protein FLJ13885 (FLJ13885)	FLJ13885
215150_at	-1.4		clone HQ0131 PRO0131	
221120_at	-1.4		hypothetical protein FLJ20306 (FLJ20306)	FLJ20306
214293_at	-1.4		hypothetical protein FLJ10849	FLJ10849
222286_at	-1.4		ESTs	
221625_at	-1.4		cDNA DKFZp761G18121 (from clone DKFZp761G18121)	
220720_x_at	-1.4		hypothetical protein FLJ14346 (FLJ14346)	FLJ14346
219071_x_at	-1.4		hypothetical protein (LOC51236)	LOC51236
214807_at	-1.4		mRNA; cDNA DKFZp564O0862 (from clone DKFZp564O0862)	
215786_at	-1.4		cDNA FLJ12108 fis, clone MAMMA1000009	
219834_at	-1.4		hypothetical protein FLJ21579 (FLJ21579)	FLJ21579
215435_at	-1.4		cDNA FLJ11921 fis, clone HEMBB1000318	
208137_x_at	-1.4		hypothetical protein MGC5384 (MGC5384)	MGC5384
209288_s_at	-1.4		mRNA; cDNA DKFZp434A0530 (from clone DKFZp434A0530)	
220338_at	-1.4		hypothetical protein FLJ10244 (FLJ10244)	FLJ10244
215191_at	-1.4		cDNA FLJ14085 fis, clone HEMBB1002534	
219975_x_at	-1.4		hypothetical protein FLJ11106 (FLJ11106)	FLJ11106

44822_s_at	-1.4		IMAGE-2501711	
216287_at	-1.4		cDNA FLJ11868 fis, clone HEMBA1006993	
216114_at	-1.4		mRNA; cDNA DKFZp586H201 (from clone DKFZp586H201)	
219472_at	-1.4		hypothetical protein MGC11266 (MGC11266)	MGC11266
213413_at	-1.4	-1.6	cDNA FLJ13555 fis, clone PLACE1007677	
212772_s_at	-1.4		cDNA DKFZp547P193 (from clone DKFZp547P193); partial cds	DKFZp547P193
71933_at	-1.4		IMAGE-1845931	
220537_at	-1.4		hypothetical protein FLJ20126 (FLJ20126)	FLJ20126
210679_x_at	-1.4		clone MGC:3878, mRNA, complete cds	BCL7A
215373_x_at	-1.4		hypothetical protein FLJ12151	FLJ12151
214068_at	-1.4		clone 24505 mRNA sequence	
216206_x_at	-1.4		clone IMAGE:3829438, mRNA, partial cds	
217519_at	-1.4		ESTs	
217653_x_at	-1.4		ESTs	
215063_x_at	-1.4		mRNA; cDNA DKFZp547B026 (from clone DKFZp547B026)	
208246_x_at	-1.4		hypothetical protein FLJ20006 (FLJ20006)	FLJ20006
207365_x_at	-1.4		KIAA0570 gene product	KIAA0570
212354_at	-1.5		KIAA1077 protein	KIAA1077
213089_at	-1.5		Human DNA sequence from clone RP11-110H4 on chromosome 5 Contains a pseudogene similar to GUSB (glucuronidase, beta), a gene for a membrane protein, ESTs, STSs, GSSs and CpG islands	
219717_at	-1.5		hypothetical protein FLJ20280 (FLJ20280)	FLJ20280
215688_at	-1.5		mRNA full length insert cDNA clone EUROIMAGE 288936	
48031_r_at	-1.5	-1.7	IMAGE-241806	
213001_at	-1.5		clone 23767 and 23782 mRNA sequences	
220398_at	-1.5		hypothetical protein MGC4170 (MGC4170)	MGC4170
215801_at	-1.5		mRNA; cDNA DKFZp434G1615 (from clone DKFZp434G1615)	
220774_at	-1.5		hypothetical protein FLJ20071 (FLJ20071)	FLJ20071
218309_at	-1.5	-1.3	hypothetical protein PRO1489 (PRO1489)	PRO1489
215057_at	-1.5		clone 161455 breast expressed mRNA from chromosome X	
215246_at	-1.5		cDNA FLJ20082 fis, clone COL03245	
214715_x_at	-1.5		mRNA for FLJ00032 protein, partial cds	
44040_at	-1.5		IMAGE-936607	
215588_x_at	-1.5		cDNA: FLJ21305 fis, clone COL02124	
215602_at	-1.5		mRNA for FLJ00048 protein, partial cds	FLJ00048
220944_at	-1.5		hypothetical protein SBBI67 (LOC57115)	LOC57115
215864_at	-1.5		cDNA: FLJ21424 fis, clone COL04157	
215284_at	-1.5		clone 24407 mRNA sequence	
216012_at	-1.5		unidentified mRNA, partial sequence	
215179_x_at	-1.5		cDNA FLJ13781 fis, clone PLACE4000465	
216090_x_at	-1.5		mRNA full length insert cDNA clone EUROIMAGE 117929	
34206_at	-1.5		Homo sapiens mRNA for KIAA0782 protein, partial cds	
217310_s_at	-1.6		KIAA1041 protein	KIAA1041
212233_at	-1.6	-1.3	mRNA for 3UTR of unknown protein	
220517_at	-1.6		hypothetical protein FLJ10381 (FLJ10381)	FLJ10381
220195_at	-1.6		hypothetical protein FLJ11113 (FLJ11113)	FLJ11113
204665_at	-1.6		hypothetical protein FLJ21168 (FLJ21168)	FLJ21168
215625_at	-1.6		cDNA FLJ10586 fis, clone NT2RP2003986	
220580_at	-1.6		hypothetical protein FLJ22476 (FLJ22476)	FLJ22476
213909_at	-1.6		cDNA FLJ12280 fis, clone MAMMA1001744	
216527_at	-1.6		mRNA; cDNA DKFZp564D193 (from clone DKFZp564D193)	
216440_at	-1.6		cDNA: FLJ21215 fis, clone COL00526	
215957_at	-1.6		unknown mRNA, sequence	
215888_at	-1.6		cDNA: FLJ23236 fis, clone COL00725	
208120_x_at	-1.6		hypothetical protein FKSG63 (FKSG63)	FKSG63
215768_at	-1.7		mRNA; cDNA DKFZp564P016 (from clone DKFZp564P016)	
220063_at	-1.7		hypothetical protein FLJ13273 (FLJ13273)	FLJ13273
215306_at	-1.7	-2.3	mRNA; cDNA DKFZp586N2020 (from clone DKFZp586N2020)	
220484_at	-1.7		hypothetical protein FLJ11006 (FLJ11006)	FLJ11006
215628_x_at	-1.7		mRNA; cDNA DKFZp564M193 (from clone DKFZp564M193)	
218211_s_at	-1.7	-1.6	hypothetical protein MGC2771 (MGC2771)	MGC2771
215439_x_at	-1.7		cDNA FLJ11924 fis, clone HEMBB1000343	
215206_at	-1.7		cDNA: FLJ21490 fis, clone COL05464	
219572_at	-1.7	-1.4	hypothetical protein FLJ20761 (FLJ20761)	FLJ20761
216110_x_at	-1.7		cDNA FLJ14080 fis, clone HEMBB1002152	
217554_at	-1.7		ESTs	
216647_at	-1.7		mRNA; cDNA DKFZp586L1824 (from clone DKFZp586L1824)	
217449_at	-1.7		mRNA; cDNA DKFZp434D1516 (from clone DKFZp434D1516)	
212574_x_at	-1.7		chromosome 19, cosmid R32184	
59433_at	-1.7		IMAGE-272275	
217042_at	-1.7		mRNA; cDNA DKFZp564M1462 (from clone DKFZp564M1462); partial cds	
217164_at	-1.7		cDNA FLJ14046 fis, clone HEMBA1006461	
216444_at	-1.7		cDNA FLJ14076 fis, clone HEMBB1001925	
216175_at	-1.7		cDNA: FLJ21623 fis, clone COL07915	
216229_x_at	-1.7		H.sapiens HCG II mRNA.	HCGII-7
222073_at	-1.8		ESTs	
215479_at	-1.8	-1.4	cDNA FLJ20780 fis, clone COL04256	
216499_at	-1.8		mRNA; cDNA DKFZp434K0610 (from clone DKFZp434K0610)	
220523_at	-1.8		hypothetical protein FLJ22601 (FLJ22601)	FLJ22601
215250_at	-1.8		cDNA FLJ12140 fis, clone MAMMA1000340	
215132_at	-1.8		mRNA; cDNA DKFZp434E2423 (from clone DKFZp434E2423)	
220586_at	-1.8		hypothetical protein FLJ12178 (FLJ12178)	FLJ12178
209436_at	-1.8		Homo sapiens mRNA for KIAA0762 protein, partial cds.	KIAA0762
220572_at	-1.9		hypothetical protein DKFZp547G183 (DKFZp547G183)	DKFZp547G183
216051_x_at	-1.9		cDNA FLJ11983 fis, clone HEMBB1001337	
214920_at	-1.9		cDNA FLJ11022 fis, clone PLACE1003771	
220467_at	-1.9		hypothetical protein FLJ21272 (FLJ21272)	FLJ21272
215825_at	-1.9		clone 24487 mRNA sequence	
215405_at	-1.9		cDNA: FLJ21419 fis, clone COL04084	

215311_at	-1.9		mRNA full length insert cDNA clone EUROIMAGE 21920	
214078_at	-2		clone 24540 mRNA sequence	
222300_at	-2		ESTs	
216595_at	-2		mRNA; cDNA DKFZp564D042 (from clone DKFZp564D042)	
213429_at	-2		cDNA DKFZp564B222 (from clone DKFZp564B222)	
221011_s_at	-2		hypothetical protein DKFZp566J091 (DKFZP566J091)	DKFZP566J091
215039_at	-2		Homo sapiens cDNA FLJ11317 fis, clone PLACE1010261, moderately similar to SEGREGATION DISTORTER PROTEIN	
216086_at	-2		Homo sapiens mRNA for KIAA1054 protein, partial cds.	KIAA1054
220608_s_at	-2		PRO1914 protein	PRO1914
215597_x_at	-2.1		cDNA FLJ11353 fis, clone HEMBA1000042	
205955_at	-2.2		hypothetical protein FLJ11136 (FLJ11136)	FLJ11136
218974_at	-2.2	-1.4	hypothetical protein FLJ10159 (FLJ10159)	FLJ10159
220575_at	-2.2		hypothetical protein FLJ11800 (FLJ11800)	FLJ11800
213125_at	-2.2		DKFZP586L151 protein	DKFZP586L151
213241_at	-2.2	-1.5	clone 23785 mRNA sequence	
216712_at	-2.2		mRNA; cDNA DKFZp762O1415 (from clone DKFZp762O1415)	
222115_x_at	-2.3		Similar to RIKEN cDNA 3930401K13 gene, clone IMAGE:3454556, mRNA, partial cds	
220509_at	-2.3		hypothetical protein PRO1777 (PRO1777)	PRO1777
221144_at	-2.3		hypothetical protein PRO1048 (PRO1048)	PRO1048
219895_at	-2.4		hypothetical protein FLJ20716 (FLJ20716)	FLJ20716
220096_at	-2.4		hypothetical protein FLJ20378 (FLJ20378)	FLJ20378
216745_x_at	-2.4		cDNA: FLJ20953 fis, clone ADSE01979	
220232_at	-2.5		hypothetical protein FLJ21032 (FLJ21032)	FLJ21032
216219_at	-2.5		mRNA; cDNA DKFZp434D2030 (from clone DKFZp434D2030); partial cds	
215028_at	-2.6		mRNA from chromosome 5q21-22, clone:FBR89	
201295_s_at	-2.7		DKFZP564A122 protein	DKFZP564A122
222320_at	-2.7		ESTs	
215515_at	-2.8		mRNA; cDNA DKFZp564G103 (from clone DKFZp564G103)	
220193_at	-3.6	-1.3	hypothetical protein FLJ22938 (FLJ22938)	FLJ22938
213872_at		5.5	hypothetical protein FLJ12619	FLJ12619
215220_s_at		4.3	Homo sapiens cDNA FLJ13049 fis, clone NT2RP3001428, highly similar to NUCLEOPROTEIN TPR.	
218147_s_at		3.9	Homo sapiens AD-017 protein (LOC55830), mRNA.	LOC55830
214985_at		3.8	Homo sapiens clone 24739 mRNA sequence.	
221595_at		3.6	mRNA; cDNA DKFZp564O0523 (from clone DKFZp564O0523)	
33148_at		3.4	Cluster Incl. AI459274:tk11f11.x1 Homo sapiens cDNA, 3 end /clone=IMAGE-2150733	
215029_at		3.3	mRNA; cDNA DKFZp586E2317 (from clone DKFZp586E2317)	
219279_at		2.8	hypothetical protein FLJ20220	FLJ20220
212220_at		2.6	KIAA0077 protein	KIAA0077
218195_at		2.6	hypothetical protein FLJ12910 (FLJ12910)	FLJ12910
209512_at	1.1	2.5	Similar to RIKEN cDNA 2610207I16 gene, clone MGC:10940	
219905_at		2.5	hypothetical protein PRO2801 (PRO2801)	PRO2801
218784_s_at		2.5	hypothetical protein FLJ11101 (FLJ11101)	FLJ11101
222180_at		2.4	cDNA FLJ14122 fis, clone MAMMA1002033	
204364_s_at		2.3	hypothetical protein FLJ13110	FLJ13110
212926_at		2.2	KIAA0594 protein	KIAA0594
204508_s_at		2.2	Homo sapiens, hypothetical protein FLJ20151, clone MGC:1073, mRNA, complete cds.	FLJ20151
216804_s_at		2.2	Homo sapiens cDNA: FLJ23564 fis, clone LNG10773	
221553_at		2.1	mRNA; cDNA DKFZp564K142 (from clone DKFZp564K142)	
206500_s_at		2.1	hypothetical protein FLJ11186 (FLJ11186)	FLJ11186
218167_at		2.1	hypothetical protein (LOC51321)	LOC51321
221263_s_at		2.1	hypothetical protein MGC3133 (MGC3133)	MGC3133
206652_at		2.1	hypothetical protein (HSPC050)	HSPC050
218640_s_at		2	hypothetical protein FLJ13187 (FLJ13187)	FLJ13187
206992_s_at		2	hypothetical protein (HSU79253)	HSU79253
207170_s_at		2	DKFZP586A011 protein (DKFZP586A011)	DKFZP586A011
217949_s_at		2	hypothetical protein IMAGE3455200 (IMAGE3455200)	IMAGE3455200
215252_at		2	Homo sapiens cDNA: FLJ21350 fis, clone COL02751	
202537_s_at		2	Homo sapiens CGI-84 protein mRNA, complete cds.	
214805_at		1.8	Human clone 23933 mRNA sequence	
221826_at		1.8	Homo sapiens cDNA FLJ12793 fis, clone NT2RP2002033	
217926_at		1.8	Homo sapiens HSPC023 protein (HSPC023), mRNA	HSPC023
219043_s_at		1.8	Homo sapiens hypothetical protein MGC3062 (MGC3062), mRNA	MGC3062
212634_at		1.8	KIAA0776 protein	KIAA0776
212979_s_at		1.8	Homo sapiens PRO2751 mRNA, complete cds	
217717_s_at		1.7	GW128 protein	GW128
218751_s_at		1.7	Homo sapiens hypothetical protein FLJ11071 (FLJ11071), mRNA	FLJ11071
219449_s_at		1.7	Homo sapiens hypothetical protein FLJ20533 (FLJ20533), mRNA	FLJ20533
203762_s_at		1.7	CGI-60 protein	LOC51626
202378_s_at		1.7	leptin receptor gene-related protein	HSOBRGRP
212451_at		1.6	KIAA0256 gene product	KIAA0256
218229_s_at		1.6	KIAA1513 protein (KIAA1513), mRNA	KIAA1513
201917_s_at		1.6	hypothetical protein FLJ10618	FLJ10618
218929_at		1.6	Homo sapiens hypothetical protein FLJ20036 (FLJ20036), mRNA	FLJ20036
212840_at		1.5	KIAA0794	KIAA0794
212720_at		1.5	CL25022 (hypothetical protein)	CL25022
201683_x_at		1.5	KIAA0737 gene product	KIAA0737
48808_at		1.5	IMAGE-1704443	
212638_s_at		1.5	Homo sapiens mRNA; cDNA DKFZp434D2111 (from clone DKFZp434D2111)	
214734_at		1.5	Homo sapiens mRNA for KIAA0624 protein, partial cds.	KIAA0624
212749_s_at		1.5	DKFZP586C1620 protein	DKFZP586C1620
215780_s_at		1.5	Human DNA sequence from PAC 30P20 on chromosome Xq21.1-Xq21.3. Contains set pseudogene, ESTs and STS	
208795_s_at		1.5	Human mRNA for P1cdc47, complete cds.	
220925_at		1.4	hypothetical protein FLJ21613 similar to ratcorneal wound healing related protein	FLJ21613
213272_s_at		1.4	hypothetical protein from clone 24796	LOC57146
218521_s_at		1.4	Homo sapiens hypothetical protein FLJ11011 (FLJ11011), mRNA	FLJ11011
220295_x_at		1.4	Homo sapiens hypothetical protein FLJ20354 (FLJ20354), mRNA	FLJ20354

216449_x_at		1.4	Homo sapiens cDNA: FLJ22209 fis, clone HRC01496	
218605_at		1.4	Homo sapiens hypothetical protein FLJ23182 (FLJ23182), mRNA	FLJ23182
219679_s_at		1.4	Homo sapiens hypothetical protein PRO1741 (PRO1741), mRNA	PRO1741
212195_at		1.4	Homo sapiens mRNA; cDNA DKFZp564F053 (from clone DKFZp564F053)	
202852_s_at		1.4	Homo sapiens hypothetical protein FLJ11506 (FLJ11506), mRNA	FLJ11506
201101_s_at		1.4	KIAA0164 gene product	KIAA0164
209669_s_at		1.3	Homo sapiens, Similar to DKFZP564M2423 protein, clone MGC:684, mRNA, complete cds	
201918_at		1.3	hypothetical protein FLJ10618	FLJ10618
214723_x_at		1.3	Homo sapiens mRNA for KIAA1641 protein, partial cds.	KIAA1641
212388_at		1.3	Homo sapiens mRNA for KIAA1057 protein, partial cds.	KIAA1057
218947_s_at		1.2	Homo sapiens hypothetical protein FLJ10486 (FLJ10486), mRNA	FLJ10486
212442_s_at		1.2	Homo sapiens cDNA: FLJ21238 fis, clone COL01115	
218449_at		1.2	Homo sapiens hypothetical protein FLJ11200 (FLJ11200), mRNA	FLJ11200
202386_s_at		1.2	Homo sapiens KIAA0430 gene product (KIAA0430), mRNA.	KIAA0430
219158_s_at		1.2	Homo sapiens hypothetical protein FLJ13340 (FLJ13340), mRNA	FLJ13340
212632_at		1.2	Homo sapiens clone 24889 mRNA sequence	
219858_s_at		1.2	Homo sapiens hypothetical protein FLJ20160 (FLJ20160), mRNA	FLJ20160
220083_x_at		1.2	CGI-70 protein	LOC51630
212655_at		1.1	Homo sapiens mRNA for KIAA0579 protein, partial cds.	KIAA0579
219387_at		1.1	Homo sapiens hypothetical protein (LOC55580), mRNA.	LOC55580
212096_s_at		1.1	Homo sapiens mRNA; cDNA DKFZp586D1519 (from clone DKFZp586D1519).	KIAA1288
212232_at		1.1	Homo sapiens mRNA for KIAA1014 protein, partial cds.	KIAA1014
212402_at		1.1	KIAA0853 protein	KIAA0853
219503_s_at		1.1	Homo sapiens hypothetical protein FLJ11036 (FLJ11036), mRNA	FLJ11036
214949_at		1.1	Homo sapiens mRNA; cDNA DKFZp586L141 (from clone DKFZp586L141)	
219496_at		1.1	Homo sapiens hypothetical protein FLJ21870 (FLJ21870), mRNA	FLJ21870
212644_s_at		1.1	Homo sapiens cDNA: FLJ21927 fis, clone HEP04178, highly similar to HSU90909 Human clone 23722 mRNA sequence	
212228_s_at		1.1	hypothetical protein DKFZp434K046	DKFZp434K046
213617_s_at		1.1	DKFZP586M1523 protein	DKFZP586M1523
212397_at		1.1	Homo sapiens mRNA; cDNA DKFZp434I0812 (from clone DKFZp434I0812); partial cds	
218108_at		1.1	Homo sapiens hypothetical protein FLJ10483 (FLJ10483), mRNA	FLJ10483
216101_at		1	Homo sapiens mRNA; cDNA DKFZp564B083 (from clone DKFZp564B083)	
221580_s_at		1	Homo sapiens, clone MGC:5306, mRNA, complete cds	
210463_x_at		1	Homo sapiens, hypothetical protein FLJ20244, clone MGC:1066, mRNA, complete cds	
202060_at		1	Homo sapiens KIAA0155 gene product (KIAA0155), mRNA	KIAA0155
202753_at		1	Homo sapiens KIAA0107 gene product (KIAA0107), mRNA.	KIAA0107
201083_s_at		1	KIAA0164 gene product	KIAA0164
209711_at		1	KIAA0260 protein	KIAA0260
219474_at		1	Homo sapiens hypothetical protein FLJ23186 (FLJ23186), mRNA	FLJ23186
215577_at		-1	Homo sapiens cDNA FLJ12000 fis, clone HEMBB1001531	HEMBB1001531
214749_s_at		-1	Homo sapiens cDNA FLJ20811 fis, clone ADSE01435.	FLJ20811
212323_s_at		-1	KIAA0453 protein	KIAA0453
32091_at		-1	Homo sapiens mRNA for KIAA0446 protein, complete cds	
212450_at		-1	Homo sapiens mRNA for KIAA0256 protein, partial cds.	KIAA0256
208851_s_at		-1	Homo sapiens mRNA; cDNA DKFZp761B15121 (from clone DKFZp761B15121); complete cds	
220708_at		-1	Homo sapiens hypothetical protein FLJ11370 (FLJ11370), mRNA	FLJ11370
58900_at		-1	IMAGE-990806	
218630_at		-1	Homo sapiens hypothetical protein FLJ20345 (FLJ20345), mRNA	FLJ20345
220079_s_at		-1	Homo sapiens hypothetical protein FLJ11328 (FLJ11328), mRNA	FLJ11328
218394_at		-1	Homo sapiens hypothetical protein FLJ22386 (FLJ22386), mRNA	FLJ22386
207283_at		-1	Homo sapiens hypothetical protein DKFZp547I014 (DKFZp547I014), mRNA	DKFZp547I014
218020_s_at		-1	Homo sapiens hypothetical protein FLJ13222 (FLJ13222), mRNA	FLJ13222
54632_at		-1	IMAGE-1855342	
46665_at		-1	IMAGE-2470926	
64064_at		-1	IMAGE-2126397	
53912_at		-1	IMAGE-301963	
217824_at		-1	CGI-76 protein	LOC51632
213625_at		-1	hypothetical protein P1 p373c6	
40225_at		-1	Homo sapiens mRNA for HsGAK, complete cds	
212946_at	-1.1	-1.1	Homo sapiens cDNA: FLJ21779 fis, clone HEP00210.	KIAA0564
212124_at		-1.1	Homo sapiens clone 24800 mRNA sequence.	KIAA1224
36129_at		-1.1	Homo sapiens KIAA0397 mRNA, complete cds	
32259_at		-1.1	Human mRNA for KIAA0388 gene, complete cds	
217719_at		-1.1	Homo sapiens HSPC025 (HSPC025), mRNA.	HSPC025
203906_at		-1.1	KIAA0763 gene product	KIAA0763
213547_at		-1.1	Homo sapiens mRNA for KIAA0667 protein, partial cds.	KIAA0667
203169_at		-1.1	Homo sapiens KIAA0258 gene product (KIAA0258), mRNA.	KIAA0258
AFFX-hum_alu_at		-1.1	Human Alu-Sq subfamily consensus sequence.	
221569_at		-1.1	Homo sapiens mRNA; cDNA DKFZp434N031 (from clone DKFZp434N031); complete cds	
220953_s_at		-1.1	Homo sapiens hypothetical protein (FLJ20476), mRNA	FLJ20476
219501_at		-1.1	Homo sapiens hypothetical protein FLJ10094 (FLJ10094), mRNA	FLJ10094
63009_at		-1.1	IMAGE-1723132	
53076_at		-1.1	IMAGE-1664324	
55081_at		-1.1	IMAGE-323922	
59697_at		-1.1	IMAGE-1090172	
218632_at		-1.1	Homo sapiens hypothetical protein FLJ21156 (FLJ21156), mRNA	FLJ21156
218637_at		-1.1	hypothetical protein IMPACT	IMPACT
60471_at		-1.1	IMAGE-1047339	
33323_r_at		-1.1	Cluster Incl. X57348:H.sapiens mRNA (clone 9112)	
33322_i_at		-1.1	Cluster Incl. X57348:H.sapiens mRNA (clone 9112)	
218771_at		-1.1	Homo sapiens hypothetical protein FLJ10782 (FLJ10782), mRNA	FLJ10782
65635_at		-1.1	DKFZp434M1928	
218916_at		-1.1	Homo sapiens hypothetical protein FLJ23436 (FLJ23436), mRNA	FLJ23436
49327_at		-1.1	IMAGE-2124784	
47571_at		-1.1	IMAGE-429176	
218902_at		-1.2	Homo sapiens hypothetical protein FLJ20005 (FLJ20005), mRNA	FLJ20005
212052_s_at		-1.2	Homo sapiens mRNA for KIAA0676 protein, partial cds.	KIAA0676

206507_at		-1.2	Homo sapiens KIAA0426 gene product (KIAA0426), mRNA	KIAA0426
203355_s_at		-1.2	Homo sapiens KIAA0942 protein (KIAA0942), mRNA.	KIAA0942
213196_at		-1.2	KIAA0326 protein	KIAA0326
208276_at		-1.2	Homo sapiens clone FLB7343 (LOC51221), mRNA.	LOC51221
212395_s_at		-1.2	KIAA0090 protein	KIAA0090
205250_s_at		-1.2	Homo sapiens KIAA0373 gene product (KIAA0373), mRNA.	KIAA0373
202572_s_at	-1.1	-1.2	Homo sapiens KIAA0964 protein (KIAA0964), mRNA.	KIAA0964
45572_s_at		-1.2	IMAGE-2504821	
222099_s_at		-1.2	DKFZP434D1335 protein	DKFZP434D1335
213004_at		-1.2	Homo sapiens clone 23767 and 23782 mRNA sequences	
57532_at		-1.2	IMAGE-2712427	
215154_at		-1.2	Homo sapiens mRNA; cDNA DKFZp434G043 (from clone DKFZp434G043)	
214052_x_at		-1.2	KIAA1096 protein	KIAA1096
213393_at		-1.2	Human clone 23908 mRNA sequence	
51158_at		-1.2	IMAGE-2270985	
218010_x_at		-1.2	Homo sapiens hypothetical protein MGC2479 (MGC2479), mRNA	MGC2479
213083_at		-1.2	Homo sapiens mRNA for putative Sqv-7-like protein, partial.	SQV7L
214744_s_at		-1.2	Homo sapiens cDNA FLJ11898 fis, clone HEMBA1007322	
57739_at		-1.2	IMAGE-2473332	
219765_at		-1.2	Homo sapiens hypothetical protein FLJ12586 (FLJ12586), mRNA	FLJ12586
216288_at		-1.2	Homo sapiens cDNA FLJ13867 fis, clone THYRO1001262	
60528_at		-1.2	IMAGE-299589	
212970_at		-1.2	Homo sapiens mRNA; cDNA DKFZp434E033 (from clone DKFZp434E033)	
50376_at		-1.2	IMAGE-1891867	
210360_s_at		-1.2	Homo sapiens PRO1941 mRNA, complete cds.	KIAA0429
213085_s_at		-1.3	Homo sapiens mRNA for KIAA0869 protein, partial cds.	KIAA0869
221621_at		-1.3	Homo sapiens clone FLB3442 PRO0872 mRNA, complete cds.	
213628_at		-1.3	KIAA0761 protein	KIAA0761
212353_at	-1.9	-1.3	KIAA1077 protein	KIAA1077
215766_at		-1.3	Homo sapiens mRNA; cDNA DKFZp434D044 (from clone DKFZp434D044)	
221767_x_at		-1.3	hypothetical protein PRO2900	PRO2900
36552_at		-1.3	Homo sapiens mRNA; cDNA DKFZp586P0123 (from clone DKFZp586P0123)	
50277_at		-1.3	IMAGE-990785	
218776_s_at		-1.3	Homo sapiens hypothetical protein FLJ23375 (FLJ23375), mRNA	FLJ23375
214798_at		-1.3	Homo sapiens cDNA: FLJ21771 fis, clone COLF7779	
44783_s_at		-1.3	IMAGE-37665	
213904_at		-1.3	Homo sapiens mRNA; cDNA DKFZp547E184 (from clone DKFZp547E184)	
45749_at		-1.3	IMAGE-743210	
217481_x_at		-1.3	Homo sapiens mRNA; cDNA DKFZp586F1622 (from clone DKFZp586F1622)	
52255_s_at		-1.3	IMAGE-2562160	
213485_s_at		-1.3	Homo sapiens mRNA for FLJ00002 protein, partial cds	
217335_at		-1.3	Homo sapiens cDNA FLJ13477 fis, clone PLACE1003638	FLJ11292
215903_s_at		-1.4	KIAA0807 protein	KIAA0807
212755_at		-1.4	KIAA1040 protein	KIAA1040
219906_at		-1.4	Homo sapiens hypothetical protein FLJ10213 (FLJ10213), mRNA	FLJ10213
65718_at		-1.4	IMAGE-2243340	
219874_at		-1.4	Homo sapiens hypothetical protein FLJ23188 (FLJ23188), mRNA	FLJ23188
220390_at		-1.4	Homo sapiens hypothetical protein FLJ23598 (FLJ23598), mRNA	FLJ23598
222116_s_at		-1.4	Homo sapiens mRNA; cDNA DKFZp762O207 (from clone DKFZp762O207).	FLJ20748
218687_s_at		-1.4	Homo sapiens hypothetical protein FLJ20063 (FLJ20063), mRNA	FLJ20063
212281_s_at		-1.4	hypothetical protein	MAC30
212447_at		-1.4	Homo sapiens HSPC284 mRNA, partial cds.	DKFZP566C134
209794_at		-1.5	Homo sapiens KIAA0411 mRNA, complete cds.	KIAA0411
213204_at		-1.5	Homo sapiens mRNA for KIAA0708 protein, partial cds.	KIAA0708
204793_at		-1.5	Homo sapiens KIAA0443 gene product (KIAA0443), mRNA.	KIAA0443
215034_s_at		-1.5	Homo sapiens cDNA FLJ13302 fis, clone OVARC1001357	
56256_at		-1.5	IMAGE-491573	
212991_at		-1.5	Homo sapiens mRNA; cDNA DKFZp434C0118 (from clone DKFZp434C0118); partial cds	
220456_at		-1.5	Homo sapiens hypothetical protein FLJ11112 (FLJ11112), mRNA	FLJ11112
215235_at		-1.5	Homo sapiens mRNA; cDNA DKFZp564P0562 (from clone DKFZp564P0562); partial cds	
213810_s_at		-1.5	hypothetical protein FLJ10342	FLJ10342
221995_s_at		-1.5	hypothetical protein MGC3243	MGC3243
212473_s_at		-1.5	Homo sapiens cDNA: FLJ22463 fis, clone HRC10126	
219805_at		-1.5	Homo sapiens hypothetical protein FLJ22965 (FLJ22965), mRNA	FLJ22965
44654_at		-1.5	IMAGE-2314928	
205434_s_at		-1.6	KIAA1048 protein	KIAA1048
36030_at		-1.6	Homo sapiens mRNA; cDNA DKFZp586I2223 (from clone DKFZp586I2223)	
218517_at		-1.6	Homo sapiens hypothetical protein FLJ22479 (FLJ22479), mRNA	FLJ22479
54970_at		-1.6	IMAGE-1408473	
219525_at		-1.6	Homo sapiens hypothetical protein FLJ10847 (FLJ10847), mRNA	FLJ10847
204353_s_at		-1.6	Homo sapiens, clone MGC:10280, mRNA, complete cds	DKFZP586D211
43427_at		-1.6	IMAGE-2488324	
209459_s_at		-1.6	Homo sapiens NPD009 mRNA, complete cds.	NPD009
215461_at		-1.6	Homo sapiens chromosome 19, cosmid R31343	
210703_at		-1.7	Homo sapiens PRO2259 mRNA, complete cds.	
49452_at		-1.7	IMAGE-1667483	
217490_at		-1.7	Homo sapiens mRNA; cDNA DKFZp564P073 (from clone DKFZp564P073)	
212662_at		-1.7	Homo sapiens cDNA: FLJ22296 fis, clone HRC04468	
214725_at		-1.7	Homo sapiens mRNA; cDNA DKFZp564O1262 (from clone DKFZp564O1262)	
212980_at		-1.7	Homo sapiens mRNA; cDNA DKFZp586J101 (from clone DKFZp586J101)	
216142_at		-1.7	Homo sapiens mRNA; cDNA DKFZp434L092 (from clone DKFZp434L092)	
218330_s_at		-1.7	Homo sapiens hypothetical protein FLJ10633 (FLJ10633), mRNA	FLJ10633
214983_at		-1.7	Homo sapiens mRNA; cDNA DKFZp434I143 (from clone DKFZp434I143).	DKFZP434I143
40016_g_at		-1.8	Human mRNA for KIAA0303 gene, partial cds	
215525_at		-1.8	Homo sapiens mRNA; cDNA DKFZp586A0423 (from clone DKFZp586A0423).	
213316_at		-1.8	Homo sapiens mRNA; cDNA DKFZp586L0120 (from clone DKFZp586L0120)	
208964_s_at		-1.8	Homo sapiens mRNA; cDNA DKFZp762M2311 (from clone DKFZp762M2311); complete cds	
214719_at		-1.8	Homo sapiens cDNA: FLJ23067 fis, clone LNG04993	

213924_at		-1.8	hypothetical protein FLJ11585	FLJ11585
213422_s_at		-1.9	Homo sapiens mRNA; cDNA DKFZp586E2023 (from clone DKFZp586E2023)	
218651_s_at		-1.9	Homo sapiens hypothetical protein FLJ11196 (FLJ11196), mRNA	FLJ11196
217132_at		-1.9	Homo sapiens clone 24587 mRNA sequence.	
212494_at		-2	Homo sapiens mRNA for KIAA1075 protein, partial cds.	KIAA1075
213836_s_at	-1.1	-2	KIAA1001 protein	KIAA1001
37547_at		-2	Human clone IMAGE-22181 unknown protein mRNA, partial cds	
39549_at		-2	IMAGE-2372060	
215314_at		-2	Homo sapiens cDNA FLJ10270 fis, clone HEMBB1001096	
206048_at		-2.1	Homo sapiens putative zinc finger protein from EUROIMAGE 566589 (LOC58495), mRNA.	LOC58495
220376_at		-2.4	Homo sapiens hypothetical protein FLJ21302 (FLJ21302), mRNA	FLJ21302
214951_at		-2.5	Homo sapiens mRNA; cDNA DKFZp564M1916 (from clone DKFZp564M1916); partial cds	
222078_at		-2.7	KIAA1535 protein	KIAA1535
56748_at		-2.7	Cluster Incl. X90539:HSGT483 Homo sapiens cDNA	
220615_s_at		-2.8	Homo sapiens hypothetical protein FLJ10462 (FLJ10462), mRNA	FLJ10462
218899_s_at		-3.3	Homo sapiens hypothetical protein FLJ12015 (FLJ12015), mRNA	FLJ12015
216415_at		-3.5	Homo sapiens cDNA: FLJ23140 fis, clone LNG09065	
219619_at		-3.6	Homo sapiens hypothetical protein DKFZp761C07121 (DKFZp761C07121), mRNA	
213609_s_at		-3.8	Homo sapiens mRNA for KIAA0927 protein, partial cds.	KIAA0927
219973_at		-5.3	Homo sapiens hypothetical protein FLJ23548 (FLJ23548), mRNA	FLJ23548
217959_s_at		2.3	Homo sapiens PTD009 protein	PTD009
218728_s_at		1.3	Homo sapiens HSPC163 protein (HSPC163), mRNA.	HSPC163
217907_at	1.1		Homo sapiens HSPC071 protein (HSPC071), mRNA	HSPC071
216347_s_at		-1.2	Homo sapiens cDNA FLJ13126 fis, clone NT2RP3002909, weakly similar to P53-BINDING PROTEIN 2.	KIAA0771 protein
220940_at	-1.1		Homo sapiens KIAA1641 protein (KIAA1641), mRNA. /hypothetical protein FLJ21281	KIAA1641
204403_x_at	-1.4		Homo sapiens KIAA0738 gene product (KIAA0738), mRNA.	KIAA0738
218631_at	-1		Homo sapiens hypothetical protein PP5395 (PP5395), mRNA.	PP5395
212956_at	-1.2		KIAA0882 protein	KIAA0882
206169_x_at	-1.3		Homo sapiens KIAA1031 protein (KIAA1031), mRNA.	KIAA1031
209883_at	-1.4		Homo sapiens C1orf17 mRNA, complete cds.	KIAA0584
205096_at	-1		Homo sapiens KIAA0618 gene product (KIAA0618), mRNA.	KIAA0618
211454_x_at	-1.6	-1.3	Homo sapiens FKSG51 (FKSG51) mRNA, complete cds	FKSG51
214034_at	-1.7		Homo sapiens mRNA for KIAA0525 protein, partial cds.	KIAA0525

List of the genes that are differentially expressed in $\alpha6^{+}$/MHCI^{-} cells and in $\alpha6^{+}$/MHCI^{+} cells. Entire Affymetrix probe set and their annotated genes that are up-regulated ≥ 2-fold in either $\alpha6^{+}$/MHCI^{-} cells or $\alpha6^{+}$/MHCI^{+} cells sorted according to their functions. Some of the genes are involved in multiple processes in the cell and could be placed in several tables. The table shows the difference in the expression $\alpha6^{+}$/MHCI^{+} cells vs. $\alpha6^{+}$/MHCI^{-} cells. "–"sign indicates that the gene is upregulated in $\alpha6^{+}$/MHCI^{-} cells. The numbers that show the difference in the level of gene expression are in log2 scale.
