# Supplementary material for: Transcriptional profiling of putative human epithelial stem cells
Source: BMC Genomics. 2008 Jul 30;9:359. doi: 10.1186/1471-2164-9-359 (PMC2536675; doi:10.1186/1471-2164-9-359)
Supplement: Additional file 2 — List of the entire genes that are differentially expressed in either α6+/MHCI+ cells or α6+/MHCI- cells and are consistently upregulated or down regulated in both arrays. "-"sign indicates that the gene is upregulated in α6+/MHCI- cells. The numbers that show the difference in the level of gene expression are in log2 scale. [file 1471-2164-9-359-S2.pdf]

Probe Set	Exp. 1 Signal Log Ratio	Exp. 2 Signal Log Ratio	Description	Gene Symbol
1053_at	1.3	0.8	Human replication factor C, 40-kDa subunit (A1) mRNA, complete cds	
200041_s_at	1.7	1	HLA-B associated transcript-1	D6S81E
200072_s_at	0.6	1.3	M4 protein deletion mutant	HNRPM
200079_s_at	0.6	0.3	lysyl-tRNA synthetase	KARS
200593_s_at	0.8	0.1	heterogeneous nuclear ribonucleoprotein U(scaffold attachment factor A)	HNRPU
200602_at	-0.6	-1.2	amyloid beta (A4) precursor protein (proteasenexin-II, Alzheimer disease)	APP
200613_at	0.5	1.3	adaptor-related protein complex 2, mu 1 subunit	AP2M1
200638_s_at	0.3	2.2	tyrosine 3-monooxygenasetryptophan5-monooxygenase activation protein, zeta polypeptide	YWHAZ
200644_at	-1.8	-1	macrophage myristoylated alanine-rich C kinasesubstrate	MACMARCKS
200662_s_at	0.4	0.7	translocase of outer mitochondrial membrane 20(yeast) homolog	KIAA0016
200669_s_at	0.6	0.5	ubiquitin-conjugating enzyme E2D 3 (homologousto yeast UBC45)	UBE2D3
200685_at	0.3	0.1	splicing factor, arginineserine-rich 11	SFRS11
200692_s_at	1.3	2.7	heat shock 70kD protein 9B (mortalin-2)	HSPA9B
200693_at	0.4	0.4	tyrosine 3-monooxygenasetryptophan5-monooxygenase activation protein, theta polypeptide	YWHAQ
200728_at	0.7	2	ARP2 (actin-related protein 2, yeast) homolog	ACTR2
200737_at	0.7	0.8	phosphoglycerate kinase 1	PGK1
200745_s_at	0.7	0.4	beta-subunit signal transducing proteins GSGI	GNB1
200750_s_at	0.9	3.5	GTP binding protein	RAN
200751_s_at	0.9	1.7	heterogeneous nuclear ribonucleoprotein C (C1C2)	HNRPC
200760_s_at	0.9	1.2	vitamin A responsive; cytoskeleton related	JWA
200762_at	-1.9	-1.9	dihydropyrimidinase-like 2	DPYSL2
200806_s_at	1.8	1.7	heat shock 60kD protein 1 (chaperonin)	HSPD1
200814_at	0.4	1	proteasome (prosome, macropain) activatorsubunit 1 (PA28 alpha)	PSME1
200818_at	0.5	1.2	ATP synthase, H+ transporting, mitochondrial F1complex, O subunit (oligomycin sensitivity conferringprotein)	ATP5O
200842_s_at	0.5	0.6	glutamyl-prolyl-tRNA synthetase	EPRS
200843_s_at	0.3	0.1	glutamyl-prolyl tRNA synthetase	EPRS
200844_s_at	0.7	0.1	anti-oxidant protein 2 (non-selenium glutathione peroxidase, acidic calcium-independent phospholipase A2)	KIAA0106
200848_at	0.9	1.2	S-adenosylhomocysteine hydrolase-like 1	AHCYL1
200853_at	0.8	1.3	H2A histone family, member Z	H2AFZ
200863_s_at	0.7	0.4	RAB11A, member RAS oncogene family /FL=gb:NM_004663.1 gb:AF000231.1	RAB11A
200876_s_at	0.5	3.1	proteasome (prosome, macropain) subunit, betatype, 1	PSMB1
200878_at	1.2	0.5	endothelial PAS domain protein 1	EPAS1
200881_s_at	0.8	0.7	heat shock protein, DNAJ-like 2	HSJ2
200883_at	1.4	1	ubiquinol-cytochrome c reductase core proteinII	UQCRC2
200900_s_at	2.8	1.4	mannose-6-phosphate receptor (cation dependent)	M6PR
200904_at	0.6	1.6	HLA-E	HLA-E
200910_at	0.3	0.9	chaperonin containing TCP1, subunit 3 (gamma)	CCT3
200920_s_at	0.6	3.5	B-cell translocation gene 1, anti-proliferative	BTG1
200927_s_at	0.4	0.9	GTPase Rab14	Rab14
200978_at	0.8	1.8	malate dehydrogenase 1, NAD (soluble)	MDH1
200993_at	1	1.2	RAN binding protein 7	RANBP7
200996_at	0.5	1.8	ARP3 (actin-related protein 3, yeast) homolog	ACTR3
201001_s_at	1.2	0.2	ubiquitin-conjugating enzyme E2 variant 1	UBE2V1
201014_s_at	1.4	2	multifunctional polypeptide similar to SAICARsynthetase and AIR carboxylase	ADE2H1
201027_s_at	1.3	0.5	translation initiation factor IF2	IF2
201030_x_at	0.6	1	lactate dehydrogenase B	LDHB
201051_at	0.8	0.7	putative human HLA class II associated protein I	PHAP1
201067_at	1	0.3	proteasome (prosome, macropain) 26S subunit, ATPase, 2	PSMC2
201068_s_at	1	1	proteasome (prosome, macropain) 26S subunit,ATPase, 2	PSMC2
201110_s_at	-2.3	-1.9	thrombospondin 1	THBS1
201112_s_at	1.1	2.1	chromosome segregation 1 (yeast homolog)-like	CSE1L
201117_s_at	-1.9	-1.3	carboxypeptidase E precursor	CPE
201144_s_at	1	0.8	eukaryotic translation initiation factor 2,subunit 1 (alpha, 35kD)	EIF2S1
201163_s_at	-2.2	-1.6	insulin-like growth factor binding protein 7	IGFBP7
201176_s_at	0.6	2.5	archain	ARCN1
201179_s_at	0.5	0.5	guanine nucleotide binding protein (G protein), alpha inhibiting activity polypeptide 3	GNAI1
201196_s_at	0.8	0.4	S-adenosylmethionine decarboxylase 1	AMD2
201198_s_at	1.2	1	proteasome (prosome, macropain) 26S subunit, non-ATPase, 1	PSMD1
201202_at	0.5	0.6	proliferating cell nuclear antigen	PCNA
201214_s_at	0.6	1.4	protein phosphatase 1, regulatory subunit 7	PPP1R7
201237_at	0.5	0.2	capping protein (actin filament) muscle Z-line, alpha 2	CAPZA2
201241_at	0.5	0.6	DEADH (Asp-Glu-Ala-AspHis) box polypeptide 1	DDX1
201258_at	0.6	2.4	ribosomal protein S16	RPS16

201263_at	0.4	1	threonyl-tRNA synthetase	TARS
201274_at	0.4	0.6	proteasome (prosome, macropain) subunit, alphatype, 5	PSMA5
201291_s_at	4.5	2.1	topoisomerase (DNA) II alpha (170kD)	TOP2A
201292_at	2	2.1	topoisomerase (DNA) II alpha (170kD)	TOP2A
201301_s_at	0.4	0.5	annexin A4	ANXA4
201303_at	0.8	0.9	KIAA0111 gene product	KIAA0111
201317_s_at	0.7	0.9	proteasome (prosome, macropain) subunit, alphatype, 2	PSMA2
201322_at	0.3	1	ATP synthase, H+ transporting, mitochondrial F1complex, beta polypeptide	ATP5B
201325_s_at	0.4	2.1	epithelial membrane protein 1	EMP1
201328_at	0.9	0.5	v-ets avian erythroblastosis virus E26 oncogene homolog 2	ETS2
201330_at	0.4	0.8	arginyl-tRNA synthetase	RARS
201358_s_at	-0.6	-1.2	coatomer protein complex, subunit beta	COPB
201416_at	-0.9	-1.2	SRY (sex determining region Y)-box 4	SOX4
201441_at	0.4	0.5	cytochrome c oxidase subunit VIb	COX6B
201444_s_at	0.6	0.8	ATPase, H+ transporting, lysosomal (vacuolarproton pump) membrane sector associated protein M8-9	APT6M8-9
201454_s_at	0.3	1.4	aminopeptidase puromycin sensitive	NPEPPS
201457_x_at	0.5	1.3	kinetochore protein BUB3	BUB3
201462_at	0.5	1.7	KIAA0193 gene product	KIAA0193
201477_s_at	1	0.3	ribonucleotide reductase M1 polypeptide	RRM1
201487_at	0.9	0.6	cathepsin C	CTSC
201502_s_at	0.4	0.2	nuclear factor of kappa light polypeptide gene enhancer in B-cells inhibitor, alpha	NFKBIA
201506_at	1.2	1.3	transforming growth factor, beta-induced, 68kD	TGFBI
201532_at	0.8	1.8	proteasome (prosome, macropain) subunit, alphatype, 3	PSMA3
201552_at	0.3	1.9	lysosomal-associated membrane protein 1	LAMP1
201577_at	1.2	1.2	Homo sapiens non-metastatic cells 1, protein (NM23A) expressed in (NME1), mRNA. / non-metastatic cells 1 protein	NME1
201593_s_at	0.8	1.4	uncharacterized hypothalamus protein HT010	HT010
201606_s_at	1.3	0.7	nuclear phosphoprotein similar to S. cerevisiae PWP1	PWP1
201608_s_at	0.6	0.1	nuclear phosphoprotein similar to S. cerevisiae PWP1	PWP1
201659_s_at	0.9	1.1	ADP-ribosylation factor-like 1	ARL1
201663_s_at	1.4	0.2	chromosome-associated polypeptide C	CAP-C
201664_at	0.8	0.5	hypothetical protein	DKFZp434F205
201672_s_at	0.5	0.3	ubiquitin specific protease 14 (tRNA-guaninetransglycosylase)	USP14
201694_s_at	-0.8	-1.3	early growth response 1	EGR1
201725_at	1.4	1.1	D123 gene product	D123
201756_at	0.6	0.1	replication protein A2 (32kD)	RPA2
201758_at	0.3	0.9	tumor susceptibility gene 101	TSG101
201779_s_at	0.8	1.2	RING zinc finger protein RZF	RNF13
201811_x_at	-1	-1.2	SH3-domain binding protein 5 (BTK-associated)	SH3BP5
201827_at	0.7	0.9	PRO2451	SMARCD2
201831_s_at	3.1	4.4	vesicle docking protein p115	P115
201842_s_at	0.2	0.4	EGF-containing fibulin-like extracellular matrix protein 1	EFEMP1
201859_at	-1.6	-1.1	proteoglycan 1, secretory granule	PRG1
201890_at	1.1	0.7	ribonucleotide reductase M2 polypeptide	RRM2
201897_s_at	1.1	1.6	CDC28 protein kinase 1	CKS1
201903_at	0.8	0.8	ubiquinol-cytochrome c reductase core protein I	UQCRC1
201906_s_at	0.4	0.1	HYA22 protein	HYA22
201912_s_at	0.4	0.1	G1 to S phase transition 1	GSPT1
201927_s_at	0.5	4.2	plakophilin 4	PKP4
201931_at	0.8	1.9	electron transfer flavoprotein, alphapolypeptide	ETFA
201946_s_at	1.2	2.6	chaperonin containing TCP1, subunit 2 (beta)	CCT2
201947_s_at	0.9	0.3	chaperonin containing TCP1, subunit 2 (beta)	CCT2
201970_s_at	1.7	2.9	nuclear autoantigenic sperm protein(histone-binding)	NASP
201975_at	-0.7	-1	restin (Reed-Steinberg cell-expressedintermediate filament-associated protein)	RSN
201999_s_at	0.6	0.9	t-complex-associated-testis-expressed 1-like 1	TCTEL1
202028_s_at	-0.6	-1.4	ribosomal protein L38	RPL38
202040_s_at	0.5	0.1	retinoblastoma-binding protein 2	RBBP2
202042_at	0.6	0.4	histidyl tRNA synthetase	HARS
202080_s_at	0.6	0.3	KIAA1042 protein	KIAA1042
202084_s_at	0.8	0.7	SEC14 (S. cerevisiae)-like 1	SEC14L1
202107_s_at	0.8	0.4	minichromosome maintenance deficient (S.cerevisiae) 2 (mitotin)	MCM2
202113_s_at	0.9	3.1	sorting nexin 2	SNX2
202149_at	-0.7	-1.2	enhancer of filamentation 1 (cas-like docking; Crk-associated substrate related)	HEF1
202154_x_at	0.2	0.8	tubulin, beta, 4	TUBB4
202157_s_at	-1	-1.2	RNA-binding protein BRUNOL3	BRUNOL3
202168_at	0.3	0.9	TATA box binding protein (TBP)-associatedfactor, RNA polymerase II, G, 32kD	TAF2G
202174_s_at	0.4	0.5	pericentriolar material 1	PCM1
202199_s_at	-0.7	-0.6	SFRS protein kinase 1	SRPK1
202202_s_at	-0.9	-1.4	laminin, alpha 4 precursor	LAMA4

202206_at	0.5	1.6	ADP-ribosylation factor-like 7	ARL7
202209_at	1.1	0.8	Lsm3 protein	LSM3
202225_at	0.4	0.9	v-crk avian sarcoma virus CT10 oncogene homolog	CRK
202243_s_at	0.8	2.4	proteasome (prosome, macropain) subunit, betatype, 4	PSMB4
202263_at	0.4	0.3	cytochrome b5 reductase 1 (B5R.1)	LOC51706
202299_s_at	0.3	0.1	hepatitis B virus x-interacting protein	XIP
202300_at	0.5	1.9	hepatitis B virus x-interacting protein	XIP
202347_s_at	0.5	0.7	E2 ubiquitin-conjugating enzyme	LIG
202352_s_at	0.6	0.5	proteasome (prosome, macropain) 26S subunit, non-ATPase, 12	PSMD12
202378_s_at	0.9	1.7	leptin receptor gene-related protein	HSOBRGRP
202403_s_at	-1.3	-2	collagen, type I, alpha 2	COL1A2
202436_s_at	-2.2	-1.2	cytochrome P450, subfamily I (dioxin-inducible), polypeptide 1 (glaucoma 3, primary infantile)	CYP1B1
202437_s_at	-3.5	-2.7	cytochrome P450, subfamily I (dioxin-inducible),polypeptide 1	CYP1B1
202441_at	0.2	0.2	similar to Caenorhabditis elegans protein C42C1.9	KEO4
202446_s_at	0.5	0.7	phospholipid scramblase 1	PLSCR1
202487_s_at	1	0.7	purine-rich element binding protein B	PURB
202499_s_at	-0.8	-1	solute carrier family 2 (facilitated glucosetransporter), member 3	SLC2A3
202503_s_at	0.9	0.5	KIAA0101 gene product	KIAA0101
202546_at	1	3.2	vesicle-associated membrane protein 8	VAMP8
202548_s_at	0.4	0.7	PAK-interacting exchange factor beta	P85SPR
202552_s_at	-0.8	-1.3	cysteine-rich motor neuron 1	CRIM1
202554_s_at	0.6	1.3	glutathione S-transferase M3 (brain)	GSTM3
202572_s_at	-1.1	-1.2	KIAA0964 protein	KIAA0964
202589_at	1	0.4	thymidylate synthetase	TYMS
202591_s_at	0.6	1.2	single-stranded DNA-binding protein	SSBP
202594_at	0.4	0.5	MY047 protein	MY047
202596_at	1	0.2	endosulfine alpha	ENSA
202655_at	0.5	0.2	arginine-rich protein	ARMET
202659_at	0.6	0.9	proteasome (prosome, macropain) subunit, betatype, 10	PSMB10
202686_s_at	-0.8	-1.6	AXL receptor tyrosine kinase isoform 1precursor	AXL
202687_s_at	0.6	1	Apo-2 ligand	TNFSF10
202688_at	0.4	2.7	tumor necrosis factor (ligand) superfamily,member 10	TNFSF10
202697_at	0.8	0.6	cleavage and polyadenylation specific factor 5,25 kD subunit	CPSF5
202705_at	1.7	0.6	cyclin B2	CCNB2
202706_s_at	0.5	0.3	UMP synthase	UMPS
202712_s_at	1	0.8	ubiquitous mitochondrial creatine kinaseprecursor	CKMT1
202729_s_at	-0.8	-1.1	latent transforming growth factor beta bindingprotein 1 precursor	LTBP1
202737_s_at	0.5	0.4	U6 snRNA-associated Sm-like protein	LSM4
202746_at	-1.2	-0.8	integral membrane protein 2A	ITM2A
202753_at	0.5	1	KIAA0107 gene product	KIAA0107
202794_at	0.7	0.5	inositol polyphosphate-1-phosphatase	INPP1
202852_s_at	0.4	1.4	hypothetical protein FLJ11506	FLJ11506
202854_at	0.5	0.2	hypoxanthine phosphoribosyltransferase 1	HPRT1
202859_x_at	0.6	0.3	interleukin 8	IL8
202876_s_at	-0.8	-0.9	pre-B-cell leukemia transcription factor 2	PBX2
202899_s_at	0.9	1.7	splicing factor, arginineserine-rich 3	SFRS3
202900_s_at	0.9	0.8	nucleoporin 88kD	NUP88
202908_at	-1.1	-1.2	Wolfram syndrome protein	WFS1
202911_at	0.6	0.7	mutS (E. coli) homolog 6	MSH6
202923_s_at	0.7	0.1	glutamate-cysteine ligase	GCLC
202936_s_at	-1.1	-0.9	transcription factor SOX9	SOX9
202941_at	1	0.1	NADH dehydrogenase (ubiquinone) flavoprotein 2(24kD)	NDUFV2
202990_at	0.9	0.6	phosphorylase, glycogen; liver (Hers disease,glycogen storage disease type VI)	PYGL
203039_s_at	1.1	0.3	NADH dehydrogenase (ubiquinone) Fe-S protein 1(75kD) (NADH-coenzyme Q reductase)	NDUFS1
203074_at	0.8	0.7	annexin VIII	ANXA8
203098_at	0.4	0.2	hypothetical protein	DKFZp586C1622
203103_s_at	1.1	1.6	nuclear matrix protein NMP200 related tosplicing factor PRP19	NMP200
203105_s_at	1.4	0.5	dynamin 1-like protein, isoform 1	DNM1L
203126_at	0.8	1.1	inositol(myo)-1(or 4)-monophosphatase 2	IMPA2
203202_at	0.5	0.6	HIV-1 rev binding protein 2	HRB2
203203_s_at	1.1	1.4	HIV-1 rev binding protein 2	HRB2
203213_at	1.7	1.5	cell division cycle 2, G1 to S and G2 to M	CDC2
203247_s_at	0.4	0.4	zinc finger protein 24 (KOX 17)	ZNF24
203255_at	-0.3	-1.2	vitiligo-associated protein VIT-1	VIT1
203259_s_at	0.3	0.8	Similar to CGI-130 protein	LOC51020
203336_s_at	0.8	0.3	integrin cytoplasmic domain-associated protein 1	ICAP-1A
203341_at	0.4	0.6	CCAAT-box-binding transcription factor	CBF2
203345_s_at	1.3	1.7	putative DNA binding protein	M96
203358_s_at	0.8	0.2	enhancer of zeste (Drosophila) homolog 2	EZH2
203359_s_at	0.6	0.1	c-myc binding protein	MYCBP

203362_s_at	1	0.8	MAD2-like 1	MAD2L1
203405_at	0.9	0.9	Down syndrome critical region protein 2	DSCR2
203428_s_at	0.8	1.2	Homo sapiens mRNA for CIA, complete cds.	DKFZP547E2110
203455_s_at	-0.6	-0.9	spermidinespermine N1-acetyltransferase	SAT
203460_s_at	0.5	0.7	presenilin 1 isoform I-463	PSEN1
203517_at	0.3	1	metaxin 2	MTX2
203554_x_at	1.1	1	pituitary tumor-transforming protein 1	PTTG1
203560_at	0.8	1.2	gamma-glutamyl hydrolase (conjugase,folylpolygammaglutamyl hydrolase) precursor	GGH
203583_at	0.5	0.3	DKFZP564G0222 protein	DKFZP564G0222
203603_s_at	-2.2	-1.6	zinc finger homeobox 1B	ZFHX1B
203621_at	0.7	0.9	NADH dehydrogenase (ubiquinone) 1 betasubcomplex, 5 (16kD, SGDH)	NDUFB5
203637_s_at	-0.4	-1	midline 1	MID1
203697_at	-3.9	-3.2	Fritz	FRZB
203698_s_at	-2.8	-2.7	frizzled-related protein	FRZB
203706_s_at	-1.1	-1.5	frizzled 7	FZD7
203711_s_at	1.1	0.6	3-hydroxyisobutyryl-Coenzyme A hydrolase	HIBCH
203712_at	1.1	0.9	KIAA0020 gene product	KIAA0020
203721_s_at	0.5	0.9	CGI-48 protein	LOC51096
203755_at	1.6	0.5	budding uninhibited by benzimidazoles 1 (yeasthomolog), beta	BUB1B
203764_at	1.9	1.4	KIAA0008 gene product	KIAA0008
203798_s_at	1.2	0.9	visinin-like 1	VSNL1
203800_s_at	0.5	0.7	hypothetical protein LOC63931	LOC63931
203856_at	1	0.4	vaccinia related kinase 1	VRK1
203903_s_at	-1.2	-1.8	hephaestin	HEPH
203962_s_at	1	0.2	nebulette	NEBL
203964_at	0.6	0.3	N-myc and STAT interactor	NMI
204026_s_at	1.1	0.9	ZW10 interactor	ZWINT
204033_at	0.8	0.4	thyroid hormone receptor interactor 13	TRIP13
204082_at	0.6	0.8	pre-B-cell leukemia transcription factor 3	PBX3
204112_s_at	0.6	0.3	histamine N-methyltransferase	HNMT
204119_s_at	0.9	0.3	adenosine kinase short form	ADK
204135_at	-0.9	-1	downregulated in ovarian cancer 1	DOC1
204143_s_at	0.4	1.1	rTS beta protein	HSRTSBETA
204146_at	1.1	0.9	RAD51-interacting protein	PIR51
204151_x_at	0.9	0.1	aldo-keto reductase family 1, member C1(dihydrodiol dehydrogenase 1; 20-alpha(3-alpha)-hydroxysteroid dehydrogenase)	AKR1C1
204154_at	-1.4	-1.1	cysteine dioxygenase, type I	CDO1
204159_at	0.6	0.8	cyclin-dependent kinase inhibitor 2C (p18,inhibits CDK4)	CDKN2C
204162_at	2.9	1.9	highly expressed in cancer, rich in leucineheptad repeats	HEC
204170_s_at	1.1	0.7	CDC28 protein kinase 2	CKS2
204197_s_at	-1.6	-1.1	runt-related transcription factor 3	RUNX3
204237_at	-1.2	-1	CED-6 protein	CED-6
204271_s_at	-2	-1.3	endothelin receptor	
204273_at	-2.8	-1.3	endothelin receptor type B, isoform 1	EDNRB
204279_at	1.4	0.4	proteasome (prosome, macropain) subunit, betatype, 9 (large multifunctional protease 2)	PSMB9
204285_s_at	0.7	1.3	phorbol-12-myristate-13-acetate-induced protein 1	PMAIP1
204286_s_at	0.3	0.6	phorbol-12-myristate-13-acetate-induced protein1	PMAIP1
204305_at	0.9	0.9	mitochondrial intermediate peptidase	MIPEP
204362_at	0.8	1.1	SKAP55 homologue	SKAP-HOM
204364_s_at	0.8	2.3	hypothetical protein FLJ13110	FLJ13110
204373_s_at	0.8	0.6	KIAA0480 gene product	KIAA0480
204400_at	-0.6	-1	signal transduction protein (SH3 containing)	EFS2
204409_s_at	0.5	1.1	Homo sapiens, clone MGC:12282, mRNA, complete cds.	EIF1AY
204444_at	1.2	0.9	kinesin-like 1	KNSL1
204451_at	-0.7	-1.3	frizzled 1	FZD1
204464_s_at	-1.1	-1.4	endothelin receptor type A	EDNRA
204508_s_at	0.7	2.2	hypothetical protein FLJ20151	FLJ20151
204529_s_at	-1.8	-2.2	KIAA0808 gene product	KIAA0808
204531_s_at	1	0.1	breast cancer 1, early onset	BRCA1
204548_at	0.5	0.9	steroidogenic acute regulatory protein	STAR
204602_at	-1.3	-1.9	dickkopf (Xenopus laevis) homolog 1	DKK1
204620_s_at	-2	-1.4	chondroitin sulfate proteoglycan 2 (versican)	CSPG2
204636_at	0.4	2.2	collagen, type XVII, alpha 1	COL17A1
204682_at	-0.9	-1	latent transforming growth factor beta bindingprotein 2	LTBP2
204712_at	-6.6	-3.8	Wnt inhibitory factor-1	WIF-1
204725_s_at	1	0.5	NCK adaptor protein 1	NCK1
204766_s_at	3.7	3.3	nudix (nucleoside diphosphate linked moietyX)-type motif 1	NUDT1
204772_s_at	1	0.3	transcription termination factor, RNA polymeraseI	TTF1
204777_s_at	-2	-1.2	T-cell differentiation protein MAL, isoform a	MAL

204780_s_at	0.9	0.4	tumor necrosis factor receptor superfamily, member 6	TNFRSF6
204793_at	-0.9	-1.5	KIAA0443 gene product	KIAA0443
204808_s_at	0.6	1.4	transmembrane protein 5	TMEM5
204820_s_at	0.7	0.1	butyrophilin, subfamily 3, member A3	BTN3A3
204822_at	1.1	1.1	TTK protein kinase	TTK
204825_at	2	0.2	KIAA0175 gene product	KIAA0175
204836_at	-2	-1.9	glycine dehydrogenase (decarboxylating; glycinedecarboxylase, glycine cleavage system protein P)	GLDC
204869_at	-1.6	-1.7	proprotein convertase subtilisinkexin type 2	PCSK2
204872_at	-0.7	-1	BCE-1 protein	BCE-1
204905_s_at	0.3	0.5	eukaryotic translation elongation factor 1epsilon 1	EEF1E1
204948_s_at	-1.1	-1.9	follistatin isoform FST344 precursor	FST
204962_s_at	1.5	1.7	centromere protein A	CENPA
204976_s_at	0.7	0.2	Alport syndrome, mental retardation, midface hypoplasia and elliptocytosis chromosomal region gene 1	AMMECR1
205001_s_at	1.3	1.6	dead box, Y isoform	DBY
205029_s_at	0.7	0.6	fatty acid binding protein 7, brain	FABP7
205053_at	0.9	1	primase, polypeptide 1 (49kD)	PRIM1
205061_s_at	0.7	0.6	polymyositisscleroderma autoantigen 1 (75kD)	PMSCL1
205064_at	-1.5	-1.3	small proline-rich protein 1B (cornifin)	SPRR1B
205066_s_at	-1.6	-2.1	ectonucleotide pyrophosphatasephosphodiesterase1	ENPP1
205078_at	0.5	0.8	phosphatidylinositol glycan, class F	PIGF
205170_at	-1.5	-1.4	signal transducer and activator of transcription2, 113kD	STAT2
205176_s_at	0.4	0.6	integrin beta 3 binding protein(beta3-endonexin)	ITGB3BP
205194_at	-0.9	-1.1	phosphoserine phosphatase	PSPH
205246_at	0.8	2.5	peroxisome biogenesis factor 13	PEX13
205328_at	-1.5	-0.7	claudin 10	CLDN10
205337_at	-2.5	-1.2	dopachrome tautomerase (dopachrome delta-isomerase, tyrosine-related protein 2)	DCT
205345_at	1	2.5	BRCA1 associated RING domain 1	BARD1
205372_at	-0.6	-1.3	pleiomorphic adenoma gene 1	PLAG1
205379_at	0.2	0.5	carbonyl reductase 3	CBR3
205383_s_at	-1.1	-1.5	zinc finger protein 288	ZNF288
205404_at	-2.1	-2.3	hydroxysteroid (11-beta) dehydrogenase 1	HSD11B1
205412_at	0.8	0.4	acetyl-Coenzyme A acetyltransferase 1 precursor	ACAT1
205415_s_at	0.3	1.6	Machado-Joseph disease (spinocerebellar ataxia 3, olivopontocerebellar ataxia 3, autosomal dominant, ataxin 3)	MJD
205428_s_at	-0.6	-1	calbindin 2, full length protein isoform	CALB2
205434_s_at	-0.8	-1.6	KIAA1048 protein	KIAA1048
205440_s_at	1.2	0.6	neuropeptide Y receptor Y1	NPY1R
205443_at	0.7	1.2	small nuclear RNA activating complex,polypeptide 1, 43kD	SNAPC1
205480_s_at	0.7	1.6	UDP-glucose pyrophosphorylase 2	UGP2
205489_at	-1	-1.5	crystallin, mu	CRYM
205590_at	-1.3	-2.6	RAS guanyl releasing protein 1	RASGRP1
205601_s_at	-0.8	-0.9	homeo box B5	HOXB5
205659_at	-1.6	-2.5	histone deacetylase 7B	HDAC7B-PENDING
205690_s_at	0.3	0.9	maternal G10 transcript	G10
205694_at	-2.3	-1.5	tyrosinase-related protein 1	TYRP1
205711_x_at	0.5	2.3	ATP synthase, H+ transporting, mitochondrial F1complex, gamma polypeptide 1	ATP5C1
205752_s_at	-1	-1.2	glutathione S-transferase M5	GSTM5
205794_s_at	-1.8	-1.6	neuro-oncological ventral antigen 1, isoform 1	NOVA1
205848_at	-2.1	-2.6	growth arrest-specific 2	GAS2
205891_at	0.5	1.5	adenosine A2b receptor	ADORA2B
205909_at	1.4	0.6	polymerase (DNA directed), epsilon 2	POLE2
206002_at	-2.1	-2.3	G protein-coupled receptor 64	GPR64
206140_at	-5	-4.7	LIM homeobox protein 2	LHX2
206144_at	-1.1	-1	BAI1-associated protein 1	BAIAP1
206276_at	2	0.5	lymphocyte antigen 6 complex, locus D	E48
206295_at	0.6	0.6	interleukin 18	IL-18
206302_s_at	-0.5	-1.1	nudix (nucleoside diphosphate linked moietyX)-type motif 4	NUDT4
206314_at	-0.8	-1.2	zinc finger protein	ZFP
206323_x_at	-1.2	-1.2	oligophrenin 1, Rho-GTPase acivating protein	OPHN1
206364_at	1.3	0.5	KIAA0042 gene product	KIAA0042
206377_at	-1.3	-1.2	forkhead box F2	FOXF2
206400_at	0.3	1.8	galectin 7	LGALS7
206404_at	-1	-1.7	fibroblast growth factor 9 (glia-activatingfactor)	FGF9
206421_s_at	1.8	4	serine (or cysteine) proteinase inhibitor, cladeB (ovalbumin), member 7	SERPINB7
206426_at	-1	-1.5	melan-A	MLANA
206461_x_at	0.4	0.7	metallothionein 1H	MT1H
206465_at	-2.3	-3.5	very long-chain acyl-CoA synthetase; lipidosin	KIAA0631

206542_s_at	0.7	1	SWISNF related, matrix associated, actin dependent regulator of chromatin, subfamily a, member 2	SMARCA2
206562_s_at	1.2	1.5	casein kinase 1, alpha 1	CSNK1A1
206632_s_at	0.7	0.1	phorbolin (similar to apolipoprotein B mRNAediting protein)	DJ742C19.2
206642_at	0.7	1.4	desmoglein 1 preproprotein	DSG1
206734_at	-0.9	-1.1	jerky (mouse) homolog-like	JRKL
206766_at	-1.1	-1.4	integrin alpha 10 subunit	ITGA10
206953_s_at	-1	-1.6	latrophilin	KIAA0786
207016_s_at	-2.2	-2	RALDH2-T	RALDH2
207076_s_at	0.6	1.6	argininosuccinate synthetase	ASS
207165_at	2.1	1.6	hyaluronan-mediated motility receptor (RHAMM)	HMMR
207173_x_at	-1.8	-2.4	OB-cadherin-1	osf-4
207175_at	-1.9	-2.3	adipose most abundant gene transcript 1	APM1
207184_at	-1.1	-1.5	solute carrier family 6 (neurotransmittertransporter, GABA), member 13	SLC6A13
207291_at	1.1	0.8	transmembrane gamma-carboxyglutamic acid protein4	TMG4
207332_s_at	0.6	0.6	transferrin receptor (p90, CD71)	TFRC
207345_at	-2	-2.3	follistatin isoform FST317 precursor	FST
207369_at	-1.4	-1.8	bombesin-like receptor 3	BRS3
207431_s_at	0.7	0.2	degenerative spermatocyte	DEGS
207573_x_at	0.6	1	ATP synthase, H+ transporting, mitochondrialF1F0, subunit g	ATP5JG
207594_s_at	-1	-1.7	synaptojanin 1	SYNJ1
207654_x_at	0.6	0.6	down-regulator of transcription 1	DR1
207749_s_at	0.2	0.9	protein phosphatase 2 (formerly 2A), regulatorysubunit B (PR 72), alpha isoform and (PR 130), betaisoform	PPP2R3
207981_s_at	-0.9	-2.4	estrogen-related receptor gamma	ESRRG
208029_s_at	0.3	1.4	putative integral membrane transporter	LC27
208066_s_at	0.4	0.4	general transcription factor IIB	GTF2B
208079_s_at	2	0.3	serinethreonine kinase 6	STK6
208095_s_at	1.8	1.2	calciumcalmodulin-dependent protein kinase (CaMkinase) II gamma	CAMK2G
208216_at	-0.9	-1.2	distal-less homeobox 4	DLX4
208309_s_at	1	1.4	mucosa associated lymphoid tissue lymphomatranslocation gene 1	MALT1
208393_s_at	0.6	0.6	RAD50 (S. cerevisiae) homolog	RAD50
208517_x_at	0.7	1.3	basic transcription factor 3	BTF3
208581_x_at	0.3	0.9	metallothionein 1X	MT1X
208606_s_at	0.7	3.6	wingless-type MMTV integration site family,member 4	WNT4
208612_at	0.9	0.6	ER-60 protease	GRP58
208626_s_at	-0.7	-1.1	Similar to membrane protein of cholinergicsynaptic vesicles	VATI
208636_at	-0.7	-1.1	actinin, alpha 1	ACTN1
208650_s_at	-1.5	-1.1	CD24 antigen (small cell lung carcinoma cluster 4 antigen)	CD24
208662_s_at	1	1.4	tetratricopeptide repeat domain 3	TTC3
208670_s_at	0.8	1.4	PNAS-26	CRI1
208671_at	0.5	0.9	Diff33 protein homolog	KIAA1253
208679_s_at	0.4	2.2	PNAS-139	ARPC2
208680_at	0.5	0.9	Human natural killer cell enhancing factor (NKEFA) mRNA, complete cds.	NKEFA
208685_x_at	0.2	1	bromodomain-containing 2	BRD2
208687_x_at	0.6	0.6	constitutive heat shock protein 70	HSC70
208689_s_at	0.5	1.9	ribophorin II	RPN2
208695_s_at	-0.5	-1.1	ribosomal protein L39	RPL39
208696_at	0.5	2.3	PNAS-102	CCT5
208708_x_at	0.6	0.1	hypothetical protein	DKFZp564N1916
208726_s_at	0.6	0.8	eukaryotic translation initiation factor 2,subunit 2 (beta, 38kD)	EIF2S2
208734_x_at	0.8	2.6	GTP-binding protein	RAB2
208736_at	0.3	0.5	p21-Arc	ARPC3
208739_x_at	0.8	0.7	MIF2 suppressor	HSMT3
208743_s_at	0.8	4.8	tyrosine 3-monooxygenasetryptophan5-monooxygenase activation protein, beta polypeptide	YWHAB
208756_at	0.2	0.7	TGF-beta receptor interacting protein 1	EIF3S2
208758_at	0.8	0.9	5-aminoimidazole-4-carboxamide ribonucleotidetransformylase	ATIC
208761_s_at	0.3	2.5	SUMO-1	UBL1
208771_s_at	0.3	0.4	leukotriene A4 hydrolase	LTA4H
208805_at	0.9	1	proteasome (prosome, macropain) subunit, alphatype 6	PSMA6
208812_x_at	0.6	0.4	major histocompatibility complex, class I, C	HLA-C
208827_at	0.5	0.4	Similar to proteasome (prosome, macropain)subunit, beta type 6	PSMB6
208835_s_at	1.1	0.9	cisplatin resistance-associated overexpressed protein	LUC7A
208851_s_at	-0.7	-1	hypothetical protein	DKFZp761B15121
208864_s_at	1	1	thioredoxin	TXN
208898_at	0.4	0.3	vacuolar ATP synthase subunit D homolog	VATD
208909_at	0.9	1	ubiquinol-cytochrome c reductase, Rieskeiron-sulfur polypeptide 1	UQCRFS1
208910_s_at	0.8	4.1	splicing factor	C1QBP
208940_at	0.5	1.2	SELENOPHOSPHATE SYNTHETASE ; Human selenium donor protein	SPS

208974_x_at	0.4	0.4	karyopherin (importin) beta 1	KPNB1
208991_at	0.4	0.6	signal transducer and activator of transcription 3 (acute-phase response factor)	STAT3
208993_s_at	1.7	0.3	peptidyl-prolyl isomerase G (cyclophilin G)	PPIG
209009_at	0.6	1.1	Similar to esterase 10	ESD
209024_s_at	1.2	1	NS1-associated protein 1	NSAP1
209040_s_at	1.5	3.4	proteasome subunit LMP7	LMP7
209042_s_at	0.6	0.5	Similar to ubiquitin-conjugating enzyme E2G 2(homologous to yeast UBC7)	UBE2G2
209043_at	0.5	1.2	bifunctional ATP sulfurylaseadenosine5-phosphosulfate kinase	PAPSS1
209059_s_at	0.2	1.6	hMBF1alpha	EDF1
209104_s_at	0.7	1.5	likely homolog of yeast Nhp2, component of theHACA snoRNP; hypothetical protein FLJ20479	NOLA2
209118_s_at	-0.8	-1.1	alpha-tubulin	TUBA3
209140_x_at	1	2	major histocompatibility complex	HLA-B39
209157_at	0.6	0.1	Homo sapiens DNJ3CPR3 mRNA, complete cds.	DNJ3CPR3
209161_at	0.9	2.4	PRP4STKWD splicing factor	HPRP4P
209168_at	-1.5	-1.3	glycoprotein M6B	GPM6B
209170_s_at	-2.5	-1.7	glycoprotein M6B	m6b1
209225_x_at	0.2	0.2	karyopherin (importin) beta 2	KPNB2
209228_x_at	0.6	0.5	Putative prostate cancer tumor suppressor	N33
209242_at	-0.9	-1.2	paternally expressed 3	PEG3
209288_s_at	-1.4	-0.9	hypothetical protein	DKFZp434A0530
209301_at	1.2	0.9	carbonic anhydrase II	CA2
209330_s_at	1.3	0.9	heterogeneous nuclear ribonucleoprotein D (hnRNPD)	HNRPD
209335_at	-0.8	-0.8	decorin	DCN
209337_at	0.5	0.3	lens epithelium-derived growth factor	PSIP1
209360_s_at	-2.3	-1.4	AML1b protein	AML1
209369_at	0.6	0.8	1,2-cyclic-inositol-phosphate phosphodiesterase	ANX3
209382_at	0.4	1.6	RNA polymerase III subunit	RPC62
209392_at	-2	-1.7	autotaxin	ENPP2
209393_s_at	0.9	2.6	cap-binding protein 4EHP	EIF4EL3
209409_at	-1.1	-1.8	growth factor receptor-bound protein 10	KIAA0207
209421_at	0.7	1.3	mutS (E. coli) homolog 2 (colon cancer, nonpolyposis type 1)	hMSH2
209435_s_at	-1.3	-1	Unknown (protein for MGC:3182)	
209448_at	0.6	0.4	Tat-interacting protein (30kD)	TIP30
209459_s_at	-0.8	-1.6	Homo sapiens NPD009 mRNA, complete cds.	NPD009
209481_at	0.4	0.7	Homo sapiens HSNFRK (HSNFRK) mRNA, complete cds.	HSNFRK
209512_at	1.1	2.5	Similar to RIKEN cDNA 2610207I16 gene	
209572_s_at	0.8	0.4	embryonic ectoderm development protein	EED
209579_s_at	0.6	1	methyl-CpG binding domain protein 4	MBD4
209583_s_at	-2.9	-1.5	brain my033 protein	MOX2
209586_s_at	0.5	0.3	prune protein	HTCD37
209608_s_at	0.9	0.5	acetyl-Coenzyme A acetyltransferase 2 (acetoacetyl Coenzyme A thiolase)	ACAT2
209620_s_at	1.9	0.5	ABC transporter 7 protein	hABC7
209656_s_at	-1.1	-2.4	hypothetical protein	DKFZp761J17121
209669_s_at	0.8	1.3	Similar to DKFZP564M2423 protein	
209694_at	0.4	0.8	6-pyruvoyltetrahydropterin synthase	PTS
209699_x_at	1	0.2	dihydrodiol dehydrogenase	AKR1C2
209714_s_at	0.7	0.9	cyclin-dependent kinase associated proteinphosphatase	CDKN3
209732_at	0.7	1.4	Similar to C-type (calcium dependent,carbohydrate-recognition domain) lectin, superfamilymember 2 (activation-induced)	CLECSF2
209771_x_at	-1.8	-1.6	CD24 antigen (small cell lung carcinoma cluster 4 antigen)	CD24
209773_s_at	1.4	2.3	ribonucleotide reductase M2 polypeptide	RRM2
209790_s_at	0.4	0.9	caspase 6, apoptosis-related cysteine protease	CASP6
209846_s_at	1	2	Similar to butyrophilin, subfamily 3, member A2	BTN3A2
209849_s_at	1.2	0.8	Homo sapiens Rad51C (RAD51C) mRNA, complete cds.	RAD51C
209861_s_at	1.2	2.2	eIF-2-associated p67 homolog	MNPEP
209891_at	0.4	0.1	Homo sapiens AD024 mRNA, complete cds.	AD024
210053_at	0.3	0.4	TATA box binding protein (TBP)-associated factor, RNA polymerase II, D, 100kD	TAF2D
210059_s_at	0.6	0.2	mitogen-activated protein kinase 13	MAPK13
210093_s_at	0.3	1.4	Mago homolog	MAGOH
210095_s_at	0.6	2	Human growth hormone-dependent insulin-like growth factor-binding protein mRNA, complete cds.	IGFBP1
210105_s_at	-1.6	-1.8	FYN oncogene related to SRC, FGR, YES	FYN
210115_at	2.8	1.3	ribosomal protein L39	
210136_at	0.9	1.8	myelin basic protein	MBP
210147_at	-2.4	-2.6	mono-ADP-ribosyltransferase	htMART
210162_s_at	-1.1	-1.7	Human NF-ATc mRNA, complete cds.	NFATC1
210178_x_at	0.6	1.3	TLS-associated protein TASR	TASR1
210247_at	-1.3	-1	synapsin II	SYN2
210250_x_at	0.4	0.3	adenylosuccinate lyase	ADSL

210296_s_at	0.6	0.9	peroxisomal membrane protein 3 (35kD, Zellwegersyndrome)	PXMP3
210335_at	0.8	0.3	PAM COOH-terminal interactor protein 1	PCIP1
210365_at	-1.8	-1.5	AML1a protein	AML1
210374_x_at	-1	-0.8	prostaglandin E receotor EP3 subtype 4 isoform	PTGER3
210426_x_at	0.6	2.4	Human orphan hormone nuclear receptor RORalpha1 mRNA, complete cds.	RORA
210479_s_at	0.6	2.5	Human transcription factor RZR-alpha mRNA, complete cds.	RORA
210517_s_at	-1.1	-1	gravin	AKAP12
210544_s_at	1.1	0.6	aldehyde dehydrogenase 10 (fatty aldehydedehydrogenase)	ALDH3A2
210567_s_at	1.3	0.2	Similar to S-phase kinase-associated protein 2 (p45)	SKP2
210574_s_at	0.5	0.6	Homo sapiens NPD011 (NPD011) mRNA, complete cds.	NPD011
210653_s_at	0.4	0.9	branched chain alpha-keto acid dehydrogenaseE1-beta subunit	BCKDHB
210759_s_at	1.2	1.1	prosomal protein P30-33K	pros-30
210766_s_at	0.7	0.9	cellular apoptosis susceptibility protein	CSE1
210792_x_at	2.4	0.6	Siva-2	SIVA
210800_at	-1.3	-1.2	Unknown (protein for MGC:12262)	TIMM8A
210830_s_at	0.9	1.7	paraoxonase	PON2
210944_s_at	-0.9	-1.4	Similar to calpain 3, (p94)	CAPN3
210946_at	-1	-1.3	type-2 phosphatidic acid phosphatase alpha-2	PAP2-a2
210947_s_at	0.6	0.6	Human MSH3 gene, complete cds.	MSH3
210986_s_at	-0.6	-0.9	tropomyosin isoform	TPM1
210993_s_at	0.3	0.8	mad-related protein MADR1	MADR1
211015_s_at	0.7	1.4	heat shock protein 70	hsp70
211071_s_at	-1.3	-1	ALL1-fused gene from chromosome 1q	
211075_s_at	1.1	1.8	integrin associated protein	
211276_at	-1.3	-2.4	brain my048 protein	
211297_s_at	1.5	2.3	protein serinethreonine kinase	CDK7
211340_s_at	-1	-1.5	MUC18 glycoprotein	MCAM
211368_s_at	1.1	0.2	Interleukin 1-beta converting enzyme isoform epsilon	IL1BCE
211372_s_at	0.5	0.8	soluble type II interleukin-1 receptor	IL1R2
211445_x_at	-1.1	-0.9	Homo sapiens FKSG17 (FKSG17) mRNA, complete cds.	FKSG17
211454_x_at	-1.6	-1.3	Homo sapiens FKSG51 (FKSG51) mRNA, complete cds.	FKSG51
211466_at	-1.2	-1.1	nuclear factor I B3	NFIB
211600_at	-2.3	-1.5	glomerular epithelial protein 1	GLEPP1
211623_s_at	0.5	1.2	Human casein kinase II beta subunit mRNA, complete cds.	CSNK2B
211701_s_at	-1.3	-1.3	magphinin beta	TRO
211762_s_at	1.1	0.7	karyopherin alpha 2 (RAG cohort 1, importinalpha 1)	
211936_at	1.1	0.6	endoplasmic reticulum lumenal Ca2+ bindingprotein grp78	HSPA5
211959_at	-0.9	-1.1	Human insulin-like growth factor binding protein 5 (IGFBP5) mRNA	
211968_s_at	1.6	2.2	heat shock 90kD protein 1, alpha	HSPCA
211980_at	-1.9	-1.2	collagen, type IV, alpha 1	COL4A1
211984_at	0.5	0.6	matrix Gla protein	MGP
211985_s_at	1.1	0.8	matrix Gla protein	MGP
212007_at	1	2.4	UBX domain-containing 1	UBXDC1
212012_at	-2.1	-1.9	Melanoma associated gene	D2S448
212030_at	0.5	0.8	S164 protein	S164
212038_s_at	0.7	1.9	voltage-dependent anion channel 1	VDAC1
212052_s_at	-0.4	-1.2	KIAA0676 protein	KIAA0676
212096_s_at	0.7	1.1	KIAA1288 protein	KIAA1288
212129_at	0.5	0.1	hypothetical protein MGC5466	MGC5466
212180_at	0.6	0.7	Homo sapiens mRNA; cDNA DKFZp564E2222 (from clone DKFZp564E2222)	
212190_at	-1.8	-2.5	trinucleotide repeat containing 3	TNRC3
212199_at	0.4	1.1	Human putative ribosomal protein S1 mRNA	
212230_at	-1.6	-1.3	Homo sapiens phosphatidic acid phosphatase type 2B (PPAP2B), mRNA	
212233_at	-1.6	-1.3	H.sapiens mRNA for 3UTR of unknown protein	
212247_at	0.6	0.6	KIAA0225 protein	KIAA0225
212264_s_at	0.5	0.9	KIAA0261 protein	KIAA0261
212266_s_at	0.7	1.2	splicing factor, arginineserine-rich 5	SFRS5
212296_at	0.5	0.1	26S proteasome-associated pad1 homolog	POH1
212327_at	0.4	0.1	KIAA1102 protein	KIAA1102
212330_at	0.4	0.6	hypothetical protein FLJ13576	FLJ13576
212337_at	0.4	0.7	peptidylprolyl isomerase B (cyclophilin B)	PPIB
212353_at	-1.9	-1.3	KIAA1077 protein	KIAA1077
212397_at	0.3	1.1	hypothetical protein	DKFZp434I0812
212419_at	-1	-2	Homo sapiens mRNA; cDNA DKFZp564L0822 (from clone DKFZp564L0822)	
212425_at	-0.8	-1.2	secretory carrier membrane protein 1	SCAMP1
212428_at	0.7	0.8	KIAA0368 protein	KIAA0368
212442_s_at	0.5	1.2	Homo sapiens cDNA: FLJ21238 fis, clone COL01115	
212450_at	-0.7	-1	KIAA0256 protein	KIAA0256
212473_s_at	-0.9	-1.5	Homo sapiens cDNA: FLJ22463 fis, clone HRC10126	
212510_at	0.7	0.2	KIAA0089 protein	KIAA0089

212544_at	0.6	1.9	thyroid hormone receptor interactor 3	TRIP3
212591_at	0.7	0.8	RBP1-like protein	BCAA
212613_at	1.2	2.3	butyrophilin, subfamily 3, member A2	BTN3A2
212621_at	0.4	0.7	Homo sapiens mRNA for KIAA0286 gene, partial cds.	KIAA0286
212626_x_at	0.3	1.2	heterogeneous nuclear ribonucleoprotein C (C1C2)	HNRPC
212627_s_at	0.6	0.3	KIAA0116 protein	KIAA0116
212749_s_at	0.6	1.5	DKFZP586C1620 protein	DKFZP586C1620
212761_at	-0.6	-1.2	transcription factor 7-like 2 (T-cell specific, HMG-box)	TCF7L2
212766_s_at	0.2	0.2	Homo sapiens, Similar to hypothetical protein FLJ12484, clone MGC:3008, mRNA, complete	
212768_s_at	-4.5	-2	differentially expressed in hematopoietic lineages	GW112
212773_s_at	0.4	0.4	translocase of outer mitochondrial membrane 20 (yeast) homolog	KIAA0016
212865_s_at	-1.3	-1.1	collagen, type XIV, alpha 1 (undulin)	COL14A1
212915_at	-2	-2.6	KIAA1095 protein	KIAA1095
212916_at	-0.4	-1.1	KIAA1111 protein	KIAA1111
212918_at	0.5	0.3	hypothetical protein FLJ22028	FLJ22028
212946_at	-1.1	-1.1	KIAA0564 protein	KIAA0564
212977_at	0.9	1.1	G protein-coupled receptor	RDC1
212980_at	-0.5	-1.7	Homo sapiens mRNA; cDNA DKFZp586J101 (from clone DKFZp586J101)	
213026_at	0.8	1.4	Apg12 (autophagy 12, S. cerevisiae)-like	APG12L
213080_x_at	-0.5	-0.9	ribosomal protein L5	RPL5
213088_s_at	0.9	0.6	KIAA0974 protein	KIAA0974
213097_s_at	0.4	0.2	zuotin related factor 1	ZRF1
213106_at	-1.3	-1.5	Homo sapiens clone 23664 and 23905 mRNA sequence	
213137_s_at	0.4	0.7	protein tyrosine phosphatase, non-receptor type 2	PTPN2
213169_at	-0.9	-1.3	Homo sapiens clone TUA8 Cri-du-chat region mRNA	
213183_s_at	-1.5	-2.1	cyclin-dependent kinase inhibitor 1C (p57, Kip2)	CDKN1C
213212_x_at	-0.9	-1.3	golgin-67	KIAA0855
213241_at	-2.2	-1.5	Homo sapiens clone 23785 mRNA sequence.	
213260_at	-1	-1.7	forkhead box C1	FOXC1
213266_at	-0.5	-1.3	gamma tubulin ring complex protein (76p gene)	76P
213275_x_at	-1.4	-1	cathepsin B	CTSB
213305_s_at	1	3.8	protein phosphatase 2A B56-gamma1	PP2A
213307_at	-1.8	-1.6	cortactin SH3 domain-binding protein	KIAA1022
213326_at	-1.1	-1.4	vesicle-associated membrane protein 1 (synaptobrevin 1)	VAMP1
213327_s_at	0.8	0.7	ubiquitin specific protease 12	USP12
213350_at	-0.7	-1.8	ribosomal protein S11	RPS11
213366_x_at	0.4	1.5	ATP synthase, H+ transporting, mitochondrial F1 complex, gamma polypeptide 1	ATP5C1
213379_at	1.1	0.2	Homo sapiens clone 640 unknown mRNA, complete sequence.	CL640
213413_at	-1.4	-1.6	Homo sapiens cDNA FLJ13555 fis, clone PLACE1007677	
213423_x_at	0.5	0.1	Putative prostate cancer tumor suppressor	N33
213427_at	0.9	0.2	ribonuclease P, 40kD subunit	RPP40
213428_s_at	-0.7	-1.1	collagen, type VI, alpha 1	COL6A1
213447_at	-1	-1	imprinted in Prader-Willi syndrome	IPW
213454_at	0.6	0.6	cortistatin	CORT
213465_s_at	0.9	1.1	protein phosphatase 1, regulatory subunit 7	PPP1R7
213476_x_at	0.3	3.2	tubulin, beta, 4	TUBB4
213486_at	-1	-1.6	hypothetical protein DKFZp761N09121	DKFZP761N09121
213496_at	-1.9	-1.3	KIAA0455 gene product	KIAA0455
213571_s_at	0.5	0.1	eukaryotic translation initiation factor 4E-like 3	EIF4EL3
213574_s_at	0.9	0.1	karyopherin (importin) beta 1	KPNB1
213588_x_at	-0.4	-1	ribosomal protein L14	RPL14
213622_at	-1.5	-1.6	collagen, type IX, alpha 2	COL9A2
213627_at	-0.6	-0.8	hepatocellular carcinoma associated protein; breast cancer associated gene 1	JCL-1
213649_at	1	1.6	splicing factor, arginineserine-rich 7 (35kD)	SFRS7
213653_at	1.2	0.8	putative methyltransferase	M6A
213655_at	0.5	0.4	tyrosine 3-monooxygenasetryptophan 5-monooxygenase activation protein, epsilon polypeptide	YWHAE
213702_x_at	-0.6	-0.9	N-acylsphingosine amidohydrolase (acid ceramidase)	ASAH
213729_at	1	1.1	Huntingtin-interacting protein A	HYPA
213742_at	-0.9	-0.1	Homo sapiens cDNA FLJ11680 fis, clone HEMBA1004820	
213803_at	0.9	1.2	karyopherin (importin) beta 1	KPNB1
213836_s_at	-1.1	-2	KIAA1001 protein	KIAA1001
213880_at	-1.6	-2	G protein-coupled receptor 49	GPR49
213883_s_at	-0.5	-0.9	glucose phosphate isomerase	GPI
213895_at	0.8	0.8	epithelial membrane protein 1	EMP1
213900_at	-0.8	-1.1	Friedreich ataxia region gene X123	X123
214001_x_at	-0.9	-1.9	ribosomal protein S10	RPS10
214041_x_at	-1.6	-2.5	ribosomal protein L37a	RPL37A
214045_at	0.8	1.1	lipoic acid synthetase	LAS
214086_s_at	0.8	0.9	ADP-ribosyltransferase (NAD+; poly (ADP-ribose) polymerase)-like 2	ADPRTL2

214152_at	-0.6	-1.2	phosphatidylinositol glycan, class B	PIGB
214214_s_at	0.2	0.3	complement component 1, q subcomponent binding protein	C1QBP
214414_x_at	-2.7	-2.2	hemoglobin, alpha 1	HBA1
214459_x_at	0.7	1.1	Cw1 antigen	HLA-C
214499_s_at	0.5	1.7	Bcl-2-associated transcription factor shortform	KIAA0164
214501_s_at	0.7	0.8	histone macroH2A1.1	H2AFY
214582_at	-1.6	-1.6	2,3-cyclic nucleotide 3 phosphodiesterase	CNP
214610_at	-1.1	-1.3	cytochrome P450, subfamily XIB (steroid 11-beta-hydroxylase), polypeptide 1	CYP11B1
214710_s_at	2.2	1.8	cyclin B1	CCNB1
214734_at	0.5	1.5	KIAA0624 protein	KIAA0624
214741_at	1.2	3.4	zinc finger protein 131 (clone pHZ-10)	ZNF131
214772_at	-1.5	-1.6	G2 protein	G2
214798_at	-0.9	-1.3	Homo sapiens cDNA: FLJ21771 fis, clone COLF7779	
214861_at	-3.1	-1.5	gene amplified in squamous cell carcinoma 1; KIAA0780 protein	KIAA0780
214866_at	-0.9	-1.3	urokinase plasminogen activator receptor	PLAUR
214882_s_at	0.7	0.8	splicing factor, arginineserine-rich 2	SFRS2
214911_s_at	0.6	1.3	bromodomain-containing 2	BRD2
214949_at	0.4	1.1	Homo sapiens mRNA; cDNA DKFZp586L141 (from clone DKFZp586L141).	
214951_at	-0.2	-2.5	hypothetical protein	DKFZp564M1916
214996_at	-0.6	-0.8	Homo sapiens mRNA full length insert cDNA clone EUROIMAGE 362780.	
215067_x_at	-1.4	-1	Homo sapiens cDNA FLJ12333 fis, clone MAMMA1002198, highly similar to THIOREDOXIN PEROXIDASE 1	
215091_s_at	0.5	0.8	general transcription factor IIIA	GTF3A
215148_s_at	-1.3	-0.9	amyloid beta (A4) precursor protein-binding, family A, member 3 (X11-like 2)	APBA3
215160_x_at	0.4	0.8	FSHD region gene 1	FRG1
215171_s_at	0.3	0.1	translocase of inner mitochondrial membrane 17 (yeast) homolog A	TIM17
215204_at	-1.4	-1.1	Homo sapiens cDNA FLJ14090 fis, clone MAMMA1000264	
215227_x_at	0.3	0.5	acid phosphatase 1, soluble	ACP1
215306_at	-1.7	-2.3	Homo sapiens mRNA; cDNA DKFZp586N2020 (from clone DKFZp586N2020)	
215313_x_at	0.9	0.9	major histocompatibility complex, class I, A	HLA-A
215424_s_at	1.3	1.5	SKI-INTERACTING PROTEIN	SNW1
215479_at	-1.8	-1.4	Homo sapiens cDNA FLJ20780 fis, clone COL04256	
215516_at	0.7	2	laminin, beta 4	LAMB4
215525_at	-0.5	-1.8	Homo sapiens mRNA; cDNA DKFZp586A0423 (from clone DKFZp586A0423)	
215629_s_at	0.6	0.4	BCMS-upstream neighbor-like (BCMSUNL)	BCMSUNL
215695_s_at	-0.7	-1.9	glycogenin-2 delta	glycogenin-2
215704_at	-2.4	-2	filaggrin	FLG
215773_x_at	0.6	0.2	poly-(ADP-ribose) polymerase II	PARP2
216034_at	-1.5	-1.9	Homo sapiens immunoglobulin lambda gene locus DNA, clone:288A10	
216061_x_at	-1	-1.1	platelet-derived growth factor beta polypeptide (simian sarcoma viral (v-sis) oncogene homolog)	PDGFB
216064_s_at	0.8	0.8	aspartylglucosaminidase	AGA
216088_s_at	0.6	5.2	proteasome (prosome, macropain) subunit, alpha type, 7	PSMA7
216194_s_at	0.4	0.3	cytoskeleton-associated protein 1	CKAP1
216246_at	-1	-1.8	ribosomal protein S20	RPS20
216274_s_at	0.8	0.5	signal peptidase complex (18kD)	SPC18
216341_s_at	-1.7	-1.4	gonadotropin-releasing hormone receptor	GNRHR
216379_x_at	-1.8	-1.6	Homo sapiens CD24 signal transducer mRNA.	
216526_x_at	0.8	0.7	major histocompatibility complex, class I, C	HLA-C
216652_s_at	0.7	0.5	Homo sapiens mRNA; cDNA DKFZp434H0872 (from clone DKFZp434H0872).	
216977_x_at	0.9	1.1	U2 snRNP-specific A protein	SNRPA1
216988_s_at	0.6	1.4	protein tyrosine phosphatase type IVA, member 2	PTP4A2
217427_s_at	1.6	0.4	TUP1 like enhancer of SPLIT gene 1	TUPLE1
217627_at	-0.6	-0.9	ESTs, Weakly similar to S47072 finger protein HZF10, Krueppel-related (H.sapiens)	
217717_s_at	0.7	1.7	GW128 protein	GW128
217722_s_at	0.6	1.2	mesenchymal stem cell protein DSC92	LOC51335
217747_s_at	0.3	1	ribosomal protein S9	RPS9
217762_s_at	0.8	0.2	RAB31, member RAS oncogene family	RAB31
217764_s_at	1	0.5	small GTP-binding protein rab22b	RAB31
217772_s_at	1.1	0.2	mitochondrial carrier homolog 2	MTCH2
217835_x_at	0.6	1	putative Rab5-interacting protein	LOC55969
217848_s_at	0.4	0.7	pyrophosphatase (inorganic)	PP
217850_at	0.3	0.7	putative nucleotide binding protein,estradiol-induced	E2IG3
217871_s_at	0.4	1.8	macrophage migration inhibitory factor(glycosylation-inhibiting factor)	MIF
217897_at	-1.4	-1.6	FXYD domain-containing ion transport regulator 6	FXYD6
217898_at	0.6	0.3	chromosome 11 hypothetical protein ORF3	LOC56851
217919_s_at	1	0.2	PTD007 protein	PTD007
217933_s_at	0.4	1.7	leucine aminopeptidase	LOC51056
217955_at	0.7	1.5	MIL1 protein	MIL1
217959_s_at	0.9	2.3	PTD009 protein	PTD009
217976_s_at	0.2	0.2	dynein light chain-A	LOC51143

217985_s_at	0.8	2.1	bromodomain adjacent to zinc finger domain, 1A	BAZ1A
217987_at	0.6	0.4	hypothetical protein	FLJ20752
217991_x_at	-1.1	-0.7	hypothetical protein FLJ10355	FLJ10355
217995_at	0.7	1	CGI-44 protein; sulfide dehydrogenase like(yeast)	CGI-44
218002_s_at	0.3	0.1	small inducible cytokine subfamily B(Cys-X-Cys), member 14 (BRAK)	SCYB14
218009_s_at	1.6	0.9	protein regulator of cytokinesis 1	PRC1
218011_at	0.6	0.8	ubiquitin-like 5	UBL5
218020_s_at	-0.6	-1	hypothetical protein FLJ13222	FLJ13222
218031_s_at	0.4	0.9	hypothetical protein PRO1635	PRO1635
218039_at	1.5	0.5	Homo sapiens clone HQ0310 PRO0310p1 (LOC51203), mRNA.	LOC51203
218042_at	0.3	0.6	COP9 complex subunit 4	LOC51138
218049_s_at	0.6	1.5	L13 protein	L13
218088_s_at	0.8	0.3	Rag C protein	GTR2
218104_at	0.3	0.1	hypothetical protein FLJ20287	FLJ20287
218108_at	0.5	1.1	hypothetical protein FLJ10483	FLJ10483
218109_s_at	0.7	0.2	hypothetical protein FLJ14153	FLJ14153
218118_s_at	1.6	0.9	translocase of inner mitochondrial membrane 23(yeast) homolog	TIM23
218123_at	1.4	1.5	Homo sapiens chromosome 21 open reading frame 59 (C21ORF59), mRNA. / hypothetical protein FLJ20467	C21ORF59
218135_at	0.8	0.3	CDA14	LOC51290
218140_x_at	0.6	0.4	APMCF1 protein	APMCF1
218160_at	0.4	0.4	NADH dehydrogenase (ubiquinone) 1 alphasubcomplex, 8 (19kD, PGIV)	NDUFA8
218163_at	0.4	0.3	MCT-1 protein	MCT-1
218167_at	0.6	2.1	hypothetical protein	LOC51321
218185_s_at	0.5	0.2	hypothetical protein FLJ10511	FLJ10511
218190_s_at	0.4	0.7	ubiquinol-cytochrome c reductase complex (7.2kD)	HSPC051
218211_s_at	-1.7	-1.6	hypothetical protein MGC2771	MGC2771
218229_s_at	0.2	1.6	KIAA1513 protein	KIAA1513
218238_at	0.7	0.4	GTP-binding protein	NGB
218249_at	0.7	0.1	hypothetical protein FLJ21952	FLJ21952
218250_s_at	0.5	0.4	CCR4-NOT transcription complex, subunit 7	CNOT7
218252_at	1	0.6	cytoskeleton associated protein 2	CKAP2
218268_at	0.5	0.2	hypothetical protein FLJ12085	FLJ12085
218309_at	-1.5	-1.3	hypothetical protein PRO1489	PRO1489
218330_s_at	-0.8	-1.7	hypothetical protein FLJ10633	FLJ10633
218333_at	0.5	0.3	CGI-101 protein	LOC51009
218349_s_at	1.1	2.3	hypothetical protein FLJ10036	FLJ10036
218373_at	0.4	0.9	hypothetical protein FLJ13258 similar to fusedtoes	FLJ13258
218375_at	0.7	0.8	hypothetical protein MGC3037	MGC3037
218399_s_at	0.5	0.7	hypothetical protein FLJ20764	FLJ20764
218415_at	0.4	0.1	vacuolar protein sorting 33B (yeast homolog)	VPS33B
218447_at	0.6	0.4	DC13 protein	DC13
218449_at	0.7	1.2	hypothetical protein FLJ11200	FLJ11200
218450_at	0.2	1.6	heme-binding protein	HEBP
218477_at	0.6	0.7	PTD011 protein	PTD011
218502_s_at	-2	-1.6	trichorhinophalangeal syndrome I gene	TRPS1
218535_s_at	0.1	0.4	hypothetical protein FLJ11159	FLJ11159
218542_at	2	2.2	hypothetical protein FLJ10540	FLJ10540
218557_at	0.9	1.6	Nit protein 2	NIT2
218558_s_at	1	0.7	hypothetical protein	PRED22
218561_s_at	0.4	0.9	CGI-203 protein	CGI-203
218574_s_at	-1.3	-2.7	LIM and cysteine-rich domains 1	LMCD1
218585_s_at	1.5	0.9	L2DTL protein	L2DTL
218587_s_at	0.5	0.4	x 010 protein	MDS010
218605_at	0.5	1.4	hypothetical protein FLJ23182	FLJ23182
218622_at	1	1.6	hypothetical protein MGC5585	MGC5585
218637_at	-0.9	-1.1	hypothetical protein IMPACT	IMPACT
218662_s_at	1.7	0.2	chromosome condensation protein G	HCAP-G
218663_at	3.9	3.6	chromosome condensation protein G	HCAP-G
218721_s_at	0.7	0.7	hypothetical protein FLJ20505	FLJ20505
218728_s_at	0.9	1.3	HSPC163 protein	HSPC163
218751_s_at	2.3	1.7	hypothetical protein FLJ11071	FLJ11071
218768_at	0.8	0.1	nuclear pore complex protein	NUP107
218771_at	-0.8	-1.1	hypothetical protein FLJ10782	FLJ10782
218784_s_at	0.7	2.5	hypothetical protein FLJ11101	FLJ11101
218802_at	0.6	0.3	hypothetical protein FLJ20647	FLJ20647
218827_s_at	0.3	0.3	hypothetical protein FLJ10352	FLJ10352
218852_at	1	0.6	hypothetical protein FLJ20644	FLJ20644
218856_at	0.3	0.3	hypothetical protein	LOC51323
218859_s_at	1	0.9	HDCMC28P protein	HDCMC28P
218875_s_at	0.5	0.9	F-box only protein 5	FBXO5

218883_s_at	1.1	0.9	hypothetical protein FLJ23468	FLJ23468
218898_at	0.8	0.6	hypothetical protein FLJ22282	FLJ22282
218932_at	0.5	0.4	hypothetical protein FLJ20729	FLJ20729
218947_s_at	0.4	1.2	hypothetical protein FLJ10486	FLJ10486
218974_at	-2.2	-1.4	hypothetical protein FLJ10159	FLJ10159
218993_at	0.3	0.6	hypothetical protein FLJ10581	FLJ10581
219031_s_at	0.5	0.7	hypothetical protein	HSPC031
219038_at	0.7	0.3	hypothetical protein FLJ11565	FLJ11565
219043_s_at	0.4	1.8	hypothetical protein MGC3062	MGC3062
219087_at	1.9	2.2	asporin (LRR class 1)	ASPN
219104_at	1.1	0.2	C3HC4-like zinc finger protein	ZFP26
219147_s_at	0.3	0.2	hypothetical protein FLJ20559	FLJ20559
219148_at	1.7	1.1	Homo sapiens PDZ-binding kinase; T-cell originated protein kinase (TOPK), mRNA.	TOPK
219158_s_at	0.3	1.2	hypothetical protein FLJ13340	FLJ13340
219162_s_at	0.3	0.6	CGI-113 protein	LOC51017
219212_at	1.1	1.1	heat shock protein hsp70-related protein	LOC51182
219258_at	0.8	0.4	hypothetical protein FLJ20516	FLJ20516
219279_at	0.2	2.8	hypothetical protein FLJ20220	FLJ20220
219304_s_at	-1.2	-1.4	spinal cord-derived growth factor-B	SCDGF-B
219306_at	1.3	0.8	kinesin-like protein 2	hklp2
219347_at	0.6	0.1	hypothetical protein FLJ10956	FLJ10956
219449_s_at	0.3	1.7	hypothetical protein FLJ20533	FLJ20533
219493_at	1.3	0.9	hypothetical protein FLJ22009	FLJ22009
219496_at	0.8	1.1	hypothetical protein FLJ21870	FLJ21870
219553_at	0.7	0.5	Homo sapiens NME7 (NME7), mRNA.	NME7
219555_s_at	1.8	3.5	uncharacterized bone marrow protein BM039	BM039
219572_at	-1.7	-1.4	hypothetical protein FLJ20761	FLJ20761
219645_at	-1.3	-1.6	skeletal muscle calsequestrin 1	CASQ1
219732_at	-1	-1.4	hypothetical protein FLJ20300	FLJ20300
219757_s_at	-1	-0.1	hypothetical protein FLJ20392	FLJ20392
219759_at	1.2	0.6	aminopeptidase	LOC64167
219787_s_at	1	1.1	hypothetical protein FLJ10461	FLJ10461
219795_at	-1.1	-2.1	solute carrier family 6 (neurotransmittertransporter), member 14	SLC6A14
219905_at	0.9	2.5	hypothetical protein PRO2801	PRO2801
219918_s_at	1.4	1.3	hypothetical protein FLJ10517	FLJ10517
219932_at	0.5	1	very long-chain acyl-CoA synthetase homolog 1	VLCS-H1
219951_s_at	-1.1	-1.2	hypothetical protein FLJ10600	FLJ10600
219979_s_at	1	2.1	hypothetical protein	HSPC138
219998_at	0.6	0.4	HSPC159 protein	HSPC159
220115_s_at	-1.7	-1.2	cadherin 10, type 2 (T2-cadherin)	CDH10
220175_s_at	0.6	0.1	hypothetical protein from clone 1659351	LOC57397
220183_s_at	2.4	3.7	nudix (nucleoside diphosphate linked moietyX)-type motif 6	NUDT6
220193_at	-3.6	-1.3	hypothetical protein FLJ22938	FLJ22938
220239_at	0.3	0.1	SBBI26 protein	SBBI26
220329_s_at	1.1	0.5	hypothetical protein FLJ20627	FLJ20627
220403_s_at	0.8	0.5	p53-regulated apoptosis-inducing protein 1	P53AIP1
220494_s_at	1	3.6	lipopolysaccharide specific response-68 protein	LSR68
220533_at	-1.3	-1.4	hypothetical protein FLJ13385	FLJ13385
220625_s_at	-1.5	-2.1	Ets transcription factor ESE-2b	ELF5
220867_s_at	-1.2	-1	solute carrier family 24(sodiumpotassiumcalcium exchanger), member 2	SLC24A2
220897_at	-1.1	-1.5	hypothetical protein FLJ11556	FLJ11556
220925_at	0.8	1.4	hypothetical protein FLJ21613 similar to ratcorneal wound healing related protein	FLJ21613
220941_s_at	0.8	0.2	hypothetical protein LOC54149	YG81
221263_s_at	0.4	2.1	hypothetical protein MGC3133	MGC3133
221381_s_at	0.2	1.1	mortality factor 4	MORF4
221437_s_at	1.3	2.6	mitochondrial ribosomal protein S15	MRPS15
221452_s_at	1.1	1	hypothetical protein MGC1223	MGC1223
221464_at	-1.7	-2	olfactory receptor, family 1, subfamily D,member 2	OR1D2
221504_s_at	0.9	1.3	Vacuolar proton pump subunit SFD alpha isoform	LOC51606
221521_s_at	0.6	0.2	Homo sapiens, HSPC037 protein, clone MGC:673, mRNA, complete cds.	LOC51659
221524_s_at	1.1	1.2	Homo sapiens Rag D mRNA, complete cds.	RAGD
221547_at	0.7	0.4	pre-mRNA splicing factor similar to S.cerevisiae Prp18	PRP18
221580_s_at	0.6	1	Homo sapiens, clone MGC:5306, mRNA, complete cds.	MGC5306
221622_s_at	0.4	0.5	Homo sapiens HT026 mRNA, complete cds.	HT007
221702_s_at	0.3	1	BBP-like protein 2	BLP2
221748_s_at	-0.6	-1.3	tensin	TNS
221766_s_at	1	0.2	hypothetical protein FLJ20037	FLJ20037
221776_s_at	1.4	0.8	bromodomain-containing 7	BRD7
221803_s_at	0.2	0.7	nuclear receptor binding factor-2	NRBF-2
221823_at	0.4	0.3	Homo sapiens clone 23860 mRNA sequence	
221829_s_at	1	0.1	karyopherin (importin) beta 2	KPNB2

221916_at	-1.3	-2	hypothetical protein	DKFZp434B0417
221943_x_at	-0.8	-1.6	ribosomal protein L38	RPL38
221974_at	-0.8	-1	imprinted in Prader-Willi syndrome	IPW
221986_s_at	1	2.5	hypothetical protein FLJ20059	FLJ20059
221989_at	0.5	1.1	ribosomal protein L10	RPL10
222037_at	1.2	3.4	minichromosome maintenance deficient (S. cerevisiae) 4	MCM4
222040_at	0.4	0.9	heterogeneous nuclear ribonucleoprotein A1	HNRPA1
222077_s_at	1.1	0.6	GTPase activating protein	ID-GAP
222108_at	-1	-1.4	Human BAC clone GS1-99H8	
222113_s_at	-1	-1.3	epidermal growth factor receptor substrate EPS15R	EPS15R
222231_s_at	0.3	0.6	hypothetical protein PRO1855	PRO1855
222380_s_at	-0.8	-0.9	ESTs	
266_s_at	-1.9	-1.5	Homo sapiens CD24 signal transducer mRNA, complete cds and 3 region	
34031_i_at	0.6	1.6	Human Krit1 mRNA, complete cds	
36129_at	-0.6	-1.1	Homo sapiens KIAA0397 mRNA, complete cds	
36552_at	-0.6	-1.3	Homo sapiens mRNA; cDNA DKFZp586P0123 (from clone DKFZp586P0123)	
36830_at	1.2	0.6	Human mitochondrial intermediate peptidase precursor (MIPEP) mRNA, mitochondrial gene encoding mitochondrial protein, complete cds	
37547_at	-0.9	-2	Human clone IMAGE-22181 unknown protein mRNA, partial cds	
37577_at	0.9	0.9	Human clone 23719 mRNA sequence	
38241_at	1	3.2	Human butyrophilin (BTF3) mRNA, complete cds	
39705_at	-0.7	-0.9	Homo sapiens mRNA for KIAA0700 protein, partial cds	
39891_at	-1.1	-1	Cluster Incl. AI246730:qk40b01.x1 Homo sapiens cDNA, 3 end	
40016_g_at	-0.6	-1.8	Human mRNA for KIAA0303 gene, partial cds	
41387_r_at	-0.9	-0.9	Human mRNA for KIAA0346 gene, partial cds	
44702_at	-1	-1.3		
44783_s_at	-0.9	-1.3		
45749_at	-1	-1.3		
47550_at	-0.8	-0.9		
47571_at	-0.7	-1.1		
48031_r_at	-1.5	-1.7		
48808_at	0.8	1.5		
49327_at	-0.9	-1.1		
49878_at	-1.1	-0.8		
51158_at	-0.9	-1.2		
51774_s_at	-0.9	-0.8		
52285_f_at	0.5	0.2		
54632_at	-0.4	-1		
56256_at	-0.5	-1.5		
57739_at	-0.6	-1.2		
58780_s_at	-1.2	-1.6		
58900_at	-0.9	-1		
60471_at	-1	-1.1		
60528_at	-0.7	-1.2		
65635_at	-0.5	-1.1		
AFFX-hum_alu_at	-0.6	-1.1	Human Alu-Sq subfamily consensus sequence.	Alu-Sq
AFFX-M27830_M_at	-0.6	-1.1	Human 28S ribosomal RNA gene, complete cds.	28S rRNA

List of the entire genes that are differentially expressed in either $\alpha6^{+}$/MHCI^{+} cells or $\alpha6^{+}$/MHCI^{-} cells and are consistently upregulated or down regulated in both arrays. "–"sign indicates that the gene is upregulated in $\alpha6^{+}$/MHCI^{-} cells. The numbers that show the difference in the level of gene expression are in log2 scale.
