# Supplementary material for: Transcriptional profiling of putative human epithelial stem cells
Source: BMC Genomics. 2008 Jul 30;9:359. doi: 10.1186/1471-2164-9-359 (PMC2536675; doi:10.1186/1471-2164-9-359)
Supplement: Additional file 3 — List of the genes that are differentially expressed in α6+/MHCI+cells vs. α6+/MHCI- cells and also expressed in human hair follicle SCs (HHFSC). "-"sign indicates that the gene is upregulated in α6+/MHCI- cells. The numbers that show the difference in the level of gene expression are in log2 scale. [file 1471-2164-9-359-S3.pdf]

The Genes upregulated in human hair follicle bulge cells and in human alpha 6+/MHCI- cells

214774_x_at		-1.5	trinucleotide repeat containing 9	TNRC9
217996_at	-1		pleckstrin homology-like domain, family A, member 1	PHLDA1
204712_at	-6.6	-3.8	Wnt inhibitory factor-1	WIF-1
221127_s_at		-1.6	regulated in glioma	RIG
214247_s_at		-1	regulated in glioma	RIG
200762_at	-1.9	-1.9	dihydropyrimidinase-like 2	DPYSL2
209167_at	-2		glycoprotein M6B	GPM6B
209168_at	-1.5	-1.3	glycoprotein M6B	GPM6B
209169_at	-1.9		glycoprotein M6B	GPM6B
209170_s_at	-2.5	-1.7	glycoprotein M6B	m6b1
201431_s_at	-1.4		dihydropyrimidinase-like 3	DPYSL3
204451_at	-0.7	-1.3	frizzled (Drosophila) homolog 1	FZD1
210162_s_at	-1.1	-1.7	NF-ATc mRNA	NFATC1
207345_at	-2	-2.3	follistatin, transcript variant FST317	FST
204948_s_at	-1.1	-1.9	follistatin, transcript variant FST344	FST
205338_s_at	-2.2		dopachrome tautomerase (dopachrome delta-isomerase, tyrosine-related protein 2)	DCT
205337_at	-2.5	-1.2	dopachrome tautomerase (dopachrome delta-isomerase, tyrosine-related protein 2)	DCT
216512_s_at	-1.1		dopachrome tautomerase (dopachrome delta-isomerase, tyro	DCT

The Genes upregulated in human hair follicle bulge cells

but down regulated in human alpha 6+/MHCI- cells

202196_s_at		2.5	dickkopf (Xenopus laevis) homolog 3	DKK3
204734_at		1.2	keratin 15	KRT15

The Genes downregulated in human hair follicle bulge cells and in human alpha 6+/MHCI- cells

203213_at	1.7	1.5	cell division cycle 2, G1 to S and G2 to M	CDC2
203214_x_at	1.4		cell division cycle 2, G1 to S and G2 to M	CDC2
210559_s_at	1.2		CDC2 delta T	CDC2
218009_s_at	1.6	0.9	protein regulator of cytokinesis 1	PRC1
209773_s_at	1.4	2.3	ribonucleotide reductase M2 polypeptide	RRM2
201890_at	1.1	0.7	ribonucleotide reductase M2 polypeptide	RRM2
204026_s_at	1.1	0.9	ZW10 interactor	ZWINT
211762_s_at	1.1	0.7	karyopherin alpha 2 (RAG cohort 1, importinalpha 1)	
204767_s_at	1.1		flap structure-specific endonuclease 1	FEN1
201291_s_at	4.5	2.1	topoisomerase (DNA) II alpha (170kD)	TOP2A
201292_at	2	2.1	topoisomerase (DNA) II alpha (170kD)	TOP2A
202589_at	1	0.4	thymidylate synthetase	TYMS
201453_x_at		1.2	Ras homolog enriched in brain 2	RHEB2

The Genes that are downregulated in human hair follicle bulge cells but upregulated in human alpha 6+/MHCI- cells

206935_at		-1.8	protocadherin 8	PCDH8
209884_s_at	-1.5		sodium bicarbonate cotransporter 3	SLC4A7
37892_at		-3.7	alpha-1 type XI collagen	COL11A1
211340_s_at	-1	-1.5	MUC18 glycoprotein mRNA	MCAM
204682_at	-0.9	-1	latent transforming growth factor beta binding protein 2	LTBP2
204273_at	-2.8	-1.3	endothelin receptor type B	EDNRB
208650_s_at	-1.5	-1.1	CD24 antigen (small cell lung carcinoma cluster 4 antigen)	CD24
209771_x_at	-1.8	-1.6	CD24 antigen (small cell lung carcinoma cluster 4 antigen)	CD24
266_s_at	-1.9	-1.5	CD24 signal transducer	
208651_x_at	-1.8		signal transducer CD24	CD24
216379_x_at	-1.8	-1.6	CD24 signal transducer	

List of the genes that are differentially expressed in α6^{+}/MHCI^{+} cells vs. α6^{+}/MHCI^{-} cells and also expressed in human hair follicle SCs (HHFSC). "–"sign indicates that the gene is upregulated in α6^{+}/MHCI^{-} cells. The numbers that show the difference in the level of gene expression are in log2 scale.
