# Supplementary material for: Transcriptional profiling of putative human epithelial stem cells
Source: BMC Genomics. 2008 Jul 30;9:359. doi: 10.1186/1471-2164-9-359 (PMC2536675; doi:10.1186/1471-2164-9-359)
Supplement: Additional file 4 — List of the genes that are differentially expressed in α6+/MHCI+cells vs. α6+/MHCI- cells and also expressed in murine hair follicle SCs (MHFSC). "-"sign indicates that the gene is upregulated in α6+/MHCI- cells. The numbers that show the difference in the level of gene expression are in log2 scale. [file 1471-2164-9-359-S4.pdf]

Genes that are upregulated in mouse keratinocyte SCs and in human alpha 6+/MHCI- cells

206140_at	-5	-4.7	LIM homeobox protein 2	LHX2
213730_x_at		-1	transcription factor 3 (E2A immunoglobulin enhancer binding factors E12E47)	TCF3
209153_s_at		-1.3	transcription factor 3 (E2A immunoglobulin enhancer binding factors E12E47)	TCF3
221016_s_at	-1.5		HMG-box transcription factor TCF-3	TCF3
204712_at	-6.6	-3.8	Wnt inhibitory factor-1	WIF-1
218502_s_at	-2	-1.6	trichorhinophalangeal syndrome I gene	TRPS1
221234_s_at	-1.1		BTB and CNC homology 1, basic leucine zipper transcription factor 2	BACH2
212865_s_at	-1.3	-1.1	collagen, type XIV, alpha 1 (undulin)	COL14A1
202729_s_at	-0.8	-1.1	latent transforming growth factor beta binding protein 1	LTBP1
201431_s_at	-1.4		dihydropyrimidinase-like 3	DSLY3
213880_at	-1.6	-2	G protein-coupled receptor 49	GPR49
209291_at		-1	inhibitor of DNA binding 4, dominant negative helix-loop-helix protein	ID4
205066_s_at	-1.6	-2.1	ectonucleotide pyrophosphatasephosphodiesterase 1	ENPP1
203184_at	-1		fibrillin 2 (congenital contractural arachnodactyly)	FBN2
220048_at	P		ectodysplasin 1, anhidrotic receptor	EDAR
213260_at	-1	-1.7	forkhead box C1	FOXC1
201426_s_at	-1.7		vimentin	VIM
206028_s_at	-1.5		c-mer proto-oncogene tyrosine kinase	MERTK
205489_at	-1	-1.5	crystallin, mu	CRYM
213436_at	-1.4		cannabinoid receptor 1 (brain)	CNR1
200832_s_at	-2		Scd mRNA for stearoyl-CoA desaturase	Scd
205338_s_at	-2.2		dopachrome tautomerase (dopachrome delta-isomerase, tyrosine-related protein 2)	DCT
205337_at	-2.5	-1.2	dopachrome tautomerase (dopachrome delta-isomerase, tyrosine-related protein 2)	DCT
216512_s_at	-1.1		dopachrome tautomerase (dopachrome delta-isomerase, tyrosine-related protein 2)	DCT
204273_at	-2.8	-1.3	endothelin receptor type B	EDNRB
204121_at	-1.2		growth arrest and DNA-damage-inducible, gamma	GADD45G
210365_at	-1.8	-1.5	AML1 mRNA for AML1a protein (alternatively spliced product)	AML1
209360_s_at	-2.3	-1.4	AML1 mRNA for AML1b protein (alternatively spliced product)	AML1
205255_x_at	-1.1		transcription factor 7 (T-cell specific, HMG-box)	TCF7
201117_s_at	-1.9	-1.3	carboxypeptidase E precursor	CPE
209167_at	-2		glycoprotein M6B	GPM6B
209168_at	-1.5	-1.3	glycoprotein M6B	GPM6B
209169_at	-1.9		glycoprotein M6B	GPM6B
209170_s_at	-2.5	-1.7	glycoprotein M6B	m6b1
211535_s_at		-2.5	fibroblast growth factor receptor	FGFR1
220792_at	-1.5		PR domain containing 5	PRDM5
210105_s_at	-1.6	-1.8	FYN oncogene related to SRC, FGR, YES	FYN
201163_s_at	-2.2	-1.6	insulin-like growth factor binding protein 7	IGFBP7
210162_s_at	-1.1	-1.7	NF-ATc mRNA	NFATC1
204736_s_at		-2.1	melanoma-associated chondroitin sulfateproteoglycan 4	CSPG4
221731_x_at	-1.5		chondroitin sulfate proteoglycan 2 (versican)	CSPG2
204620_s_at	-2	-1.4	chondroitin sulfate proteoglycan 2 (versican)	CSPG2
203945_at		-1.4	arginase, type II, nuclear gene encoding mitochondrial protein, mRNA	ARG2
209583_s_at	-2.9	-1.5	brain my033 protein mRNA	MOX2
207147_at		-1.8	distal-less homeo box 2	DLX2
219935_at	-1.6		a disintegrin-like and metalloprotease (reprolysin type) with thrombospondin type 1 motif, 5 (aggrecanase-2)	ADAMTS5
217996_at	-1		pleckstrin homology-like domain, family A, member 1	PHLDA1
203706_s_at	-1.1	-1.5	frizzled (Drosophila) homolog 7	FZD7
203817_at		-1.5	guanylate cyclase 1, soluble, beta 3	GUCY1B3
206630_at	-2.1		tyrosinase (oculocutaneous albinism IA)	TYR
202403_s_at	-1.3	-2	collagen, type I, alpha 2	COL1A2
206002_at	-2.1	-2.3	G protein-coupled receptor 64	GPR64
205752_s_at	-1	-1.2	glutathione S-transferase M5	GSTM5
212226_s_at		-1.2	phosphatidic acid phosphatase type 2B	PPAP2B
212230_at	-1.6	-1.3	phosphatidic acid phosphatase type 2B	PPAP2B
207233_s_at	-1.8		microphthalmia-associated transcription factor	MITF
219511_s_at		-1.2	synuclein alpha interacting protein	SNCAIP
213931_at	-1		inhibitor of DNA binding 2, dominant negative helix-loop-helix protein	ID2
211343_s_at		-1.8	(clones HT-(125,133)) alpha-2 type IV collagen	COL4A2
202936_s_at	-1.1	-0.9	SRY (sex determining region Y)-box 9 (campomelic dysplasia, autosomal sex-reversal)	SOX9
202935_s_at	-1.9		SRY (sex determining region Y)-box 9 (campomelic dysplasia, autosomal sex-reversal)	SOX9
212372_at		-1.1	Homo sapiens cDNA: FLJ23324 fis, clone HEP12482, highly similar to HUMMYOHCB Human nonmuscle myosin heavy chain-B (MYH10) mRNA.	
207069_s_at		-1.6	MAD (mothers against decapentaplegic, Drosophila) homolog 6	MADH6

201626_at		-1.2	insulin induced gene 1	INSIG1
201860_s_at	-1.4		plasminogen activator, tissue	PLAT
200762_at	-1.9	-1.9	dihydropyrimidinase-like 2	DPYSL2
209242_at	-0.9	-1.2	paternally expressed 3	PEG3
213764_s_at		-2.8	paternally expressed 3	PEG3
211466_at	-1.2	-1.1	nuclear factor I B3 mRNA,	NFIB
201278_at	-1.9		disabled (Drosophila) homolog 2 (mitogen-responsive phosphoprotein)	DAB2
211959_at	-0.9	-1.1	Human insulin-like growth factor binding protein 5	IGFBP5
202746_at	-1.2	-0.8	integral membrane protein 2A	ITM2A
205696_s_at		-1.5	GDNF family receptor alpha 1	GFRA1
201952_at		-1.6	activated leucocyte cell adhesion molecule	ALCAM
206382_s_at		-2.6	brain-derived neurotrophic factor	BDNF
203666_at	-1.1		stromal cell-derived factor 1	SDF1
211161_s_at	-1		PRO3121	COL3A1
211980_at	-1.9	-1.2	collagen, type IV, alpha 1	COL4A1
211343_s_at		-1.8	(clones HT-(125,133)) alpha-2 type IV collagen	COL4A2
203325_s_at		-1.1	collagen, type V, alpha 1	COL5A1
213428_s_at	-0.7	-1.1	collagen, type VI, alpha 1	COL6A1
201645_at	-1.3		hexabrachion (tenascin C, cytotactin)	HXB
213680_at		P	keratin 6A	KRT6A
204682_at	-0.9	-1	latent transforming growth factor beta binding protein 2	LTBP2
208636_at	-0.7	-1.1	actinin, alpha 1	ACTN1
203914_x_at	-1.1		hydroxyprostaglandin dehydrogenase 15-(NAD)	HPGD
200602_at	-0.6	-1.2	amyloid beta (A4) precursor protein (protease nexin-II, Alzheimer disease)	APP
210835_s_at		-1	ribeye mRNA	CTBP2
32811_at	-1.1		myosin-I beta	MYO1B
203759_at	-1.5		sialyltransferase 4C (beta-galactosidase alpha-2,3-sialytransferase)	SIAT4C
202668_at		-1.3	ephrin-B2	EFNB2
204037_at		-1.2	endothelial differentiation, lysophosphatidic acid G-protein-coupled receptor, 2	EDG2
202436_s_at	-2.2	-1.2	cytochrome P450, subfamily I (dioxin-inducible), polypeptide 1 (glaucoma 3, primary infantile	CYP1B1
202435_s_at	-2.3		cytochrome P450, subfamily I (dioxin-inducible), polypeptide 1 (glaucoma 3, primary infantile	CYP1B1
202437_s_at	-3.5	-2.7	cytochrome P450, subfamily I (dioxin-inducible), polypeptide 1 (glaucoma 3, primary infantile	CYP1B1
206346_at	-1.1		prolactin receptor	PRLR
213591_at		-1.1	aldehyde dehydrogenase 7 family, member A1	ALDH7A1
205399_at		-1.6	doublecortin and CaM kinase-like 1	DCAMKL1
214607_at		-1.7	p21 (CDKN1A)-activated kinase 3	PAK3

Genes that are upregulated in mouse keratinocyte SCs but downregulated in human alpha 6+/MHCI- cells (upregulated in human alpha 6+/MHCI+ cells)

208310_s_at	1.1		follistatin-like 1	FSTL1
208893_s_at		3.5	clone MGC:12852, mRNA	DUSP6
217764_s_at	1	0.5	small GTP-binding protein rab22b mRNA	RAB31
202206_at	0.5	1.6	ADP-ribosylation factor-like 7	ARL7
202196_s_at		2.5	dickkopf (Xenopus laevis) homolog 3	DKK3
205289_at		1.3	bone morphogenetic protein 2	BMP2
201970_s_at	1.7	2.9	nuclear autoantigenic sperm protein (histone-binding)	NASP
205081_at		1.4	cysteine-rich protein 1 (intestinal)	CRIP1
221524_s_at	1.1	1.2	Rag D	RAGD
214693_x_at		4.4	phosphatidic acid phosphatase type 2A	PPAP2A
208864_s_at	1	1	thioredoxin	TXN
212249_at		1	phosphoinositide-3-kinase, regulatory subunit, polypeptide 1 (p85 alpha)	PIK3R1
201802_at	1.3		solute carrier family 29 (nucleoside transporters), member 1	SLC29A1
205909_at	1.4	0.6	polymerase (DNA directed), epsilon 2	POLE2
210809_s_at		1.1	osteoblast specific factor 2	osf-2
209129_at		1.4	Human zyxin related protein ZRP-1 mRNA, complete cds.	TRIP6
200626_s_at	-0.5	1.4	matrin 3	MATR3

Genes that are downregulated in both mouse keratinocyte SCs and in human alpha 6+/MHCI- cells

203888_at	1.1		thrombomodulin	THBD
203748_x_at		4.2	RNA binding motif, single stranded interacting protein 1 (RBMS1), transcript variant MSSP-2	RBMS1
207266_x_at		3.5	RNA binding motif, single stranded interacting protein 1 (RBMS1), transcript variant MSSP-3	RBMS1
202431_s_at		1	v-myc avian myelocytomatosis viral oncogene homolog	MYC
203981_s_at		1.8	ATP-binding cassette, sub-family D (ALD), member 4	ABCD4
205480_s_at	0.7	1.6	UDP-glucose pyrophosphorylase 2	UGP2
212143_s_at	1.1		insulin-like growth factor binding protein 3	IGFBP3
208606_s_at	0.7	3.6	wingless-type MMTV integration site family, member 4	WNT4
204750_s_at		1.8	desmocollin 2	DSC2

200800_s_at		2.4	heat shock 70kD protein 1A	HSPA1A
202712_s_at	1	0.8	creatine kinase, mitochondrial 1 (ubiquitous), nuclear gene encoding mitochondrial protein, mRNA	CKMT1
209305_s_at		1.4	growth arrest and DNA-damage-inducible proteinGADD45beta	GADD45B
210426_x_at	0.6	2.4	orphan hormone nuclear receptor RORalpha1	RORA
		1.8	annexin A2 / Human lipocortin (LIP) 2 pseudogene mRNA, complete cds-like region	ANXA2
204636_at	0.4	2.2	collagen, type XVII, alpha 1	COL17A1
204863_s_at		1.7	interleukin 6 signal transducer (gp130, oncostatin M receptor)	IL6ST
201012_at		1.4	annexin I	ANXA1
208944_at		1.3	TGF-betaIIR alpha	TGFBR2
206400_at	0.3	1.8	lectin, galactoside-binding, soluble, 7 (galectin 7)	LGALS7
201820_at	1		keratin 5 (epidermolysis bullosa simplex, Dowling-MearaKobnerWeber-Cockayne types)	KRT5
204734_at		1.2	keratin 15	KRT15
204614_at		1	serine (or cysteine) proteinase inhibitor, cladeB (ovalbumin), member 2	SERPINB2
206421_s_at	1.8	4	serine (or cysteine) proteinase inhibitor, clade B (ovalbumin), member 7	SERPINB7
219936_s_at		1.3	G protein-coupled receptor 87	GPR87
201506_at	1.2	1.3	transforming growth factor, beta-induced, 68kD	TGFBI
201487_at	P	P	cathepsin C	CTSC
203798_s_at	1.2	0.9	visinin-like 1	VSNL1
206165_s_at		1.7	calcium activated chloride channel 2 precursor	CLCA2
206276_at	2	0.5	lymphocyte antigen 6 complex, locus D	E48
212022_s_at		2	antigen identified by monoclonal antibody Ki-67	MKI67
204170_s_at	1.1	0.7	CDC28 protein kinase 2	CKS2
218009_s_at	1.6	0.9	protein regulator of cytokinesis 1	PRC1
211911_x_at	1.1		MHC class I HLA B71	HLA-B
217456_x_at		1.1	MHC class I HLA-B51	HLA-B
208729_x_at	2.4		major histocompatibility complex, class I, B	HLA-B
209140_x_at	1	2	MHC HLA-B39	HLA-B39
214459_x_at	0.7	1.1	Cw1 antigen mRNA	HLA-C
211799_x_at	2.9		HLA class I heavy chain	HLA-Cw*1701
200905_x_at		1	major histocompatibility complex, class I, E	HLA-E
200904_at	0.6	1.6	H.sapiens HLA-E gene.	HLA-E
200041_s_at	1.7	1	HLA-B associated transcript-1	D6S81E
212384_at		1	HLA-B associated transcript-1	D6S81E
221455_s_at		3.8	wingless-type MMTV integration site family, member 3	WNT3
214710_s_at	2.2	1.8	cyclin B1	CCNB1
202705_at	1.7	0.6	cyclin B2	CCNB2
208711_s_at		1.3	cyclin D1 (PRAD1: parathyroid adenomatosis 1)	CCND1
200951_s_at		3.3	cyclin D2	CCND2
205393_s_at	1.7		CHK1 (checkpoint, S.pombe) homolog	CHEK1
203968_s_at	1.6		CDC6 (cell division cycle 6, S. cerevisiae) homolog	CDC6

Genes that are downregulated in mouse keratinocyte SCs but upregulated in human alpha 6+/MHCI- cells (downregulated in human alpha 6+/MHCI+ cells)

219773_at	-1		NADPH oxidase 4	NOX4
213348_at	-1.1		cyclin-dependent kinase inhibitor 1C (p57, Kip2)	CDKN1C
213183_s_at	-1.5	-2.1	cyclin-dependent kinase inhibitor 1C (p57, Kip2)	CDKN1C
222043_at		-1.2	clusterin (complement lysis inhibitor, SP-40,40, sulfated glycoprotein 2, testosterone-repressed prostate message 2, apolipoprotein J)	CLU
218313_s_at	-1.1		UDP-N-acetyl-alpha-D-galactosamine:polypeptide N-acetylgalactosaminyltransferase 7 (GalNAc-T7)	GALNT7
204869_at	-1.6	-1.7	proprotein convertase subtilisinkexin type 2	PCSK2
213560_at		-1.3	growth arrest and DNA-damage-inducible, beta	GADD45B
207002_s_at	-1		pleiomorphic adenoma gene-like 1 isoform 1	PLAGL1
207943_x_at	-1		pleiomorphic adenoma gene-like 1 isoform 1	PLAGL1
205372_at	-0.6	-1.3	pleiomorphic adenoma gene-like 1 isoform 1	PLAGL1
205861_at	-1		Spi-B transcription factor (Spi-1PU.1 related)	SPIB
202820_at	-1		aryl hydrocarbon receptor	AHR
209120_at		-1	nuclear receptor subfamily 2, group F, member 2	NR2F2
202002_at		-1.5	acetyl-Coenzyme A acyltransferase 2 (mitochondrial 3-oxoacyl-Coenzyme A thiolase)	ACAA2
204584_at		-2.5	L1 cell adhesion molecule (hydrocephalus, stenosis of aqueduct of Sylvius 1, MASA (mental retardation, aphasia, shuffling gait and adducted thumbs) syndrome, spastic paraplegia 1)	L1CAM
202019_s_at	-1.1		LanC (bacterial lantibiotic synthetase component C)-like 1	LANCL1
202709_at	-1		fibromodulin precursor	FMOD
221963_x_at	-1.1		thrombospondin 1	THBS1
201110_s_at	-2.3	-1.9	thrombospondin 1	THBS1
205590_at	-1.3	-2.6	RAS guanyl releasing protein 1 (calcium and DAG-regulated)	RASGRP1

List of the genes that are differentially expressed in $\alpha6^{+}$/$MHCI^{+}$ cells vs. $\alpha6^{+}$/$MHCI^{-}$ cells and also expressed in murine hair follicle SCs (MHFSC). "–"sign indicates that the gene is upregulated in $\alpha6^{+}$/$MHCI^{-}$ cells. The numbers that show the difference in the level of gene expression are in log2 scale.
