# Supplementary material for: Transcriptional profiling of putative human epithelial stem cells
Source: BMC Genomics. 2008 Jul 30;9:359. doi: 10.1186/1471-2164-9-359 (PMC2536675; doi:10.1186/1471-2164-9-359)
Supplement: Additional file 5 — Expression of TCF3 targets in α6+/MHCI- and α6+/MHCI+cells. The table shows the difference in the expression in α6+/MHCI+ cells vs. α6+/MHCI- cells. "-"sign indicates that the gene is upregulated in α6+/MHCI- cells. The numbers that show the difference in the level of gene expression are in log2 scale. [file 1471-2164-9-359-S5.pdf]

Tcf3 and its targets

The genes that are upregulated by Tfc3 and also upregulated in human alpha 6+/MHCI- cells

213730_x_at		-1	transcription factor 3 (E2A immunoglobulin enhancer binding factors E12E47)	TCF3
209153_s_at		-1.3	transcription factor 3 (E2A immunoglobulin enhancer binding factors E12E47)	TCF3
221016_s_at	-1.5		HMG-box transcription factor TCF-3	TCF3
213348_at	-1.1		cyclin-dependent kinase inhibitor 1C (p57, Kip2)	CDKN1C
213183_s_at	-1.5	-2.1	cyclin-dependent kinase inhibitor 1C (p57, Kip2)	CDKN1C
201538_s_at	-1.3		dual specificity phosphatase 3 (vaccinia virus phosphatase VH1-related)	DUSP3
202806_at		-1.5	drebrin 1	DBN1
206580_s_at		-1.2	EGF-containing fibulin-like extracellular matrix protein 2	EFEMP2
204154_at	-1.4	-1.1	cysteine dioxygenase, type I	CDO1
204736_s_at		-2.1	melanoma-associated chondroitin sulfateproteoglycan 4	CSPG4
214297_at		-2.1	chondroitin sulfate proteoglycan 4 (melanoma-associated)	CSPG4
211959_at	-0.9	-1.1	Human insulin-like growth factor binding protein 5	IGFBP5
213993_at		-3.9	spondin 1, (f-spondin) extracellular matrix protein	SPON1
219883_at	-1.3		potassium inwardly-rectifying channel, subfamily K, member 4	KCNK4
202686_s_at	-0.8	-1.6	AXL receptor tyrosine kinase isoform 1precursor	AXL
205064_at	-1.5	-1.3	small proline-rich protein 1B (cornifin)	SPRR1B
208161_s_at		-1.1	Homo sapiens ATP-binding cassette, sub-family C (CFTRMRP), member 3 (ABCC3), transcript variant MRP3A, mRNA.	ABCC3

The genes that are downregulated by Tfc3 and upregulated in human alpha 6+/MHCI+ cells

205809_s_at		3.3	Wiskott-Aldrich syndrome-like	WASL
205891_at	0.5	1.5	adenosine A2b receptor	ADORA2B
210426_x_at	0.6	2.4	orphan hormone nuclear receptor RORalpha1	RORA
200951_s_at		3.3	cyclin D2	CCND2
206165_s_at		1.7	calcium activated chloride channel 2 precursor	CLCA2
204822_at	1.1	1.1	TTK protein kinase	TTK
217165_x_at		1.6	metallothionein 1F (functional)	MT1F
204745_x_at		1.1	metallothionein 1G	MT1G
203560_at	0.8	1.2	gamma-glutamyl hydrolase (conjugase,folylpolygammaglutamyl hydrolase) precursor	GGH

Expression of TCF3 targets in $\alpha6^{+}$/MHCI^{-} and $\alpha6^{+}$/MHCI^{+} cells. The table shows the difference in the expression in $\alpha6^{+}$/MHCI^{+} cells vs. $\alpha6^{+}$/MHCI^{-} cells. "–"sign indicates that the gene is upregulated in $\alpha6^{+}$/MHCI^{-} cells. The numbers that show the difference in the level of gene expression are in log2 scale.
