# Supplementary material for: Transcriptional profiling of putative human epithelial stem cells
Source: BMC Genomics. 2008 Jul 30;9:359. doi: 10.1186/1471-2164-9-359 (PMC2536675; doi:10.1186/1471-2164-9-359)
Supplement: Additional file 6 — Expression of MYC targets in α6+/MHCI- and α6+/MHCI+cells. The table shows the difference in the expression in α6+/MHCI+ cells vs. α6+/MHCI- cells. "-"sign indicates that the gene is upregulated in α6+/MHCI- cells. The numbers that show the difference in the level of gene expression are in log2 scale. [file 1471-2164-9-359-S6.pdf]

Genes that are upregulated by c-myc transcription factor and upregulated in human alpha 6+/MHCI+ cells

202431_s_at		1	v-myc avian myelocytomatosis viral oncogene homolog	MYC
214710_s_at	2.2	1.8	cyclin B1	CCNB1
200951_s_at		3.3	cyclin D2	CCND2
207076_s_at	0.6	1.6	argininosuccinate synthetase	ASS
207332_s_at	0.6	0.6	transferrin receptor (p90, CD71)	TFRC
200806_s_at	1.8	1.7	heat shock 60kD protein 1 (chaperonin)	HSPD1
200692_s_at	1.3	2.7	heat shock 70kD protein 9B (mortalin-2)	HSPA9B
200800_s_at		2.4	heat shock 70kD protein 1A	HSPA1A
211968_s_at	1.6	2.2	heat shock 90kD protein 1, alpha	HSPCA
214359_s_at	1.3		heat shock 90kD protein 1, beta	HSPCB
221699_s_at	1.2		nucleolar protein GU2	
209567_at		1.5	Similar to regulator for ribosome resistance homolog (S. cerevisiae)	KIAA0112
201530_x_at		1	eukaryotic translation initiation factor 4A, isoform 1	EIF4A1
203712_at	1.1	0.9	KIAA0020 gene product	KIAA0020
212018_s_at		1.6	Homo sapiens cDNA FLJ20815 fis, clone ADSE01038, highly similar to AJ007398 Homo sapiens mRNA for PBK1 protein.	DKFZP564 M182
202532_s_at		1.1	dihydrofolate reductase	DHFR
201014_s_at	1.4	2	multifunctional polypeptide similar to SAICARsynthetase and AIR carboxylase	ADE2H1
203856_at	1	0.4	vaccinia related kinase 1	VRK1
202483_s_at		3.4	RAN binding protein 1	RANBP1
201923_at		1.3	thioredoxin peroxidase (antioxidant enzyme)	AOE372
201258_at	0.6	2.4	ribosomal protein S16	RPS16
222037_at	1.2	3.4	minichromosome maintenance deficient (S. cerevisiae) 4	MCM4
212141_at	1		minichromosome maintenance deficient (S. cerevisiae) 4	MCM4
204023_at	1.1	-0.1	replication factor C (activator 1) 4 (37kD)	RFC4
204102_s_at		2.1	translation elongation factor 2	EEF2
213649_at	1	1.6	splicing factor, arginineserine-rich 7 (35kD)	SFRS7
214141_x_at		1.2	splicing factor, arginineserine-rich 7 (35kD)	SFRS7
201273_s_at		1.6	signal recognition particle 9kD	SRP9
208864_s_at	1	1	Homo sapiens thioredoxin mRNA, complete cds.	TXN
200937_s_at		3.2	ribosomal protein L5	RPL5
221989_at	0.5	1.1	ribosomal protein L10	RPL10
203034_s_at		1.1	ribosomal protein L27a	RPL27a
209203_s_at		2.1	ribosomal protein L30	RPL30
200002_at		1.7	ribosomal protein L35	RPL35
217747_s_at	0.3	1	ribosomal protein S9	RPS9
201258_at	0.6	2.4	ribosomal protein S16	RPS16
213414_s_at		1.6	ribosomal protein S19	RPS19
205081_at		1.4	cysteine-rich protein 1 (intestinal)	CRIP1
203213_at	1.7	1.5	cell division cycle 2, G1 to S and G2 to M	CDC2
203214_x_at	1.4		cell division cycle 2, G1 to S and G2 to M	CDC2
210559_s_at	1.2		CDC2 delta T	CDC2
200853_at	0.8	1.3	H2A histone family, member Z	H2AFZ
207168_s_at		1	H2A histone family, member Y	H2AFY
217871_s_at	0.4	1.8	macrophage migration inhibitory factor (glycosylation-inhibiting factor)	MIF
212085_at		1.6	solute carrier family 25 (mitochondrial carrier; adenine nucleotide translocator), member 6	SLC25A6
208900_s_at		1.9	topoisomerase (DNA) I	TOP1
201577_at	1.2	1.2	non-metastatic cells 1 protein	NME1
204695_at	2.6		cell division cycle 25A	CDC25A
201030_x_at	0.6	1	lactate dehydrogenase B	LDHB
200920_s_at	0.6	3.5	B-cell translocation gene 1, anti-proliferative	BTG1
208930_s_at		1.7	interleukin enhancer binding factor 3, 90kD	ILF3
209330_s_at	1.3	0.9	heterogeneous nuclear ribonucleoprotein D	HNRPD
210949_s_at		1.4	Similar to eukaryotic translation initiation factor 3, subunit 8 (110kD)	EIF3S8
200841_s_at		2.5	glutamyl-prolyl-tRNA synthetase	EPRS
204531_s_at	1	0.1	breast cancer 1, early onset	BRCA1
209421_at	0.7	1.3	mutS (E. coli) homolog 2 (colon cancer, nonpolyposis type 1)	hMSH2
200973_s_at	1.2		tetraspan 3	TSPAN-3
218671_s_at	1.1		ATPase inhibitor precursor	LOC51189
200728_at	0.7	2	ARP2 (actin-related protein 2, yeast) homolog	ACTR2
213047_x_at	0.4		SET translocation (myeloid leukemia-associated)	SET
216520_s_at		2.6	tumor protein, translationally-controlled 1	TPT1
200596_s_at		1.7	eukaryotic translation initiation factor 3, subunit 10	EIF3S10
208816_x_at		1.8	annexin A2 / Human lipocortin (LIP) 2 pseudogene mRNA, complete cds-like region	ANXA2
201820_at	1		keratin 5 (epidermolysis bullosa simplex, Dowling-MearaKobnerWeber-Cockayne types)	KRT5
201305_x_at	1		acidic protein rich in leucines	SSP29
204278_s_at		1.6	Homo sapiens estrogen receptor binding site associated, antigen, 9 (EBAG9), mRNA	EBAG9

Genes that are downregulated by c-myc transcription factor and downregulated in human alpha 6+/MHCI+ cells

213931_at	-1		inhibitor of DNA binding 2, dominant negative helix-loop-helix protein	ID2
206132_at	-1.1		mutated in colorectal cancers	MCC

218637_at	-0.9	-1.1	hypothetical protein IMPACT	IMPACT
221963_x_at	-1.1		thrombospondin 1	THBS1
201110_s_at	-2.3	-1.9	thrombospondin 1	THBS1
202311_s_at		-2	collagen, type I, alpha 1	COL1A1
202403_s_at	-1.3	-2	collagen, type I, alpha 2	COL1A2
202404_s_at		-3.7	collagen, type I, alpha 2	COL1A2
211161_s_at	-1		PRO3121	COL3A1
211980_at	-1.9	-1.2	collagen, type IV, alpha 1	COL4A1
203325_s_at		-1.1	collagen, type V, alpha 1	COL5A1
213428_s_at	-0.7	-1.1	collagen, type VI, alpha 1	COL6A1
203749_s_at		-1.3	retinoic acid receptor, alpha	RARA
214023_x_at		-1.5	tubulin, beta polypeptide	TUBB
213275_x_at	-1.4	-1	cathepsin B	CTSB
202686_s_at	-0.8	-1.6	AXL receptor tyrosine kinase isoform 1precursor	AXL
204115_at		-1.1	guanine nucleotide binding protein 11	GNG11
221372_s_at		-1.8	purinergic receptor P2X, ligand-gated ionchannel, 2	P2RX2
212732_at		-1.7	maternally expressed 3	MEG3
222043_at		-1.2	clusterin (complement lysis inhibitor, SP-40,40, sulfated glycoprotein 2, testosterone-repressed prostate message 2, apolipoprotein J)	CLU
210195_s_at	-1		Human pregnancy-specific beta-1-glycoprotein mRNA PSG95, complete cds.	PSG1

Expression of MYC targets in $\alpha 6^+$/MHCI$^-$ and $\alpha 6^+$/MHCI$^+$ cells. The table shows the difference in the expression in $\alpha 6^+$/MHCI$^+$ cells vs. $\alpha 6^+$/MHCI$^-$ cells. "–"sign indicates that the gene is upregulated in $\alpha 6^+$/MHCI$^-$ cells. The numbers that show the difference in the level of gene expression are in log2 scale.
