# Supplementary material for: Transcriptional profiling of putative human epithelial stem cells
Source: BMC Genomics. 2008 Jul 30;9:359. doi: 10.1186/1471-2164-9-359 (PMC2536675; doi:10.1186/1471-2164-9-359)
Supplement: Additional file 7 — Flow cytometry data of sorted cells that donated RNA for microarray experiments. [file 1471-2164-9-359-S7.pdf]

Flow cytometry data of sorted cells that donated RNA for microarray experiments.

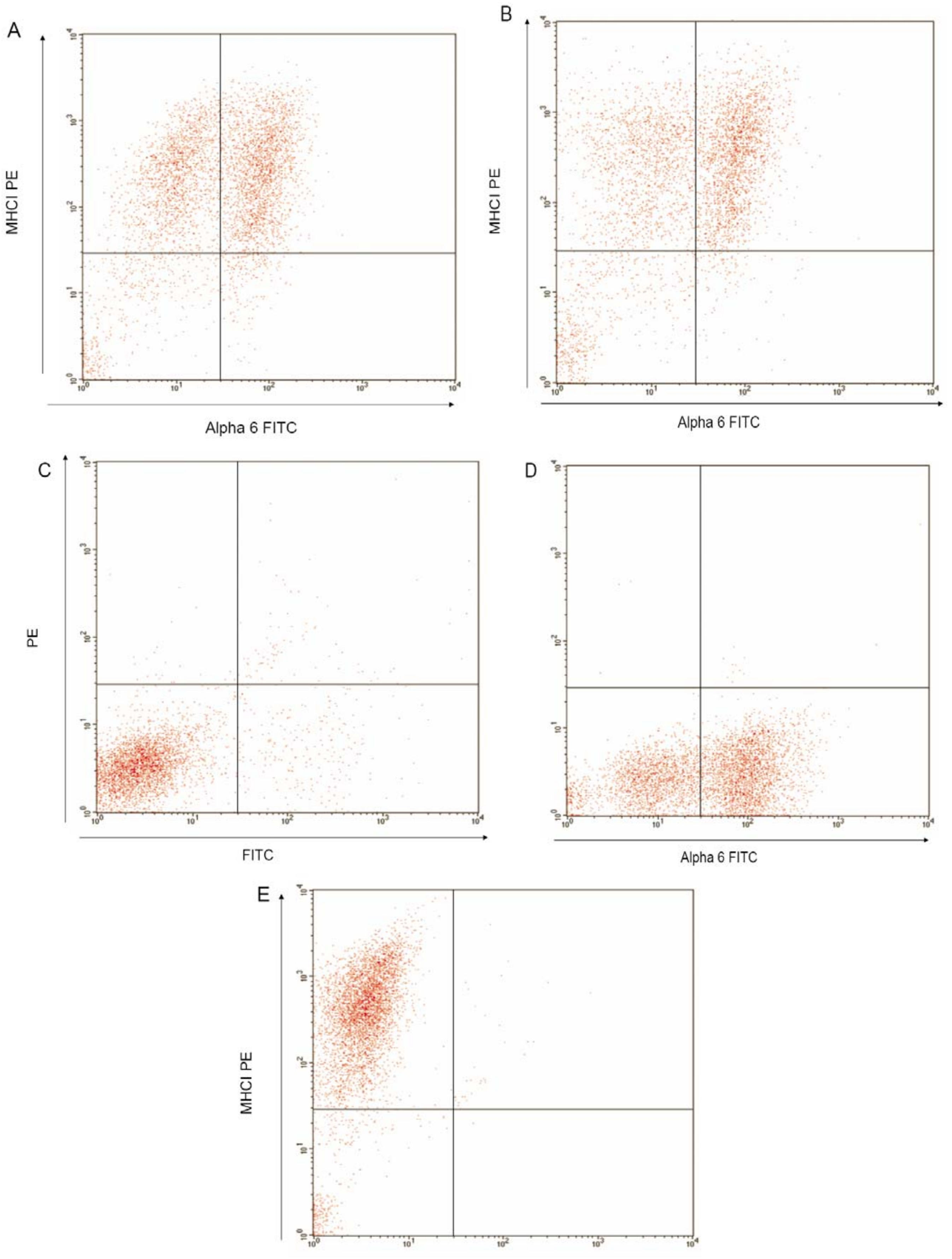

Gates in A & B were set using isotype control and single color positive controls (C-E). (A) Figure showing flow cytometry analysis of the expression of MHCI and α6 integrin on keratinocytes sorted by FACS and used in the first microarray. (B) Figure showing flow cytometry analysis of the expression of MHCI and α6 integrin on keratinocytes sorted by FACS and used in the second microarray. Cells in the lower right quadrant are $\alpha6^{+}$/$MHCI^{-}$. Cells in the upper right quadrant are $\alpha6^{+}$/$MHCI^{+}$. (C) Representative isotype control. (D) Representative single color positive control for FITC. (E) Representative single color positive control for PE.
