# Supplementary material for: Transcriptional profiling of putative human epithelial stem cells
Source: BMC Genomics. 2008 Jul 30;9:359. doi: 10.1186/1471-2164-9-359 (PMC2536675; doi:10.1186/1471-2164-9-359)
Supplement: Additional file 8 — List of the genes that are differentially expressed in α6+/MHCI+ cells and α6+/MHCIcells and are consistently upregulated or downregulated ≥ 2 fold in both arrays. "-"sign indicates that the gene is upregulated in α6+/MHCI- cells. The numbers that show the difference in the level of gene expression are in log2 scale. [file 1471-2164-9-359-S8.pdf]

Probe Set	Exp. 1 Signal Log Ratio	Exp. 2 Signal Log Ratio	Description / Product	Gene Symbol
200041_s_at	1.7	1	HLA-B associated transcript-1	D6S81E
200644_at	-1.8	-1	macrophage myristoylated alanine-rich C kinasesubstrate	MACMARCKS
200692_s_at	1.3	2.7	heat shock 70kD protein 9B (mortalin-2)	HSPA9B
200762_at	-1.9	-1.9	dihydropyrimidinase-like 2	DPYSL2
200806_s_at	1.8	1.7	heat shock 60kD protein 1 (chaperonin)	HSPD1
200883_at	1.4	1	ubiquinol-cytochrome c reductase core protein II	UQCRC2
200900_s_at	2.8	1.4	mannose-6-phosphate receptor (cation dependent)	M6PR
200993_at	1	1.2	RAN binding protein 7	RANBP7
201014_s_at	1.4	2	multifunctional polypeptide similar to SAICARsynthetase and AIR carboxylase	ADE2H1
201068_s_at	1	1	proteasome (prosome, macropain) 26S subunit,ATPase, 2	PSMC2
201110_s_at	-2.3	-1.9	thrombospondin 1	THBS1
201112_s_at	1.1	2.1	chromosome segregation 1 (yeast homolog)-like	CSE1L
201117_s_at	-1.9	-1.3	carboxypeptidase E precursor	CPE
201163_s_at	-2.2	-1.6	insulin-like growth factor binding protein 7	IGFBP7
201198_s_at	1.2	1	proteasome (prosome, macropain) 26S subunit, non-ATPase, 1	PSMD1
201291_s_at	4.5	2.1	topoisomerase (DNA) II alpha (170kD)	TOP2A
201292_at	2	2.1	topoisomerase (DNA) II alpha (170kD)	TOP2A
201506_at	1.2	1.3	transforming growth factor, beta-induced, 68kD	TGFBI
201577_at	1.2	1.2	non-metastatic cells 1 protein	NME1
201725_at	1.4	1.1	D123 gene product	D123
201811_x_at	-1	-1.2	SH3-domain binding protein 5 (BTK-associated)	SH3BP5
201831_s_at	3.1	4.4	vesicle docking protein p115	P115
201859_at	-1.6	-1.1	proteoglycan 1, secretory granule	PRG1
201897_s_at	1.1	1.6	CDC28 protein kinase 1	CKS1
201946_s_at	1.2	2.6	chaperonin containing TCP1, subunit 2 (beta)	CCT2
201970_s_at	1.7	2.9	nuclear autoantigenic sperm protein(histone-binding)	NASP
202157_s_at	-1	-1.2	RNA-binding protein BRUNOL3	BRUNOL3
202403_s_at	-1.3	-2	collagen, type I, alpha 2	COL1A2
202436_s_at	-2.2	-1.2	cytochrome P450, subfamily I (dioxin-inducible), polypeptide 1 (glaucoma 3, primary infantile)	CYP1B1
202437_s_at	-3.5	-2.7	cytochrome P450, subfamily I (dioxin-inducible),polypeptide 1	CYP1B1
202546_at	1	3.2	vesicle-associated membrane protein 8	VAMP8
202572_s_at	-1.1	-1.2	Homo sapiens KIAA0964 protein (KIAA0964), mRNA.	KIAA0964
202908_at	-1.1	-1.2	Wolfram syndrome protein	WFS1
203103_s_at	1.1	1.6	nuclear matrix protein NMP200 related tosplicing factor PRP19	NMP200
203203_s_at	1.1	1.4	HIV-1 rev binding protein 2	HRB2
203213_at	1.7	1.5	cell division cycle 2, G1 to S and G2 to M	CDC2
203345_s_at	1.3	1.7	putative DNA binding protein	M96
203554_x_at	1.1	1	pituitary tumor-transforming protein 1	PTTG1
203603_s_at	-2.2	-1.6	zinc finger homeobox 1B	ZFHX1B
203697_at	-3.9	-3.2	Human Fritz mRNA, complete cds.	FRZB
203698_s_at	-2.8	-2.7	frizzled-related protein	FRZB
203706_s_at	-1.1	-1.5	frizzled 7	FZD7
203764_at	1.9	1.4	KIAA0008 gene product	KIAA0008
203903_s_at	-1.2	-1.8	hephaestin	HEPH
204154_at	-1.4	-1.1	cysteine dioxygenase, type I	CDO1
204162_at	2.9	1.9	highly expressed in cancer, rich in leucineheptad repeats	HEC
204197_s_at	-1.6	-1.1	runt-related transcription factor 3	RUNX3
204237_at	-1.2	-1	CED-6 protein	CED-6
204271_s_at	-2	-1.3	endothelin receptor	ETs
204273_at	-2.8	-1.3	endothelin receptor type B, isoform 1	EDNRB
204464_s_at	-1.1	-1.4	endothelin receptor type A	EDNRA
204529_s_at	-1.8	-2.2	KIAA0808 gene product	KIAA0808
204602_at	-1.3	-1.9	dickkopf (Xenopus laevis) homolog 1	DKK1
204620_s_at	-2	-1.4	chondroitin sulfate proteoglycan 2 (versican)	CSPG2
204712_at	-6.6	-3.8	Wnt inhibitory factor-1	WIF-1
204766_s_at	3.7	3.3	nudix (nucleoside diphosphate linked moietyX)-type motif 1	NUDT1
204777_s_at	-2	-1.2	T-cell differentiation protein MAL, isoform a	MAL
204822_at	1.1	1.1	TTK protein kinase	TTK
204836_at	-2	-1.9	glycine dehydrogenase (decarboxylating; glycinedecarboxylase, glycine cleavage system protein P)	GLDC
204869_at	-1.6	-1.7	proprotein convertase subtilisinkexin type 2	PCSK2
204948_s_at	-1.1	-1.9	follistatin isoform FST344 precursor	FST
204962_s_at	1.5	1.7	centromere protein A	CENPA

205001_s_at	1.3	1.6	dead box, Y isoform	DBY
205064_at	-1.5	-1.3	small proline-rich protein 1B (cornifin)	SPRR1B
205066_s_at	-1.6	-2.1	ectonucleotide pyrophosphatasephosphodiesterase1	ENPP1
205170_at	-1.5	-1.4	signal transducer and activator of transcription2, 113kD	STAT2
205337_at	-2.5	-1.2	dopachrome tautomerase (dopachrome delta-isomerase, tyrosine-related protein 2)	DCT
205345_at	1	2.5	BRCA1 associated RING domain 1	BARD1
205383_s_at	-1.1	-1.5	zinc finger protein 288	ZNF288
205404_at	-2.1	-2.3	hydroxysteroid (11-beta) dehydrogenase 1	HSD11B1
205489_at	-1	-1.5	crystallin, mu	CRYM
205590_at	-1.3	-2.6	RAS guanyl releasing protein 1	RASGRP1
205659_at	-1.6	-2.5	histone deacetylase 7B	HDAC7B-PENDING
205694_at	-2.3	-1.5	tyrosinase-related protein 1	TYRP1
205752_s_at	-1	-1.2	glutathione S-transferase M5	GSTM5
205794_s_at	-1.8	-1.6	neuro-oncological ventral antigen 1, isoform 1	NOVA1
205848_at	-2.1	-2.6	growth arrest-specific 2	GAS2
206002_at	-2.1	-2.3	G protein-coupled receptor 64	GPR64
206140_at	-5	-4.7	LIM homeobox protein 2	LHX2
206144_at	-1.1	-1	BAI1-associated protein 1	BAIAP1
206323_x_at	-1.2	-1.2	oligophrenin 1, Rho-GTPase acivating protein	OPHN1
206377_at	-1.3	-1.2	forkhead box F2	FOXF2
206404_at	-1	-1.7	fibroblast growth factor 9 (glia-activatingfactor)	FGF9
206421_s_at	1.8	4	serine (or cysteine) proteinase inhibitor, cladeB (ovalbumin), member 7	SERPINB7
206426_at	-1	-1.5	melan-A	MLANA
206465_at	-2.3	-3.5	very long-chain acyl-CoA synthetase; lipidosin	KIAA0631
206562_s_at	1.2	1.5	casein kinase 1, alpha 1	CSNK1A1
206766_at	-1.1	-1.4	integrin alpha 10 subunit	ITGA10
206953_s_at	-1	-1.6	latrophilin	KIAA0786
207016_s_at	-2.2	-2	Homo sapiens mRNA for RALDH2-T, complete cds.	RALDH2
207165_at	2.1	1.6	hyaluronan-mediated motility receptor (RHAMM)	HMMR
207173_x_at	-1.8	-2.4	OB-cadherin-1	osf-4
207175_at	-1.9	-2.3	adipose most abundant gene transcript 1	APM1
207184_at	-1.1	-1.5	solute carrier family 6 (neurotransmittertransporter, GABA), member 13	SLC6A13
207345_at	-2	-2.3	follistatin isoform FST317 precursor	FST
207369_at	-1.4	-1.8	bombesin-like receptor 3	BRS3
207594_s_at	-1	-1.7	synaptojanin 1	SYNJ1
208095_s_at	1.8	1.2	calciumcalmodulin-dependent protein kinase (CaMkinase) II gamma	CAMK2G
208309_s_at	1	1.4	mucosa associated lymphoid tissue lymphomatranslocation gene 1	MALT1
208650_s_at	-1.5	-1.1	CD24 antigen (small cell lung carcinoma cluster 4 antigen)	CD24
208662_s_at	1	1.4	tetratricopeptide repeat domain 3	TTC3
209024_s_at	1.2	1	NS1-associated protein 1	NSAP1
209040_s_at	1.5	3.4	proteasome subunit LMP7	LMP7
209140_x_at	1	2	Homo sapiens MHC HLA-B39 mRNA, complete cds.	HLA-B39
209168_at	-1.5	-1.3	glycoprotein M6B	GPM6B
209170_s_at	-2.5	-1.7	Homo sapiens m6b1 mRNA, complete cds.	m6b1
209360_s_at	-2.3	-1.4	AML1b protein	AML1
209392_at	-2	-1.7	autotaxin	ENPP2
209409_at	-1.1	-1.8	growth factor receptor-bound protein 10	KIAA0207
209435_s_at	-1.3	-1	Homo sapiens, clone MGC:3182, mRNA, complete cds.	
209512_at	1.1	2.5	Homo sapiens, Similar to RIKEN cDNA 2610207I16 gene, clone MGC:10940, mRNA, complete cds.	
209583_s_at	-2.9	-1.5	brain my033 protein	MOX2
209656_s_at	-1.1	-2.4	Homo sapiens mRNA; cDNA DKFZp761J17121 (from clone DKFZp761J17121); complete cds. / hypothetical protein	DKFZp761J17121
209771_x_at	-1.8	-1.6	CD24 antigen (small cell lung carcinoma cluster 4 antigen)	CD24
209773_s_at	1.4	2.3	ribonucleotide reductase M2 polypeptide	RRM2
209846_s_at	1	2	Similar to butyrophilin, subfamily 3, member A2	BTN3A2
209861_s_at	1.2	2.2	eIF-2-associated p67 homolog	MNPEP
210105_s_at	-1.6	-1.8	FYN oncogene related to SRC, FGR, YES	FYN
210115_at	2.8	1.3	ribosomal protein L39	
210147_at	-2.4	-2.6	mono-ADP-ribosyltransferase	htMART
210162_s_at	-1.1	-1.7	NF-Atc	NFATC1
210247_at	-1.3	-1	synapsin II	SYN2
210365_at	-1.8	-1.5	AML1a protein	AML1
210517_s_at	-1.1	-1	gravin	AKAP12
210759_s_at	1.2	1.1	prosomal protein P30-33K	pros-30
210800_at	-1.3	-1.2	Homo sapiens, clone MGC:12262, mRNA, complete cds.	TIMM8A
210946_at	-1	-1.3	type-2 phosphatidic acid phosphatase alpha-2	PAP2-a2
211071_s_at	-1.3	-1	Homo sapiens, ALL1-fused gene from chromosome 1q, clone MGC:4013, mRNA, complete cds.	
211075_s_at	1.1	1.8	integrin associated protein	

211276_at	-1.3	-2.4	brain my048 protein	
211297_s_at	1.5	2.3	protein serinethreonine kinase	CDK7
211340_s_at	-1	-1.5	MUC18 glycoprotein	MCAM
211454_x_at	-1.6	-1.3	Homo sapiens FKSG51 (FKSG51) mRNA, complete cds.	FKSG51
211466_at	-1.2	-1.1	nuclear factor I B3	NFIB
211600_at	-2.3	-1.5	glomerular epithelial protein 1	GLEPP1
211701_s_at	-1.3	-1.3	magphinin beta	TRO
211968_s_at	1.6	2.2	heat shock 90kD protein 1, alpha	HSPCA
211980_at	-1.9	-1.2	collagen, type IV, alpha 1	COL4A1
212007_at	1	2.4	UBX domain-containing 1	UBXDC1
212012_at	-2.1	-1.9	Melanoma associated gene	D2S448
212190_at	-1.8	-2.5	trinucleotide repeat containing 3	TNRC3
212230_at	-1.6	-1.3	Homo sapiens phosphatidic acid phosphatase type 2B (PPAP2B), mRNA	
212233_at	-1.6	-1.3	H.sapiens mRNA for 3UTR of unknown protein	
212353_at	-1.9	-1.3	KIAA1077 protein	KIAA1077
212419_at	-1	-2	Homo sapiens mRNA; cDNA DKFZp564L0822 (from clone DKFZp564L0822)	
212613_at	1.2	2.3	butyrophilin, subfamily 3, member A2	BTN3A2
212768_s_at	-4.5	-2	differentially expressed in hematopoietic lineages	GW112
212865_s_at	-1.3	-1.1	collagen, type XIV, alpha 1 (undulin)	COL14A1
212915_at	-2	-2.6	KIAA1095 protein	KIAA1095
212946_at	-1.1	-1.1	KIAA0564 protein	KIAA0564
213106_at	-1.3	-1.5	Homo sapiens clone 23664 and 23905 mRNA sequence	
213183_s_at	-1.5	-2.1	cyclin-dependent kinase inhibitor 1C (p57, Kip2)	CDKN1C
213241_at	-2.2	-1.5	Homo sapiens clone 23785 mRNA sequence	
213260_at	-1	-1.7	forkhead box C1	FOXC1
213275_x_at	-1.4	-1	cathepsin B	CTSB
213305_s_at	1	3.8	protein phosphatase 2A B56-gamma1	PP2A
213307_at	-1.8	-1.6	cortactin SH3 domain-binding protein	KIAA1022
213326_at	-1.1	-1.4	vesicle-associated membrane protein 1 (synaptobrevin 1)	VAMP1
213413_at	-1.4	-1.6	Homo sapiens cDNA FLJ13555 fis, clone PLACE1007677	
213447_at	-1	-1	imprinted in Prader-Willi syndrome	IPW
213486_at	-1	-1.6	hypothetical protein DKFZp761N09121	DKFZP761N09121
213496_at	-1.9	-1.3	KIAA0455 gene product	KIAA0455
213622_at	-1.5	-1.6	collagen, type IX, alpha 2	COL9A2
213649_at	1	1.6	splicing factor, arginineserine-rich 7 (35kD)	SFRS7
213729_at	1	1.1	Huntingtin-interacting protein A	HYPA
213836_s_at	-1.1	-2	KIAA1001 protein	KIAA1001
213880_at	-1.6	-2	G protein-coupled receptor 49	GPR49
214041_x_at	-1.6	-2.5	ribosomal protein L37a	RPL37A
214414_x_at	-2.7	-2.2	hemoglobin, alpha 1	HBA1
214582_at	-1.6	-1.6	2,3-cyclic nucleotide 3 phosphodiesterase	CNP
214610_at	-1.1	-1.3	cytochrome P450, subfamily XIB (steroid 11-beta-hydroxylase), polypeptide 1	CYP11B1
214710_s_at	2.2	1.8	cyclin B1	CCNB1
214741_at	1.2	3.4	zinc finger protein 131 (clone pHZ-10)	ZNF131
214772_at	-1.5	-1.6	G2 protein	G2
214861_at	-3.1	-1.5	gene amplified in squamous cell carcinoma 1; KIAA0780 protein	KIAA0780
215067_x_at	-1.4	-1	Homo sapiens cDNA FLJ12333 fis, clone MAMMA1002198, highly similar to THIOREDOXIN PEROXIDASE 1	
215204_at	-1.4	-1.1	Homo sapiens cDNA FLJ14090 fis, clone MAMMA1000264	
215306_at	-1.7	-2.3	Homo sapiens mRNA; cDNA DKFZp586N2020 (from clone DKFZp586N2020).	
215424_s_at	1.3	1.5	SKI-INTERACTING PROTEIN	SNW1
215479_at	-1.8	-1.4	Homo sapiens cDNA FLJ20780 fis, clone COL04256.	
215704_at	-2.4	-2	filaggrin	FLG
216034_at	-1.5	-1.9	Homo sapiens immunoglobulin lambda gene locus DNA, clone:288A10	
216061_x_at	-1	-1.1	platelet-derived growth factor beta polypeptide (simian sarcoma viral (v-sis) oncogene homolog)	PDGFB
216246_at	-1	-1.8	ribosomal protein S20	RPS20
216341_s_at	-1.7	-1.4	gonadotropin-releasing hormone receptor	GNRHR
216379_x_at	-1.8	-1.6	Homo sapiens cDNA FLJ20161 fis, clone COL09252, highly similar to L33930 Homo sapiens CD24 signal transducer mRNA.	
217897_at	-1.4	-1.6	FXYD domain-containing ion transport regulator 6	FXYD6
218123_at	1.4	1.5	Homo sapiens chromosome 21 open reading frame 59 (C21ORF59), mRNA. / hypothetical protein FLJ20467	C21ORF59
218211_s_at	-1.7	-1.6	hypothetical protein MGC2771	MGC2771
218309_at	-1.5	-1.3	hypothetical protein PRO1489	PRO1489
218349_s_at	1.1	2.3	hypothetical protein FLJ10036	FLJ10036
218502_s_at	-2	-1.6	trichorhinophalangeal syndrome I gene	TRPS1
218542_at	2	2.2	hypothetical protein FLJ10540	FLJ10540
218574_s_at	-1.3	-2.7	LIM and cysteine-rich domains 1	LMCD1
218622_at	1	1.6	hypothetical protein MGC5585	MGC5585

218663_at	3.9	3.6	chromosome condensation protein G	HCAP-G
218751_s_at	2.3	1.7	hypothetical protein FLJ11071	FLJ11071
218974_at	-2.2	-1.4	hypothetical protein FLJ10159	FLJ10159
219087_at	1.9	2.2	asporin (LRR class 1)	ASPN
219148_at	1.7	1.1	Homo sapiens PDZ-binding kinase; T-cell originated protein kinase (TOPK), mRNA.	TOPK
219212_at	1.1	1.1	heat shock protein hsp70-related protein	LOC51182
219304_s_at	-1.2	-1.4	spinal cord-derived growth factor-B	SCDGF-B
219555_s_at	1.8	3.5	uncharacterized bone marrow protein BM039	BM039
219572_at	-1.7	-1.4	hypothetical protein FLJ20761	FLJ20761
219645_at	-1.3	-1.6	skeletal muscle calsequestrin 1	CASQ1
219732_at	-1	-1.4	hypothetical protein FLJ20300	FLJ20300
219787_s_at	1	1.1	hypothetical protein FLJ10461	FLJ10461
219795_at	-1.1	-2.1	solute carrier family 6 (neurotransmittertransporter), member 14	SLC6A14
219918_s_at	1.4	1.3	hypothetical protein FLJ10517	FLJ10517
219951_s_at	-1.1	-1.2	hypothetical protein FLJ10600	FLJ10600
219979_s_at	1	2.1	Homo sapiens hypothetical protein (HSPC138), mRNA.	HSPC138
220115_s_at	-1.7	-1.2	cadherin 10, type 2 (T2-cadherin)	CDH10
220183_s_at	2.4	3.7	nudix (nucleoside diphosphate linked moietyX)-type motif 6	NUDT6
220193_at	-3.6	-1.3	hypothetical protein FLJ22938	FLJ22938
220494_s_at	1	3.6	lipopolysaccharide specific response-68 protein	LSR68
220533_at	-1.3	-1.4	hypothetical protein FLJ13385	FLJ13385
220625_s_at	-1.5	-2.1	Ets transcription factor ESE-2b	ELF5
220867_s_at	-1.2	-1	solute carrier family 24(sodiumpotassiumcalcium exchanger), member 2	SLC24A2
220897_at	-1.1	-1.5	hypothetical protein FLJ11556	FLJ11556
221437_s_at	1.3	2.6	mitochondrial ribosomal protein S15	MRPS15
221452_s_at	1.1	1	hypothetical protein MGC1223	MGC1223
221464_at	-1.7	-2	olfactory receptor, family 1, subfamily D,member 2	OR1D2
221524_s_at	1.1	1.2	Rag D	RAGD
221916_at	-1.3	-2	hypothetical protein	DKFZp434B0417
221986_s_at	1	2.5	hypothetical protein FLJ20059	FLJ20059
222037_at	1.2	3.4	minichromosome maintenance deficient (S. cerevisiae) 4	MCM4
222108_at	-1	-1.4	Human BAC clone GS1-99H8	
222113_s_at	-1	-1.3	epidermal growth factor receptor substrate EPS15R	EPS15R
266_s_at	-1.9	-1.5	Homo sapiens CD24 signal transducer mRNA, complete cds and 3 region	
38241_at	1	3.2	Human butyrophilin (BTF3) mRNA, complete cds	
39891_at	-1.1	-1		
44702_at	-1	-1.3		
45749_at	-1	-1.3		
48031_r_at	-1.5	-1.7		
58780_s_at	-1.2	-1.6		
60471_at	-1	-1.1		

List of the genes that are differentially expressed in α6^+/MHCI^+ cells and α6^+/MHCI^- cells and are consistently upregulated or downregulated ≥ 2 fold in both arrays. "–"sign indicates that the gene is upregulated in α6^+/MHCI^- cells. The numbers that show the difference in the level of gene expression are in log2 scale.
